# Algal and Cyanobacterial Hydrolysates as Serum-Reducing Supplements Across Mammalian, Avian and Fish Cells with Identification of Bioactive Chromopeptides

**DOI:** 10.3390/foods15162941

**Published:** 2026-08-21

**Authors:** Nikolina Sibinčić-Georgieva, Ljubodrag Aleksić, Nađa Grozdanić Stanisavljević, Lora Tubić, Boško Tanasković, Nikola Gligorijević, Marija Stojadinović, Simeon Minić

**Affiliations:** 1 Innovative Centre, Faculty of Chemistry, University of Belgrade, 11000 Belgrade, Serbia; nsibincic@chem.bg.ac.rs; 2 Department of Biochemistry & Center of Excellence for Molecular Food Sciences, Faculty of Chemistry, University of Belgrade, 11000 Belgrade, Serbia; 3 Institute for Oncology and Radiology of Serbia, 11000 Belgrade, Serbia; 4 Department of Chemistry, Institute of Chemistry, Technology, and Metallurgy, National Institute of the Republic of Serbia, University of Belgrade, 11000 Belgrade, Serbia

**Keywords:** algae, hydrolysate, cell culture, cell proliferation, serum-reducing supplement

## Abstract

Maintaining sustainable production of healthy and nutritious food represents a major global challenge. Cultivated meat could reduce the environmental impact of livestock production, but it remains costly, largely due to the expense of fetal bovine serum, a common cell culture media supplement. Algal and cyanobacterial proteins, particularly phycobiliproteins, represent promising sustainable sources of potentially bioactive peptides that may contribute to the development of serum-reduced or serum-free culture media. This study investigated the potential of hydrolysates prepared using the protein extracts of the red alga *Porphyra haitanensis* and the cyanobacterium *Arthrospira platensis* as functional supplements for cell cultivation in a low-serum environment. Twelve hydrolysates were generated by treating protein extracts from *Arthrospira platensis* and *Porphyra haitanensis* with six proteolytic enzymes: pepsin, trypsin, chymotrypsin, subtilisin, thermolysin and proteinase K. The effects of these hydrolysates were studied in three cell models of mammalian (CHO-K1), avian (QM7), and fish (MACK1) origin. The effects of hydrolysates on cell viability, proliferation, and antioxidant activity were evaluated. *A. platensis*–chymotrypsin hydrolysate significantly improved QM7 cell viability compared to control in both 80% reduced-serum and tryptose phosphate broth conditions (*p* ≤ 0.0001). Additionally, peptide composition analysis and identification of amino acid sequences provided preliminary insights into potentially bioactive chromopeptides associated with the maintenance and promotion of cellular growth. Algae/cyanobacteria-derived hydrolysates positively regulated cell viability and proliferation in serum-reduced conditions, although effects were dependent on the algal species, enzymatic treatment, and cell type. These findings highlight their potential as sustainable supplements for alternative cell culture media.

## 1. Introduction

The global population is growing rapidly and surpassed 8 billion people on 15 November 2022, according to the United Nations Population Fund (UNFPA) [1]. Despite this growth, the Food and Agriculture Organization (FAO) estimates that approximately 713–757 million people may have faced hunger in 2023. Additionally, about 2.33 billion people globally experience moderate or severe food insecurity, with 71.5% of people unable to afford a healthy diet living in low-income countries [2]. Projections indicate that the world population could reach 9 billion by 2050, placing even greater pressure on food supplies and posing significant challenges to global food security. Addressing these challenges requires the implementation of practical and sustainable agricultural strategies that enhance food production, improve nutrition, and strengthen food security in an effort to eliminate hunger worldwide [3]. While population growth, urbanization, and increased income together pose great challenges to agriculture and food systems, the natural resources necessary to support them are already strained. Agriculture is an important factor in environmental issues like pollution, land degradation, climate change, the loss of biodiversity, and greenhouse gas emissions, and this is particularly related to animal husbandry and meat production [4,5]. The demand for animal-sourced meat is expected to rise by 73% in 2050 [6]. Animal husbandry and consumption contribute to numerous environmental pressures that threaten sustainability, including greenhouse gas emissions (GHGEs), land use and degradation, scarcity-weighted water consumption, nutrient pollution, fertilizer and pesticide use, and food waste generated throughout the supply chain. Addressing these is essential for mitigating climate change and promoting the efficient use of natural resources. Given that agriculture, especially the production of animal-based foods, accounts for approximately 30% of global greenhouse gas emissions, 70% of freshwater use, and more than one-third of potentially cultivable land use, its environmental footprint is a critical consideration in sustainability efforts [7].

In recent years, two alternative protein sources have emerged as prominent substitutes for traditional meat. Both have advantages and disadvantages when compared to one another and to traditional meat. The first alternative is plant-based meat analogs, composed of plant proteins, and the other, more recent, is called cultivated meat—meat grown from muscle or fat cells [8]. Whether alternative food products are better than animal products depends on how the comparison is made, how the foods are produced, and which animal product is used as the benchmark. Critical scientific assessments are necessary to obtain objective insight into these questions. To feed a growing global population sustainably, ongoing technological improvements will be needed in both traditional agriculture and alternative food production systems. Traditional and alternative meat technologies should not exclude one another but mutually support each other to efficiently answer increasing demands for nutritious foods [9].

Cultivated meat is grown in laboratories using cell culture techniques. This technology offers several potential advantages compared to traditional meat, including improved sustainability, a lower environmental impact, better resource utilization, and more efficient production. These characteristics make cultivated meat a promising eco-friendly alternative to traditional meat, addressing concerns such as land and water usage, as well as greenhouse gas emissions [10]. The production of cultivated meat involves the cultivation of appropriate muscle satellite cells and other cell types, supplying them with nutritious cell growth media supplemented with growth factors and signaling molecules, utilizing scaffolds to obtain certain shapes that mimic meat of animal origin [10,11]. The initial goal is to produce cultivated meat using stem cells obtained directly from the animals, while the final goal is to develop and use cell lines that would be independent of animals [12]. While the environmental benefits of cultivated meat sound promising, this technology will need to overcome several major obstacles before this product can be mass produced on a large scale. Those obstacles are the cost of production, safety regulations, and consumer acceptance [13,14].

Since its conceptualization, significant progress has been made in the field of meat cultivation. However, a major obstacle hindering the scale-up of this technology is the persistently high processing price. Nevertheless, a recent study made a price projection on a large scale if planned advancements were to be achieved regarding cell culture media and estimated that 1 kg of cultivated meat could cost $63 (if produced in California, USA), which is a significant advancement from 2013 when the same amount would have cost $2.3 million. The same study identified cell culture media, bioreactors, and labor as major influencers of production costs, accounting for about 80% of the total cost of the final product [12]. Serum-based cell culture media are still widely used for cell culturing in general, which defeats the purpose of meat cultivation, especially regarding animal well-being, considering how fetal bovine serum is obtained. Serum contains micronutrients, trace elements, attachment factors, hormones, growth factors, and stabilizers, all important for cell growth. On the other hand, its usage increases contamination risks from viruses and prions [15], and batch-to-batch variability is another limitation [11].

Algae and cyanobacteria are promising sources of nutrients such as proteins, amino acids, carbohydrates, lipids, minerals, and vitamins [16]. Their use in cultivated meat production can also be coupled with other sustainable practices, including CO_2_ fixation and waste media recycling [17]. Circular cultivation systems, where algae and mammalian cells reuse growth media with appropriate modifications, are also being explored [18,19]. While few studies that investigate algal- and cyanobacterial-derived products as alternatives to fetal bovine serum (FBS) for cultivated meat production have been published [20,21,22,23,24], the cyanobacterium *A. platensis* (commonly known as microalga Spirulina) and the red macroalga *P. haitanensis* have been widely used as a high-protein supplement in human nutrition, aquaculture, and for nutraceutical purposes. Their vivid color originates from light-harvesting phycobiliproteins (PBPs), specifically from covalently bound (via thioether bond) tetrapyrrole chromophores (phycobilins). Relevant examples are C-phycocyanin (C-PC) and allophycocyanin (APC) from Spirulina, and R-Phycoerythrin (R-PE) and R-Phycocyanin (R-PC) from *Porphyra* [25]. Numerous studies have shown the potent bioactive potential of algal and cyanobacterial PBPs [26,27,28]. In a previous study, we tested the potential of using algal and cyanobacterial extracts to reduce or replace serum in media for cellular meat cultivation [20]. It was shown that the best extract was the one from *A. platensis*, which supported cell growth through multiple passages.

In this study, we aimed to increase the diversity of algal and cyanobacterial extracts by using various proteases to hydrolyze PBPs and generate diverse peptide mixtures. It is well known that enzymatic hydrolysis of food proteins liberates structurally diverse bioactive peptides with antimicrobial, metal-binding, antioxidative and growth-promoting activities [29,30], which could potentially improve the proliferation and differentiation of muscle cells for meat cultivation [31]. However, the generally poor water solubility of industrially relevant plant proteins [32] and limited knowledge of the structure–activity relationships of the resulting peptides restrict their potential application as serum-reducing supplements or full-serum alternatives. In this context, hydrolysis of water-soluble algal PBPs could generate not only “regular” peptides, but also chromopeptides, which may have greater bioactive potential due to their enhanced antioxidative and metal-binding properties [33]. Given their low molecular weight, they may have favorable properties for cellular uptake and could potentially function as signaling molecules. In this context, we explored the potential of algal and cyanobacterial hydrolysates as serum-reducing supplements for cell culture media used in meat cultivation. Phycobiliproteins from cyanobacteria *Arthrospira platensis* (*A. platensis*) and red macroalgae *Porphyra haitanensis* (*P. haitanensis*) were digested using a selection of proteases, followed by the partial purification of hydrolysates using size exclusion chromatography and tangential flow filtration. The ability of hydrolysates thus obtained to promote the viability and growth of CHO, QM7, and MACK-1 cell lines in serum-reduced conditions was tested. The hydrolysates with the highest potential in promoting cell growth and viability were further purified by reversed-phase high-performance liquid chromatography, followed by the determination of the sequences of purified chromopeptides by tandem mass spectrometry.

## 2. Materials and Methods

### 2.1. Materials

Fetal Bovine Serum (FBS) of South American origin (cat. no. A5256701; Lot 2781406) and Tryptose Phosphate Broth (TPB, 18050039) were purchased from Gibco (Grand Island, New York, NY, USA). Recombinant human fibroblast growth factor 2 (FGF-2) (cat. no. HZ-1285, Lot 110-03CC-04, ≥6.00 × 10^6^ U/mg) was purchased from Proteintech Group (Rosemont, IL, USA). Ham’s F12 Kaighn’s modification media (SH30526.01) was purchased from Cytiva (Marlborough, MA, USA). L-15 Medium Leibovitz (L1518), Medium 199 (M4530), 100× penicillin–streptomycin (P07B1), 1 M HEPES buffer solution (H0887), 7-hydroxy-3H-phenoxazin-3-one-10-oxide (resazurin); 3-amino-7-dimethylamino-2-methylphenazine hydrochloride (neutral red, NR), trypan blue solution, 0.25% Trypsin-EDTA, 2,2′-azino-bis (3-ethylbenzothiazoline-6-sulfonic acid) (ABTS), 6-hydroxy-2,5,7,8-tetramethylchroman-2-carboxylic acid (Trolox) and all other chemicals were supplied by Sigma-Aldrich, Merck (Darmstadt, Germany). Cells were maintained in T-75 and T-25 BioLite™ tissue culture-treated flasks with filter caps from Thermo Fisher Scientific, Inc. (Waltham, MA, USA). Cell-based experiments were performed in clear, flat-bottom 96-well, 48-well, and 12-well BioLite™Plastic sterile labware (Thermo Fisher Scientific, Inc.). Food-grade *Arthrospira platensis* powder was purchased from We Are One (Sombor, Serbia). Food-grade *Porphyra haitanensis* was purchased from Fujian Friday Trading Co., Ltd. (Fuzhou, China). Chymotrypsin (Art.-Nr. 0238.9, ≥1000 USP-U/mg) was purchased from Carl Roth GmbH + Co. KG (Karlsruhe, Germany). Pepsin (P7000), thermolysin (P1512), and subtilisin (P5380) were purchased from Sigma-Aldrich, Merck (Darmstadt, Germany). Trypsin (37292.02) was purchased from SERVA Electrophoresis GmbH (Heidelberg, Germany). Proteinase K (MB086) was purchased from HiMedia Laboratories LLC (Kennett Square, PA, USA).

### 2.2. Preparation and Quantification of Hydrolysates

#### 2.2.1. Preparation of Hydrolysates

Dry *A. platensis* and *P. haitanensis* powders were suspended in a 20 mM phosphate buffer, pH 7.0 (1 g of powdered sample per 20 mL of buffer). Extraction of phycobiliproteins was performed for 4 h with constant stirring at room temperature. The resulting slurry was centrifuged at 10,000× *g* for 30 min at 4 °C. Proteins in the supernatant were precipitated with ammonium sulfate (65% final concentration). The precipitate was dissolved in 5 mM phosphate buffer (pH 7.0) and dialyzed overnight against the same buffer. Following dialysis, partially purified PBPs were again centrifuged (see above). Concentrations of PBPs in supernatants were determined by recording their absorption spectra using an LLG-uniSPEC 4 spectrophotometer (LLG labware, Meckenheim, Germany) and calculated according to the following equations [25]:

[C-PC] (mg/mL) = (A_618_ − (0.474A_652_))/5.34

[A-PC] (mg/mL) = (A_652_ − (0.205A_618_))/5.09

[R-PC] (mg/mL) = 0.154(A_618_ − A_730_)

[R-PE] (mg/mL) = 0.1247((A_564_ − A_730_) − 0.4583(A_618_ − A_730_))

Digestion of partially purified PBPs was performed using six different proteases: pepsin, trypsin, chymotrypsin, subtilisin, thermolysin, and proteinase K. Two solutions were used as starting materials: one containing 5 mg/mL of PBPs from *A. platensis* and the other containing 2 mg/mL of PBPs from *P. haitanensis*. Pepsin digestion was performed after adjusting the pH of PBP samples to 2.0 using 1 M HCl. To digest the PBPs in the samples, 100 U/mg of pepsin was used. Digestion lasted 16 h at 37 °C under constant stirring at 200 rpm. The next day, the pH of the digestion mixture was adjusted to 7.0 using 300 mM NaHCO_3_ to inactivate pepsin.

Trypsin, chymotrypsin, thermolysin, and subtilisin digestions were performed in 20 mM Tris buffer, pH 8, with 5, 0.5, 3, and 0.3 U per mg of PBPs, respectively. The buffer for PBP digestion by chymotrypsin also included 1 mM CaCl_2_. Proteinase K digestion was performed in a 20 mM phosphate buffer, pH 7.2, with 5 U/mg of PBPs. Each digestion lasted 16 h at 37 °C under constant stirring at 200 rpm. Immediately after digestion, obtained chromopeptides were separated from undigested proteins and proteases using a Sephadex G-25 column (Cytiva, Marlborough, MA, USA). The same procedure was used for each sample. Samples (40 mL) were applied to the packed column (400 mL). Elution was performed using a 5 mM phosphate buffer (pH 7.0). Undigested PBPs were eluted in the first 225 mL and were discarded. Chromopeptides were then collected and concentrated by a tangential flow filtration system (MinimateEvo, Pall Corporation, Port Washington, NY, USA) using a membrane with a 1 kDa cut-off. The concentrated retentate enriched with chromopeptides was freeze-dried using an Alpha 2–4 LD plus freeze-dryer (Martin Christ, Osterode am Harz, Germany), dissolved in a minimum amount of Milli-Q water, and chromopeptide concentrations were determined. Samples were stored at −20 °C until use.

#### 2.2.2. Quantification of Chromopeptides

Chromopeptide quantification was performed by VIS absorption spectrophotometry using whole protease digests of PBPs as standards, with a known concentration of chromophores [34]. Standard curves were obtained after a serial dilution of digests (between 10 and 200 μM chromophore concentration for *A. platensis* digests, and between 5 and 100 μM for *P. haitanensis* digests) in 5 mM phosphate buffer (pH 7.0). The absorbance of standard solutions and isolated chromopeptides from *A. platensis*-derived PBPs was recorded between 580 and 590 nm and between 490 and 500 nm in the case of *P. haitanensis*.

### 2.3. Evaluation of the Antioxidant Activity of Hydrolysates

#### 2.3.1. ABTS^•+^ Radical Scavenging Assay

An ABTS radical scavenging assay was performed as previously described with minor modifications [35]. The ABTS radical cation (ABTS^•+^) was produced by mixing a 7 mM ABTS aqueous solution with a 2.5 mM potassium persulfate solution (1:1; *v*/*v*). The mixture was then incubated in the dark at room temperature overnight to allow the complete formation of the radical cation. The ABTS^•+^ working solution was prepared by diluting the stock solution using a 100 mM phosphate buffer (pH 7.0) to obtain an absorbance of 0.9 at 734 nm. The working solution (375 µL) was mixed with 125 µL of sample (hydrolysates) or Trolox at 10 µM concentration and incubated for 30 min. The obtained mixture was pipetted into a 96-well plate, and absorbance at 734 nm was measured.

#### 2.3.2. Reducing Power Assay

The reducing power of chromopeptides was measured according to the method of Oyaizu with modifications as described in Minic et al. [33]. To 50 μL of 0.2 M phosphate buffer (pH 6.6) and 50 μL of 1% potassium ferricyanide, 20 μL of sample or Trolox at 60 µM concentration was added. After incubation at 50 °C for 20 min, 25 μL of 20% trichloroacetic acid was added to the reaction mixture. To each incubated mixture, 145 μL of Milli-Q water and 12 μL of 0.1% FeCl_3_ were added. After 10 min, absorbance was measured at 700 nm. Reducing power, expressed as Trolox equivalents (TEs), was calculated by dividing the sample and Trolox absorbances.

### 2.4. Cell Lines and Culturing Conditions

Chinese hamster ovarian epithelial-like cells (CHO-K1) (CRL-2147) and quail muscle myoblasts (QM7) (CCL-61) were obtained from the American Type Culture Collection (ATCC) (Manassas, VA, USA). Atlantic mackerel myoblasts (MACK1) were purchased from Kerafast (Newark, CA, USA) (#ETU008-FP). CHO-K1 cells were cultivated in Ham’s F12 Kaighan’s modification media supplemented with 10% (*v*/*v*) FBS and 1% (*v*/*v*) penicillin–streptomycin at 37 °C in a humidified 5% CO_2_ atmosphere. CHO-K1 cells were used within passages 10 to 25. QM7 cells were maintained in Medium 199 with Earle’s BSS supplemented with 10% (*v*/*v*) FBS, 10% (*v*/*v*) TPB and 1% (*v*/*v*) penicillin–streptomycin at 37 °C in a humidified 5% CO_2_ atmosphere. QM7 cells were used between passages 22 and 36. MACK1 cells were cultivated in media composed of L-15 Medium Leibovitz, 20% (*v*/*v*) FBS, 10 ng/mL FGF-2, 20 mM HEPES, and 1% (*v*/*v*) penicillin–streptomycin at 27 °C in a CO_2_-free environment. FGF-2 was used at a higher concentration than originally recommended by the supplier for this cell line to account for the lower reported specific activity of the purchased product. MACK1 cells were used within passages 115 to 130.

### 2.5. Cell Viability Assays

#### 2.5.1. Resazurin Assay

The resazurin protocol was adapted from Kumar, Nagarajan, and Uchil [36] with modifications described in Sibinčić et al. [20]. CHO-K1, QM7, and MACK1 cells were seeded at 5000; 10,000; and 20,000 cells/well, respectively, in 96-well plates with 200 µL of complete media, and allowed to attach overnight. The following day, cells were centrifuged at 300× *g* for 5 min and washed with PBS to remove residual serum, then treated for 72 h with low-serum medium containing hydrolysates at concentrations of 1–20 µM. CHO-K1 and QM7 cells were tested in media supplemented with 1% FBS, whereas MACK1 cells were tested in 2% FBS-containing media, representing a 90% reduction in serum relative to each cell line’s routine maintenance conditions. For QM7 cells, a secondary screening was subsequently performed in modified culture conditions (2% FBS, 2% TPB). Untreated cells were included as a control. Following incubation, plates were centrifuged at 500× *g* for 10 min, and the medium was removed. Subsequently, 100 µL of serum-free medium containing 0.03 mg/mL resazurin was added to each well. CHO-K1 cells were incubated for approximately 1–2 h and QM7 cells for approximately 3–4 h at 37 °C in a humidified atmosphere containing 5% CO_2_, whereas MACK1 cells were incubated overnight at 27 °C in a CO_2_-free environment. After incubation, the resazurin-containing medium was transferred to black fluorescence-compatible 96-well plates (Sarstedt, Nümbrecht, Germany), and the fluorescence of the reduced product, resorufin, was measured using excitation and emission wavelengths of 530/25 nm and 590/35 nm, respectively. Cell viability was calculated by normalizing the fluorescence values of treated samples to those of untreated controls. All experimental conditions, including controls, were performed in triplicate.

#### 2.5.2. Neutral Red Uptake Assay

The neutral red uptake (NRU) assay was performed according to the protocol of Repetto, del Peso, and Zurita [37], with minor modifications as described in Sibinčić et al. [20]. CHO-K1, QM7, and MACK1 cells were seeded in the same conditions described for the resazurin assay and incubated for 72 h with hydrolysates in serum-reduced media (1% FBS for CHO-K1 and QM7 cells; 2% FBS for MACK1 cells). Following the incubation period, the culture medium was removed and 100 µL of neutral red (NR) solution (5 µg/mL in serum-free medium, prepared the day prior) was added to each well. Cells were incubated in the previously described conditions to allow dye uptake. The plates were then centrifuged at 500× *g* for 10 min, and the NR solution was discarded. The cells were washed once with 150 µL of PBS and centrifuged again at 500× *g* for 10 min. After the removal of PBS, intracellular NR was extracted by adding 150 µL of destaining solution (50% ethanol, 1% glacial acetic acid, *v*/*v*) to each well. The plates were shaken for at least 10 min or until complete dye extraction was achieved, yielding a homogeneous solution. The extracted dye was transferred to fluorescence-compatible microplates, and fluorescence was measured using excitation/emission wavelengths of 530/25 nm and 590/35 nm, respectively. Cell viability was determined by normalizing fluorescence values of treated samples to those of untreated controls. All experimental conditions, including controls, were performed in triplicate.

### 2.6. Trypan Blue Exclusion Assay

CHO-K1, QM7, and MACK1 cells were seeded at 5000; 15,000; and 20,000 cells/well in 48-well plates in 400 µL of complete medium and left to attach overnight. The next day, the cells were treated with selected algal hydrolysates (1–20 µM) in serum-reduced media (1% FBS for CHO-K1 and QM7 cells; 2% FBS for MACK1 cells). The cells were incubated for six days in media containing hydrolysates (seven days total), with a medium change after three days. On day seven, cell viability was assessed with the trypan blue (TB) exclusion assay. Prior to trypsinization, the plates were centrifuged at 500× *g* for 10 min to minimize potential cell loss. The culture medium was then removed, and the cells were detached using 100 µL of a 0.25% trypsin–EDTA solution. Trypsin activity was neutralized with 400 µL of fresh medium, and the cells were centrifuged at 500× *g* for 10 min. After supernatant removal, cell pellets were resuspended in 200 µL of fresh medium and mixed with a 0.4% (*w*/*v*) TB solution in PBS at a 1:4 (*v*/*v*) ratio. The cells were counted using a Neubauer hemocytometer under a light microscope, with viability assessed across all counting fields. The investigators were blinded during microscopy analysis. Cell morphology was monitored using the Millicell^®^ Digital Cell Imager MDCI10000 (Merck Millipore, Darmstadt, Germany). In all samples, cell viability was ≥95%. Fold change in cell number of treated cells and control cells was determined by dividing the number of viable cells at the time of passage (N) by the number of viable cells at the time of seeding (N_0_). The number of population doublings was calculated using the formula log2 (N/N_0_). All experiments were performed in triplicate. The cell media after the experiment were preserved for subsequent pH measurements, and cell morphology was monitored.

### 2.7. Separation of Chromopeptide Fractions from Hydrolysates by HPLC

Separation of the pepsin digest of C-PC was conducted on an HPLC Agilent 1260 system (Agilent, Santa Clara, CA, USA) as previously described [33] with slight modifications. Briefly, chromopeptides were separated using a semi-preparative Zorbax Eclipse XDB C-18 column (9.4 mm × 250 mm, 5 μm particles; Agilent, USA) connected with an HPLC system. The column was equilibrated with a solvent mixture containing 90% solvent A (0.1% formic acid in Milli-Q water) and 10% solvent B (0.1% formic acid in acetonitrile). Elution of peptides was done using gradient elution in the following order: 10% solvent B—one column volume, gradient from 10% to 20% solvent B—one column volume, gradient from 20% to 40% solvent B—twelve column volumes, gradient from 40% to 100% solvent B—two column volumes. The flow rate was 3 mL/min with simultaneous detection at 220, 500, and 620 nm. Each chromopeptide fraction (with absorbance at 500 or 620 nm) was collected and further analyzed by mass spectrometry.

### 2.8. Identification of Amino Acid Sequence of the Chromopeptides

Chromopeptides, separated by semi-preparative HPLC, were sequenced by high-resolution tandem mass spectrometry using an Orbitrap Exploris 240 (Thermo Fisher Scientific Inc., USA) mass spectrometer. Chromopeptide sequences were determined as previously described [31,33]. Ionization was performed in a positive mode on a heated electrospray ionization (HESI) probe. HESI parameters were: vaporizer temperature 50 °C, capillary temperature 275 °C, source voltage 3.7 kV, RF lens 70%, sheath and auxiliary gas flow 8 and 2 (arbitrary units), respectively. The acquisition time of spectra was 5 min per sample. Samples were injected directly with a flow rate of 10 μL/min. MS spectra were acquired between *m*/*z* 300 and *m*/*z* 3000. Ionized peptides were fragmented with CID (collision-induced dissociation) to obtain MS2 spectra. CID was performed with helium gas at a normalized collision energy of 30%, and the parent ions were activated at 30 ms. The ESI-MS and ESI-MS2 data were acquired with Xcalibur version 4.8 (Thermo Fisher Scientific Inc., USA). Chromopeptide identification was performed by manual de novo sequencing using NIST Mass and Fragment Calculator Software (Version 2.0; http://www.nist.gov/mml/bmd/bioanalytical/massfragcalc.cfm; URL accessed on 28 December 2025) to calculate the theoretical fragment masses of the input peptide sequences.

### 2.9. Statistical Analysis

All experiments were performed at least in two independent experiments and each experimental condition was tested in triplicate. More specifically, for cell-based experiments, this corresponded to three independent wells for each experimental condition Results are presented as mean ± SEM of triplicate runs from one representative experiment. Prior to ANOVA analyses, the normality of data distribution was assessed using the Shapiro–Wilk test. One-way ANOVA with Tukey’s multiple comparisons test was used to compare the antioxidant activities of different hydrolysates. One-way ANOVA with Dunnett’s multiple comparisons test was used to compare the viability/growth of cells at different concentrations of algal/cyanobacterial hydrolysates to the corresponding control/untreated cells. Additionally, two-way ANOVA with Tukey’s multiple comparisons test was used to evaluate differences among hydrolysates, with hydrolysate type and concentration as the two factors. The hydrolysate type × concentration interaction was tested in all two-way ANOVA analyses, and the corresponding interaction *p*-values are reported in the figure captions. In all statistical analyses, differences were considered significant if *p* < 0.05. Raw data and detailed statistical analysis are presented in Supplementary Materials (Tables S1–S28). GraphPad Prism 7.0 software (GraphPad, San Diego, CA, USA) was used for statistical analysis and graph creation.

## 3. Results

### 3.1. Antioxidant Capacity of Algal and Cyanobacterial Hydrolysates

As part of the wider characterization of the six algal/cyanobacterial hydrolysates, ABTS radical scavenging activity and reducing power were evaluated to gain insight into the antioxidant potential of these hydrolysates and their possible contribution to the effects observed in the cell-based assays.

**Figure 1:**
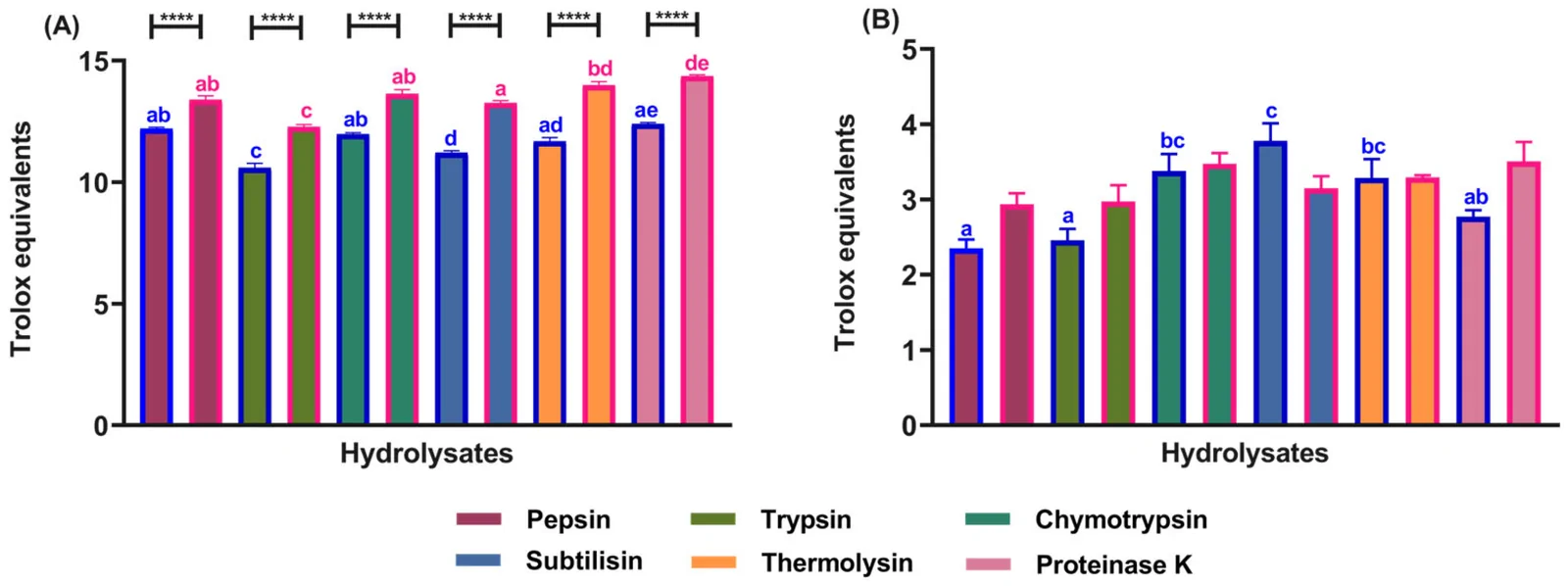
Antioxidant capacity of *A. platensis* and *P. haitanensis* hydrolysates was evaluated by (**A**) ABTS radical scavenging activity and (**B**) reducing power, with the results expressed as Trolox equivalents. Results are presented as mean ± SEM, *n* = 3 (triplicate runs from one representative experiment). Enzymes are color-coded as indicated in the legend to match the columns. Columns outlined in blue correspond to *A. platensis* hydrolysates and columns outlined in pink correspond to *P. haitanensis* hydrolysates. Different letters indicate statistically significant differences between hydrolysates within the same algae (one-way ANOVA followed by Tukey’s multiple comparisons test, *p* ≤ 0.05). Asterisks indicate statistically significant differences between *A. platensis* and *P. haitanensis* hydrolysates prepared with the same enzyme (**** *p* ≤ 0.0001).

As shown in Figure 1, the antioxidant capacities differed significantly between *A. platensis* and *P. haitanensis* hydrolysates, with *P. haitanensis* hydrolysates demonstrating significantly higher antioxidant capacity in the ABTS test (Figure 1A), but this trend is not observed in the reducing power test. Among the *A. platensis* hydrolysates, trypsin exhibited the lowest scavenging activity (10.6 Trolox equivalents (TE)), while pepsin, chymotrypsin, subtilisin and proteinase K showed comparable activities ranging between 11.7 and 12.4 TE. In *P. haitanensis* hydrolysates, proteinase K demonstrated the highest ABTS scavenging activity (14.4 TE), followed by thermolysin (14.0 TE), while pepsin, chymotrypsin and subtilisin showed comparable activities. Trypsin again exhibited the lowest activity (12.3 TE), though still considerably higher than any *A. platensis* hydrolysate. In the reducing power assay (Figure 1B), no significant interspecies differences were observed, in contrast to the ABTS test. Overall reducing power values were noticeably lower across all hydrolysates, ranging from 2.4 to 3.8 TE. *A. platensis* subtilisin hydrolysate showed the highest reducing power (3.8 TE), while the pepsin hydrolysates of both species showed the lowest values. Notably, pepsin hydrolysates did not show a consistent pattern between the two assays, having modest reducing power despite relatively high ABTS scavenging activity, particularly in *P. haitanensis*.

### 3.2. Cell Viability of CHO-K1, QM7 and MACK1 Cells Treated with Algal and Cyanobacterial Hydrolysates in Low-Serum Conditions

Cytotoxicity screening was performed with the aim of testing the ability of algal/cyanobacterial hydrolysates to promote the viability of mammalian, avian, and fish cells, as well as the concentrations at which they do this most effectively. Since fetal bovine serum (FBS) consists predominantly of proteins, particularly albumin, the peptide fractions—chromopeptides of the hydrolysates—were investigated as potential serum-reducing supplements. Therefore, hydrolysate supplementation was standardized based on total chromopeptide concentration (see Section 2.2.2). CHO-K1 and QM7 cells were routinely maintained in basal medium supplemented with 10% FBS, while MACK1 cells were cultivated in basal medium supplemented with 20% FBS (full medium composition is provided in Section 2). Although laminin (0.25 µg/cm^2^) is recommended as an ECM mimetic for adhesion, MACK1 cells were routinely maintained without laminin or any coating, with no visible cell detachment. Laminin was required only during cell thawing. For the cytotoxicity test, cells were passaged and seeded into 96-well plates containing complete medium without hydrolysates and allowed to attach overnight. An alternative approach, in which cells were seeded directly into low-serum medium supplemented with hydrolysates, was initially considered. However, this protocol was not suitable for the MACK1 cells, even when surfaces were coated with laminin-521, as it resulted in incomplete cell attachment and consequently increased variability in cell numbers between wells. Although preliminary observations suggested that this approach could be applied to CHO-K1 and QM7 cells, the same seeding and treatment protocol was used for all cell lines to ensure methodological consistency and comparability of the results across cultures. Cells were incubated with the hydrolysates in low-serum media for 72 h. This extended exposure period was selected instead of the conventional 24 h commonly used in cytotoxicity assays, as the objective was to identify hydrolysates that support long-term cell viability and proliferation. Cell viability was assessed by the resazurin test, which measures metabolic activity through the reduction in resazurin by viable cells. This assay was selected instead of the MTT assay, which is commonly regarded as the gold standard for in vitro cytotoxicity testing. In our experimental conditions, MACK1 cells exhibited limited metabolism of tetrazolium salts, resulting in insufficient sensitivity. A similar limitation was observed in another fish cell line, ZEM2S fibroblasts [20]. In contrast, the fluorescence-based readout of the resazurin assay provided greater sensitivity for detecting changes in cell viability.

**Figure 2:**
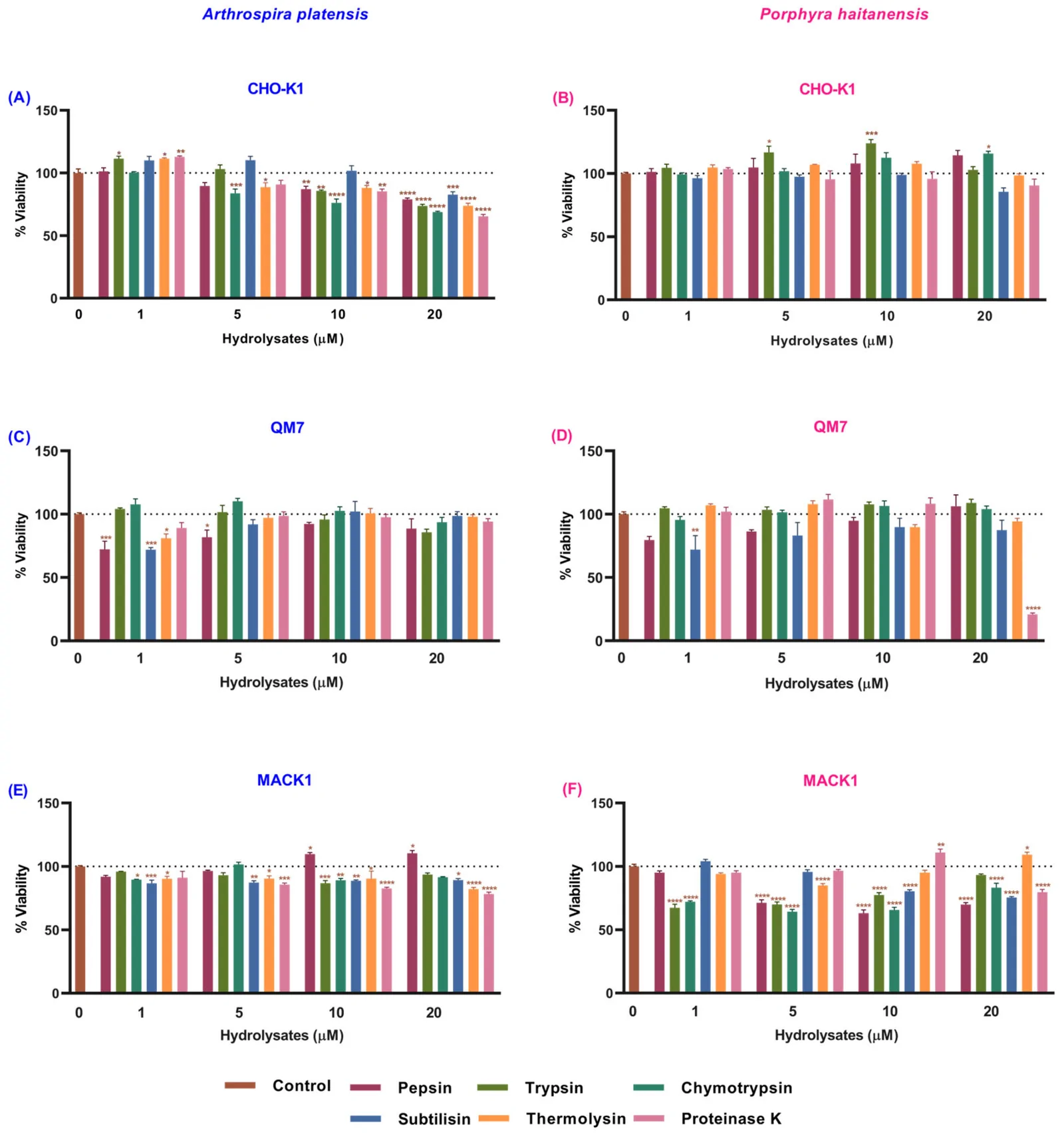
The viability of mammalian, avian, and fish cell lines treated with *A. platensis* and *P. haitanensis* hydrolysates were measured by the resazurin assay following 72 h of treatment and normalized to the untreated/control cells (100%). The results are presented as mean ± SEM, *n* = 3 (triplicate runs from one representative experiment). *A. platensis* (**A**,**C**,**E**) and *P. haitanensis* (**B**,**D**,**F**) hydrolysates were prepared using six proteolytic enzymes (pepsin, trypsin, chymotrypsin, subtilisin, thermolysin, and proteinase K) and tested at concentrations of 1, 5, 10, and 20 μM in CHO-K1 (**A**,**B**), QM7 (**C**,**D**), and MACK1 (**E**,**F**) cell lines. Hydrolysate concentrations are presented as µM based on total chromopeptide content. Enzyme names are color-coded as indicated in the legend to match the columns. Differences between individual treatment groups and the untreated control were evaluated by one-way ANOVA followed by Dunnett’s multiple comparisons test and were labeled with * (*p* ≤ 0.05), ** (*p* ≤ 0.01), *** (*p* ≤ 0.001), or **** (*p* ≤ 0.0001). Differences among hydrolysates across concentrations were analyzed by two-way ANOVA with hydrolysate type and concentration as factors, followed by Tukey’s multiple comparisons test (Tables S1–S28). The hydrolysate type × concentration interaction was significant in all panels (*p* ≤ 0.0002).

A total of twelve hydrolysates were prepared and tested: the extracts of two algal/cyanobacterial species, *A. platensis* and *P. haitanensis*, were each digested with six proteolytic enzymes: pepsin, trypsin, chymotrypsin, subtilisin, thermolysin and proteinase K. Figure 2 presents the results of the cytotoxicity screening performed by the resazurin assay, showing mean percent cell viability ± standard error of the mean (SEM) at hydrolysate concentrations of 1–20 μM. A general trend in the effect of the same algal hydrolysate across the mammalian, avian, and fish cell lines is difficult to identify, which is to be expected given the different origin of these cell lines. To provide a comprehensive and integrated view of our results, we aimed to compare data across multiple levels: between cell lines, between different algae species, between enzymes used for hydrolysis, and between algae–enzyme combinations. This is particularly important in the search for a universal serum-reducing supplement or full-serum replacement, since identifying alternatives suitable for only one cell line or cells of the same morphology addresses only part of the problem.

To improve readability and avoid the repetitive use of long hydrolysate names throughout the text, the hydrolysates are referred to by abbreviations throughout the text. Hydrolysates prepared from using *A. platensis* extracts are indicated as AP, while those derived from *P. haitanensis* are indicated as PH, followed by the enzyme used for digestion. For example, the pepsin hydrolysates are referred to as AP-pepsin and PH-pepsin, respectively.

In the CHO-K1 cell line, *A. platensis* (AP) and *P. haitanensis* (PH) hydrolysates showed distinct concentration- and enzyme-dependent effects on cell viability. For *A. platensis* (Figure 2A), the trypsin, chymotrypsin, and proteinase K hydrolysates showed a clear dose-dependent decrease in viability, with the lowest viability observed at the highest concentration tested (20 µM). At the lower concentrations (1 and 5 µM), trypsin and subtilisin hydrolysates produced the highest cell viability. In comparison, the subtilisin hydrolysate showed a more consistent effect across the tested concentration range. For *P. haitanensis* (Figure 2B), no significant differences in viability were observed at the lowest concentration (1 µM). At 5 and 10 µM, the PH-trypsin produced the highest effect. However, at the highest concentration (20 µM), the PH-trypsin-induced effect declined, whereas both the PH-pepsin and PH-chymotrypsin maintained their positive effects compared to the untreated control. These two hydrolysates were therefore selected for further testing. Overall, *P. haitanensis* hydrolysates showed stronger effects on cell viability than those prepared from *A. platensis*. Notably, even the weakest-performing *P. haitanensis* hydrolysates, prepared with thermolysin and proteinase K, resulted in higher cell viability than the corresponding hydrolysates of *A. platensis*. This difference was consistent across all concentrations, with *P. haitanensis* hydrolysates maintaining moderate effects throughout, while *A. platensis* hydrolysates showed a reduction in viability, particularly at the highest concentrations, where they appeared to have a mildly cytotoxic effect. Notably, PH-pepsin, which had a consistently moderate effect on viability in CHO-K1 cells, had a low effect on QM7 and MACK1 cell viability, suggesting a cell line-specific sensitivity to this hydrolysate.

In the QM7 cell line (Figure 2C,D), the effects of the hydrolysates differed from those observed in CHO-K1 cells. While *P. haitanensis* hydrolysates induced stronger positive effects on CHO-K1 cell viability compared to *A. platensis*, no comparable species-specific effect was observed in QM7 cell line. Interestingly, for QM7 cells, unlike CHO-K1 and MACK1 lines, four specific enzymatic hydrolysates (trypsin, chymotrypsin, thermolysin, and proteinase K) produced comparable effects regardless of algal species. This specific finding prompted a second re-evaluation (detailed in Section Re-Evaluation of Selected Candidates in QM7 Cells Under Reduced FBS/TPB Conditions). Minor algal species-specific differences in QM7 cells were only observed following pepsin and subtilisin treatments: AP-pepsin performed worse than PH-pepsin, whereas the opposite trend was noted for subtilisin (AP-subtilisin performed better than PH-subtilisin). However, these variations were not pronounced enough to be included in the subsequent re-evaluation.

In the MACK1 cell line, the response pattern differed from that observed in both CHO-K1 and QM7 cells. For *A. platensis* (Figure 2E), no significant differences in viability were observed between the hydrolysates, except for the pepsin hydrolysate, which produced the most favorable effect, with higher concentrations producing higher effects on viability. Overall, *A. platensis* hydrolysates showed moderate effects in MACK1 cells, with no significant cytotoxicity observed at any concentrations tested, which is comparable to the results obtained for QM7 cells. AP-pepsin, which had a moderate to low effect in viability in CHO-K1 and QM7 cells showed the strongest effect on MACK1 cell viability, suggesting the existence of a cell line-specific sensitivity to this hydrolysate. A marked difference between the impact of *P. haitanensis* digests on QM7 and CHO-K1 cell lines on the one hand, and MACK-1 cell line on the other (Figure 2F), was observed. PH-subtilisin showed clear dose-dependent effects on viability; however, the best effects were produced by PH-thermolysin and PH-proteinase K, which is comparable to the QM7 cell results.

Hydrolysates were selected for the second round of viability testing if they met at least one of the following criteria: (i) no reduction in cell viability at concentrations equal to or greater than 10 μM, indicating good cellular tolerance, or (ii) a consistent positive effect on cell viability across the entire concentration range.

Overall, the effect of the algal/cyanobacterial hydrolysates on cell viability depends strongly on the cell line. However, a similar response pattern was found between QM7 and MACK1 cells, which may be related to their similar morphology. In CHO-K1 cells, *P. haitanensis* hydrolysates showed higher overall effects compared to *A. platensis*, and AP-pepsin and AP-chymotrypsin were chosen for further testing since they showed the strongest effect at the highest concentration. In QM7 cells, this species-related difference was not observed, while the effects of trypsin, chymotrypsin, thermolysin, and proteinase K were indistinguishable, which led to a re-evaluation. In MACK1 cells, AP-pepsin was specifically chosen for further testing from among the tested *A. platensis* hydrolysates. However, the choice between different *P. haitanensis* hydrolysates was less obvious, as both PH-thermolysin and PH-proteinase K showed promising results. Since it was important to draw a connection between the results observed in QM7 cell lines on the one hand, and MACK1 cell line on the other (due to their morphological similarity), the final decision was made only after the QM7 re-evaluation.

#### Re-Evaluation of Selected Candidates in QM7 Cells Under Reduced FBS/TPB Conditions

QM7 culture medium includes 10% FBS and 10% TPB, making it more complex to study serum alternatives for this cell line. TPB mainly consists of a tryptose—casein digest—produced with pancreatic enzymes, providing free amino acids and short peptides for cell metabolism. This indicates that QM7 cell metabolism is more complex and that alternatives to TPB should be investigated alongside FBS alternatives. At this stage of our study, investigating TPB alternatives was not our main goal. However, due to difficulties in identifying the most promising hydrolysates for use in QM7 cells based on the results of the initial resazurin assay performed in a medium containing 1% FBS and 10% TPB, we decided to modify the experimental conditions and tested the selected hydrolysates in a medium containing of 2% FBS and 2% TPB. By reducing the concentration of both supplements, we aimed to more clearly observe the differences among the previously selected hydrolysates.

**Figure 3:**
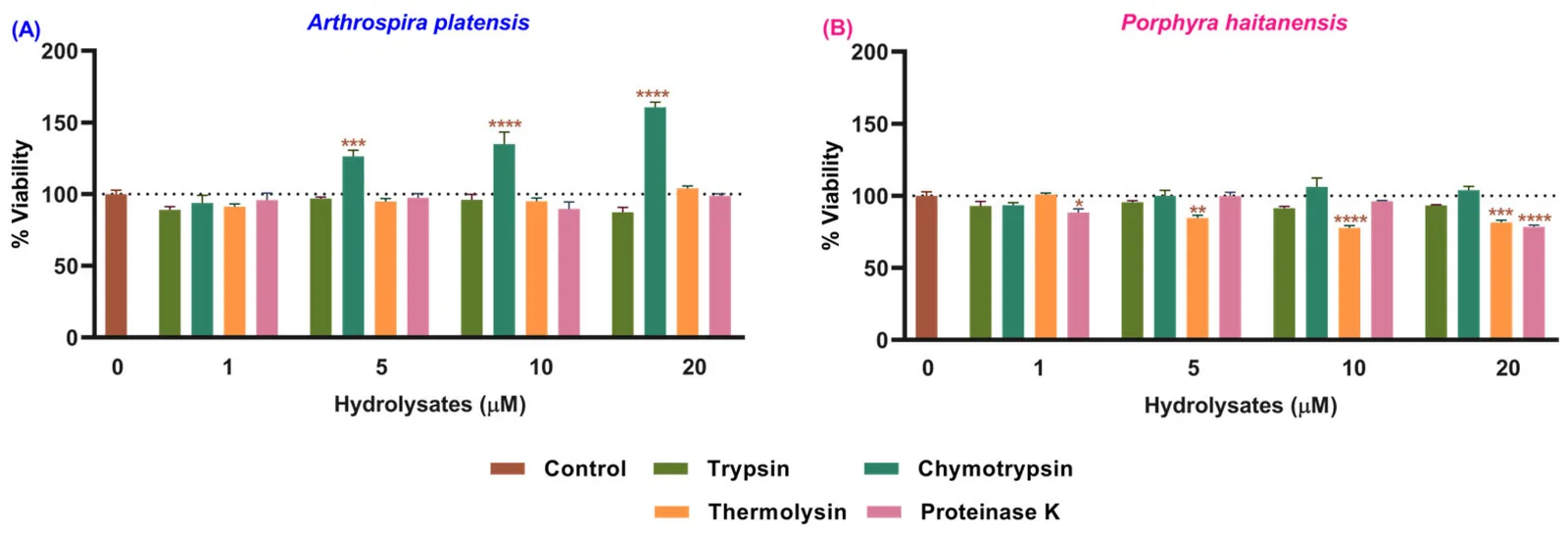
The viability of QM7 cells treated with selected (**A**) *A. platensis* and (**B**) *P. haitanensis* hydrolysates were measured by the resazurin test after 72 h of treatment and normalized to the untreated/control cells (100%). The results are presented as mean ± SEM, *n* = 3 (triplicate runs from one representative experiment). Hydrolysates of *A. platensis* and *P. haitanensis*—trypsin, chymotrypsin, thermolysin, and proteinase K—were re-tested in a second validation experiment to identify the best-performing hydrolysates, since the first test showed comparable effects across all four. Cells were cultured in media containing 2% FBS and 2% TPB, compared to 1% FBS and 10% TPB in the previous assay. Enzymes are color-coded as indicated in the legend, matching the corresponding columns. Differences between individual treatment groups and the untreated control were evaluated by one-way ANOVA followed by Dunnett’s multiple comparisons test and were labeled with * (*p* ≤ 0.05), ** (*p* ≤ 0.01), *** (*p* ≤ 0.001), or **** (*p* ≤ 0.0001). Differences among hydrolysates across concentrations were analyzed by two-way ANOVA with hydrolysate type and concentration as factors, followed by Tukey’s multiple comparisons test. The hydrolysate type × concentration interaction was significant in both panels (*p* ≤ 0.0001).

AP-trypsin, AP-thermolysin and AP-proteinase K showed no significant differences compared to the control, while AP-chymotrypsin showed a significant, dose-dependent effect, increasing cell viability by approximately 26%, 35%, and 61% relative to the control at 5, 10, and 20 μM, respectively (Figure 3A), making it a promising candidate for further testing. Although neither PH-thermolysin nor PH-proteinase K had strong effect (Figure 3B), we decided to choose one of them for further testing, since these two hydrolysates were relevant to the MACK1 results. Finally, we selected PH-proteinase K, as it showed an overall better result across the entire concentration range when compared to PH-thermolysin, both in MACK1 cells and in the QM7 re-evaluation.

Based on the results of both tests, and the criteria defined in the previous section, the final candidates were selected: PH-pepsin and PH-chymotrypsin for CHO-K1 cells; AP-chymotrypsin and PH-proteinase K for QM7 cells; and AP-pepsin and PH-proteinase K for MACK1 cells.

### 3.3. Cell Viability of CHO-K1, QM7 and MACK1 Cells Treated with Selected Algal and Cyanobacterial Hydrolysates Assessed by the Neutral Red Uptake Assay

**Figure 4:**
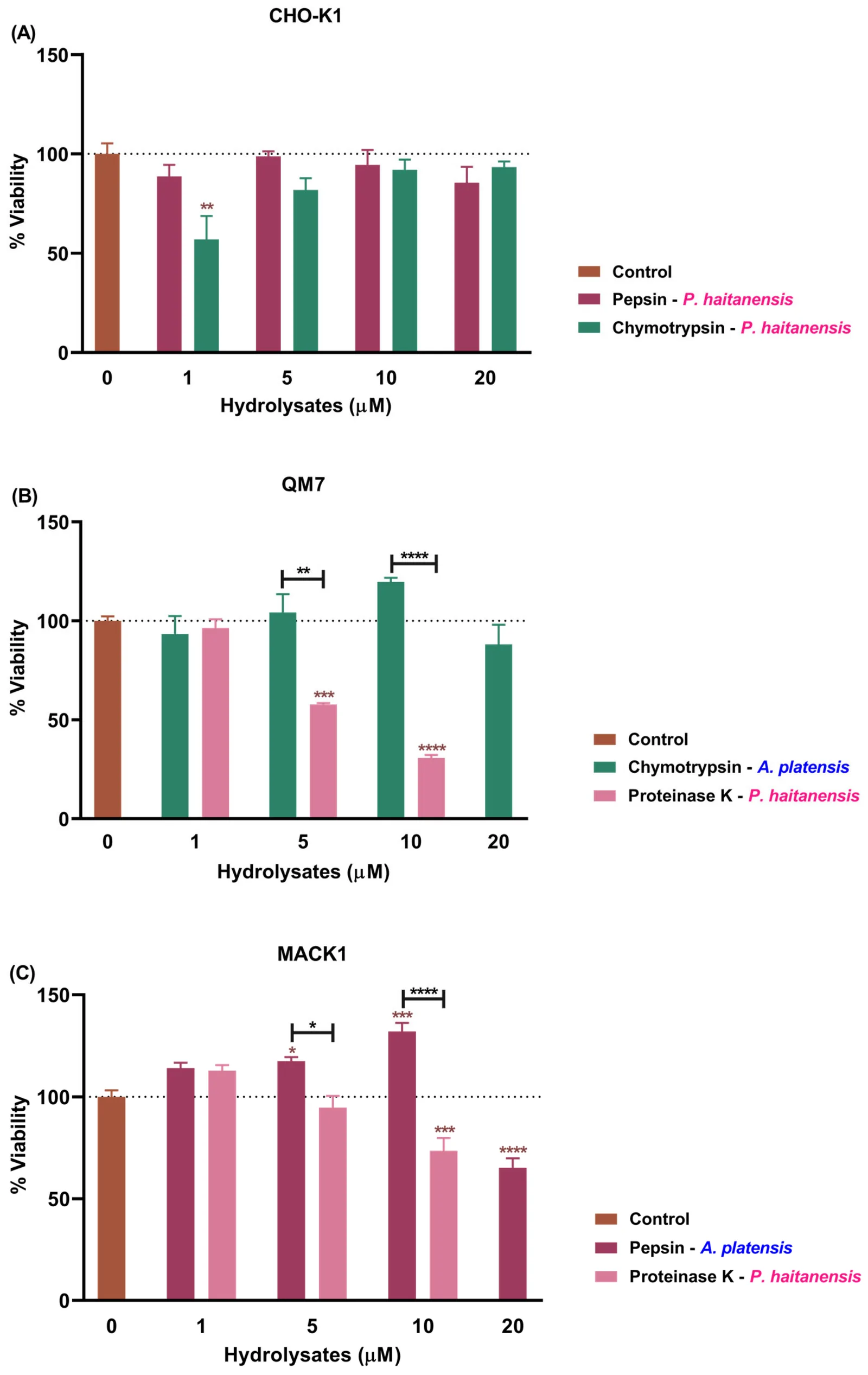
The viability of (**A**) CHO-K1, (**B**) QM7 and (**C**) MACK1 cells treated with selected *A. platensis* and *P. haitanensis* hydrolysates was measured by the neutral red uptake assay following 72 h of treatment and normalized to the untreated/control cells (100%). The results are presented as mean ± SEM, *n* = 3 (triplicate runs from one representative experiment). PH-pepsin and PH-chymotrypsin were tested in CHO-K1 cells. AP-chymotrypsin and PH-proteinase K were evaluated in QM7 cells, while AP-pepsin and PH-proteinase K were tested in MACK1 cells. Enzyme names are colour-coded as indicated in the legend to match the columns. Statistically significant differences between the control and the treatment groups are labeled with * (*p* ≤ 0.05), ** (*p* ≤ 0.01), *** (*p* ≤ 0.001), or **** (*p* ≤ 0.0001). Brown asterisks indicate significant differences vs. the untreated control (colour-matched to the control column) (one-way ANOVA followed by Dunnett’s multiple comparisons test); black asterisks indicate significant differences between the corresponding enzyme hydrolysates of the two algae species at the same concentration (two-way ANOVA followed by Tukey’s multiple comparisons test). A significant interaction between hydrolysate type and concentration was detected by two-way ANOVA in graph (**A**) (*p* ≤ 0.05), graph (**B**) (*p* ≤ 0.0001) and graph (**C**) (*p* ≤ 0.0001).

The most promising hydrolysates for each cell line were further evaluated by an additional viability assay, the NRU test, in identical experimental conditions as the resazurin test. Unlike the resazurin assay, which measures cellular redox potential, the NRU test provides an independent assessment of cell viability based on lysosomal integrity and function. In CHO-K1 cells, no significant differences in viability were observed between media supplemented with PH-pepsin and PH-chymotrypsin (Figure 4A), consistent with the resazurin results. Overall, both showed a moderate effect, with the pepsin hydrolysate leading to consistently higher viability across the tested concentration range. Similarly, in QM7 and MACK1 cells, the overall trends were preserved. AP-chymotrypsin produced significantly better effects than PH-proteinase K at higher concentrations in QM7 cells, as did AP-pepsin in MACK1 cells. In both cell lines, the effect of PH-proteinase K declined markedly with increasing concentration; for example, in QM7 cells, viability was reduced by approximately 42% and 69% relative to the control at 5 and 10 μM, respectively, while at 20 μM virtually no viable cells were detected (Figure 4B,C). The opposite trend was found for the other hydrolysates: viability improved as concentration increased. For MACK1 cells specifically, AP-pepsin significantly increased cell viability by approximately 18% and 32% relative to the control at 5 and 10 μM, respectively (Figure 4C). Importantly, these results once again confirmed the similar pattern between MACK1 and QM7 responses, with *A. platensis* hydrolysates producing better effects in both cell lines.

Opposite trends in the impact of the proteinase K hydrolysate on cell viability were observed in the resazurin and NRU assays. This discrepancy may be explained by the different parameters measured by the two assays: while the resazurin assay measures overall cellular metabolic activity, the NRU assay measures lysosomal membrane integrity, which is not necessarily a direct indicator of overall cell metabolic health. Even though there was a slight discrepancy between the results of two assays, the overall trends in cell line responses were preserved, and most importantly, the similar response pattern between QM7 and MACK1 responses was observed. In general, the obtained results and trends underline the importance of performing two mechanistically different assays for viability assessment in order to clearly identify the most potent hydrolysate.

### 3.4. Effects of Algal and Cyanobacterial Hydrolysates on CHO-K1, QM7 and MACK1 Cell Proliferation

This experiment aimed to evaluate the proliferative capacity of CHO-K1, QM7, and MACK1 cells in the presence of selected hydrolysates in reduced-serum conditions, corresponding to a 90% reduction in serum compared to standard culture medium conditions. A 7-day proliferation assay is more reliable for assessing long-term cellular responses than short-term cytotoxicity assays, as it measures how cells grow over several cell cycles. This enables the detection of changes in cell growth, rather than short-term effects on cell metabolism or viability only. Cells were seeded in 48-well plates in complete media (without laminin-521) to allow attachment overnight. The following day, cells were centrifuged to minimize cell loss, washed with PBS to remove trace FBS, and finally cultivated in low-serum medium supplemented with the selected hydrolysates for 144 h (168 h in total). At the end of the incubation period, cells were harvested and counted using trypan blue to assess the effects of the hydrolysates on cell proliferation. As previously described for the resazurin assay, the same seeding and treatment protocol involving overnight attachment in complete medium followed by treatment in low-serum medium was applied for this assay as well. This approach was adopted because direct seeding into low-serum medium resulted in incomplete attachment and increased variability in MACK1 cells; therefore, the same protocol was used across all cell lines to ensure methodological consistency and comparability of results.

**Figure 5:**
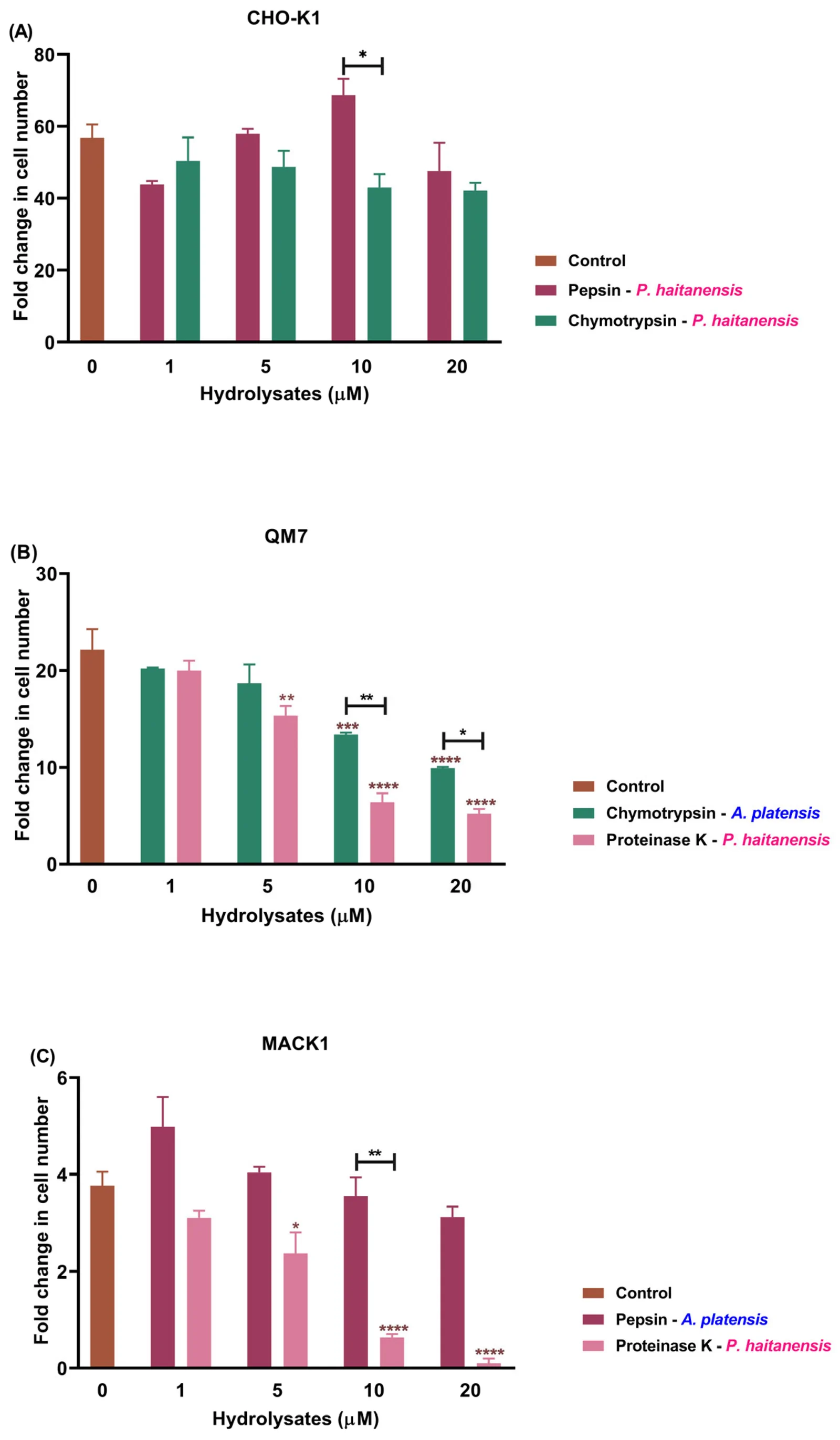
The fold change in cell number relative to day 0 of (**A**) CHO-K1, (**B**) QM7 and (**C**) MACK1 cells treated with selected hydrolysates was determined at day 7 by trypan blue exclusion counting. The results are presented as mean ± SEM, *n* = 3 (triplicate runs from one representative experiment). PH-pepsin and PH-chymotrypsin were tested in CHO-K1 cells. AP-chymotrypsin and PH-proteinase K were evaluated in QM7 cells, AP-pepsin and PH-proteinase K were tested in MACK1 cells. Enzyme names are color-coded as indicated in the legend to match the columns. Differences between individual treatment groups and the untreated control were evaluated by one-way ANOVA followed by Dunnett’s multiple comparisons test and were labeled with * (*p* ≤ 0.05), ** (*p* ≤ 0.01), *** (*p* ≤ 0.001), or **** (*p* ≤ 0.0001). Brown asterisks indicate significant differences vs. the untreated control (color-matched to the control column) (Dunnett’s multiple comparisons test); black asterisks indicate significant differences between the corresponding enzyme hydrolysates of the two algae species at the same concentration (two-way ANOVA followed by Tukey’s multiple comparisons test). A significant interaction between hydrolysate type and concentration was detected by two-way ANOVA in graph (**A**) (*p* ≤ 0.05) and graph (**B**) (*p* ≤ 0.05).

We decided to test the effects of the same hydrolysates in CHO-K1, QM7 and MACK1 cells. The proliferation assay was expected to provide clarification on the differences observed in the effect of proteinase K hydrolysates in QM7 and MACK1 cells. The results are presented as fold change in cell number (N/N_0_), while population doublings are reported in Supplementary Materials as log2fold change (Table S29). In CHO-K1 cells (Figure 5A), PH-pepsin produced a higher fold change, reaching a maximum effect at 10 μM with the population doubling 6.10 ± 0.12 times. However, when comparing population doublings, no significant differences were found between the pepsin and chymotrypsin hydrolysates, or compared to the control, indicating that neither hydrolysate negatively affected cell proliferation.

In QM7 and MACK1 cell lines, the PH-proteinase K hydrolysate resulted in significantly lower proliferation compared to the other hydrolysates tested (Figure 5B). In QM7 cells, the highest hydrolysate concentration reduced the proliferation rate approximately two-fold compared with the control. Similarly, MACK1 cells showed a marked decrease in proliferation, indicating substantial loss of viable cells. AP-chymotrypsin (QM7) and AP-pepsin (MACK1) both showed a dose-dependent effect on cell proliferation. However, AP-pepsin demonstrated a stronger positive effect on MACK1 cell proliferation than the chymotrypsin hydrolysate on QM7 cells.

Fold changes in cell number also differed between cell lines, which was expected given their different origins. CHO-K1 cells showed the highest multiplication factor, likely due to being the least serum-dependent among the three cell-lines. MACK1 proliferated more slowly than CHO-K1 and QM7 cells (Figure 5C). The slower proliferation is consistent with the limited applicability of the MTT assay and the requirement for overnight incubation with the neutral red and resazurin for assessing their viability. The morphology of cells treated with the best-performing hydrolysates for each cell line was also monitored, and no morphological changes were observed compared with the control (Figure S1). Additionally, spent culture media was collected for pH measurement, no extreme pH changes were found, indicating that hydrolysate treatment did not significantly affect overall acidification of the culture medium (Table S30).

### 3.5. Purification and Identification of Chromopeptides Obtained by Enzymatic Digestion of PBPs

Partially purified hydrolysates with the potential to promote cell viability in reduced-serum conditions (see above) were further purified by reverse-phase HPLC to fractionate chromopeptides (Figures S2–S6). Purified chromopeptides, obtained in different fractions, were sequenced by tandem mass spectrometry using their calculated molecular masses (Tables S31–S35, Figures S7–S42), known sequences of PBPs from *A. platensis* and *P. haitanensis* (Tables S31–S35), and the position of chromophore binding [33,34]. The analysis of MS2 spectra of parent ions confirmed known sequences. CID cleaves the thioether bond between the PEB and PCB chromophores and the peptide; thus, ions representing PEB (*m*/*z* ratio 587.29) and peptides without chromophore occur in MS2 spectra of chromopeptides (Tables S36–S114). MS2 spectra also contain a dominant peak with an *m*/*z* ratio of 466.23, arising from the loss of the terminal pyrrole ring by CID fragmentation of PEB and PCB chromophores, respectively, as previously described for the CID fragmentation of tetrapyrroles [33,34,38]. The peak with an *m*/*z* ratio at 299.14 arises from half of the PEB/PCB chromophores and is less abundant.

**Figure 6:**
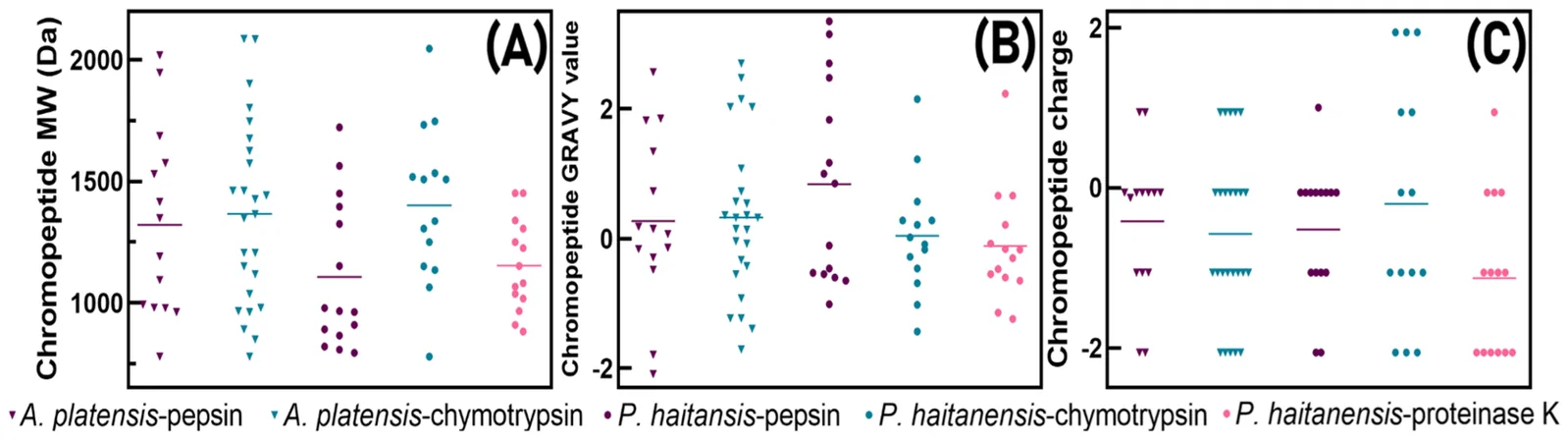
(**A**) Distribution of size, (**B**) GRAVY index (Grand Average of Hydropathicity) and (**C**) charge (at neutral pH) of chromopeptides across five preselected hydrolysates.

We identified 65 different chromopeptides in total, obtained by pepsin and chymotrypsin hydrolysation of *A. platensis* PBPs, as well as pepsin, chymotrypsin, and proteinase K hydrolysation of *P. haitanensis* PBSs (Table 1). Identified chromopeptides vary in size from 2 to 16 amino acid residues, confirming the high propensity of PBPs towards protease digestion. In general, AP-pepsin and AP-chymotrypsin and PH-chymotrypsin contain chromopeptides of larger size compared to PH-pepsin and PH-chymotrypsin (Figure 6A). Most hydrolysates exhibit a similar Grand Average of Hydropathicity (GRAVY) index, while PH-pepsin contains chromopeptides with higher values (Figure 6B). The distributions of charge in different hydrolysates are similar, with the exception of PH-proteinase K, which has a higher number of negatively charged peptides (Figure 6C).

## 4. Discussion

This study evaluated the effects of algal and cyanobacterial protein hydrolysates on cells in reduced-serum conditions by examining cell viability, proliferation, and morphology. The hydrolysates were prepared using cyanobacterium *A. platensis* and red macroalga *P. haitanensis* extracts and pepsin, trypsin, chymotrypsin, subtilisin, thermolysin, and proteinase K. Three animal cell lines were selected for this study: mammalian—CHO-K1, avian—QM7, and fish—MACK1. CHO-K1 cells are epithelial-like in morphology, whereas QM7 and MACK1 cells have a fibroblast-like morphology. CHO-K1 cells are widely used as a robust mammalian model in industrial biotechnology, toxicology and food-related research applications. This cell line was included in this type of study as an easily maintained model for initial screening of hydrolysates’ serum-reducing effects before extending to more relevant cell lines. Moreover, this cell line is widely used as an industrial expression system, and the obtained results are relevant to biotechnological applications where minimizing serum concentration addresses concerns such as costs, variability, contamination risk, etc. QM7 cells originate from quail skeletal muscle and serve as an avian myoblast model for studies of muscle growth and differentiation. MACK1 cells are derived from Atlantic mackerel myoblasts and represent a fish muscle cell model relevant for aquatic cell culture applications and cultivated seafood research. Moreover, MACK1 is a relatively recently established cell line and remains less extensively utilized compared to more established models, which leaves considerable space for further investigation and optimization in cell culture applications and cellular agriculture. These cell lines are relevant in vitro model systems for low-serum or serum-free media development because they represent key vertebrate production systems used in alternative protein technologies. Together, they provide complementary model systems for evaluating media formulations that support robust and scalable cell proliferation in low-serum cell culture applications. At the same time, these cell lines do not fully represent the myoblasts of major livestock species relevant to commercial cultivated meat production, such as bovine satellite cells, porcine myoblasts and poultry muscle stem cells. Future studies incorporating these livestock-derived cell types will be necessary to validate applicability of these hydrolysates specifically for cultivated meat applications.

Animal product-free hydrolysates have been identified as promising supplements for serum-free CHO cell culture media, as they can enhance cell growth and recombinant protein productivity without negatively affecting product quality [39]. Hydrolysates contain bioactive peptides and growth factors that may enhance cell proliferation, improve protein quality, or both. In addition, they exhibit anti-apoptotic, antioxidant, antibacterial, antidiabetic, and immunomodulatory activities [40]. CHO cells have been widely used in studies concerning cell for cultivation in serum-free chemically defined media, because of their use as an expression system for recombinant protein and antibody production. Several studies have demonstrated that supplementation with non-animal-derived protein hydrolysates, including yeast, soy, wheat gluten, pea, rice and rapeseed hydrolysates improve CHO cell growth, viability, recombinant protein productivity and antibody production under serum-free conditions [41,42,43]. Algal extracts have also been studied as serum replacements or functional supplements for use in CHO cell cultivation: a *Chlorella vulgaris*-derived growth factor enhanced CHO cell proliferation and recombinant protein expression in serum-reduced conditions [44], and a heat-treated *Galdieria sulphuraria* extract performed comparably or better than standard FBS supplementation in boosting CHO cell growth [45].

In our study, we used PH-pepsin and PH-chymotrypsin, as they led to a dose-dependent increase in CHO cells’ viability, as determined by the resazurin assay. No significant differences in the cell viability-boosting properties were found between the PH-pepsin and PH-chymotrypsin. Furthermore, neither hydrolysate caused a loss in cell viability, even at the maximum tested concentration. On the other hand, the proliferation assay revealed only a partial dose-dependent increase in the number of CHO cells in the presence of PH-pepsin (up to 10 µM), while the presence of PH-chymotrypsin did not induce any boost in CHO cells’ growth. This discrepancy between the viability test and proliferation assays could be explained by differences in the incubation time between these two assays (3 days vs. 7 days for viability and proliferation assays, respectively). The selection of these two *P. haitanensis* hydrolysates was further justified by their antioxidant capacities, as *P. haitanensis* hydrolysates showed significantly higher ABTS radical scavenging activity than *A. platensis* hydrolysates. A previous study demonstrated that the presence of positively charged side chains in the peptide sequence stimulates the growth of CHO cells, while non-positively charged peptides could also stimulate CHO cell growth, but less so [46]. In this context, our study demonstrated the presence of one positively charged peptide in PH-pepsin, and five in PH-chymotrypsin. Moreover, one patent described the positive effect of short peptides containing C-terminal arginine and obtained from an animal-derived peptone on the growth of eukaryote and prokaryote cells [US 2009/0143248 A1; 2009], while our study identified three peptides with arginine residues at the C-terminus in PH-chymotrypsin. It is also well known that smaller peptides may be more bioactive because of the higher probability of their entry into cells when compared to larger peptides [47], while the presence of hydrophobic amino acid residues may facilitate interactions with cell membranes [48]. In light of this, PH-pepsin contains eight chromopeptides with positive GRAVY values, which may represent one of the structural features potentially contributing to the observed growth-promoting activity.

Protein hydrolysates are gaining attention as promising components in replacing serum bioactive components for cultivated meat production. While several studies have demonstrated the potential of protein hydrolysates to replace FBS and support cell growth in CHO cell line [39,42,43], there is little data concerning their effects on the viability and growth of QM7 cells in serum-reduced or serum-free conditions. Previous studies have revealed that plant-based hydrolysates enhance muscle cell proliferation and differentiation in serum-free conditions by activating multiple signaling pathways, such as PI3K/Akt/mTOR and ERK/AMPK/PGC-1α [49]. In our study, among twelve different hydrolysates, AP-chymotrypsin demonstrated the highest potential to promote the viability of QM7 cells in serum-reduced conditions. This beneficial effect was confirmed by the NRU assay, with the highest improvement in cell viability observed at 10 μM. Moreover, this positive trend was observed not only in a low-serum media supplemented with 10% TPB, but also in low-serum media containing 2% TPB. Re-evaluation of the effects of selected hydrolysates in both low-serum and low-TPB conditions raised important questions regarding the optimization of sustainable media formulations for QM7 cell cultivation; future studies should investigate both serum and additional supplement alternatives. In a previous study, supplementation with 2% chicken serum improved QM7 cell growth and proliferation, demonstrating its potential as an alternative to 10% TPB in standard QM7 culture conditions [50]. The AP-chymotrypsin hydrolysate contains eight chromopeptides with lysin and/or arginine residues, and a recent study demonstrated peptides rich in Lys and Arg, enhance cell adhesion, proliferation, and differentiation [51]. These observations are in good agreement with our study and raise the hypothesis that chromopeptides containing positively charged amino acid residues may contribute to the observed effects on QM7 cell viability.

To the best of our knowledge, this is the first study which systematically explores the effects of chromopeptides derived from algal and cyanobacterial PBPs on the viability and growth of myoblasts in serum-reduced conditions. So far, almost all studies dealing with algal and cyanobacterial PBPs have been based on the use of intact extracts or whole protein fractions. Besides serum replacement applications, algae have also been explored as biological filters: for example, it was shown that treating used QM7 cell culture media with *Chlorella sorokiniana* resulted in a non-cytotoxic medium that enhanced QM7 metabolic activity [52]. Further, it was shown that an aqueous extract of *Anabaena* sp. PCC 7120 successfully supported muscle cell proliferation in serum-reduced conditions [23], while C-phycocyanin incorporated into chitosan/cellulose nanofilms promoted cell proliferation and represented a functional platform for cultivated meat production [53]. Similarly, Sibinčić et al. [20] showed that extracts of *Dunaliella tertiolecta* and *A. platensis*, among a total of 26 examined extracts of algae and cyanobacteria, showed the highest compatibility with QM7 cells during short-term cultivation in serum-reduced conditions [20]. Future research should clarify the molecular mechanisms of their activity, including the potential activation of signaling pathways, as well as the contribution of individual chromopeptides rich in positively charged amino acid residues in the regulation of adhesion, proliferation, and differentiation of QM7 cells and other types of myoblasts. Additionally, particular attention should be paid to the potential role of chromophores in chromopeptides’ ability to support myoblast growth and viability.

To date, only a few studies have explored potential serum replacement strategies for the MACK1 cell cultivation purposes. Compared to finding serum alternatives for CHO-K1 and QM7 cell cultivation, finding suitable serum replacements for MACK1 cell cultivation is more challenging due to higher basal culture conditions, including two-fold higher FBS supplementation as well as the addition of FGF-2 to growth media. Among the tested cell lines, the comparison between QM7 and MACK1 cells was particularly important due to their myogenic morphology. The responses of these two cell lines were comparable, as demonstrated by their similar sensitivity to the proteinase K hydrolysate in both viability and proliferative assays. For both cell lines, the most promising hydrolysates were prepared using PBPs of the same species, *A. platensis*. This finding is consistent with our previous study, in which an *A. platensis* extract also showed the most favorable effect on QM7 and ZEM2S (zebrafish fibroblast) long-term proliferation [20]. In contrast to the CHO-K1 cell line, where biological effects appeared to be associated with the antioxidant capacity of the hydrolysates, no such association was observed in QM7 and MACK1 cells. Instead, *A. platensis* hydrolysates exhibited the strongest proliferative effects despite their lower antioxidant activity compared with *P. haitanensis* hydrolysates. These findings suggest that the effects of the algal/cyanobacterial hydrolysates on cell proliferation may involve more complex underlying mechanisms. In the first study investigating serum replacements for MACK1 cell cultivation, cell growth was evaluated after a 3-day period of cell cultivation in serum-reduced cell growth media (2.5% FBS, 0.25 µg/cm^2^ laminin-511) supplemented with various protein isolates—lupin, pea, rapeseed, chlorella (species not provided), red algae (*P. yezoensis*), spirulina (*A. platensis*) and yeast protein isolates. Rapeseed protein isolate (RPI) significantly improved MACK1 cell growth at 200 and 400 µg/mL, while chlorella (100 µg/mL) and spirulina (50 µg/mL) also enhanced proliferation. Bovine serum albumin- and recombinant human albumin-containing media were used as controls and functionally only BSA (0.4–1 mg/mL) promoted MACK1 cell growth in serum-free conditions [54].

In our 7-day proliferation experiment, the AP-pepsin hydrolysate stimulated MACK1 cell proliferation at concentrations of up to 5 μM (which is approximately equal to 50 µg/mL of total peptide concentration, based on the molecular weight of chromophores, PBP subunits, and the number of attached chromophore per phycobiliprotein subunit) in 2% FBS-supplemented cell media without laminin, suggesting a potentially stronger or more robust response in reduced-serum conditions and in the absence of cell adhesion-supporting compounds such as laminin during a longer treatment period. In another study, the effect of microbial lysates on MACK1 cell growth in the presence of 2.5% FBS. *V. natriegens* ATCC14048, *L. lactis* MG1363, *L. plantarum* WCFS1 and *B. subtilis* 168 lysates inhibited MACK1 growth at ≥10 µg/mL, while *S. cerevisiae* CEN.PK (up to 100 µg/mL), *E. coli* BL21(DE3) (10 µg/mL) and *E. coli* Nissle 1917 (10–50 µg/mL) lysates improved proliferation [55]. Previous studies tested low-molecular-weight protein fractions prepared by size-fractionation of isolates rather than enzymatic hydrolysis, which may explain differences in peptide composition and bioactivity profiles and, consequently, in their biological effects on cells.

Moreover, the bioactivity of AP-pepsin in MACK1 cells may potentially be associated with specific structural characteristics of the identified peptides. Five short peptides (2–4 amino acid residues) were identified, all of which are composed of at least 50% hydrophobic residues and have positive GRAVY values. Based on these sequence characteristics, their hydrophobic nature could potentially favor interactions with the lipid bilayer, while their small size may facilitate cellular uptake; however, further studies are needed to prove the membrane interaction and cellular permeation of chromopeptides. In addition, this hydrolysate contains longer hydrophilic peptides that would be expected to have different membrane-interaction properties based on their physicochemical characteristics, although their cellular behavior remains to be experimentally determined. However, two of these peptides are rich both in aspartic acid and lysine (AA**D**QRG**KDK**CAR**D**, A**D**QRG**KDK**CAR**D**), suggesting a potential role in metal ion chelation and consequently regulation of the extracellular redox environment [56,57].

Lim et al. [54] also developed an L-15/F12 medium (2.5% FBS) supplemented with RPI (0.1–0.2 mg/mL), including insulin, linoleic acid, L-ascorbate-2-phosphate, sodium selenite, FGF-2 (20-fold higher relative to typical culture conditions), TGF-β and transferrin. For the purposes of short-term effect analysis (3 days), MACK1 cells were seeded in complete media (20% FBS) for at least 4 h, whereas for the purposes of investigating long-term effects, the cells were adapted to 10% FBS media for a minimum of three passages before being cultured in 2.5% FBS supplementation. This medium formulation supported MACK1 culturing over 18 passages with maintained proliferation and normal morphology [54]. This aligns with our findings regarding seeding conditions, where direct seeding of MACK1 cells in serum-reduced conditions was not feasible even in the presence of laminin, highlighting the necessity of prior adaptation to cultivation in at least 50% reduced-serum media and/or stepwise serum reduction to achieve long-term successful culture in low-serum conditions. The adhesion capacity of MACK1 cells has also been addressed in another study, where *Saccharina latissima* and *Palmaria palmata* were explored as scaffold materials for cultivated seafood. MACK1 cells showed poor attachment to the surface coatings (laminin 511-E8, poly-L-lysine), even after 2 h or overnight incubation in complete medium, as the authors suggest, likely due to MACK1 cells’ poor robustness rather than scaffold-related limitations [58]. Current research suggests that the long-term culturing of MACK1 cells in serum-reduced conditions requires a highly complex formulation with multiple growth factors and additional supplements, which challenges the concept of a true serum replacement. Although such approaches may address the ethical concerns associated with FBS use, they may not provide a cost-effective alternative.

## 5. Conclusions

In this study, we applied a comprehensive screening approach to algal and cyanobacterial protein hydrolysates to identify the most promising candidates as potential serum-reducing supplements in cultivated meat applications. This work builds upon a previously established workflow for evaluating serum substitutes, which enabled the systematic expansion of testing across multiple additional animal cell lines. *P. haitanensis* hydrolysates exhibited the highest cell growth-promoting activity in CHO-K1 cells, which is consistent with the higher antioxidant capacity of these hydrolysates. In contrast, *A. platensis* hydrolysates showed stimulatory effects on QM7 and MACK1 cell growth. The comparable response in these two cell lines may be associated with their common myogenic characteristics, although more studies are needed to confirm this relationship. In addition, analysis of the peptide composition of these hydrolysates and amino acid sequences of the peptides provided preliminary insight into potential bioactive chromopeptides associated with the promotion or maintenance of cell growth and proliferation. While the antioxidant activity of the hydrolysates suggests their potential bioactive effects, direct validation through pathway-specific analyses (e.g., ERK/MAPK, PI3K/AKT, mTOR signaling), oxidative stress and apoptosis/proliferation markers or omics-based analysis of the identified peptides was not included in the present study, as these analyses require more complex experimental study design and were beyond the scope of this screening study. In this context, future studies investigating purified or synthetic chromopeptides together with pathway-specific analyses could provide deeper insight into the mechanism of action at the cellular level. Together with the screening strategy, this biochemical characterization is intended to support the identification of relationships between hydrolysate composition and their ability to sustain normal cell growth. Overall, these findings may contribute to the development of improved culture media formulations for sustainable cell-based meat and seafood production.

## Figures and Tables

**Table 1:** Sequences of chromopeptides determined by mass spectrometry.

Sample	Sequence	Mass	PBP	Sample	Sequence	Mass	PBP
***P. haitanensis*** **Pepsin**	TGTPQGDCS	1450.61	R-PC	***A. platensis*** **Pepsin**	AADQRGKDKCARD	2019.95	C-PC
	CS	794.33	R-PC		ADQRGKDKCARD	1947.93	C-PC
	ATADGDCS or TADGDCSA	1324.53	R-PE		ITPGDCSA or TPGDCSAL	1348.60	C-PC
	ADGDCS	1152.44	R-PE		TCLRD	1190.54	APC
	GDCS	966.38	R-PC/ R-PE		PAGITPGDCSA	1575.73	C-PC
	CSA	865.37	R-PC/R-PE		PAGITPGDC	1415.64	C-PC
	DCS	909.36	R-PC/ R-PE		DPAGITPGDCSA	1686.73	C-PC
	RMAACLRDGE (Met-ox)	1722.79	R-PE		DPAGITPGDC	1530.67	C-PC
	LTGTPQGDCS	1563.69	R-PC		TATC	980.43	APC
	ATADGDCSA	1395.57	R-PE		CSAL	978.45	C-PC
	CV	806.37	R-PE		AACL or AACI	962.46	APC/ C-PC
	CSAL	978.45	R-PC/ R-PE		ATCL	992.47	APC
	AACL	962.46	R-PC/ R-PE		CA or/and AC	778.34	APC/ C-PC
	ACL	891.42	R-PC/ R-PE		TATCL	1093.52	APC
	CL	820.38	R-PC/ R-PE	***A. platensis*** **Chymotrypsin**	AADQRGKDKCA	1747.84	C-PC
***P. haitanensis*** **Chymotrypsin**	CVPRDM	1305.59	R-PE		ADQRGKDKCA	1676.80	C-PC
	AKCAR	1134.58	R-PC		AACLR	1118.56	C-PC
	KCAR	1063.54	R-PC		GDCS	966.38	C-PC
	NRRMAACL	1518.73	R-PE		GKDKCA	1206.57	C-PC
	YTSRRMAACLRDME	1732.79	R-PC		GDCSA	1037.42	C-PC
	RMAACL or MAACLR	1249.60	R-PC/ R-PE		AACLRDM	1364.63	C-PC
	GTPQGDCSAL	1533.68	R-PE		TPGDCSAL	1348.60	C-PC
	GDCSAL	1150.50	R-PE		NDPAGITPGDCSA	1802.78	C-PC
	TADGDCSALA	1508.65	R-PE		GEEMTATC (Met-ox)	1442.57	APC
	DGDCSALA	1336.56	R-PE		AACIRDLD	1461.70	APC
	LTGTPQGDCSAL	1747.81	R-PC		MTATCLRDLDY (Met-ox)	1902.85	APC
	TADGDCSALA	1508.65	R-PE		PAGITPGDCSA	1573.71	C-PC
	PGEAGDSQEKVNKC	2046.94	R-PE		DKCARDIGY	1625.75	C-PC
	AC	778.34	R-PC/ R-PE		AAC	849.37	C-PC
***P. haitanensis*** **Proteinase K**	GDCSALVAE	1451.66	R-PC		TATC	980.43	APC
	ADGDCSAL	1338.58	R-PE		AIVNDPAGITPGDCSA	2085.97	C-PC
	ADGDCSA	1225.50	R-PE		GEEMTATC	1426.58	APC
	LCVP	1017.51	R-PE		GDCSAL	1150.50	C-PC
	SNASC	1066.44	R-PE		AACL	962.46	C-PC
	GDC	881.36	R-PE/R-PC		AC	778.34	C-PC
	GDCS	966.38	R-PE/R-PC		ACL	891.42	C-PC
	GDCSA	1037.42	R-PE/R-PC				
	DGDCSA	1152.44	R-PE				
	GDCSALVAE	1451.66	R-PC				
	DCS	909.36	R-PE/R-PC				
	RDRLC	1249.65	R-PE				
	DGDCS	1081.41	R-PE				
	CVPRDM	1305.59	R-PE				

## Data Availability

The original contributions presented in the study are included in the article/Supplementary Materials, further inquiries can be directed to the corresponding author.

## Supplementary Materials

The following supporting information can be downloaded at https://www.mdpi.com/article/10.3390/foods15162941/s1. Tables S1–S28. Individual data points and statistical analyses for cell viability and growth assays. Table S29. Population doubling (log2fold change; day 7 vs. day 0) of CHO-K1, QM7 and MACK1 cells treated with selected algal hydrolysates. Figure S1. Morphology of CHO-K1, QM7 and MACK1 cells treated with 5 μM of the selected algal hydrolysate. Table S30. pH measurements of the cell media following the trypan blue exclusion counting test. Figures S2–S6. RP-HPLC chromatograms of the separation (C18 column) of chromopeptides from PBPs digests. Figures S7–S42. MS spectra of chromopeptides purified by RP-HPLC. Tables S31–S35. Chromopeptide sequences identified by tandem mass spectrometry after proteases digestion of PBPs from *Porphyra haitanensis* and *Arthrospira platensis*. Tables S36–S114. Fragmentation (MS2 spectra) of identified chromopeptides.

## Abbreviations

The following abbreviations are used in this manuscript:

FBS	Fetal Bovine Serum
PBP	Phycobiliprotein

## References

[1] United Nations Population Fund (UNFPA) (2023). State of World Population 2023: 8 Billion Lives, Infinite Possibilities—The Case for Rights and Choices.

[2] Food and Agriculture Organization of the United Nations, International Fund for Agricultural Development, UNICEF, World Food Programme, World Health Organization (2024). The State of Food Security and Nutrition in the World 2024: Financing to End Hunger, Food Insecurity and Malnutrition in All Its Forms.

[3] Oluwole O., Ibidapo O., Arowosola T., Raji F., Zandonadi R.P., Alasqah I., Lho L.H., Han H., Raposo A. (2023). Sustainable transformation agenda for enhanced global food and nutrition security: A narrative review. Front. Nutr..

[4] Gerber P.J., Steinfeld H., Henderson B., Mottet A., Opio C., Dijkman J., Falcucci A., Tempio G. (2013). Tackling Climate Change through Livestock: A Global Assessment of Emissions and Mitigation Opportunities.

[5] Cai J., Wang S., Li Y., Dong S., Liang J., Liu Y., Li S. (2024). Industrialization Progress and Challenges of Cultivated Meat. J. Future Foods.

[6] Food and Agriculture Organization of the United Nations (2011). World Livestock 2011: Livestock in Food Security.

[7] Espinosa-Marrón A., Adams K., Sinno L., Cantu-Aldana A., Tamez M., Marrero A., Bhupathiraju S.N., Mattei J. (2022). Environmental impact of animal-based food production and the feasibility of a shift toward sustainable plant-based diets in the United States. Front. Sustain..

[8] Rubio N.R., Xiang N., Kaplan D.L. (2020). Plant-based and cell-based approaches to meat production. Nat. Commun..

[9] Van Eenennaam A.L., Werth S.J. (2021). Animal board invited review: Animal agriculture and alternative meats—Learning from past science communication failures. Animal.

[10] Ahmad S.S., Younis K., Lee G.E., Baral A., Shaikh S., Lee E.J., Ahmad K., Hur S.J., Choi I. (2025). The current scenario in cultivated meat production and the challenges for industrialization. Food Biosci..

[11] Santos A.C.A., Camarena D.E.M., Roncoli Reigado G., Chambergo F.S., Nunes V.A., Trindade M.A., Stuchi Maria-Engler S. (2023). Tissue engineering challenges for cultivated meat to meet the real demand of a global market. Int. J. Mol. Sci..

[12] Garrison G.L., Biermacher J.T., Brorsen B.W. (2022). How much will large-scale production of cell-cultivated meat cost?. J. Agric. Food Res..

[13] United States Department of Agriculture, Food Safety and Inspection Service, U.S. Department of Health and Human Services, Food and Drug Administration (2020). Formal Agreement Between USDA and FDA Relative to Cooperation and Coordination.

[14] Bryant C., Barnett J. (2018). Consumer acceptance of cultivated meat: A systematic review. Meat Sci..

[15] Zhang J., Liu Y., Chen Y., Yuan L., Liu H., Wang J., Liu Q., Zhang Y. (2020). Adipose-derived stem cells: Current applications and future directions in the regeneration of multiple tissues. Stem Cells Int..

[16] Ahmad A., Banat F., Alsafar H., Hasan S.W. (2022). Algae biotechnology for industrial wastewater treatment, bioenergy production, and high-value bioproducts. Sci. Total Environ..

[17] Post M.J., Levenberg S., Kaplan D.L., Genovese N., Fu J., Bryant C.J., Negowetti N., Verzijden K., Moutsatsou P. (2020). Scientific, sustainability and regulatory challenges of cultivated meat. Nat. Food.

[18] Haraguchi Y., Okamoto Y., Shimizu T. (2022). A circular cell culture system using microalgae and mammalian myoblasts for the production of sustainable cultivated meat. Arch. Microbiol..

[19] Haraguchi Y., Shimizu T. (2021). Microalgal culture in animal cell waste medium for sustainable ‘cultivated food’ production. Arch. Microbiol..

[20] Sibinčić N., Krstić Ristivojević M., Gligorijević N., Veličković L., Ćulafić K., Jovanović Z., Ivanov A., Tubić L., Vialleix C., Michel T. (2024). Screening algal and cyanobacterial extracts to identify potential substitutes for fetal bovine serum in cellular meat cultivation. Foods.

[21] Yamanaka K., Haraguchi Y., Takahashi H., Kawashima I., Shimizu T. (2023). Development of serum-free and grain-derived-nutrient-free medium using microalga-derived nutrients and mammalian cell-secreted growth factors for sustainable cultivated meat production. Sci. Rep..

[22] Okamoto Y., Haraguchi Y., Yoshida A., Takahashi H., Yamanaka K., Sawamura N., Asahi T., Shimizu T. (2022). Proliferation and differentiation of primary bovine myoblasts using *Chlorella vulgaris* extract for sustainable production of cultivated meat. Biotechnol. Prog..

[23] Ghosh J., Haraguchi Y., Asahi T., Nakao Y., Shimizu T. (2023). Muscle cell proliferation using water-soluble extract from nitrogen-fixing cyanobacteria *Anabaena* sp. PCC 7120 for sustainable cultivated meat production. Biochem. Biophys. Res. Commun..

[24] Jeong Y., Choi W.Y., Park A., Lee Y.J., Lee Y., Park G.H., Lee S.J., Lee W.K., Ryu Y.K., Kang D.H. (2021). Marine cyanobacterium *Spirulina maxima* as an alternate to the animal cell culture medium supplement. Sci. Rep..

[25] Nikolic M.R., Minic S., Macvanin M., Stanic-Vucinic D., Cirkovic Velickovic T., Jacob-Lopes E., Queiroz Zepka L. (2020). Analytical protocols in phycobiliproteins analysis. Pigments from Microalgae Handbook.

[26] Fernández-Rojas B., Hernández-Juárez J., Pedraza-Chaverri J. (2014). Nutraceutical properties of phycocyanin. J. Funct. Foods.

[27] Sonani R.R., Singh N.K., Kumar J., Thakar D., Madamwar D. (2014). Concurrent purification and antioxidant activity of phycobiliproteins from *Lyngbya* sp. A09DM: An antioxidant and anti-aging potential of phycoerythrin in *Caenorhabditis elegans*. Process Biochem..

[28] Feng Y., Lu H., Hu J., Zheng B., Zhang Y. (2022). Anti-aging effects of R-phycocyanin from *Porphyra haitanensis* on HUVEC cells and *Drosophila melanogaster*. Mar. Drugs.

[29] Sánchez A., Vázquez A. (2017). Bioactive peptides: A review. Food Qual. Saf..

[30] Obaidi I., Mota L.M., Quigley A., Butler M. (2021). The role of protein hydrolysates in prolonging viability and enhancing antibody production of CHO cells. Appl. Microbiol. Biotechnol..

[31] Hubalek S., Post M.J., Moutsatsou P. (2022). Towards resource-efficient and cost-efficient cultured meat. Curr. Opin. Food Sci..

[32] Day L., Cakebread J.A., Loveday S.M. (2022). Food proteins from animals and plants: Differences in the nutritional and functional properties. Trends Food Sci. Technol..

[33] Minic S.L., Stanic-Vucinic D., Mihailovic J., Krstic M., Nikolic M.R., Velickovic T.C. (2016). Digestion by pepsin releases biologically active chromopeptides from C-phycocyanin, a blue-colored biliprotein of microalga Spirulina. J. Proteom..

[34] Gligorijević N., Radović J., Radibratović M., Đorđević M., Spasić D., Nedić O., Nikolić M., Minić S. (2025). Pancreatin digestion of B-phycoerythrin from Porphyridium purpureum releases chromopeptides with inhibitory activity towards SARS-CoV-2 main protease. Algal Res..

[35] Re R., Pellegrini N., Proteggente A., Pannala A., Yang M., Rice-Evans C. (1999). Antioxidant activity applying an improved ABTS radical cation decolorization assay. Free Radic. Biol. Med..

[36] Kumar P., Nagarajan A., Uchil P.D. (2018). Analysis of cell viability by the alamarBlue assay. Cold Spring Harb. Protoc..

[37] Repetto G., Del Peso A., Zurita J.L. (2008). Neutral red uptake assay for the estimation of cell viability/cytotoxicity. Nat. Protoc..

[38] Quinn K.D., Nguyen N.Q., Wach M.M., Wood T.D. (2012). Tandem mass spectrometry of bilin tetrapyrroles by electrospray ionization and collision-induced dissociation. Rapid Commun. Mass Spectrom..

[39] Du Q., Zhang X., Wang T., Wang X. (2022). Effects and mechanisms of animal-free hydrolysates on recombination protein yields in CHO cells. Appl. Microbiol. Biotechnol..

[40] Ho Y.Y., Lu H.K., Lim Z.F.S., Lim H.W., Ho Y.S., Ng S.K. (2021). Applications and analysis of hydrolysates in animal cell culture. Bioresour. Bioprocess..

[41] Kim S.H., Lee G.M. (2009). Development of serum-free medium supplemented with hydrolysates for the production of therapeutic antibodies in CHO cell cultures using design of experiments. Appl. Microbiol. Biotechnol..

[42] Balabashin D.S., Kaliberda E.N., Smirnov I.V., Mokrushina Y.A., Bobik T.V., Aliev T.K., Dolgikh D.A., Kirpichnikov M.P. (2020). Development of a serum-free media based on the optimal combination of recombinant protein additives and hydrolysates of non-animal origin to produce immunoglobulins. Appl. Biochem. Microbiol..

[43] Farges B., Chenu S., Marc A., Goergen J.L. (2008). Kinetics of IFN-γ producing CHO cells and other industrially relevant cell lines in rapeseed-supplemented batch cultures. Process Biochem..

[44] Ng J.Y., Chua M.L., Zhang C., Hong S., Kumar Y., Gokhale R., Ee P.L.R. (2020). *Chlorella vulgaris* extract as a serum replacement that enhances mammalian cell growth and protein expression. Front. Bioeng. Biotechnol..

[45] Eisenberg H., Hütker S., Berger F., Lang I. (2025). Native proteins from *Galdieria sulphuraria* to replace fetal bovine serum in mammalian cell culture. Appl. Microbiol. Biotechnol..

[46] Mosser M., Kapel R., Aymes A., Bonanno L.M., Olmos E., Chevalot I., Marc I., Marc A. (2012). Chromatographic fractionation of yeast extract: A strategy to identify physicochemical properties of compounds promoting CHO cell culture. Process Biochem..

[47] Karami Z., Akbari-Adergani B. (2019). Bioactive food derived peptides: A review on correlation between structure of bioactive peptides and their functional properties. J. Food Sci. Technol..

[48] Yan Y., Liu Y., Zhang X., Zan L., Fang X. (2025). The promotion of cell proliferation by food-derived bioactive peptides: Sources and mechanisms. Metabolites.

[49] Jiang J., Tan J., Jiang B., Wu D., Yao X., Zhao Z., Zhao Y., Wu W., Fu Y. (2025). Serum-free media for cultivated meat: Insights into protein hydrolysates as proliferation enhancers. Food Res. Int..

[50] Choi S., Lee S.I., Shin S. (2022). Effect of Culture Medium Containing Chicken Serum on Growth and Differentiation of QM7 Quail Muscle Cells. Korean J. Poult. Sci..

[51] Kong Y., Toh N.P., Wu Y., Huang D. (2023). Trypsin-treated chickpea protein hydrolysate enhances the cytoaffinity of microbeads for cultivated meat application. Food Res. Int..

[52] Thyden R., Dominko T., Weathers P., Freitas dos Santos A.C., Perreault L., Reddig D., Kloster J., Gaudette G. (2025). Recycling spent animal cell culture media using the thermally resistant microalga *Chlorella sorokiniana*. Syst. Microbiol. Biomanuf..

[53] Park S., Jung S., Heo J., Koh W.G., Lee S., Hong J. (2021). Chitosan/cellulose-based porous nanofilm delivering c-phycocyanin: A novel platform for the production of cost-effective cultivated meat. ACS Appl. Mater. Interfaces.

[54] Lim T., Chang H., Saad M.K., Joyce C.M., Park B., O’Beirne S.X., Cohen M.A., Kaplan D.L. (2024). Development of serum-reduced medium for mackerel muscle cell line cultivation. ACS Sustain. Chem. Eng..

[55] Dolgin J., Chakravarty D., Sullivan S.F., Cai Y., Lim T., Yamaguchi P., Balkan J.E., Xu L., Olawoyin A.D., Lee K. (2025). Microbial lysates as low-cost serum replacements in cellular agriculture media formulation. Food Res. Int..

[56] Zhang X., Cao D., Sun X., Sun S., Xu N. (2019). Preparation and identification of antioxidant peptides from protein hydrolysate of marine alga *Gracilariopsis lemaneiformis*. J. Appl. Phycol..

[57] Csire G., Canabady-Rochelle L., Averlant-Petit M.-C., Selmeczi K., Stefan L. (2020). Both metal-chelating and free radical-scavenging synthetic pentapeptides as efficient inhibitors of reactive oxygen species generation. Metallomics.

[58] van der Tol F. (2025). Making Sashimi: Developing Seaweed-Based Scaffolds for Cultivated Seafood. Master’s Thesis.

