# Supplementary material for: Algal and Cyanobacterial Hydrolysates as Serum-Reducing Supplements Across Mammalian, Avian and Fish Cells with Identification of Bioactive Chromopeptides
Source: Foods. 2026 Aug 21;15(16):2941. doi: 10.3390/foods15162941 (PMC13512191; doi:10.3390/foods15162941)
Supplement: Supplementary file 1 [file foods-15-02941-s001.zip › foods-4463141-supplementary.pdf]

SUPPLEMENTARY MATERIAL FOR

Algal and cyanobacterial hydrolysates as serum-reducing sup-plements across mammalian, avian and fish cells with identi-fication of bioactive chromopeptides

Nikolina Sibinčić Georgieva[1], Ljubodrag Aleksić[2], Nađa Grozdanić Stanisavljević[3], Lora Tubić[2], Boško Tanasković[2], Nikola Gligorijević[4], Marija Stojadinović[2], Simeon Minić[2]*

Table S1. The viability data for A. platensis hydrolysates tested on CHO-K1 cell line by resazurin assay

Sample (μM)	Control			AP - pepsin			AP - trypsin			AP - chymotrypsin			AP - subtilisin			AP - thermolysin			AP - proteinase K		
**0**	96.64	106.59	96.81	x																	
**1**	x			101.50	96.52	105.99	114.12	112.62	107.61	99.96	98.97	101.83	108.48	105.25	116.35	110.35	111.24	112.59	111.40	113.87	113.29
**5**				91.54	84.23	93.11	107.28	105.57	96.54	88.36	86.00	76.65	104.02	113.02	113.68	91.26	93.13	81.98	97.00	90.19	85.52
**10**				87.47	83.13	90.75	84.19	87.16	85.41	81.66	75.94	71.04	100.91	109.24	95.27	90.73	84.33	89.65	86.01	88.39	81.63
**20**				77.23	79.40	80.48	72.09	76.18	72.75	69.98	68.71	67.69	79.27	87.05	82.28	78.29	71.78	72.56	62.33	67.01	67.33

Table S2A. One-way ANOVA statistical analysis for *A. platensis* hydrolysates tested on CHO-K1 cell line by resazurin assay

**ANOVA summary**	
**F**	33.36
**P value**	<0.0001
**P value summary**	****
**Significant diff. among means (P < 0.05)?**	Yes
**R square**	0.9412

**ANOVA table**	**SS**	**DF**	**MS**	**F (DFn, DFd)**	**P value**
**Treatment (between columns)**	14697	24	612.4	F (24, 50) = 33.36	P<0.0001
**Residual (within columns)**	917.9	50	18.36		
**Total**	15615	74			

Table S2B. Dunnett's multiple comparisons test (One-way ANOVA) for *A. platensis* hydrolysates tested on CHO-K1 cell line by resazurin assay

**Dunnett's multiple comparisons test**	**Mean Diff.**	**95.00% CI of diff.**	**Significant?**	**Summary**	**Adjusted P Value**
Control vs. Pepsin 1 µM	-1.336	-12.10 to 9.425	No	ns	0.9995
Control vs. Trypsin 1 µM	-11.45	-22.21 to -0.6875	Yes	*	0.0303
Control vs. Chymotrpysin 1 µM	-0.2541	-11.02 to 10.51	No	ns	>0.9999
Control vs. Pepsin 5 µM	10.37	-0.3906 to 21.13	No	ns	0.0656
Control vs. Trypsin 5 µM	-3.132	-13.89 to 7.629	No	ns	0.9945
Control vs. Chymotrpysin 5 µM	16.33	5.567 to 27.09	Yes	***	0.0005
Control vs. Pepsin 10 µM	12.88	2.124 to 23.65	Yes	**	0.0099
Control vs. Trypsin 10 µM	14.41	3.653 to 25.18	Yes	**	0.0027
Control vs. Chymotrypsin 10 µM	23.79	13.03 to 34.55	Yes	****	<0.0001
Control vs. Pepsin 20 µM	20.96	10.20 to 31.72	Yes	****	<0.0001

Control vs. Trypsin 20 µM	26.33	15.57 to 37.09	Yes	****	<0.0001
Control vs. Chymotrypsin 20 µM	31.20	20.44 to 41.96	Yes	****	<0.0001
Control vs. Subtilisin 1 µM	-10.01	-20.77 to 0.7562	No	ns	0.0839
Control vs. Thermoylsin 1 µM	-11.42	-22.18 to -0.6568	Yes	*	0.0310
Control vs. Proteinase K 1 µM	-12.87	-23.64 to -2.113	Yes	**	0.0099
Control vs. Subtilisin 5 µM	-10.16	-20.92 to 0.5981	No	ns	0.0755
Control vs. Thermolysin 5 µM	11.29	0.5286 to 22.05	Yes	*	0.0341
Control vs. Proteinase K 5 µM	9.186	-1.575 to 19.95	No	ns	0.1412
Control vs. Subtilisin 10 µM	-1.698	-12.46 to 9.063	No	ns	0.9993
Control vs. Thermolysin 10 µM	11.92	1.163 to 22.69	Yes	*	0.0212
Control vs. Proteinase K 10 µM	14.76	4.002 to 25.52	Yes	**	0.0020
Control vs. Subtilisin 20 µM	17.25	6.490 to 28.01	Yes	***	0.0002
Control vs. Thermolysin 20 µM	26.14	15.38 to 36.90	Yes	****	<0.0001
Control vs. Proteinase K 20 µM	34.68	23.92 to 45.44	Yes	****	<0.0001

Table S2C. Two-way ANOVA statistical analysis for *A. platensis* hydrolysates tested on CHO-K1 cell line by resazurin assay

**Two-way ANOVA**	Ordinary				
**Alpha**	0.05				
**Source of Variation**	**% of total variation**	**P value**	**P value summary**	**Significant?**	
**Interaction**	8.328	<0.0001	****	Yes	
**Row (concentration) Factor**	70.63	<0.0001	****	Yes	
**Column (enzyme) Factor**	15.45	<0.0001	****	Yes	
**ANOVA table**	**SS**	**DF**	**MS**	**F (DFn, DFd)**	**P value**

**Interaction**	1269	15	84.61	F (15, 48) = 4.759 P<0.0001
**Row Factor**	10762	3	3587	F (3, 48) = 201.8 P<0.0001
**Column Factor**	2354	5	470.7	F (5, 48) = 26.48 P<0.0001
**Residual**	853.3	48	17.78	

Tabe S2D. Tukey's multiple comparisons test for *A. platensis* hydrolysates tested on CHO-K1 cell line by resazurin assay

**Tukey's multiple comparisons test**	**Mean Diff.**	**95.00% CI of diff.**	**Significant?**	**Summary**	**Adjusted P Value**
1 µM:Pepsin vs. 1 µM:Trypsin	-10.11	-23.38 to 3.157	No	ns	0.3721
1 µM:Pepsin vs. 1 µM:Chymotrypsin	1.082	-12.19 to 14.35	No	ns	>0.9999
1 µM:Pepsin vs. 1 µM:Subtilisin	-8.689	-21.96 to 4.581	No	ns	0.6541
1 µM:Pepsin vs. 1 µM:Thermolysin	-10.06	-23.33 to 3.212	No	ns	0.3821
1 µM:Pepsin vs. 1 µM:Proteinase K	-11.52	-24.79 to 1.752	No	ns	0.1701
1 µM:Pepsin vs. 5 µM:Pepsin	11.71	-1.563 to 24.98	No	ns	0.1508
1 µM:Pepsin vs. 5 µM:Trypsin	-1.796	-15.07 to 11.47	No	ns	>0.9999
1 µM:Pepsin vs. 5 µM:Chymotrypsin	17.66	4.395 to 30.93	Yes	**	0.0011
1 µM:Pepsin vs. 5 µM:Subtilisin	-8.905	-22.17 to 4.364	No	ns	0.6103
1 µM:Pepsin vs. 5 µM:Thermolysin	12.55	-0.7216 to 25.82	No	ns	0.0852
1 µM:Pepsin vs. 5 µM:Proteinase K	10.43	-2.839 to 23.70	No	ns	0.3174
1 µM:Pepsin vs. 10 µM:Pepsin	14.22	0.9514 to 27.49	Yes	*	0.0236
1 µM:Pepsin vs. 10 µM:Trypsin	15.75	2.480 to 29.02	Yes	**	0.0064
1 µM:Pepsin vs. 10 µM:Chymotrypsin	25.12	11.85 to 38.39	Yes	****	<0.0001
1 µM:Pepsin vs. 10 µM:Subtilisin	-0.4704	-13.74 to 12.80	No	ns	>0.9999
1 µM:Pepsin vs. 10 µM:Thermolysin	13.10	-0.1687 to 26.37	No	ns	0.0568
1 µM:Pepsin vs. 10 µM:Proteinase K	15.99	2.724 to 29.26	Yes	**	0.0051

1 µM:Pepsin vs. 20 µM:Pepsin	22.30	9.030 to 35.57	Yes	****	<0.0001
1 µM:Pepsin vs. 20 µM:Trypsin	27.66	14.39 to 40.93	Yes	****	<0.0001
1 µM:Pepsin vs. 20 µM:Chymotrypsin	32.54	19.27 to 45.81	Yes	****	<0.0001
1 µM:Pepsin vs. 20 µM:Subtilisin	18.47	5.202 to 31.74	Yes	***	0.0005
1 µM:Pepsin vs. 20 µM:Thermolysin	27.13	13.86 to 40.40	Yes	****	<0.0001
1 µM:Pepsin vs. 20 µM:Proteinase K	35.78	22.51 to 49.05	Yes	****	<0.0001
1 µM:Trypsin vs. 1 µM:Chymotrypsin	11.19	-2.075 to 24.46	No	ns	0.2072
1 µM:Trypsin vs. 1 µM:Subtilisin	1.424	-11.85 to 14.69	No	ns	>0.9999
1 µM:Trypsin vs. 1 µM:Thermolysin	0.05518	-13.21 to 13.32	No	ns	>0.9999
1 µM:Trypsin vs. 1 µM:Proteinase K	-1.404	-14.67 to 11.87	No	ns	>0.9999
1 µM:Trypsin vs. 5 µM:Pepsin	21.82	8.550 to 35.09	Yes	****	<0.0001
1 µM:Trypsin vs. 5 µM:Trypsin	8.316	-4.953 to 21.59	No	ns	0.7267
1 µM:Trypsin vs. 5 µM:Chymotrypsin	27.78	14.51 to 41.05	Yes	****	<0.0001
1 µM:Trypsin vs. 5 µM:Subtilisin	1.208	-12.06 to 14.48	No	ns	>0.9999
1 µM:Trypsin vs. 5 µM:Thermolysin	22.66	9.391 to 35.93	Yes	****	<0.0001
1 µM:Trypsin vs. 5 µM:Proteinase K	20.54	7.274 to 33.81	Yes	****	<0.0001
1 µM:Trypsin vs. 10 µM:Pepsin	24.33	11.06 to 37.60	Yes	****	<0.0001
1 µM:Trypsin vs. 10 µM:Trypsin	25.86	12.59 to 39.13	Yes	****	<0.0001
1 µM:Trypsin vs. 10 µM:Chymotrypsin	35.24	21.97 to 48.51	Yes	****	<0.0001
1 µM:Trypsin vs. 10 µM:Subtilisin	9.642	-3.627 to 22.91	No	ns	0.4609
1 µM:Trypsin vs. 10 µM:Thermolysin	23.21	9.944 to 36.48	Yes	****	<0.0001
1 µM:Trypsin vs. 10 µM:Proteinase K	26.11	12.84 to 39.38	Yes	****	<0.0001
1 µM:Trypsin vs. 20 µM:Pepsin	32.41	19.14 to 45.68	Yes	****	<0.0001
1 µM:Trypsin vs. 20 µM:Trypsin	37.78	24.51 to 51.04	Yes	****	<0.0001

1 µM:Trypsin vs. 20 µM:Chymotrypsin	42.65	29.38 to 55.92	Yes	****	<0.0001
1 µM:Trypsin vs. 20 µM:Subtilisin	28.58	15.32 to 41.85	Yes	****	<0.0001
1 µM:Trypsin vs. 20 µM:Thermolysin	37.24	23.97 to 50.51	Yes	****	<0.0001
1 µM:Trypsin vs. 20 µM:Proteinase K	45.89	32.62 to 59.16	Yes	****	<0.0001
1 µM:Chymotrypsin vs. 1 µM:Subtilisin	-9.771	-23.04 to 3.499	No	ns	0.4358
1 µM:Chymotrypsin vs. 1 µM:Thermolysin	-11.14	-24.41 to 2.130	No	ns	0.2141
1 µM:Chymotrypsin vs. 1 µM:Proteinase K	-12.60	-25.87 to 0.6704	No	ns	0.0821
1 µM:Chymotrypsin vs. 5 µM:Pepsin	10.62	-2.645 to 23.89	No	ns	0.2865
1 µM:Chymotrypsin vs. 5 µM:Trypsin	-2.878	-16.15 to 10.39	No	ns	>0.9999
1 µM:Chymotrypsin vs. 5 µM:Chymotrypsin	16.58	3.313 to 29.85	Yes	**	0.0030
1 µM:Chymotrypsin vs. 5 µM:Subtilisin	-9.987	-23.26 to 3.282	No	ns	0.3950
1 µM:Chymotrypsin vs. 5 µM:Thermolysin	11.47	-1.804 to 24.74	No	ns	0.1756
1 µM:Chymotrypsin vs. 5 µM:Proteinase K	9.349	-3.921 to 22.62	No	ns	0.5196
1 µM:Chymotrypsin vs. 10 µM:Pepsin	13.14	-0.1306 to 26.41	No	ns	0.0552
1 µM:Chymotrypsin vs. 10 µM:Trypsin	14.67	1.398 to 27.94	Yes	*	0.0163
1 µM:Chymotrypsin vs. 10 µM:Chymotrypsin	24.04	10.77 to 37.31	Yes	****	<0.0001
1 µM:Chymotrypsin vs. 10 µM:Subtilisin	-1.552	-14.82 to 11.72	No	ns	>0.9999
1 µM:Chymotrypsin vs. 10 µM:Thermolysin	12.02	-1.251 to 25.29	No	ns	0.1228
1 µM:Chymotrypsin vs. 10 µM:Proteinase K	14.91	1.643 to 28.18	Yes	*	0.0132
1 µM:Chymotrypsin vs. 20 µM:Pepsin	21.22	7.948 to 34.49	Yes	****	<0.0001
1 µM:Chymotrypsin vs. 20 µM:Trypsin	26.58	13.31 to 39.85	Yes	****	<0.0001
1 µM:Chymotrypsin vs. 20 µM:Chymotrypsin	31.46	18.19 to 44.73	Yes	****	<0.0001
1 µM:Chymotrypsin vs. 20 µM:Subtilisin	17.39	4.120 to 30.66	Yes	**	0.0014
1 µM:Chymotrypsin vs. 20 µM:Thermolysin	26.05	12.78 to 39.32	Yes	****	<0.0001

1 µM:Chymotrypsin vs. 20 µM:Proteinase K	34.70	21.43 to 47.96	Yes	****	<0.0001
1 µM:Subtilisin vs. 1 µM:Thermolysin	-1.369	-14.64 to 11.90	No	ns	>0.9999
1 µM:Subtilisin vs. 1 µM:Proteinase K	-2.828	-16.10 to 10.44	No	ns	>0.9999
1 µM:Subtilisin vs. 5 µM:Pepsin	20.40	7.126 to 33.67	Yes	****	<0.0001
1 µM:Subtilisin vs. 5 µM:Trypsin	6.893	-6.377 to 20.16	No	ns	0.9298
1 µM:Subtilisin vs. 5 µM:Chymotrypsin	26.35	13.08 to 39.62	Yes	****	<0.0001
1 µM:Subtilisin vs. 5 µM:Subtilisin	-0.2162	-13.49 to 13.05	No	ns	>0.9999
1 µM:Subtilisin vs. 5 µM:Thermolysin	21.24	7.967 to 34.51	Yes	****	<0.0001
1 µM:Subtilisin vs. 5 µM:Proteinase K	19.12	5.850 to 32.39	Yes	***	0.0003
1 µM:Subtilisin vs. 10 µM:Pepsin	22.91	9.640 to 36.18	Yes	****	<0.0001
1 µM:Subtilisin vs. 10 µM:Trypsin	24.44	11.17 to 37.71	Yes	****	<0.0001
1 µM:Subtilisin vs. 10 µM:Chymotrypsin	33.81	20.54 to 47.08	Yes	****	<0.0001
1 µM:Subtilisin vs. 10 µM:Subtilisin	8.219	-5.051 to 21.49	No	ns	0.7449
1 µM:Subtilisin vs. 10 µM:Thermolysin	21.79	8.520 to 35.06	Yes	****	<0.0001
1 µM:Subtilisin vs. 10 µM:Proteinase K	24.68	11.41 to 37.95	Yes	****	<0.0001
1 µM:Subtilisin vs. 20 µM:Pepsin	30.99	17.72 to 44.26	Yes	****	<0.0001
1 µM:Subtilisin vs. 20 µM:Trypsin	36.35	23.08 to 49.62	Yes	****	<0.0001
1 µM:Subtilisin vs. 20 µM:Chymotrypsin	41.23	27.96 to 54.50	Yes	****	<0.0001
1 µM:Subtilisin vs. 20 µM:Subtilisin	27.16	13.89 to 40.43	Yes	****	<0.0001
1 µM:Subtilisin vs. 20 µM:Thermolysin	35.82	22.55 to 49.09	Yes	****	<0.0001
1 µM:Subtilisin vs. 20 µM:Proteinase K	44.47	31.20 to 57.74	Yes	****	<0.0001
1 µM:Thermolysin vs. 1 µM:Proteinase K	-1.460	-14.73 to 11.81	No	ns	>0.9999
1 µM:Thermolysin vs. 5 µM:Pepsin	21.76	8.495 to 35.03	Yes	****	<0.0001
1 µM:Thermolysin vs. 5 µM:Trypsin	8.261	-5.008 to 21.53	No	ns	0.7370

1 µM:Thermolysin vs. 5 µM:Chymotrypsin	27.72	14.45 to 40.99	Yes	****	<0.0001
1 µM:Thermolysin vs. 5 µM:Subtilisin	1.152	-12.12 to 14.42	No	ns	>0.9999
1 µM:Thermolysin vs. 5 µM:Thermolysin	22.61	9.336 to 35.88	Yes	****	<0.0001
1 µM:Thermolysin vs. 5 µM:Proteinase K	20.49	7.219 to 33.76	Yes	****	<0.0001
1 µM:Thermolysin vs. 10 µM:Pepsin	24.28	11.01 to 37.55	Yes	****	<0.0001
1 µM:Thermolysin vs. 10 µM:Trypsin	25.81	12.54 to 39.08	Yes	****	<0.0001
1 µM:Thermolysin vs. 10 µM:Chymotrypsin	35.18	21.91 to 48.45	Yes	****	<0.0001
1 µM:Thermolysin vs. 10 µM:Subtilisin	9.587	-3.682 to 22.86	No	ns	0.4718
1 µM:Thermolysin vs. 10 µM:Thermolysin	23.16	9.889 to 36.43	Yes	****	<0.0001
1 µM:Thermolysin vs. 10 µM:Proteinase K	26.05	12.78 to 39.32	Yes	****	<0.0001
1 µM:Thermolysin vs. 20 µM:Pepsin	32.36	19.09 to 45.63	Yes	****	<0.0001
1 µM:Thermolysin vs. 20 µM:Trypsin	37.72	24.45 to 50.99	Yes	****	<0.0001
1 µM:Thermolysin vs. 20 µM:Chymotrypsin	42.60	29.33 to 55.87	Yes	****	<0.0001
1 µM:Thermolysin vs. 20 µM:Subtilisin	28.53	15.26 to 41.80	Yes	****	<0.0001
1 µM:Thermolysin vs. 20 µM:Thermolysin	37.19	23.92 to 50.46	Yes	****	<0.0001
1 µM:Thermolysin vs. 20 µM:Proteinase K	45.83	32.57 to 59.10	Yes	****	<0.0001
1 µM:Proteinase K vs. 5 µM:Pepsin	23.22	9.954 to 36.49	Yes	****	<0.0001
1 µM:Proteinase K vs. 5 µM:Trypsin	9.721	-3.549 to 22.99	No	ns	0.4455
1 µM:Proteinase K vs. 5 µM:Chymotrypsin	29.18	15.91 to 42.45	Yes	****	<0.0001
1 µM:Proteinase K vs. 5 µM:Subtilisin	2.612	-10.66 to 15.88	No	ns	>0.9999
1 µM:Proteinase K vs. 5 µM:Thermolysin	24.07	10.80 to 37.33	Yes	****	<0.0001
1 µM:Proteinase K vs. 5 µM:Proteinase K	21.95	8.678 to 35.22	Yes	****	<0.0001
1 µM:Proteinase K vs. 10 µM:Pepsin	25.74	12.47 to 39.01	Yes	****	<0.0001
1 µM:Proteinase K vs. 10 µM:Trypsin	27.27	14.00 to 40.54	Yes	****	<0.0001

1 µM:Proteinase K vs. 10 µM:Chymotrypsin	36.64	23.37 to 49.91	Yes	****	<0.0001
1 µM:Proteinase K vs. 10 µM:Subtilisin	11.05	-2.223 to 24.32	No	ns	0.2261
1 µM:Proteinase K vs. 10 µM:Thermolysin	24.62	11.35 to 37.89	Yes	****	<0.0001
1 µM:Proteinase K vs. 10 µM:Proteinase K	27.51	14.24 to 40.78	Yes	****	<0.0001
1 µM:Proteinase K vs. 20 µM:Pepsin	33.82	20.55 to 47.09	Yes	****	<0.0001
1 µM:Proteinase K vs. 20 µM:Trypsin	39.18	25.91 to 52.45	Yes	****	<0.0001
1 µM:Proteinase K vs. 20 µM:Chymotrypsin	44.06	30.79 to 57.33	Yes	****	<0.0001
1 µM:Proteinase K vs. 20 µM:Subtilisin	29.99	16.72 to 43.26	Yes	****	<0.0001
1 µM:Proteinase K vs. 20 µM:Thermolysin	38.65	25.38 to 51.92	Yes	****	<0.0001
1 µM:Proteinase K vs. 20 µM:Proteinase K	47.29	34.02 to 60.56	Yes	****	<0.0001
5 µM:Pepsin vs. 5 µM:Trypsin	-13.50	-26.77 to -0.2335	Yes	*	0.0418
5 µM:Pepsin vs. 5 µM:Chymotrypsin	5.958	-7.312 to 19.23	No	ns	0.9835
5 µM:Pepsin vs. 5 µM:Subtilisin	-20.61	-33.88 to -7.342	Yes	****	<0.0001
5 µM:Pepsin vs. 5 µM:Thermolysin	0.8413	-12.43 to 14.11	No	ns	>0.9999
5 µM:Pepsin vs. 5 µM:Proteinase K	-1.276	-14.55 to 11.99	No	ns	>0.9999
5 µM:Pepsin vs. 10 µM:Pepsin	2.514	-10.76 to 15.78	No	ns	>0.9999
5 µM:Pepsin vs. 10 µM:Trypsin	4.043	-9.226 to 17.31	No	ns	>0.9999
5 µM:Pepsin vs. 10 µM:Chymotrypsin	13.42	0.1470 to 26.69	Yes	*	0.0447
5 µM:Pepsin vs. 10 µM:Subtilisin	-12.18	-25.45 to 1.092	No	ns	0.1103
5 µM:Pepsin vs. 10 µM:Thermolysin	1.394	-11.88 to 14.66	No	ns	>0.9999
5 µM:Pepsin vs. 10 µM:Proteinase K	4.287	-8.982 to 17.56	No	ns	0.9998
5 µM:Pepsin vs. 20 µM:Pepsin	10.59	-2.677 to 23.86	No	ns	0.2914
5 µM:Pepsin vs. 20 µM:Trypsin	15.96	2.686 to 29.23	Yes	**	0.0053
5 µM:Pepsin vs. 20 µM:Chymotrypsin	20.83	7.564 to 34.10	Yes	****	<0.0001

5 µM:Pepsin vs. 20 µM:Subtilisin	6.765	-6.504 to 20.03	No	ns	0.9405
5 µM:Pepsin vs. 20 µM:Thermolysin	15.42	2.152 to 28.69	Yes	**	0.0085
5 µM:Pepsin vs. 20 µM:Proteinase K	24.07	10.80 to 37.34	Yes	****	<0.0001
5 µM:Trypsin vs. 5 µM:Chymotrypsin	19.46	6.192 to 32.73	Yes	***	0.0002
5 µM:Trypsin vs. 5 µM:Subtilisin	-7.109	-20.38 to 6.161	No	ns	0.9087
5 µM:Trypsin vs. 5 µM:Thermolysin	14.34	1.075 to 27.61	Yes	*	0.0213
5 µM:Trypsin vs. 5 µM:Proteinase K	12.23	-1.042 to 25.50	No	ns	0.1066
5 µM:Trypsin vs. 10 µM:Pepsin	16.02	2.748 to 29.29	Yes	**	0.0050
5 µM:Trypsin vs. 10 µM:Trypsin	17.55	4.277 to 30.82	Yes	**	0.0012
5 µM:Trypsin vs. 10 µM:Chymotrypsin	26.92	13.65 to 40.19	Yes	****	<0.0001
5 µM:Trypsin vs. 10 µM:Subtilisin	1.326	-11.94 to 14.60	No	ns	>0.9999
5 µM:Trypsin vs. 10 µM:Thermolysin	14.90	1.628 to 28.17	Yes	*	0.0134
5 µM:Trypsin vs. 10 µM:Proteinase K	17.79	4.521 to 31.06	Yes	***	0.0010
5 µM:Trypsin vs. 20 µM:Pepsin	24.10	10.83 to 37.37	Yes	****	<0.0001
5 µM:Trypsin vs. 20 µM:Trypsin	29.46	16.19 to 42.73	Yes	****	<0.0001
5 µM:Trypsin vs. 20 µM:Chymotrypsin	34.34	21.07 to 47.61	Yes	****	<0.0001
5 µM:Trypsin vs. 20 µM:Subtilisin	20.27	6.999 to 33.54	Yes	****	<0.0001
5 µM:Trypsin vs. 20 µM:Thermolysin	28.92	15.66 to 42.19	Yes	****	<0.0001
5 µM:Trypsin vs. 20 µM:Proteinase K	37.57	24.30 to 50.84	Yes	****	<0.0001
5 µM:Chymotrypsin vs. 5 µM:Subtilisin	-26.57	-39.84 to -13.30	Yes	****	<0.0001
5 µM:Chymotrypsin vs. 5 µM:Thermolysin	-5.117	-18.39 to 8.153	No	ns	0.9974
5 µM:Chymotrypsin vs. 5 µM:Proteinase K	-7.234	-20.50 to 6.036	No	ns	0.8948
5 µM:Chymotrypsin vs. 10 µM:Pepsin	-3.444	-16.71 to 9.826	No	ns	>0.9999
5 µM:Chymotrypsin vs. 10 µM:Trypsin	-1.915	-15.18 to 11.35	No	ns	>0.9999

5 µM:Chymotrypsin vs. 10 µM:Chymotrypsin	7.459	-5.811 to 20.73	No	ns	0.8667
5 µM:Chymotrypsin vs. 10 µM:Subtilisin	-18.14	-31.40 to -4.866	Yes	***	0.0007
5 µM:Chymotrypsin vs. 10 µM:Thermolysin	-4.564	-17.83 to 8.706	No	ns	0.9995
5 µM:Chymotrypsin vs. 10 µM:Proteinase K	-1.671	-14.94 to 11.60	No	ns	>0.9999
5 µM:Chymotrypsin vs. 20 µM:Pepsin	4.635	-8.635 to 17.90	No	ns	0.9994
5 µM:Chymotrypsin vs. 20 µM:Trypsin	9.998	-3.272 to 23.27	No	ns	0.3930
5 µM:Chymotrypsin vs. 20 µM:Chymotrypsin	14.88	1.606 to 28.14	Yes	*	0.0136
5 µM:Chymotrypsin vs. 20 µM:Subtilisin	0.8072	-12.46 to 14.08	No	ns	>0.9999
5 µM:Chymotrypsin vs. 20 µM:Thermolysin	9.464	-3.806 to 22.73	No	ns	0.4964
5 µM:Chymotrypsin vs. 20 µM:Proteinase K	18.11	4.843 to 31.38	Yes	***	0.0007
5 µM:Subtilisin vs. 5 µM:Thermolysin	21.45	8.184 to 34.72	Yes	****	<0.0001
5 µM:Subtilisin vs. 5 µM:Proteinase K	19.34	6.066 to 32.61	Yes	***	0.0002
5 µM:Subtilisin vs. 10 µM:Pepsin	23.13	9.857 to 36.40	Yes	****	<0.0001
5 µM:Subtilisin vs. 10 µM:Trypsin	24.66	11.39 to 37.92	Yes	****	<0.0001
5 µM:Subtilisin vs. 10 µM:Chymotrypsin	34.03	20.76 to 47.30	Yes	****	<0.0001
5 µM:Subtilisin vs. 10 µM:Subtilisin	8.435	-4.835 to 21.70	No	ns	0.7042
5 µM:Subtilisin vs. 10 µM:Thermolysin	22.01	8.736 to 35.28	Yes	****	<0.0001
5 µM:Subtilisin vs. 10 µM:Proteinase K	24.90	11.63 to 38.17	Yes	****	<0.0001
5 µM:Subtilisin vs. 20 µM:Pepsin	31.20	17.94 to 44.47	Yes	****	<0.0001
5 µM:Subtilisin vs. 20 µM:Trypsin	36.57	23.30 to 49.84	Yes	****	<0.0001
5 µM:Subtilisin vs. 20 µM:Chymotrypsin	41.44	28.18 to 54.71	Yes	****	<0.0001
5 µM:Subtilisin vs. 20 µM:Subtilisin	27.38	14.11 to 40.65	Yes	****	<0.0001
5 µM:Subtilisin vs. 20 µM:Thermolysin	36.03	22.76 to 49.30	Yes	****	<0.0001
5 µM:Subtilisin vs. 20 µM:Proteinase K	44.68	31.41 to 57.95	Yes	****	<0.0001

5 µM:Thermolysin vs. 5 µM:Proteinase K	-2.117	-15.39 to 11.15	No	ns	>0.9999
5 µM:Thermolysin vs. 10 µM:Pepsin	1.673	-11.60 to 14.94	No	ns	>0.9999
5 µM:Thermolysin vs. 10 µM:Trypsin	3.202	-10.07 to 16.47	No	ns	>0.9999
5 µM:Thermolysin vs. 10 µM:Chymotrypsin	12.58	-0.6943 to 25.84	No	ns	0.0835
5 µM:Thermolysin vs. 10 µM:Subtilisin	-13.02	-26.29 to 0.2511	No	ns	0.0604
5 µM:Thermolysin vs. 10 µM:Thermolysin	0.5529	-12.72 to 13.82	No	ns	>0.9999
5 µM:Thermolysin vs. 10 µM:Proteinase K	3.446	-9.824 to 16.72	No	ns	>0.9999
5 µM:Thermolysin vs. 20 µM:Pepsin	9.752	-3.518 to 23.02	No	ns	0.4396
5 µM:Thermolysin vs. 20 µM:Trypsin	15.11	1.845 to 28.38	Yes	*	0.0111
5 µM:Thermolysin vs. 20 µM:Chymotrypsin	19.99	6.722 to 33.26	Yes	***	0.0001
5 µM:Thermolysin vs. 20 µM:Subtilisin	5.924	-7.346 to 19.19	No	ns	0.9845
5 µM:Thermolysin vs. 20 µM:Thermolysin	14.58	1.311 to 27.85	Yes	*	0.0175
5 µM:Thermolysin vs. 20 µM:Proteinase K	23.23	9.959 to 36.50	Yes	****	<0.0001
5 µM:Proteinase K vs. 10 µM:Pepsin	3.790	-9.479 to 17.06	No	ns	>0.9999
5 µM:Proteinase K vs. 10 µM:Trypsin	5.319	-7.950 to 18.59	No	ns	0.9957
5 µM:Proteinase K vs. 10 µM:Chymotrypsin	14.69	1.423 to 27.96	Yes	*	0.0159
5 µM:Proteinase K vs. 10 µM:Subtilisin	-10.90	-24.17 to 2.368	No	ns	0.2458
5 µM:Proteinase K vs. 10 µM:Thermolysin	2.670	-10.60 to 15.94	No	ns	>0.9999
5 µM:Proteinase K vs. 10 µM:Proteinase K	5.563	-7.706 to 18.83	No	ns	0.9925
5 µM:Proteinase K vs. 20 µM:Pepsin	11.87	-1.401 to 25.14	No	ns	0.1357
5 µM:Proteinase K vs. 20 µM:Trypsin	17.23	3.962 to 30.50	Yes	**	0.0016
5 µM:Proteinase K vs. 20 µM:Chymotrypsin	22.11	8.839 to 35.38	Yes	****	<0.0001
5 µM:Proteinase K vs. 20 µM:Subtilisin	8.041	-5.228 to 21.31	No	ns	0.7766
5 µM:Proteinase K vs. 20 µM:Thermolysin	16.70	3.428 to 29.97	Yes	**	0.0027

5 µM:Proteinase K vs. 20 µM:Proteinase K	25.35	12.08 to 38.62	Yes	****	<0.0001
10 µM:Pepsin vs. 10 µM:Trypsin	1.529	-11.74 to 14.80	No	ns	>0.9999
10 µM:Pepsin vs. 10 µM:Chymotrypsin	10.90	-2.367 to 24.17	No	ns	0.2457
10 µM:Pepsin vs. 10 µM:Subtilisin	-14.69	-27.96 to -1.422	Yes	*	0.0159
10 µM:Pepsin vs. 10 µM:Thermolysin	-1.120	-14.39 to 12.15	No	ns	>0.9999
10 µM:Pepsin vs. 10 µM:Proteinase K	1.773	-11.50 to 15.04	No	ns	>0.9999
10 µM:Pepsin vs. 20 µM:Pepsin	8.079	-5.191 to 21.35	No	ns	0.7701
10 µM:Pepsin vs. 20 µM:Trypsin	13.44	0.1721 to 26.71	Yes	*	0.0438
10 µM:Pepsin vs. 20 µM:Chymotrypsin	18.32	5.049 to 31.59	Yes	***	0.0006
10 µM:Pepsin vs. 20 µM:Subtilisin	4.251	-9.019 to 17.52	No	ns	0.9998
10 µM:Pepsin vs. 20 µM:Thermolysin	12.91	-0.3621 to 26.18	No	ns	0.0656
10 µM:Pepsin vs. 20 µM:Proteinase K	21.56	8.287 to 34.83	Yes	****	<0.0001
10 µM:Trypsin vs. 10 µM:Chymotrypsin	9.373	-3.896 to 22.64	No	ns	0.5147
10 µM:Trypsin vs. 10 µM:Subtilisin	-16.22	-29.49 to -2.951	Yes	**	0.0042
10 µM:Trypsin vs. 10 µM:Thermolysin	-2.649	-15.92 to 10.62	No	ns	>0.9999
10 µM:Trypsin vs. 10 µM:Proteinase K	0.2440	-13.03 to 13.51	No	ns	>0.9999
10 µM:Trypsin vs. 20 µM:Pepsin	6.550	-6.720 to 19.82	No	ns	0.9560
10 µM:Trypsin vs. 20 µM:Trypsin	11.91	-1.357 to 25.18	No	ns	0.1318
10 µM:Trypsin vs. 20 µM:Chymotrypsin	16.79	3.520 to 30.06	Yes	**	0.0025
10 µM:Trypsin vs. 20 µM:Subtilisin	2.722	-10.55 to 15.99	No	ns	>0.9999
10 µM:Trypsin vs. 20 µM:Thermolysin	11.38	-1.891 to 24.65	No	ns	0.1854
10 µM:Trypsin vs. 20 µM:Proteinase K	20.03	6.757 to 33.30	Yes	***	0.0001
10 µM:Chymotrypsin vs. 10 µM:Subtilisin	-25.59	-38.86 to -12.32	Yes	****	<0.0001
10 µM:Chymotrypsin vs. 10 µM:Thermolysin	-12.02	-25.29 to 1.247	No	ns	0.1225

10 µM:Chymotrypsin vs. 10 µM:Proteinase K	-9.129	-22.40 to 4.140	No	ns	0.5645
10 µM:Chymotrypsin vs. 20 µM:Pepsin	-2.824	-16.09 to 10.45	No	ns	>0.9999
10 µM:Chymotrypsin vs. 20 µM:Trypsin	2.539	-10.73 to 15.81	No	ns	>0.9999
10 µM:Chymotrypsin vs. 20 µM:Chymotrypsin	7.416	-5.853 to 20.69	No	ns	0.8722
10 µM:Chymotrypsin vs. 20 µM:Subtilisin	-6.651	-19.92 to 6.618	No	ns	0.9491
10 µM:Chymotrypsin vs. 20 µM:Thermolysin	2.005	-11.26 to 15.27	No	ns	>0.9999
10 µM:Chymotrypsin vs. 20 µM:Proteinase K	10.65	-2.616 to 23.92	No	ns	0.2820
10 µM:Subtilisin vs. 10 µM:Thermolysin	13.57	0.3018 to 26.84	Yes	*	0.0396
10 µM:Subtilisin vs. 10 µM:Proteinase K	16.46	3.195 to 29.73	Yes	**	0.0034
10 µM:Subtilisin vs. 20 µM:Pepsin	22.77	9.500 to 36.04	Yes	****	<0.0001
10 µM:Subtilisin vs. 20 µM:Trypsin	28.13	14.86 to 41.40	Yes	****	<0.0001
10 µM:Subtilisin vs. 20 µM:Chymotrypsin	33.01	19.74 to 46.28	Yes	****	<0.0001
10 µM:Subtilisin vs. 20 µM:Subtilisin	18.94	5.673 to 32.21	Yes	***	0.0003
10 µM:Subtilisin vs. 20 µM:Thermolysin	27.60	14.33 to 40.87	Yes	****	<0.0001
10 µM:Subtilisin vs. 20 µM:Proteinase K	36.25	22.98 to 49.52	Yes	****	<0.0001
10 µM:Thermolysin vs. 10 µM:Proteinase K	2.893	-10.38 to 16.16	No	ns	>0.9999
10 µM:Thermolysin vs. 20 µM:Pepsin	9.199	-4.071 to 22.47	No	ns	0.5502
10 µM:Thermolysin vs. 20 µM:Trypsin	14.56	1.292 to 27.83	Yes	*	0.0178
10 µM:Thermolysin vs. 20 µM:Chymotrypsin	19.44	6.169 to 32.71	Yes	***	0.0002
10 µM:Thermolysin vs. 20 µM:Subtilisin	5.371	-7.899 to 18.64	No	ns	0.9952
10 µM:Thermolysin vs. 20 µM:Thermolysin	14.03	0.7580 to 27.30	Yes	*	0.0276
10 µM:Thermolysin vs. 20 µM:Proteinase K	22.68	9.407 to 35.95	Yes	****	<0.0001
10 µM:Proteinase K vs. 20 µM:Pepsin	6.306	-6.964 to 19.58	No	ns	0.9698
10 µM:Proteinase K vs. 20 µM:Trypsin	11.67	-1.601 to 24.94	No	ns	0.1545

10 µM:Proteinase K vs. 20 µM:Chymotrypsin	16.55	3.276 to 29.82	Yes	**	0.0031
10 µM:Proteinase K vs. 20 µM:Subtilisin	2.478	-10.79 to 15.75	No	ns	>0.9999
10 µM:Proteinase K vs. 20 µM:Thermolysin	11.13	-2.135 to 24.40	No	ns	0.2148
10 µM:Proteinase K vs. 20 µM:Proteinase K	19.78	6.513 to 33.05	Yes	***	0.0001
20 µM:Pepsin vs. 20 µM:Trypsin	5.363	-7.907 to 18.63	No	ns	0.9953
20 µM:Pepsin vs. 20 µM:Chymotrypsin	10.24	-3.029 to 23.51	No	ns	0.3496
20 µM:Pepsin vs. 20 µM:Subtilisin	-3.828	-17.10 to 9.442	No	ns	>0.9999
20 µM:Pepsin vs. 20 µM:Thermolysin	4.829	-8.441 to 18.10	No	ns	0.9988
20 µM:Pepsin vs. 20 µM:Proteinase K	13.48	0.2079 to 26.75	Yes	*	0.0426
20 µM:Trypsin vs. 20 µM:Chymotrypsin	4.877	-8.392 to 18.15	No	ns	0.9987
20 µM:Trypsin vs. 20 µM:Subtilisin	-9.191	-22.46 to 4.079	No	ns	0.5519
20 µM:Trypsin vs. 20 µM:Thermolysin	-0.5341	-13.80 to 12.74	No	ns	>0.9999
20 µM:Trypsin vs. 20 µM:Proteinase K	8.114	-5.155 to 21.38	No	ns	0.7637
20 µM:Chymotrypsin vs. 20 µM:Subtilisin	-14.07	-27.34 to -0.7984	Yes	*	0.0267
20 µM:Chymotrypsin vs. 20 µM:Thermolysin	-5.411	-18.68 to 7.858	No	ns	0.9947
20 µM:Chymotrypsin vs. 20 µM:Proteinase K	3.237	-10.03 to 16.51	No	ns	>0.9999
20 µM:Subtilisin vs. 20 µM:Thermolysin	8.657	-4.613 to 21.93	No	ns	0.6606
20 µM:Subtilisin vs. 20 µM:Proteinase K	17.31	4.036 to 30.57	Yes	**	0.0015
20 µM:Thermolysin vs. 20 µM:Proteinase K	8.649	-4.621 to 21.92	No	ns	0.6622

Table S3. The viability data for *P. haitanensis* hydrolysates tested on CHO-K1 cell line by resazurin

**Sample (µM)**	**Control**			**PH - pepsin**			**PH - trypsin**			**PH - chymotrypsin**			**PH - subtilisin**			**PH - thermolysin**			**PH - proteinase K**		
**0**	98.14	101.26	100.60	x																	
**1**	x			100.94	102.17	100.66	110.11	101.73	101.75	98.46	97.61	101.71	92.89	95.72	100.20	108.32	104.69	101.15	102.57	102.39	105.67

**5**		97.90	103.51	112.97	126.08	114.01	110.12	105.84	100.65	99.11	96.13	96.05	100.22	106.80	106.48	107.48	102.01	102.24	81.80
**10**		102.43	103.74	117.79	128.88	118.90	123.97	120.47	108.16	108.91	97.32	97.87	101.38	110.76	107.32	105.13	100.88	101.62	85.06
**20**		110.71	112.12	120.20	103.77	106.80	98.07	116.04	118.69	112.51	82.00	91.57	83.02	96.76	98.07	100.79	95.87	95.29	80.41

Table S4A. One-way ANOVA statistical analysis for *P. haitanensis* hydrolysates tested on CHO-K1 cell line by resazurin

**ANOVA summary**					
**F**	5.737				
**P value**	<0.0001				
**P value summary**	****				
**Significant diff. among means (P < 0.05)?**	Yes				
**R square**	0.7336				
**ANOVA table**	**SS**	**DF**	**MS**	**F (DFn, DFd)**	**P value**
**Treatment (between columns)**	5352	24	223.0	F (24, 50) = 5.737	P<0.0001
**Residual (within columns)**	1944	50	38.87		
**Total**	7296	74			

Table S4B. Dunnett's multiple comparisons test for *P. haitanensis* hydrolysates tested on CHO-K1 cell line by resazurin

**Dunnett's multiple comparisons test**	**Mean Diff.**	**95.00% CI of diff.**	**Significant?**	**Summary**	**Adjusted P Value**
Control vs. Pepsin 1 µM	-1.258	-16.92 to 14.40	No	ns	0.9997
Control vs. Trypsin 1 µM	-4.530	-20.19 to 11.13	No	ns	0.9946
Control vs. Chymotrpysin 1 µM	0.7407	-14.92 to 16.40	No	ns	0.9998
Control vs. Pepsin 5 µM	-4.790	-20.45 to 10.87	No	ns	0.9940
Control vs. Trypsin 5 µM	-16.74	-32.40 to -1.076	Yes	*	0.0291
Control vs. Chymotrpysin 5 µM	-1.866	-17.53 to 13.79	No	ns	0.9995

Control vs. Pepsin 10 µM	-7.985	-23.64 to 7.675	No	ns	0.7653
Control vs. Trypsin 10 µM	-23.91	-39.57 to -8.254	Yes	***	0.0004
Control vs. Chymotrypsin 10 µM	-12.51	-28.17 to 3.149	No	ns	0.1995
Control vs. Pepsin 20 µM	-14.35	-30.01 to 1.313	No	ns	0.0924
Control vs. Trypsin 20 µM	-2.883	-18.54 to 12.78	No	ns	0.9992
Control vs. Chymotrypsin 20 µM	-15.75	-31.41 to -0.08728	Yes	*	0.0479
Control vs. Subtilisin 1 µM	3.733	-11.93 to 19.39	No	ns	0.9989
Control vs. Thermoylsin 1 µM	-4.719	-20.38 to 10.94	No	ns	0.9941
Control vs. Proteinase K 1 µM	-3.541	-19.20 to 12.12	No	ns	0.9990
Control vs. Subtilisin 5 µM	2.531	-13.13 to 18.19	No	ns	0.9993
Control vs. Thermolysin 5 µM	-6.923	-22.58 to 8.736	No	ns	0.8945
Control vs. Proteinase K 5 µM	4.652	-11.01 to 20.31	No	ns	0.9943
Control vs. Subtilisin 10 µM	1.144	-14.52 to 16.80	No	ns	0.9997
Control vs. Thermolysin 10 µM	-7.734	-23.39 to 7.926	No	ns	0.7993
Control vs. Proteinase K 10 µM	4.146	-11.51 to 19.81	No	ns	0.9954
Control vs. Subtilisin 20 µM	14.47	-1.190 to 30.13	No	ns	0.0874
Control vs. Thermolysin 20 µM	1.460	-14.20 to 17.12	No	ns	0.9996
Control vs. Proteinase K 20 µM	9.476	-6.184 to 25.14	No	ns	0.5459

Table S4C. Two-way ANOVA statistical analysis for *P. haitanensis* hydrolysates tested on CHO-K1 cell line by resazurin

**Two-way ANOVA**	Ordinary			
**Alpha**	0.05			
**Source of Variation**	**% of total variation**	**P value**	**P value summary**	**Significant?**
**Interaction**	29.72	<0.0001	****	Yes

**Row (concentration) Factor**	7.197	0.0033	**	Yes	
**Column (enzyme) Factor**	41.04	<0.0001	****	Yes	
**ANOVA table**	**SS**	**DF**	**MS**	**F (DFn, DFd)**	**P value**
**Interaction**	2026	15	135.1	F (15, 48) = 4.313	P<0.0001
**Row Factor**	490.7	3	163.6	F (3, 48) = 5.223	P=0.0033
**Column Factor**	2798	5	559.5	F (5, 48) = 17.87	P<0.0001
**Residual**	1503	48	31.32		

Table S4D. Tukey's multiple comparisons test for *P. haitanensis* hydrolysates tested on CHO-K1 cell line by resazurin

**Tukey's multiple comparisons test**	**Mean Diff.**	**95.00% CI of diff.**	**Significant?**	**Summary**	**Adjusted P Value**
1 µM:Pepsin vs. 1 µM:Trypsin	-3.272	-20.88 to 14.34	No	ns	>0.9999
1 µM:Pepsin vs. 1 µM:Chymotrypsin	1.998	-15.61 to 19.61	No	ns	>0.9999
1 µM:Pepsin vs. 1 µM:Subtilisin	4.991	-12.62 to 22.60	No	ns	>0.9999
1 µM:Pepsin vs. 1 µM:Thermolysin	-3.462	-21.07 to 14.15	No	ns	>0.9999
1 µM:Pepsin vs. 1 µM:Proteinase K	-2.283	-19.89 to 15.33	No	ns	>0.9999
1 µM:Pepsin vs. 5 µM:Pepsin	-3.532	-21.14 to 14.08	No	ns	>0.9999
1 µM:Pepsin vs. 5 µM:Trypsin	-15.48	-33.09 to 2.133	No	ns	0.1552
1 µM:Pepsin vs. 5 µM:Chymotrypsin	-0.6079	-18.22 to 17.00	No	ns	>0.9999
1 µM:Pepsin vs. 5 µM:Subtilisin	3.789	-13.82 to 21.40	No	ns	>0.9999
1 µM:Pepsin vs. 5 µM:Thermolysin	-5.666	-23.28 to 11.95	No	ns	0.9998
1 µM:Pepsin vs. 5 µM:Proteinase K	5.909	-11.70 to 23.52	No	ns	0.9996
1 µM:Pepsin vs. 10 µM:Pepsin	-6.727	-24.34 to 10.88	No	ns	0.9977
1 µM:Pepsin vs. 10 µM:Trypsin	-22.66	-40.27 to -5.044	Yes	**	0.0019
1 µM:Pepsin vs. 10 µM:Chymotrypsin	-11.25	-28.86 to 6.359	No	ns	0.6957

1 µM:Pepsin vs. 10 µM:Subtilisin	2.402	-15.21 to 20.01	No	ns	>0.9999
1 µM:Pepsin vs. 10 µM:Thermolysin	-6.476	-24.09 to 11.14	No	ns	0.9987
1 µM:Pepsin vs. 10 µM:Proteinase K	5.404	-12.21 to 23.02	No	ns	>0.9999
1 µM:Pepsin vs. 20 µM:Pepsin	-13.09	-30.70 to 4.523	No	ns	0.4185
1 µM:Pepsin vs. 20 µM:Trypsin	-1.625	-19.24 to 15.99	No	ns	>0.9999
1 µM:Pepsin vs. 20 µM:Chymotrypsin	-14.49	-32.10 to 3.123	No	ns	0.2436
1 µM:Pepsin vs. 20 µM:Subtilisin	15.73	-1.885 to 33.34	No	ns	0.1374
1 µM:Pepsin vs. 20 µM:Thermolysin	2.717	-14.89 to 20.33	No	ns	>0.9999
1 µM:Pepsin vs. 20 µM:Proteinase K	10.73	-6.878 to 28.35	No	ns	0.7686
1 µM:Trypsin vs. 1 µM:Chymotrypsin	5.270	-12.34 to 22.88	No	ns	>0.9999
1 µM:Trypsin vs. 1 µM:Subtilisin	8.263	-9.349 to 25.87	No	ns	0.9735
1 µM:Trypsin vs. 1 µM:Thermolysin	-0.1896	-17.80 to 17.42	No	ns	>0.9999
1 µM:Trypsin vs. 1 µM:Proteinase K	0.9891	-16.62 to 18.60	No	ns	>0.9999
1 µM:Trypsin vs. 5 µM:Pepsin	-0.2598	-17.87 to 17.35	No	ns	>0.9999
1 µM:Trypsin vs. 5 µM:Trypsin	-12.21	-29.82 to 5.406	No	ns	0.5507
1 µM:Trypsin vs. 5 µM:Chymotrypsin	2.664	-14.95 to 20.28	No	ns	>0.9999
1 µM:Trypsin vs. 5 µM:Subtilisin	7.061	-10.55 to 24.67	No	ns	0.9957
1 µM:Trypsin vs. 5 µM:Thermolysin	-2.393	-20.01 to 15.22	No	ns	>0.9999
1 µM:Trypsin vs. 5 µM:Proteinase K	9.182	-8.430 to 26.79	No	ns	0.9275
1 µM:Trypsin vs. 10 µM:Pepsin	-3.455	-21.07 to 14.16	No	ns	>0.9999
1 µM:Trypsin vs. 10 µM:Trypsin	-19.38	-37.00 to -1.772	Yes	*	0.0171
1 µM:Trypsin vs. 10 µM:Chymotrypsin	-7.981	-25.59 to 9.631	No	ns	0.9817
1 µM:Trypsin vs. 10 µM:Subtilisin	5.674	-11.94 to 23.29	No	ns	0.9998
1 µM:Trypsin vs. 10 µM:Thermolysin	-3.204	-20.82 to 14.41	No	ns	>0.9999

1 µM:Trypsin vs. 10 µM:Proteinase K	8.676	-8.936 to 26.29	No	ns	0.9568
1 µM:Trypsin vs. 20 µM:Pepsin	-9.817	-27.43 to 7.795	No	ns	0.8749
1 µM:Trypsin vs. 20 µM:Trypsin	1.647	-15.96 to 19.26	No	ns	>0.9999
1 µM:Trypsin vs. 20 µM:Chymotrypsin	-11.22	-28.83 to 6.395	No	ns	0.7010
1 µM:Trypsin vs. 20 µM:Subtilisin	19.00	1.388 to 36.61	Yes	*	0.0218
1 µM:Trypsin vs. 20 µM:Thermolysin	5.990	-11.62 to 23.60	No	ns	0.9996
1 µM:Trypsin vs. 20 µM:Proteinase K	14.01	-3.606 to 31.62	No	ns	0.2977
1 µM:Chymotrypsin vs. 1 µM:Subtilisin	2.992	-14.62 to 20.60	No	ns	>0.9999
1 µM:Chymotrypsin vs. 1 µM:Thermolysin	-5.460	-23.07 to 12.15	No	ns	0.9999
1 µM:Chymotrypsin vs. 1 µM:Proteinase K	-4.281	-21.89 to 13.33	No	ns	>0.9999
1 µM:Chymotrypsin vs. 5 µM:Pepsin	-5.530	-23.14 to 12.08	No	ns	0.9999
1 µM:Chymotrypsin vs. 5 µM:Trypsin	-17.48	-35.09 to 0.1352	No	ns	0.0540
1 µM:Chymotrypsin vs. 5 µM:Chymotrypsin	-2.606	-20.22 to 15.01	No	ns	>0.9999
1 µM:Chymotrypsin vs. 5 µM:Subtilisin	1.791	-15.82 to 19.40	No	ns	>0.9999
1 µM:Chymotrypsin vs. 5 µM:Thermolysin	-7.664	-25.28 to 9.948	No	ns	0.9884
1 µM:Chymotrypsin vs. 5 µM:Proteinase K	3.911	-13.70 to 21.52	No	ns	>0.9999
1 µM:Chymotrypsin vs. 10 µM:Pepsin	-8.725	-26.34 to 8.887	No	ns	0.9544
1 µM:Chymotrypsin vs. 10 µM:Trypsin	-24.65	-42.27 to -7.042	Yes	***	0.0005
1 µM:Chymotrypsin vs. 10 µM:Chymotrypsin	-13.25	-30.86 to 4.360	No	ns	0.3955
1 µM:Chymotrypsin vs. 10 µM:Subtilisin	0.4033	-17.21 to 18.02	No	ns	>0.9999
1 µM:Chymotrypsin vs. 10 µM:Thermolysin	-8.474	-26.09 to 9.137	No	ns	0.9657
1 µM:Chymotrypsin vs. 10 µM:Proteinase K	3.406	-14.21 to 21.02	No	ns	>0.9999
1 µM:Chymotrypsin vs. 20 µM:Pepsin	-15.09	-32.70 to 2.525	No	ns	0.1866
1 µM:Chymotrypsin vs. 20 µM:Trypsin	-3.623	-21.24 to 13.99	No	ns	>0.9999

1 µM:Chymotrypsin vs. 20 µM:Chymotrypsin	-16.49	-34.10 to 1.124	No	ns	0.0931
1 µM:Chymotrypsin vs. 20 µM:Subtilisin	13.73	-3.883 to 31.34	No	ns	0.3318
1 µM:Chymotrypsin vs. 20 µM:Thermolysin	0.7192	-16.89 to 18.33	No	ns	>0.9999
1 µM:Chymotrypsin vs. 20 µM:Proteinase K	8.735	-8.876 to 26.35	No	ns	0.9539
1 µM:Subtilisin vs. 1 µM:Thermolysin	-8.452	-26.06 to 9.159	No	ns	0.9666
1 µM:Subtilisin vs. 1 µM:Proteinase K	-7.274	-24.89 to 10.34	No	ns	0.9938
1 µM:Subtilisin vs. 5 µM:Pepsin	-8.523	-26.13 to 9.089	No	ns	0.9637
1 µM:Subtilisin vs. 5 µM:Trypsin	-20.47	-38.08 to -2.857	Yes	**	0.0085
1 µM:Subtilisin vs. 5 µM:Chymotrypsin	-5.599	-23.21 to 12.01	No	ns	0.9998
1 µM:Subtilisin vs. 5 µM:Subtilisin	-1.202	-18.81 to 16.41	No	ns	>0.9999
1 µM:Subtilisin vs. 5 µM:Thermolysin	-10.66	-28.27 to 6.955	No	ns	0.7787
1 µM:Subtilisin vs. 5 µM:Proteinase K	0.9189	-16.69 to 18.53	No	ns	>0.9999
1 µM:Subtilisin vs. 10 µM:Pepsin	-11.72	-29.33 to 5.894	No	ns	0.6259
1 µM:Subtilisin vs. 10 µM:Trypsin	-27.65	-45.26 to -10.03	Yes	****	<0.0001
1 µM:Subtilisin vs. 10 µM:Chymotrypsin	-16.24	-33.86 to 1.368	No	ns	0.1058
1 µM:Subtilisin vs. 10 µM:Subtilisin	-2.589	-20.20 to 15.02	No	ns	>0.9999
1 µM:Subtilisin vs. 10 µM:Thermolysin	-11.47	-29.08 to 6.145	No	ns	0.6640
1 µM:Subtilisin vs. 10 µM:Proteinase K	0.4133	-17.20 to 18.03	No	ns	>0.9999
1 µM:Subtilisin vs. 20 µM:Pepsin	-18.08	-35.69 to -0.4677	Yes	*	0.0381
1 µM:Subtilisin vs. 20 µM:Trypsin	-6.616	-24.23 to 11.00	No	ns	0.9982
1 µM:Subtilisin vs. 20 µM:Chymotrypsin	-19.48	-37.09 to -1.868	Yes	*	0.0161
1 µM:Subtilisin vs. 20 µM:Subtilisin	10.74	-6.875 to 28.35	No	ns	0.7682
1 µM:Subtilisin vs. 20 µM:Thermolysin	-2.273	-19.88 to 15.34	No	ns	>0.9999
1 µM:Subtilisin vs. 20 µM:Proteinase K	5.743	-11.87 to 23.35	No	ns	0.9998

1 µM:Thermolysin vs. 1 µM:Proteinase K	1.179	-16.43 to 18.79	No	ns	>0.9999
1 µM:Thermolysin vs. 5 µM:Pepsin	-0.07022	-17.68 to 17.54	No	ns	>0.9999
1 µM:Thermolysin vs. 5 µM:Trypsin	-12.02	-29.63 to 5.595	No	ns	0.5799
1 µM:Thermolysin vs. 5 µM:Chymotrypsin	2.854	-14.76 to 20.47	No	ns	>0.9999
1 µM:Thermolysin vs. 5 µM:Subtilisin	7.251	-10.36 to 24.86	No	ns	0.9940
1 µM:Thermolysin vs. 5 µM:Thermolysin	-2.204	-19.82 to 15.41	No	ns	>0.9999
1 µM:Thermolysin vs. 5 µM:Proteinase K	9.371	-8.241 to 26.98	No	ns	0.9137
1 µM:Thermolysin vs. 10 µM:Pepsin	-3.265	-20.88 to 14.35	No	ns	>0.9999
1 µM:Thermolysin vs. 10 µM:Trypsin	-19.19	-36.81 to -1.582	Yes	*	0.0193
1 µM:Thermolysin vs. 10 µM:Chymotrypsin	-7.791	-25.40 to 9.820	No	ns	0.9860
1 µM:Thermolysin vs. 10 µM:Subtilisin	5.863	-11.75 to 23.48	No	ns	0.9997
1 µM:Thermolysin vs. 10 µM:Thermolysin	-3.014	-20.63 to 14.60	No	ns	>0.9999
1 µM:Thermolysin vs. 10 µM:Proteinase K	8.866	-8.746 to 26.48	No	ns	0.9471
1 µM:Thermolysin vs. 20 µM:Pepsin	-9.627	-27.24 to 7.985	No	ns	0.8925
1 µM:Thermolysin vs. 20 µM:Trypsin	1.837	-15.78 to 19.45	No	ns	>0.9999
1 µM:Thermolysin vs. 20 µM:Chymotrypsin	-11.03	-28.64 to 6.584	No	ns	0.7282
1 µM:Thermolysin vs. 20 µM:Subtilisin	19.19	1.577 to 36.80	Yes	*	0.0194
1 µM:Thermolysin vs. 20 µM:Thermolysin	6.179	-11.43 to 23.79	No	ns	0.9993
1 µM:Thermolysin vs. 20 µM:Proteinase K	14.20	-3.416 to 31.81	No	ns	0.2757
1 µM:Proteinase K vs. 5 µM:Pepsin	-1.249	-18.86 to 16.36	No	ns	>0.9999
1 µM:Proteinase K vs. 5 µM:Trypsin	-13.20	-30.81 to 4.417	No	ns	0.4034
1 µM:Proteinase K vs. 5 µM:Chymotrypsin	1.675	-15.94 to 19.29	No	ns	>0.9999
1 µM:Proteinase K vs. 5 µM:Subtilisin	6.072	-11.54 to 23.68	No	ns	0.9995
1 µM:Proteinase K vs. 5 µM:Thermolysin	-3.383	-20.99 to 14.23	No	ns	>0.9999

1 µM:Proteinase K vs. 5 µM:Proteinase K	8.193	-9.419 to 25.80	No	ns	0.9758
1 µM:Proteinase K vs. 10 µM:Pepsin	-4.444	-22.06 to 13.17	No	ns	>0.9999
1 µM:Proteinase K vs. 10 µM:Trypsin	-20.37	-37.98 to -2.761	Yes	**	0.0091
1 µM:Proteinase K vs. 10 µM:Chymotrypsin	-8.970	-26.58 to 8.642	No	ns	0.9411
1 µM:Proteinase K vs. 10 µM:Subtilisin	4.685	-12.93 to 22.30	No	ns	>0.9999
1 µM:Proteinase K vs. 10 µM:Thermolysin	-4.193	-21.80 to 13.42	No	ns	>0.9999
1 µM:Proteinase K vs. 10 µM:Proteinase K	7.687	-9.925 to 25.30	No	ns	0.9880
1 µM:Proteinase K vs. 20 µM:Pepsin	-10.81	-28.42 to 6.806	No	ns	0.7589
1 µM:Proteinase K vs. 20 µM:Trypsin	0.6581	-16.95 to 18.27	No	ns	>0.9999
1 µM:Proteinase K vs. 20 µM:Chymotrypsin	-12.21	-29.82 to 5.406	No	ns	0.5507
1 µM:Proteinase K vs. 20 µM:Subtilisin	18.01	0.3985 to 35.62	Yes	*	0.0397
1 µM:Proteinase K vs. 20 µM:Thermolysin	5.001	-12.61 to 22.61	No	ns	>0.9999
1 µM:Proteinase K vs. 20 µM:Proteinase K	13.02	-4.595 to 30.63	No	ns	0.4288
5 µM:Pepsin vs. 5 µM:Trypsin	-11.95	-29.56 to 5.665	No	ns	0.5908
5 µM:Pepsin vs. 5 µM:Chymotrypsin	2.924	-14.69 to 20.54	No	ns	>0.9999
5 µM:Pepsin vs. 5 µM:Subtilisin	7.321	-10.29 to 24.93	No	ns	0.9933
5 µM:Pepsin vs. 5 µM:Thermolysin	-2.134	-19.75 to 15.48	No	ns	>0.9999
5 µM:Pepsin vs. 5 µM:Proteinase K	9.442	-8.170 to 27.05	No	ns	0.9081
5 µM:Pepsin vs. 10 µM:Pepsin	-3.195	-20.81 to 14.42	No	ns	>0.9999
5 µM:Pepsin vs. 10 µM:Trypsin	-19.12	-36.74 to -1.512	Yes	*	0.0202
5 µM:Pepsin vs. 10 µM:Chymotrypsin	-7.721	-25.33 to 9.891	No	ns	0.9874
5 µM:Pepsin vs. 10 µM:Subtilisin	5.934	-11.68 to 23.55	No	ns	0.9996
5 µM:Pepsin vs. 10 µM:Thermolysin	-2.944	-20.56 to 14.67	No	ns	>0.9999
5 µM:Pepsin vs. 10 µM:Proteinase K	8.936	-8.676 to 26.55	No	ns	0.9431

5 µM:Pepsin vs. 20 µM:Pepsin	-9.557	-27.17 to 8.055	No	ns	0.8986
5 µM:Pepsin vs. 20 µM:Trypsin	1.907	-15.70 to 19.52	No	ns	>0.9999
5 µM:Pepsin vs. 20 µM:Chymotrypsin	-10.96	-28.57 to 6.655	No	ns	0.7381
5 µM:Pepsin vs. 20 µM:Subtilisin	19.26	1.647 to 36.87	Yes	*	0.0185
5 µM:Pepsin vs. 20 µM:Thermolysin	6.250	-11.36 to 23.86	No	ns	0.9992
5 µM:Pepsin vs. 20 µM:Proteinase K	14.27	-3.346 to 31.88	No	ns	0.2678
5 µM:Trypsin vs. 5 µM:Chymotrypsin	14.87	-2.741 to 32.48	No	ns	0.2060
5 µM:Trypsin vs. 5 µM:Subtilisin	19.27	1.655 to 36.88	Yes	*	0.0184
5 µM:Trypsin vs. 5 µM:Thermolysin	9.813	-7.799 to 27.42	No	ns	0.8752
5 µM:Trypsin vs. 5 µM:Proteinase K	21.39	3.776 to 39.00	Yes	**	0.0046
5 µM:Trypsin vs. 10 µM:Pepsin	8.751	-8.860 to 26.36	No	ns	0.9531
5 µM:Trypsin vs. 10 µM:Trypsin	-7.177	-24.79 to 10.43	No	ns	0.9947
5 µM:Trypsin vs. 10 µM:Chymotrypsin	4.225	-13.39 to 21.84	No	ns	>0.9999
5 µM:Trypsin vs. 10 µM:Subtilisin	17.88	0.2681 to 35.49	Yes	*	0.0428
5 µM:Trypsin vs. 10 µM:Thermolysin	9.002	-8.610 to 26.61	No	ns	0.9391
5 µM:Trypsin vs. 10 µM:Proteinase K	20.88	3.270 to 38.49	Yes	**	0.0065
5 µM:Trypsin vs. 20 µM:Pepsin	2.389	-15.22 to 20.00	No	ns	>0.9999
5 µM:Trypsin vs. 20 µM:Trypsin	13.85	-3.758 to 31.47	No	ns	0.3162
5 µM:Trypsin vs. 20 µM:Chymotrypsin	0.9891	-16.62 to 18.60	No	ns	>0.9999
5 µM:Trypsin vs. 20 µM:Subtilisin	31.21	13.59 to 48.82	Yes	****	<0.0001
5 µM:Trypsin vs. 20 µM:Thermolysin	18.20	0.5841 to 35.81	Yes	*	0.0355
5 µM:Trypsin vs. 20 µM:Proteinase K	26.21	8.600 to 43.82	Yes	***	0.0001
5 µM:Chymotrypsin vs. 5 µM:Subtilisin	4.397	-13.22 to 22.01	No	ns	>0.9999
5 µM:Chymotrypsin vs. 5 µM:Thermolysin	-5.058	-22.67 to 12.55	No	ns	>0.9999

5 µM:Chymotrypsin vs. 5 µM:Proteinase K	6.517	-11.09 to 24.13	No	ns	0.9985
5 µM:Chymotrypsin vs. 10 µM:Pepsin	-6.119	-23.73 to 11.49	No	ns	0.9994
5 µM:Chymotrypsin vs. 10 µM:Trypsin	-22.05	-39.66 to -4.436	Yes	**	0.0029
5 µM:Chymotrypsin vs. 10 µM:Chymotrypsin	-10.65	-28.26 to 6.967	No	ns	0.7802
5 µM:Chymotrypsin vs. 10 µM:Subtilisin	3.009	-14.60 to 20.62	No	ns	>0.9999
5 µM:Chymotrypsin vs. 10 µM:Thermolysin	-5.868	-23.48 to 11.74	No	ns	0.9997
5 µM:Chymotrypsin vs. 10 µM:Proteinase K	6.012	-11.60 to 23.62	No	ns	0.9995
5 µM:Chymotrypsin vs. 20 µM:Pepsin	-12.48	-30.09 to 5.131	No	ns	0.5085
5 µM:Chymotrypsin vs. 20 µM:Trypsin	-1.017	-18.63 to 16.59	No	ns	>0.9999
5 µM:Chymotrypsin vs. 20 µM:Chymotrypsin	-13.88	-31.49 to 3.730	No	ns	0.3128
5 µM:Chymotrypsin vs. 20 µM:Subtilisin	16.34	-1.277 to 33.95	No	ns	0.1008
5 µM:Chymotrypsin vs. 20 µM:Thermolysin	3.325	-14.29 to 20.94	No	ns	>0.9999
5 µM:Chymotrypsin vs. 20 µM:Proteinase K	11.34	-6.270 to 28.95	No	ns	0.6827
5 µM:Subtilisin vs. 5 µM:Thermolysin	-9.455	-27.07 to 8.157	No	ns	0.9071
5 µM:Subtilisin vs. 5 µM:Proteinase K	2.121	-15.49 to 19.73	No	ns	>0.9999
5 µM:Subtilisin vs. 10 µM:Pepsin	-10.52	-28.13 to 7.096	No	ns	0.7967
5 µM:Subtilisin vs. 10 µM:Trypsin	-26.44	-44.06 to -8.833	Yes	***	0.0001
5 µM:Subtilisin vs. 10 µM:Chymotrypsin	-15.04	-32.65 to 2.570	No	ns	0.1906
5 µM:Subtilisin vs. 10 µM:Subtilisin	-1.387	-19.00 to 16.22	No	ns	>0.9999
5 µM:Subtilisin vs. 10 µM:Thermolysin	-10.27	-27.88 to 7.347	No	ns	0.8270
5 µM:Subtilisin vs. 10 µM:Proteinase K	1.615	-16.00 to 19.23	No	ns	>0.9999
5 µM:Subtilisin vs. 20 µM:Pepsin	-16.88	-34.49 to 0.7340	No	ns	0.0755
5 µM:Subtilisin vs. 20 µM:Trypsin	-5.414	-23.03 to 12.20	No	ns	>0.9999
5 µM:Subtilisin vs. 20 µM:Chymotrypsin	-18.28	-35.89 to -0.6663	Yes	*	0.0338

5 µM:Subtilisin vs. 20 µM:Subtilisin	11.94	-5.673 to 29.55	No	ns	0.5920
5 µM:Subtilisin vs. 20 µM:Thermolysin	-1.071	-18.68 to 16.54	No	ns	>0.9999
5 µM:Subtilisin vs. 20 µM:Proteinase K	6.945	-10.67 to 24.56	No	ns	0.9965
5 µM:Thermolysin vs. 5 µM:Proteinase K	11.58	-6.037 to 29.19	No	ns	0.6476
5 µM:Thermolysin vs. 10 µM:Pepsin	-1.061	-18.67 to 16.55	No	ns	>0.9999
5 µM:Thermolysin vs. 10 µM:Trypsin	-16.99	-34.60 to 0.6217	No	ns	0.0709
5 µM:Thermolysin vs. 10 µM:Chymotrypsin	-5.587	-23.20 to 12.02	No	ns	0.9999
5 µM:Thermolysin vs. 10 µM:Subtilisin	8.067	-9.545 to 25.68	No	ns	0.9795
5 µM:Thermolysin vs. 10 µM:Thermolysin	-0.8105	-18.42 to 16.80	No	ns	>0.9999
5 µM:Thermolysin vs. 10 µM:Proteinase K	11.07	-6.542 to 28.68	No	ns	0.7222
5 µM:Thermolysin vs. 20 µM:Pepsin	-7.423	-25.04 to 10.19	No	ns	0.9920
5 µM:Thermolysin vs. 20 µM:Trypsin	4.041	-13.57 to 21.65	No	ns	>0.9999
5 µM:Thermolysin vs. 20 µM:Chymotrypsin	-8.824	-26.44 to 8.788	No	ns	0.9494
5 µM:Thermolysin vs. 20 µM:Subtilisin	21.39	3.781 to 39.00	Yes	**	0.0046
5 µM:Thermolysin vs. 20 µM:Thermolysin	8.383	-9.229 to 26.00	No	ns	0.9693
5 µM:Thermolysin vs. 20 µM:Proteinase K	16.40	-1.213 to 34.01	No	ns	0.0975
5 µM:Proteinase K vs. 10 µM:Pepsin	-12.64	-30.25 to 4.975	No	ns	0.4850
5 µM:Proteinase K vs. 10 µM:Trypsin	-28.57	-46.18 to -10.95	Yes	****	<0.0001
5 µM:Proteinase K vs. 10 µM:Chymotrypsin	-17.16	-34.77 to 0.4491	No	ns	0.0645
5 µM:Proteinase K vs. 10 µM:Subtilisin	-3.508	-21.12 to 14.10	No	ns	>0.9999
5 µM:Proteinase K vs. 10 µM:Thermolysin	-12.39	-30.00 to 5.226	No	ns	0.5231
5 µM:Proteinase K vs. 10 µM:Proteinase K	-0.5056	-18.12 to 17.11	No	ns	>0.9999
5 µM:Proteinase K vs. 20 µM:Pepsin	-19.00	-36.61 to -1.387	Yes	*	0.0218
5 µM:Proteinase K vs. 20 µM:Trypsin	-7.535	-25.15 to 10.08	No	ns	0.9905

5 µM:Proteinase K vs. 20 µM:Chymotrypsin	-20.40	-38.01 to -2.787	Yes	**	0.0089
5 µM:Proteinase K vs. 20 µM:Subtilisin	9.818	-7.794 to 27.43	No	ns	0.8748
5 µM:Proteinase K vs. 20 µM:Thermolysin	-3.192	-20.80 to 14.42	No	ns	>0.9999
5 µM:Proteinase K vs. 20 µM:Proteinase K	4.824	-12.79 to 22.44	No	ns	>0.9999
10 µM:Pepsin vs. 10 µM:Trypsin	-15.93	-33.54 to 1.683	No	ns	0.1242
10 µM:Pepsin vs. 10 µM:Chymotrypsin	-4.526	-22.14 to 13.09	No	ns	>0.9999
10 µM:Pepsin vs. 10 µM:Subtilisin	9.129	-8.483 to 26.74	No	ns	0.9311
10 µM:Pepsin vs. 10 µM:Thermolysin	0.2508	-17.36 to 17.86	No	ns	>0.9999
10 µM:Pepsin vs. 10 µM:Proteinase K	12.13	-5.481 to 29.74	No	ns	0.5623
10 µM:Pepsin vs. 20 µM:Pepsin	-6.362	-23.97 to 11.25	No	ns	0.9990
10 µM:Pepsin vs. 20 µM:Trypsin	5.102	-12.51 to 22.71	No	ns	>0.9999
10 µM:Pepsin vs. 20 µM:Chymotrypsin	-7.762	-25.37 to 9.850	No	ns	0.9866
10 µM:Pepsin vs. 20 µM:Subtilisin	22.45	4.842 to 40.07	Yes	**	0.0022
10 µM:Pepsin vs. 20 µM:Thermolysin	9.445	-8.167 to 27.06	No	ns	0.9079
10 µM:Pepsin vs. 20 µM:Proteinase K	17.46	-0.1512 to 35.07	No	ns	0.0545
10 µM:Trypsin vs. 10 µM:Chymotrypsin	11.40	-6.209 to 29.01	No	ns	0.6736
10 µM:Trypsin vs. 10 µM:Subtilisin	25.06	7.446 to 42.67	Yes	***	0.0003
10 µM:Trypsin vs. 10 µM:Thermolysin	16.18	-1.432 to 33.79	No	ns	0.1093
10 µM:Trypsin vs. 10 µM:Proteinase K	28.06	10.45 to 45.67	Yes	****	<0.0001
10 µM:Trypsin vs. 20 µM:Pepsin	9.567	-8.045 to 27.18	No	ns	0.8977
10 µM:Trypsin vs. 20 µM:Trypsin	21.03	3.419 to 38.64	Yes	**	0.0059
10 µM:Trypsin vs. 20 µM:Chymotrypsin	8.167	-9.445 to 25.78	No	ns	0.9766
10 µM:Trypsin vs. 20 µM:Subtilisin	38.38	20.77 to 55.99	Yes	****	<0.0001
10 µM:Trypsin vs. 20 µM:Thermolysin	25.37	7.762 to 42.99	Yes	***	0.0003

10 µM:Trypsin vs. 20 µM:Proteinase K	33.39	15.78 to 51.00	Yes	****	<0.0001
10 µM:Chymotrypsin vs. 10 µM:Subtilisin	13.65	-3.957 to 31.27	No	ns	0.3413
10 µM:Chymotrypsin vs. 10 µM:Thermolysin	4.777	-12.83 to 22.39	No	ns	>0.9999
10 µM:Chymotrypsin vs. 10 µM:Proteinase K	16.66	-0.9547 to 34.27	No	ns	0.0850
10 µM:Chymotrypsin vs. 20 µM:Pepsin	-1.836	-19.45 to 15.78	No	ns	>0.9999
10 µM:Chymotrypsin vs. 20 µM:Trypsin	9.628	-7.984 to 27.24	No	ns	0.8924
10 µM:Chymotrypsin vs. 20 µM:Chymotrypsin	-3.236	-20.85 to 14.38	No	ns	>0.9999
10 µM:Chymotrypsin vs. 20 µM:Subtilisin	26.98	9.369 to 44.59	Yes	****	<0.0001
10 µM:Chymotrypsin vs. 20 µM:Thermolysin	13.97	-3.641 to 31.58	No	ns	0.3019
10 µM:Chymotrypsin vs. 20 µM:Proteinase K	21.99	4.375 to 39.60	Yes	**	0.0031
10 µM:Subtilisin vs. 10 µM:Thermolysin	-8.878	-26.49 to 8.734	No	ns	0.9464
10 µM:Subtilisin vs. 10 µM:Proteinase K	3.002	-14.61 to 20.61	No	ns	>0.9999
10 µM:Subtilisin vs. 20 µM:Pepsin	-15.49	-33.10 to 2.121	No	ns	0.1543
10 µM:Subtilisin vs. 20 µM:Trypsin	-4.027	-21.64 to 13.59	No	ns	>0.9999
10 µM:Subtilisin vs. 20 µM:Chymotrypsin	-16.89	-34.50 to 0.7210	No	ns	0.0749
10 µM:Subtilisin vs. 20 µM:Subtilisin	13.33	-4.286 to 30.94	No	ns	0.3853
10 µM:Subtilisin vs. 20 µM:Thermolysin	0.3160	-17.30 to 17.93	No	ns	>0.9999
10 µM:Subtilisin vs. 20 µM:Proteinase K	8.332	-9.280 to 25.94	No	ns	0.9711
10 µM:Thermolysin vs. 10 µM:Proteinase K	11.88	-5.732 to 29.49	No	ns	0.6010
10 µM:Thermolysin vs. 20 µM:Pepsin	-6.613	-24.22 to 11.00	No	ns	0.9982
10 µM:Thermolysin vs. 20 µM:Trypsin	4.851	-12.76 to 22.46	No	ns	>0.9999
10 µM:Thermolysin vs. 20 µM:Chymotrypsin	-8.013	-25.62 to 9.599	No	ns	0.9809
10 µM:Thermolysin vs. 20 µM:Subtilisin	22.20	4.592 to 39.82	Yes	**	0.0026
10 µM:Thermolysin vs. 20 µM:Thermolysin	9.194	-8.418 to 26.81	No	ns	0.9267

10 µM:Thermolysin vs. 20 µM:Proteinase K	17.21	-0.4020 to 34.82	No	ns	0.0628
10 µM:Proteinase K vs. 20 µM:Pepsin	-18.49	-36.10 to -0.8810	Yes	*	0.0297
10 µM:Proteinase K vs. 20 µM:Trypsin	-7.029	-24.64 to 10.58	No	ns	0.9960
10 µM:Proteinase K vs. 20 µM:Chymotrypsin	-19.89	-37.51 to -2.281	Yes	*	0.0124
10 µM:Proteinase K vs. 20 µM:Subtilisin	10.32	-7.289 to 27.94	No	ns	0.8202
10 µM:Proteinase K vs. 20 µM:Thermolysin	-2.686	-20.30 to 14.93	No	ns	>0.9999
10 µM:Proteinase K vs. 20 µM:Proteinase K	5.330	-12.28 to 22.94	No	ns	>0.9999
20 µM:Pepsin vs. 20 µM:Trypsin	11.46	-6.148 to 29.08	No	ns	0.6644
20 µM:Pepsin vs. 20 µM:Chymotrypsin	-1.400	-19.01 to 16.21	No	ns	>0.9999
20 µM:Pepsin vs. 20 µM:Subtilisin	28.82	11.20 to 46.43	Yes	****	<0.0001
20 µM:Pepsin vs. 20 µM:Thermolysin	15.81	-1.805 to 33.42	No	ns	0.1321
20 µM:Pepsin vs. 20 µM:Proteinase K	23.82	6.211 to 41.43	Yes	***	0.0008
20 µM:Trypsin vs. 20 µM:Chymotrypsin	-12.86	-30.48 to 4.748	No	ns	0.4511
20 µM:Trypsin vs. 20 µM:Subtilisin	17.35	-0.2596 to 34.96	No	ns	0.0580
20 µM:Trypsin vs. 20 µM:Thermolysin	4.343	-13.27 to 21.95	No	ns	>0.9999
20 µM:Trypsin vs. 20 µM:Proteinase K	12.36	-5.253 to 29.97	No	ns	0.5272
20 µM:Chymotrypsin vs. 20 µM:Subtilisin	30.22	12.60 to 47.83	Yes	****	<0.0001
20 µM:Chymotrypsin vs. 20 µM:Thermolysin	17.21	-0.4050 to 34.82	No	ns	0.0629
20 µM:Chymotrypsin vs. 20 µM:Proteinase K	25.22	7.611 to 42.83	Yes	***	0.0003
20 µM:Subtilisin vs. 20 µM:Thermolysin	-13.01	-30.62 to 4.602	No	ns	0.4299
20 µM:Subtilisin vs. 20 µM:Proteinase K	-4.994	-22.61 to 12.62	No	ns	>0.9999
20 µM:Thermolysin vs. 20 µM:Proteinase K	8.016	-9.596 to 25.63	No	ns	0.9808

Table S5. The viability data for *A. platensis* hydrolysates tested on QM7 cell line by resazurin assay

Sample (µM)	Control			AP - pepsin			AP - trypsin			AP - chymotrypsin			AP - subtilisin			AP - thermolysin			AP - proteinase K		
**0**	98.10	100.21	101.69	x																	
**1**	x			82.93	60.68	73.13	105.78	102.89	103.69	117.63	105.96	103.86	71.77	74.92	69.27	82.35	86.08	74.57	83.43	86.83	97.34
**5**				93.08	75.93	76.71	98.85	94.2	111.9	105.68	113.48	109.78	96.46	84.59	94.83	98.14	101.12	92.13	96.13	94.92	104.92
**10**				94.63	91.5	90.79	102.94	91.29	93.24	107.52	99.61	110.15	98.39	90.74	117.31	104.04	105.03	92.69	93.5	98.13	101.23
**20**				96.5	73.37	96.25	90.7	82.6	83.72	89.26	98.72	102.65	99.74	92.28	103.87	100.92	96.48	96.75	98.37	93.51	90.51

Table S6A. One-way ANOVA statistical analysis for *A. platensis* hydrolysates tested on QM7 cell line by resazurin assay

**ANOVA summary**					
**F**	6.102				
**P value**	<0.0001				
**P value summary**	****				
**Significant diff. among means (P < 0.05)?**	Yes				
**R square**	0.7455				
**ANOVA table**	**SS**	**DF**	**MS**	**F (DFn, DFd)**	**P value**
**Treatment (between columns)**	6954	24	289.8	F (24, 50) = 6.102	P<0.0001
**Residual (within columns)**	2374	50	47.48		
**Total**	9328	74			

Table S6B. Dunnett's multiple comparisons test for for *A. platensis* hydrolysates tested on QM7 cell line by resazurin assay

Dunnett's multiple comparisons test	Mean Diff.	95.00% CI of diff.	Significant?	Summary	Adjusted P Value

Control vs. Pepsin 1 µM	27.75	10.44 to 45.06	Yes	***	0.0002
Control vs. Trypsin 1 µM	-4.120	-21.43 to 13.19	No	ns	0.9989
Control vs. Chymotrpysin 1 µM	-7.790	-25.10 to 9.517	No	ns	0.8816
Control vs. Pepsin 5 µM	18.09	0.7832 to 35.40	Yes	*	0.0352
Control vs. Trypsin 5 µM	-1.650	-18.96 to 15.66	No	ns	0.9996
Control vs. Chymotrpysin 5 µM	-10.21	-27.52 to 7.097	No	ns	0.5808
Control vs. Pepsin 10 µM	7.700	-9.607 to 25.01	No	ns	0.8901
Control vs. Trypsin 10 µM	4.170	-13.14 to 21.48	No	ns	0.9989
Control vs. Chymotrypsin 10 µM	-2.630	-19.94 to 14.68	No	ns	0.9994
Control vs. Pepsin 20 µM	11.29	-6.017 to 28.60	No	ns	0.4422
Control vs. Trypsin 20 µM	14.33	-2.977 to 31.64	No	ns	0.1666
Control vs. Chymotrypsin 20 µM	6.410	-10.90 to 23.72	No	ns	0.9736
Control vs. Subtilisin 1 µM	28.01	10.71 to 45.32	Yes	***	0.0002
Control vs. Thermoylsin 1 µM	19.00	1.693 to 36.31	Yes	*	0.0230
Control vs. Proteinase K 1 µM	10.80	-6.505 to 28.11	No	ns	0.5032
Control vs. Subtilisin 5 µM	8.037	-9.269 to 25.34	No	ns	0.8570
Control vs. Thermolysin 5 µM	2.868	-14.44 to 20.17	No	ns	0.9993
Control vs. Proteinase K 5 µM	1.345	-15.96 to 18.65	No	ns	0.9997
Control vs. Subtilisin 10 µM	-2.149	-19.46 to 15.16	No	ns	0.9995
Control vs. Thermolysin 10 µM	-0.5864	-17.89 to 16.72	No	ns	0.9999
Control vs. Proteinase K 10 µM	2.382	-14.92 to 19.69	No	ns	0.9994
Control vs. Subtilisin 20 µM	1.371	-15.94 to 18.68	No	ns	0.9997
Control vs. Thermolysin 20 µM	1.950	-15.36 to 19.26	No	ns	0.9996
Control vs. Proteinase K 20 µM	5.870	-11.44 to 23.18	No	ns	0.9866

Table S6C. Two-way ANOVA statistical analysis for *A. platensis* hydrolysates tested on QM7 cell line by resazurin assay

**Two-way ANOVA**	Ordinary				
**Alpha**	0.05				
**Source of Variation**	**% of total variation**	**P value**	**P value summary**	**Significant?**	
**Interaction**	30.37	0.0002	***	Yes	
**Row (concentration) Factor**	13.19	0.0001	***	Yes	
**Column (enzyme) Factor**	31.50	<0.0001	****	Yes	
**ANOVA table**	**SS**	**DF**	**MS**	**F (DFn, DFd)**	**P value**
**Interaction**	2883	15	192.2	F (15, 48) = 3.895	P=0.0002
**Row Factor**	1252	3	417.2	F (3, 48) = 8.456	P=0.0001
**Column Factor**	2989	5	597.9	F (5, 48) = 12.12	P<0.0001
**Residual**	2368	48	49.33		

Table S6D. Tukey's multiple comparisons test for *A. platensis* hydrolysates tested on QM7 cell line by resazurin assay

Tukey's multiple comparisons test	Mean Diff.	95.00% CI of diff.	Significant?	Summary	Adjusted P Value
1 µM:Pepsin vs. 1 µM:Trypsin	-31.87	-53.98 to -9.769	Yes	***	0.0003
1 µM:Pepsin vs. 1 µM:Chymotrypsin	-36.90	-59.01 to -14.80	Yes	****	<0.0001
1 µM:Pepsin vs. 1 µM:Subtilisin	0.2600	-21.84 to 22.36	No	ns	>0.9999
1 µM:Pepsin vs. 1 µM:Thermolysin	-8.753	-30.86 to 13.35	No	ns	0.9964
1 µM:Pepsin vs. 1 µM:Proteinase K	-16.95	-39.06 to 5.151	No	ns	0.3607
1 µM:Pepsin vs. 5 µM:Pepsin	-9.660	-31.76 to 12.44	No	ns	0.9878
1 µM:Pepsin vs. 5 µM:Trypsin	-29.40	-51.51 to -7.299	Yes	**	0.0011
1 µM:Pepsin vs. 5 µM:Chymotrypsin	-37.40	-59.50 to -15.30	Yes	****	<0.0001
1 µM:Pepsin vs. 5 µM:Subtilisin	-19.71	-41.82 to 2.391	No	ns	0.1388

1 µM:Pepsin vs. 5 µM:Thermolysin	-24.88	-46.99 to -2.779	Yes	*	0.0129
1 µM:Pepsin vs. 5 µM:Proteinase K	-26.41	-48.51 to -4.305	Yes	**	0.0058
1 µM:Pepsin vs. 10 µM:Pepsin	-20.06	-42.16 to 2.045	No	ns	0.1209
1 µM:Pepsin vs. 10 µM:Trypsin	-23.58	-45.68 to -1.472	Yes	*	0.0249
1 µM:Pepsin vs. 10 µM:Chymotrypsin	-33.51	-55.62 to -11.41	Yes	***	0.0001
1 µM:Pepsin vs. 10 µM:Subtilisin	-29.90	-52.00 to -7.795	Yes	***	0.0008
1 µM:Pepsin vs. 10 µM:Thermolysin	-28.34	-50.44 to -6.235	Yes	**	0.0020
1 µM:Pepsin vs. 10 µM:Proteinase K	-25.37	-47.48 to -3.269	Yes	*	0.0100
1 µM:Pepsin vs. 20 µM:Pepsin	-16.46	-38.56 to 5.645	No	ns	0.4148
1 µM:Pepsin vs. 20 µM:Trypsin	-13.43	-35.53 to 8.678	No	ns	0.7733
1 µM:Pepsin vs. 20 µM:Chymotrypsin	-24.63	-46.73 to -2.525	Yes	*	0.0147
1 µM:Pepsin vs. 20 µM:Subtilisin	-26.38	-48.49 to -4.279	Yes	**	0.0059
1 µM:Pepsin vs. 20 µM:Thermolysin	-25.80	-47.91 to -3.699	Yes	**	0.0080
1 µM:Pepsin vs. 20 µM:Proteinase K	-21.88	-43.99 to 0.2214	No	ns	0.0553
1 µM:Trypsin vs. 1 µM:Chymotrypsin	-5.030	-27.13 to 17.07	No	ns	>0.9999
1 µM:Trypsin vs. 1 µM:Subtilisin	32.13	10.03 to 54.24	Yes	***	0.0002
1 µM:Trypsin vs. 1 µM:Thermolysin	23.12	1.015 to 45.22	Yes	*	0.0311
1 µM:Trypsin vs. 1 µM:Proteinase K	14.92	-7.185 to 37.02	No	ns	0.5998
1 µM:Trypsin vs. 5 µM:Pepsin	22.21	0.1086 to 44.32	Yes	*	0.0476
1 µM:Trypsin vs. 5 µM:Trypsin	2.470	-19.63 to 24.57	No	ns	>0.9999
1 µM:Trypsin vs. 5 µM:Chymotrypsin	-5.527	-27.63 to 16.58	No	ns	>0.9999
1 µM:Trypsin vs. 5 µM:Subtilisin	12.16	-9.945 to 34.26	No	ns	0.8869
1 µM:Trypsin vs. 5 µM:Thermolysin	6.990	-15.11 to 29.09	No	ns	0.9999
1 µM:Trypsin vs. 5 µM:Proteinase K	5.463	-16.64 to 27.57	No	ns	>0.9999

1 µM:Trypsin vs. 10 µM:Pepsin	11.81	-10.29 to 33.92	No	ns	0.9105
1 µM:Trypsin vs. 10 µM:Trypsin	8.297	-13.81 to 30.40	No	ns	0.9982
1 µM:Trypsin vs. 10 µM:Chymotrypsin	-1.640	-23.74 to 20.46	No	ns	>0.9999
1 µM:Trypsin vs. 10 µM:Subtilisin	1.973	-20.13 to 24.08	No	ns	>0.9999
1 µM:Trypsin vs. 10 µM:Thermolysin	3.533	-18.57 to 25.64	No	ns	>0.9999
1 µM:Trypsin vs. 10 µM:Proteinase K	6.500	-15.60 to 28.60	No	ns	>0.9999
1 µM:Trypsin vs. 20 µM:Pepsin	15.41	-6.691 to 37.52	No	ns	0.5392
1 µM:Trypsin vs. 20 µM:Trypsin	18.45	-3.658 to 40.55	No	ns	0.2226
1 µM:Trypsin vs. 20 µM:Chymotrypsin	7.243	-14.86 to 29.35	No	ns	0.9998
1 µM:Trypsin vs. 20 µM:Subtilisin	5.490	-16.61 to 27.59	No	ns	>0.9999
1 µM:Trypsin vs. 20 µM:Thermolysin	6.070	-16.03 to 28.17	No	ns	>0.9999
1 µM:Trypsin vs. 20 µM:Proteinase K	9.990	-12.11 to 32.09	No	ns	0.9822
1 µM:Chymotrypsin vs. 1 µM:Subtilisin	37.16	15.06 to 59.27	Yes	****	<0.0001
1 µM:Chymotrypsin vs. 1 µM:Thermolysin	28.15	6.045 to 50.25	Yes	**	0.0022
1 µM:Chymotrypsin vs. 1 µM:Proteinase K	19.95	-2.155 to 42.05	No	ns	0.1264
1 µM:Chymotrypsin vs. 5 µM:Pepsin	27.24	5.139 to 49.35	Yes	**	0.0037
1 µM:Chymotrypsin vs. 5 µM:Trypsin	7.500	-14.60 to 29.60	No	ns	0.9996
1 µM:Chymotrypsin vs. 5 µM:Chymotrypsin	-0.4967	-22.60 to 21.61	No	ns	>0.9999
1 µM:Chymotrypsin vs. 5 µM:Subtilisin	17.19	-4.915 to 39.29	No	ns	0.3360
1 µM:Chymotrypsin vs. 5 µM:Thermolysin	12.02	-10.08 to 34.12	No	ns	0.8969
1 µM:Chymotrypsin vs. 5 µM:Proteinase K	10.49	-11.61 to 32.60	No	ns	0.9701
1 µM:Chymotrypsin vs. 10 µM:Pepsin	16.84	-5.261 to 38.95	No	ns	0.3724
1 µM:Chymotrypsin vs. 10 µM:Trypsin	13.33	-8.778 to 35.43	No	ns	0.7837
1 µM:Chymotrypsin vs. 10 µM:Chymotrypsin	3.390	-18.71 to 25.49	No	ns	>0.9999

1 µM:Chymotrypsin vs. 10 µM:Subtilisin	7.003	-15.10 to 29.11	No	ns	0.9999
1 µM:Chymotrypsin vs. 10 µM:Thermolysin	8.563	-13.54 to 30.67	No	ns	0.9973
1 µM:Chymotrypsin vs. 10 µM:Proteinase K	11.53	-10.57 to 33.63	No	ns	0.9272
1 µM:Chymotrypsin vs. 20 µM:Pepsin	20.44	-1.661 to 42.55	No	ns	0.1033
1 µM:Chymotrypsin vs. 20 µM:Trypsin	23.48	1.372 to 45.58	Yes	*	0.0261
1 µM:Chymotrypsin vs. 20 µM:Chymotrypsin	12.27	-9.831 to 34.38	No	ns	0.8785
1 µM:Chymotrypsin vs. 20 µM:Subtilisin	10.52	-11.58 to 32.62	No	ns	0.9693
1 µM:Chymotrypsin vs. 20 µM:Thermolysin	11.10	-11.00 to 33.20	No	ns	0.9483
1 µM:Chymotrypsin vs. 20 µM:Proteinase K	15.02	-7.085 to 37.12	No	ns	0.5876
1 µM:Subtilisin vs. 1 µM:Thermolysin	-9.013	-31.12 to 13.09	No	ns	0.9947
1 µM:Subtilisin vs. 1 µM:Proteinase K	-17.21	-39.32 to 4.891	No	ns	0.3337
1 µM:Subtilisin vs. 5 µM:Pepsin	-9.920	-32.02 to 12.18	No	ns	0.9836
1 µM:Subtilisin vs. 5 µM:Trypsin	-29.66	-51.77 to -7.559	Yes	***	0.0010
1 µM:Subtilisin vs. 5 µM:Chymotrypsin	-37.66	-59.76 to -15.56	Yes	****	<0.0001
1 µM:Subtilisin vs. 5 µM:Subtilisin	-19.97	-42.08 to 2.131	No	ns	0.1252
1 µM:Subtilisin vs. 5 µM:Thermolysin	-25.14	-47.25 to -3.039	Yes	*	0.0113
1 µM:Subtilisin vs. 5 µM:Proteinase K	-26.67	-48.77 to -4.565	Yes	**	0.0051
1 µM:Subtilisin vs. 10 µM:Pepsin	-20.32	-42.42 to 1.785	No	ns	0.1088
1 µM:Subtilisin vs. 10 µM:Trypsin	-23.84	-45.94 to -1.732	Yes	*	0.0219
1 µM:Subtilisin vs. 10 µM:Chymotrypsin	-33.77	-55.88 to -11.67	Yes	****	<0.0001
1 µM:Subtilisin vs. 10 µM:Subtilisin	-30.16	-52.26 to -8.055	Yes	***	0.0007
1 µM:Subtilisin vs. 10 µM:Thermolysin	-28.60	-50.70 to -6.495	Yes	**	0.0017
1 µM:Subtilisin vs. 10 µM:Proteinase K	-25.63	-47.74 to -3.529	Yes	**	0.0088
1 µM:Subtilisin vs. 20 µM:Pepsin	-16.72	-38.82 to 5.385	No	ns	0.3858

1 µM:Subtilisin vs. 20 µM:Trypsin	-13.69	-35.79 to 8.418	No	ns	0.7453
1 µM:Subtilisin vs. 20 µM:Chymotrypsin	-24.89	-46.99 to -2.785	Yes	*	0.0129
1 µM:Subtilisin vs. 20 µM:Subtilisin	-26.64	-48.75 to -4.539	Yes	**	0.0051
1 µM:Subtilisin vs. 20 µM:Thermolysin	-26.06	-48.17 to -3.959	Yes	**	0.0070
1 µM:Subtilisin vs. 20 µM:Proteinase K	-22.14	-44.25 to -0.03859	Yes	*	0.0491
1 µM:Thermolysin vs. 1 µM:Proteinase K	-8.200	-30.30 to 13.90	No	ns	0.9985
1 µM:Thermolysin vs. 5 µM:Pepsin	-0.9067	-23.01 to 21.20	No	ns	>0.9999
1 µM:Thermolysin vs. 5 µM:Trypsin	-20.65	-42.75 to 1.455	No	ns	0.0948
1 µM:Thermolysin vs. 5 µM:Chymotrypsin	-28.65	-50.75 to -6.542	Yes	**	0.0017
1 µM:Thermolysin vs. 5 µM:Subtilisin	-10.96	-33.06 to 11.14	No	ns	0.9541
1 µM:Thermolysin vs. 5 µM:Thermolysin	-16.13	-38.23 to 5.975	No	ns	0.4529
1 µM:Thermolysin vs. 5 µM:Proteinase K	-17.66	-39.76 to 4.448	No	ns	0.2904
1 µM:Thermolysin vs. 10 µM:Pepsin	-11.31	-33.41 to 10.80	No	ns	0.9387
1 µM:Thermolysin vs. 10 µM:Trypsin	-14.82	-36.93 to 7.281	No	ns	0.6117
1 µM:Thermolysin vs. 10 µM:Chymotrypsin	-24.76	-46.86 to -2.655	Yes	*	0.0138
1 µM:Thermolysin vs. 10 µM:Subtilisin	-21.15	-43.25 to 0.9581	No	ns	0.0767
1 µM:Thermolysin vs. 10 µM:Thermolysin	-19.59	-41.69 to 2.518	No	ns	0.1459
1 µM:Thermolysin vs. 10 µM:Proteinase K	-16.62	-38.72 to 5.485	No	ns	0.3969
1 µM:Thermolysin vs. 20 µM:Pepsin	-7.707	-29.81 to 14.40	No	ns	0.9994
1 µM:Thermolysin vs. 20 µM:Trypsin	-4.673	-26.78 to 17.43	No	ns	>0.9999
1 µM:Thermolysin vs. 20 µM:Chymotrypsin	-15.88	-37.98 to 6.228	No	ns	0.4830
1 µM:Thermolysin vs. 20 µM:Subtilisin	-17.63	-39.73 to 4.475	No	ns	0.2929
1 µM:Thermolysin vs. 20 µM:Thermolysin	-17.05	-39.15 to 5.055	No	ns	0.3505
1 µM:Thermolysin vs. 20 µM:Proteinase K	-13.13	-35.23 to 8.975	No	ns	0.8035

1 µM:Proteinase K vs. 5 µM:Pepsin	7.293	-14.81 to 29.40	No	ns	0.9997
1 µM:Proteinase K vs. 5 µM:Trypsin	-12.45	-34.55 to 9.655	No	ns	0.8647
1 µM:Proteinase K vs. 5 µM:Chymotrypsin	-20.45	-42.55 to 1.658	No	ns	0.1032
1 µM:Proteinase K vs. 5 µM:Subtilisin	-2.760	-24.86 to 19.34	No	ns	>0.9999
1 µM:Proteinase K vs. 5 µM:Thermolysin	-7.930	-30.03 to 14.17	No	ns	0.9991
1 µM:Proteinase K vs. 5 µM:Proteinase K	-9.457	-31.56 to 12.65	No	ns	0.9905
1 µM:Proteinase K vs. 10 µM:Pepsin	-3.107	-25.21 to 19.00	No	ns	>0.9999
1 µM:Proteinase K vs. 10 µM:Trypsin	-6.623	-28.73 to 15.48	No	ns	>0.9999
1 µM:Proteinase K vs. 10 µM:Chymotrypsin	-16.56	-38.66 to 5.545	No	ns	0.4036
1 µM:Proteinase K vs. 10 µM:Subtilisin	-12.95	-35.05 to 9.158	No	ns	0.8212
1 µM:Proteinase K vs. 10 µM:Thermolysin	-11.39	-33.49 to 10.72	No	ns	0.9347
1 µM:Proteinase K vs. 10 µM:Proteinase K	-8.420	-30.52 to 13.68	No	ns	0.9978
1 µM:Proteinase K vs. 20 µM:Pepsin	0.4933	-21.61 to 22.60	No	ns	>0.9999
1 µM:Proteinase K vs. 20 µM:Trypsin	3.527	-18.58 to 25.63	No	ns	>0.9999
1 µM:Proteinase K vs. 20 µM:Chymotrypsin	-7.677	-29.78 to 14.43	No	ns	0.9994
1 µM:Proteinase K vs. 20 µM:Subtilisin	-9.430	-31.53 to 12.67	No	ns	0.9908
1 µM:Proteinase K vs. 20 µM:Thermolysin	-8.850	-30.95 to 13.25	No	ns	0.9958
1 µM:Proteinase K vs. 20 µM:Proteinase K	-4.930	-27.03 to 17.17	No	ns	>0.9999
5 µM:Pepsin vs. 5 µM:Trypsin	-19.74	-41.85 to 2.361	No	ns	0.1372
5 µM:Pepsin vs. 5 µM:Chymotrypsin	-27.74	-49.84 to -5.635	Yes	**	0.0028
5 µM:Pepsin vs. 5 µM:Subtilisin	-10.05	-32.16 to 12.05	No	ns	0.9810
5 µM:Pepsin vs. 5 µM:Thermolysin	-15.22	-37.33 to 6.881	No	ns	0.5625
5 µM:Pepsin vs. 5 µM:Proteinase K	-16.75	-38.85 to 5.355	No	ns	0.3825
5 µM:Pepsin vs. 10 µM:Pepsin	-10.40	-32.50 to 11.70	No	ns	0.9727

5 µM:Pepsin vs. 10 µM:Trypsin	-13.92	-36.02 to 8.188	No	ns	0.7196
5 µM:Pepsin vs. 10 µM:Chymotrypsin	-23.85	-45.96 to -1.749	Yes	*	0.0217
5 µM:Pepsin vs. 10 µM:Subtilisin	-20.24	-42.34 to 1.865	No	ns	0.1124
5 µM:Pepsin vs. 10 µM:Thermolysin	-18.68	-40.78 to 3.425	No	ns	0.2049
5 µM:Pepsin vs. 10 µM:Proteinase K	-15.71	-37.82 to 6.391	No	ns	0.5027
5 µM:Pepsin vs. 20 µM:Pepsin	-6.800	-28.90 to 15.30	No	ns	>0.9999
5 µM:Pepsin vs. 20 µM:Trypsin	-3.767	-25.87 to 18.34	No	ns	>0.9999
5 µM:Pepsin vs. 20 µM:Chymotrypsin	-14.97	-37.07 to 7.135	No	ns	0.5937
5 µM:Pepsin vs. 20 µM:Subtilisin	-16.72	-38.83 to 5.381	No	ns	0.3855
5 µM:Pepsin vs. 20 µM:Thermolysin	-16.14	-38.25 to 5.961	No	ns	0.4514
5 µM:Pepsin vs. 20 µM:Proteinase K	-12.22	-34.33 to 9.881	No	ns	0.8823
5 µM:Trypsin vs. 5 µM:Chymotrypsin	-7.997	-30.10 to 14.11	No	ns	0.9989
5 µM:Trypsin vs. 5 µM:Subtilisin	9.690	-12.41 to 31.79	No	ns	0.9874
5 µM:Trypsin vs. 5 µM:Thermolysin	4.520	-17.58 to 26.62	No	ns	>0.9999
5 µM:Trypsin vs. 5 µM:Proteinase K	2.993	-19.11 to 25.10	No	ns	>0.9999
5 µM:Trypsin vs. 10 µM:Pepsin	9.343	-12.76 to 31.45	No	ns	0.9918
5 µM:Trypsin vs. 10 µM:Trypsin	5.827	-16.28 to 27.93	No	ns	>0.9999
5 µM:Trypsin vs. 10 µM:Chymotrypsin	-4.110	-26.21 to 17.99	No	ns	>0.9999
5 µM:Trypsin vs. 10 µM:Subtilisin	-0.4967	-22.60 to 21.61	No	ns	>0.9999
5 µM:Trypsin vs. 10 µM:Thermolysin	1.063	-21.04 to 23.17	No	ns	>0.9999
5 µM:Trypsin vs. 10 µM:Proteinase K	4.030	-18.07 to 26.13	No	ns	>0.9999
5 µM:Trypsin vs. 20 µM:Pepsin	12.94	-9.161 to 35.05	No	ns	0.8215
5 µM:Trypsin vs. 20 µM:Trypsin	15.98	-6.128 to 38.08	No	ns	0.4711
5 µM:Trypsin vs. 20 µM:Chymotrypsin	4.773	-17.33 to 26.88	No	ns	>0.9999

5 µM:Trypsin vs. 20 µM:Subtilisin	3.020	-19.08 to 25.12	No	ns	>0.9999
5 µM:Trypsin vs. 20 µM:Thermolysin	3.600	-18.50 to 25.70	No	ns	>0.9999
5 µM:Trypsin vs. 20 µM:Proteinase K	7.520	-14.58 to 29.62	No	ns	0.9996
5 µM:Chymotrypsin vs. 5 µM:Subtilisin	17.69	-4.418 to 39.79	No	ns	0.2876
5 µM:Chymotrypsin vs. 5 µM:Thermolysin	12.52	-9.588 to 34.62	No	ns	0.8592
5 µM:Chymotrypsin vs. 5 µM:Proteinase K	10.99	-11.11 to 33.09	No	ns	0.9529
5 µM:Chymotrypsin vs. 10 µM:Pepsin	17.34	-4.765 to 39.44	No	ns	0.3209
5 µM:Chymotrypsin vs. 10 µM:Trypsin	13.82	-8.281 to 35.93	No	ns	0.7301
5 µM:Chymotrypsin vs. 10 µM:Chymotrypsin	3.887	-18.22 to 25.99	No	ns	>0.9999
5 µM:Chymotrypsin vs. 10 µM:Subtilisin	7.500	-14.60 to 29.60	No	ns	0.9996
5 µM:Chymotrypsin vs. 10 µM:Thermolysin	9.060	-13.04 to 31.16	No	ns	0.9944
5 µM:Chymotrypsin vs. 10 µM:Proteinase K	12.03	-10.08 to 34.13	No	ns	0.8964
5 µM:Chymotrypsin vs. 20 µM:Pepsin	20.94	-1.165 to 43.04	No	ns	0.0838
5 µM:Chymotrypsin vs. 20 µM:Trypsin	23.97	1.869 to 46.08	Yes	*	0.0205
5 µM:Chymotrypsin vs. 20 µM:Chymotrypsin	12.77	-9.335 to 34.87	No	ns	0.8374
5 µM:Chymotrypsin vs. 20 µM:Subtilisin	11.02	-11.09 to 33.12	No	ns	0.9518
5 µM:Chymotrypsin vs. 20 µM:Thermolysin	11.60	-10.51 to 33.70	No	ns	0.9234
5 µM:Chymotrypsin vs. 20 µM:Proteinase K	15.52	-6.588 to 37.62	No	ns	0.5266
5 µM:Subtilisin vs. 5 µM:Thermolysin	-5.170	-27.27 to 16.93	No	ns	>0.9999
5 µM:Subtilisin vs. 5 µM:Proteinase K	-6.697	-28.80 to 15.41	No	ns	>0.9999
5 µM:Subtilisin vs. 10 µM:Pepsin	-0.3467	-22.45 to 21.76	No	ns	>0.9999
5 µM:Subtilisin vs. 10 µM:Trypsin	-3.863	-25.97 to 18.24	No	ns	>0.9999
5 µM:Subtilisin vs. 10 µM:Chymotrypsin	-13.80	-35.90 to 8.305	No	ns	0.7328
5 µM:Subtilisin vs. 10 µM:Subtilisin	-10.19	-32.29 to 11.92	No	ns	0.9781

5 µM:Subtilisin vs. 10 µM:Thermolysin	-8.627	-30.73 to 13.48	No	ns	0.9970
5 µM:Subtilisin vs. 10 µM:Proteinase K	-5.660	-27.76 to 16.44	No	ns	>0.9999
5 µM:Subtilisin vs. 20 µM:Pepsin	3.253	-18.85 to 25.36	No	ns	>0.9999
5 µM:Subtilisin vs. 20 µM:Trypsin	6.287	-15.82 to 28.39	No	ns	>0.9999
5 µM:Subtilisin vs. 20 µM:Chymotrypsin	-4.917	-27.02 to 17.19	No	ns	>0.9999
5 µM:Subtilisin vs. 20 µM:Subtilisin	-6.670	-28.77 to 15.43	No	ns	>0.9999
5 µM:Subtilisin vs. 20 µM:Thermolysin	-6.090	-28.19 to 16.01	No	ns	>0.9999
5 µM:Subtilisin vs. 20 µM:Proteinase K	-2.170	-24.27 to 19.93	No	ns	>0.9999
5 µM:Thermolysin vs. 5 µM:Proteinase K	-1.527	-23.63 to 20.58	No	ns	>0.9999
5 µM:Thermolysin vs. 10 µM:Pepsin	4.823	-17.28 to 26.93	No	ns	>0.9999
5 µM:Thermolysin vs. 10 µM:Trypsin	1.307	-20.80 to 23.41	No	ns	>0.9999
5 µM:Thermolysin vs. 10 µM:Chymotrypsin	-8.630	-30.73 to 13.47	No	ns	0.9970
5 µM:Thermolysin vs. 10 µM:Subtilisin	-5.017	-27.12 to 17.09	No	ns	>0.9999
5 µM:Thermolysin vs. 10 µM:Thermolysin	-3.457	-25.56 to 18.65	No	ns	>0.9999
5 µM:Thermolysin vs. 10 µM:Proteinase K	-0.4900	-22.59 to 21.61	No	ns	>0.9999
5 µM:Thermolysin vs. 20 µM:Pepsin	8.423	-13.68 to 30.53	No	ns	0.9978
5 µM:Thermolysin vs. 20 µM:Trypsin	11.46	-10.65 to 33.56	No	ns	0.9311
5 µM:Thermolysin vs. 20 µM:Chymotrypsin	0.2533	-21.85 to 22.36	No	ns	>0.9999
5 µM:Thermolysin vs. 20 µM:Subtilisin	-1.500	-23.60 to 20.60	No	ns	>0.9999
5 µM:Thermolysin vs. 20 µM:Thermolysin	-0.9200	-23.02 to 21.18	No	ns	>0.9999
5 µM:Thermolysin vs. 20 µM:Proteinase K	3.000	-19.10 to 25.10	No	ns	>0.9999
5 µM:Proteinase K vs. 10 µM:Pepsin	6.350	-15.75 to 28.45	No	ns	>0.9999
5 µM:Proteinase K vs. 10 µM:Trypsin	2.833	-19.27 to 24.94	No	ns	>0.9999
5 µM:Proteinase K vs. 10 µM:Chymotrypsin	-7.103	-29.21 to 15.00	No	ns	0.9998

5 µM:Proteinase K vs. 10 µM:Subtilisin	-3.490	-25.59 to 18.61	No	ns	>0.9999
5 µM:Proteinase K vs. 10 µM:Thermolysin	-1.930	-24.03 to 20.17	No	ns	>0.9999
5 µM:Proteinase K vs. 10 µM:Proteinase K	1.037	-21.07 to 23.14	No	ns	>0.9999
5 µM:Proteinase K vs. 20 µM:Pepsin	9.950	-12.15 to 32.05	No	ns	0.9830
5 µM:Proteinase K vs. 20 µM:Trypsin	12.98	-9.121 to 35.09	No	ns	0.8177
5 µM:Proteinase K vs. 20 µM:Chymotrypsin	1.780	-20.32 to 23.88	No	ns	>0.9999
5 µM:Proteinase K vs. 20 µM:Subtilisin	0.02667	-22.08 to 22.13	No	ns	>0.9999
5 µM:Proteinase K vs. 20 µM:Thermolysin	0.6067	-21.50 to 22.71	No	ns	>0.9999
5 µM:Proteinase K vs. 20 µM:Proteinase K	4.527	-17.58 to 26.63	No	ns	>0.9999
10 µM:Pepsin vs. 10 µM:Trypsin	-3.517	-25.62 to 18.59	No	ns	>0.9999
10 µM:Pepsin vs. 10 µM:Chymotrypsin	-13.45	-35.56 to 8.651	No	ns	0.7705
10 µM:Pepsin vs. 10 µM:Subtilisin	-9.840	-31.94 to 12.26	No	ns	0.9850
10 µM:Pepsin vs. 10 µM:Thermolysin	-8.280	-30.38 to 13.82	No	ns	0.9983
10 µM:Pepsin vs. 10 µM:Proteinase K	-5.313	-27.42 to 16.79	No	ns	>0.9999
10 µM:Pepsin vs. 20 µM:Pepsin	3.600	-18.50 to 25.70	No	ns	>0.9999
10 µM:Pepsin vs. 20 µM:Trypsin	6.633	-15.47 to 28.74	No	ns	>0.9999
10 µM:Pepsin vs. 20 µM:Chymotrypsin	-4.570	-26.67 to 17.53	No	ns	>0.9999
10 µM:Pepsin vs. 20 µM:Subtilisin	-6.323	-28.43 to 15.78	No	ns	>0.9999
10 µM:Pepsin vs. 20 µM:Thermolysin	-5.743	-27.85 to 16.36	No	ns	>0.9999
10 µM:Pepsin vs. 20 µM:Proteinase K	-1.823	-23.93 to 20.28	No	ns	>0.9999
10 µM:Trypsin vs. 10 µM:Chymotrypsin	-9.937	-32.04 to 12.17	No	ns	0.9833
10 µM:Trypsin vs. 10 µM:Subtilisin	-6.323	-28.43 to 15.78	No	ns	>0.9999
10 µM:Trypsin vs. 10 µM:Thermolysin	-4.763	-26.87 to 17.34	No	ns	>0.9999
10 µM:Trypsin vs. 10 µM:Proteinase K	-1.797	-23.90 to 20.31	No	ns	>0.9999

10 µM:Trypsin vs. 20 µM:Pepsin	7.117	-14.99 to 29.22	No	ns	0.9998
10 µM:Trypsin vs. 20 µM:Trypsin	10.15	-11.95 to 32.25	No	ns	0.9789
10 µM:Trypsin vs. 20 µM:Chymotrypsin	-1.053	-23.16 to 21.05	No	ns	>0.9999
10 µM:Trypsin vs. 20 µM:Subtilisin	-2.807	-24.91 to 19.30	No	ns	>0.9999
10 µM:Trypsin vs. 20 µM:Thermolysin	-2.227	-24.33 to 19.88	No	ns	>0.9999
10 µM:Trypsin vs. 20 µM:Proteinase K	1.693	-20.41 to 23.80	No	ns	>0.9999
10 µM:Chymotrypsin vs. 10 µM:Subtilisin	3.613	-18.49 to 25.72	No	ns	>0.9999
10 µM:Chymotrypsin vs. 10 µM:Thermolysin	5.173	-16.93 to 27.28	No	ns	>0.9999
10 µM:Chymotrypsin vs. 10 µM:Proteinase K	8.140	-13.96 to 30.24	No	ns	0.9986
10 µM:Chymotrypsin vs. 20 µM:Pepsin	17.05	-5.051 to 39.16	No	ns	0.3501
10 µM:Chymotrypsin vs. 20 µM:Trypsin	20.09	-2.018 to 42.19	No	ns	0.1196
10 µM:Chymotrypsin vs. 20 µM:Chymotrypsin	8.883	-13.22 to 30.99	No	ns	0.9956
10 µM:Chymotrypsin vs. 20 µM:Subtilisin	7.130	-14.97 to 29.23	No	ns	0.9998
10 µM:Chymotrypsin vs. 20 µM:Thermolysin	7.710	-14.39 to 29.81	No	ns	0.9994
10 µM:Chymotrypsin vs. 20 µM:Proteinase K	11.63	-10.47 to 33.73	No	ns	0.9215
10 µM:Subtilisin vs. 10 µM:Thermolysin	1.560	-20.54 to 23.66	No	ns	>0.9999
10 µM:Subtilisin vs. 10 µM:Proteinase K	4.527	-17.58 to 26.63	No	ns	>0.9999
10 µM:Subtilisin vs. 20 µM:Pepsin	13.44	-8.665 to 35.54	No	ns	0.7719
10 µM:Subtilisin vs. 20 µM:Trypsin	16.47	-5.631 to 38.58	No	ns	0.4133
10 µM:Subtilisin vs. 20 µM:Chymotrypsin	5.270	-16.83 to 27.37	No	ns	>0.9999
10 µM:Subtilisin vs. 20 µM:Subtilisin	3.517	-18.59 to 25.62	No	ns	>0.9999
10 µM:Subtilisin vs. 20 µM:Thermolysin	4.097	-18.01 to 26.20	No	ns	>0.9999
10 µM:Subtilisin vs. 20 µM:Proteinase K	8.017	-14.09 to 30.12	No	ns	0.9989
10 µM:Thermolysin vs. 10 µM:Proteinase K	2.967	-19.14 to 25.07	No	ns	>0.9999

10 µM:Thermolysin vs. 20 µM:Pepsin	11.88	-10.22 to 33.98	No	ns	0.9062
10 µM:Thermolysin vs. 20 µM:Trypsin	14.91	-7.191 to 37.02	No	ns	0.6007
10 µM:Thermolysin vs. 20 µM:Chymotrypsin	3.710	-18.39 to 25.81	No	ns	>0.9999
10 µM:Thermolysin vs. 20 µM:Subtilisin	1.957	-20.15 to 24.06	No	ns	>0.9999
10 µM:Thermolysin vs. 20 µM:Thermolysin	2.537	-19.57 to 24.64	No	ns	>0.9999
10 µM:Thermolysin vs. 20 µM:Proteinase K	6.457	-15.65 to 28.56	No	ns	>0.9999
10 µM:Proteinase K vs. 20 µM:Pepsin	8.913	-13.19 to 31.02	No	ns	0.9954
10 µM:Proteinase K vs. 20 µM:Trypsin	11.95	-10.16 to 34.05	No	ns	0.9018
10 µM:Proteinase K vs. 20 µM:Chymotrypsin	0.7433	-21.36 to 22.85	No	ns	>0.9999
10 µM:Proteinase K vs. 20 µM:Subtilisin	-1.010	-23.11 to 21.09	No	ns	>0.9999
10 µM:Proteinase K vs. 20 µM:Thermolysin	-0.4300	-22.53 to 21.67	No	ns	>0.9999
10 µM:Proteinase K vs. 20 µM:Proteinase K	3.490	-18.61 to 25.59	No	ns	>0.9999
20 µM:Pepsin vs. 20 µM:Trypsin	3.033	-19.07 to 25.14	No	ns	>0.9999
20 µM:Pepsin vs. 20 µM:Chymotrypsin	-8.170	-30.27 to 13.93	No	ns	0.9986
20 µM:Pepsin vs. 20 µM:Subtilisin	-9.923	-32.03 to 12.18	No	ns	0.9835
20 µM:Pepsin vs. 20 µM:Thermolysin	-9.343	-31.45 to 12.76	No	ns	0.9918
20 µM:Pepsin vs. 20 µM:Proteinase K	-5.423	-27.53 to 16.68	No	ns	>0.9999
20 µM:Trypsin vs. 20 µM:Chymotrypsin	-11.20	-33.31 to 10.90	No	ns	0.9436
20 µM:Trypsin vs. 20 µM:Subtilisin	-12.96	-35.06 to 9.148	No	ns	0.8202
20 µM:Trypsin vs. 20 µM:Thermolysin	-12.38	-34.48 to 9.728	No	ns	0.8705
20 µM:Trypsin vs. 20 µM:Proteinase K	-8.457	-30.56 to 13.65	No	ns	0.9977
20 µM:Chymotrypsin vs. 20 µM:Subtilisin	-1.753	-23.86 to 20.35	No	ns	>0.9999
20 µM:Chymotrypsin vs. 20 µM:Thermolysin	-1.173	-23.28 to 20.93	No	ns	>0.9999
20 µM:Chymotrypsin vs. 20 µM:Proteinase K	2.747	-19.36 to 24.85	No	ns	>0.9999

20 µM:Subtilisin vs. 20 µM:Thermolysin 0.5800 -21.52 to 22.68 No ns >0.9999

20 µM:Subtilisin vs. 20 µM:Proteinase K 4.500 -17.60 to 26.60 No ns >0.9999

20 µM:Thermolysin vs. 20 µM:Proteinase K 3.920 -18.18 to 26.02 No ns >0.9999

Table S7. The viability data for *P. haitanensis* hydrolysates tested on QM7 cell line by resazurin assay

Sample (µM)	Control			PH - pepsin			PH - trypsin			PH - chymotrypsin			PH - subtilisin			PH - thermolysin			PH - proteinase K		
**0**	96.21	102.24	101.55	x																	
**1**	x			84.79	78.61	75.33	102.54	105.12	106.48	94.73	91.38	100.41	93.97	62.07	60.04	107.59	108.54	104.66	108.88	97.37	99.74
**5**				84.07	86.30	88.49	105.33	106.06	98.71	97.99	103.28	103.00	102.12	66.86	80.47	102.83	108.97	111.88	103.78	116.06	115.14
**10**				96.04	98.52	89.77	110.23	108.78	104.18	107.83	98.93	112.63	101.17	77.12	91.12	86.53	93.14	89.90	99.63	115.40	109.82
**20**				118.16	111.97	88.42	103.13	112.21	111.28	108.14	104.40	99.47	102.32	76.02	83.76	95.33	97.81	89.67	22.87	18.46	20.99

Table S8A. One-way ANOVA statistical analysis for *P. haitanensis* hydrolysates tested on QM7 cell line by resazurin assay

ANOVA summary

F 15.59

P value <0.0001

P value summary ****

Significant diff. among means (P < 0.05)? Yes

R square 0.8821

**ANOVA table**	**SS**	**DF**	**MS**	**F (DFn, DFd)**	**P value**
**Treatment (between columns)**	25015	24	1042	F (24, 50) = 15.59	P<0.0001
**Residual (within columns)**	3342	50	66.85		
**Total**	28358	74			

Table S8B. Dunnett's multiple comparisons test for *P. haitanensis* hydrolysates tested on QM7 cell line by resazurin assay

Dunnett's multiple comparisons test	Mean Diff.	95.00% CI of diff.	Significant?	Summary	Adjusted P Value
Control vs. Pepsin 1 µM	20.43	-0.1086 to 40.96	No	ns	0.0520
Control vs. Trypsin 1 µM	-4.714	-25.25 to 15.82	No	ns	0.9990
Control vs. Chymotrpysin 1 µM	4.493	-16.04 to 25.03	No	ns	0.9990
Control vs. Pepsin 5 µM	13.71	-6.823 to 34.25	No	ns	0.4109
Control vs. Trypsin 5 µM	-3.366	-23.90 to 17.17	No	ns	0.9993
Control vs. Chymotrpysin 5 µM	-1.423	-21.96 to 19.11	No	ns	0.9997
Control vs. Pepsin 10 µM	5.223	-15.31 to 25.76	No	ns	0.9958
Control vs. Trypsin 10 µM	-7.732	-28.27 to 12.80	No	ns	0.9693
Control vs. Chymotrypsin 10 µM	-6.463	-27.00 to 14.07	No	ns	0.9891
Control vs. Pepsin 20 µM	-6.180	-26.72 to 14.36	No	ns	0.9942
Control vs. Trypsin 20 µM	-8.873	-29.41 to 11.66	No	ns	0.9095
Control vs. Chymotrypsin 20 µM	-4.004	-24.54 to 16.53	No	ns	0.9991
Control vs. Subtilisin 1 µM	27.97	7.435 to 48.51	Yes	**	0.0022
Control vs. Thermoylsin 1 µM	-6.930	-27.47 to 13.61	No	ns	0.9868
Control vs. Proteinase K 1 µM	-2.000	-22.54 to 18.54	No	ns	0.9996
Control vs. Subtilisin 5 µM	16.85	-3.685 to 37.39	No	ns	0.1746
Control vs. Thermolysin 5 µM	-7.890	-28.43 to 12.65	No	ns	0.9630
Control vs. Proteinase K 5 µM	-11.66	-32.20 to 8.875	No	ns	0.6325
Control vs. Subtilisin 10 µM	10.20	-10.34 to 30.74	No	ns	0.7934
Control vs. Thermolysin 10 µM	10.15	-10.39 to 30.69	No	ns	0.7985
Control vs. Proteinase K 10 µM	-8.280	-28.82 to 12.26	No	ns	0.9452
Control vs. Subtilisin 20 µM	12.63	-7.905 to 33.17	No	ns	0.5234

Control vs. Thermolysin 20 µM	5.730	-14.81 to 26.27	No	ns	0.9949
Control vs. Proteinase K 20 µM	79.23	58.69 to 99.77	Yes	****	<0.0001

Table S8C. Two-way ANOVA statistical analysis for *P. haitanensis* hydrolysates tested on QM7 cell line by resazurin assay

**Two-way ANOVA**	Ordinary				
**Alpha**	0.05				
**Source of Variation**	**% of total variation**	**P value**	**P value summary**	**Significant?**	
**Interaction**	63.27	<0.0001	****	Yes	
**Row (concentration) Factor**	6.580	<0.0001	****	Yes	
**Column (enzyme) Factor**	18.40	<0.0001	****	Yes	
**ANOVA table**	**SS**	**DF**	**MS**	**F (DFn, DFd)**	**P value**
**Interaction**	17876	15	1192	F (15, 48) = 17.23	P<0.0001
**Row Factor**	1859	3	619.7	F (3, 48) = 8.960	P<0.0001
**Column Factor**	5199	5	1040	F (5, 48) = 15.03	P<0.0001
**Residual**	3320	48	69.16		

Table S8D. Tukey's multiple comparisons test for *P. haitanensis* hydrolysates tested on QM7 cell line by resazurin assay

**Tukey's multiple comparisons test**	**Mean Diff.**	**95.00% CI of diff.**	**Significant?**	**Summary**	**Adjusted P Value**
1 µM:Pepsin vs. 1 µM:Trypsin	-25.14	-51.31 to 1.033	No	ns	0.0739
1 µM:Pepsin vs. 1 µM:Chymotrypsin	-15.93	-42.11 to 10.24	No	ns	0.7701

1 µM:Pepsin vs. 1 µM:Subtilisin	7.546	-18.63 to 33.72	No	ns	>0.9999
1 µM:Pepsin vs. 1 µM:Thermolysin	-27.35	-53.53 to -1.180	Yes	*	0.0313
1 µM:Pepsin vs. 1 µM:Proteinase K	-22.43	-48.60 to 3.747	No	ns	0.1864
1 µM:Pepsin vs. 5 µM:Pepsin	-6.714	-32.89 to 19.46	No	ns	>0.9999
1 µM:Pepsin vs. 5 µM:Trypsin	-23.79	-49.97 to 2.380	No	ns	0.1192
1 µM:Pepsin vs. 5 µM:Chymotrypsin	-21.85	-48.02 to 4.324	No	ns	0.2221
1 µM:Pepsin vs. 5 µM:Subtilisin	-3.579	-29.75 to 22.59	No	ns	>0.9999
1 µM:Pepsin vs. 5 µM:Thermolysin	-28.32	-54.49 to -2.146	Yes	*	0.0211
1 µM:Pepsin vs. 5 µM:Proteinase K	-32.09	-58.26 to -5.914	Yes	**	0.0040
1 µM:Pepsin vs. 10 µM:Pepsin	-15.20	-41.38 to 10.97	No	ns	0.8310
1 µM:Pepsin vs. 10 µM:Trypsin	-28.16	-54.33 to -1.985	Yes	*	0.0225
1 µM:Pepsin vs. 10 µM:Chymotrypsin	-26.89	-53.06 to -0.7163	Yes	*	0.0377
1 µM:Pepsin vs. 10 µM:Subtilisin	-10.23	-36.40 to 15.94	No	ns	0.9969
1 µM:Pepsin vs. 10 µM:Thermolysin	-10.28	-36.45 to 15.89	No	ns	0.9967
1 µM:Pepsin vs. 10 µM:Proteinase K	-28.71	-54.88 to -2.538	Yes	*	0.0178
1 µM:Pepsin vs. 20 µM:Pepsin	-26.61	-52.78 to -0.4335	Yes	*	0.0422
1 µM:Pepsin vs. 20 µM:Trypsin	-29.30	-55.47 to -3.127	Yes	*	0.0139
1 µM:Pepsin vs. 20 µM:Chymotrypsin	-24.43	-50.60 to 1.743	No	ns	0.0955
1 µM:Pepsin vs. 20 µM:Subtilisin	-7.793	-33.97 to 18.38	No	ns	>0.9999
1 µM:Pepsin vs. 20 µM:Thermolysin	-14.70	-40.87 to 11.47	No	ns	0.8676
1 µM:Pepsin vs. 20 µM:Proteinase K	58.80	32.63 to 84.97	Yes	****	<0.0001
1 µM:Trypsin vs. 1 µM:Chymotrypsin	9.207	-16.97 to 35.38	No	ns	0.9993
1 µM:Trypsin vs. 1 µM:Subtilisin	32.69	6.513 to 58.86	Yes	**	0.0030
1 µM:Trypsin vs. 1 µM:Thermolysin	-2.213	-28.39 to 23.96	No	ns	>0.9999

1 µM:Trypsin vs. 1 µM:Proteinase K	2.714	-23.46 to 28.89	No	ns	>0.9999
1 µM:Trypsin vs. 5 µM:Pepsin	18.43	-7.747 to 44.60	No	ns	0.5211
1 µM:Trypsin vs. 5 µM:Trypsin	1.347	-24.83 to 27.52	No	ns	>0.9999
1 µM:Trypsin vs. 5 µM:Chymotrypsin	3.291	-22.88 to 29.46	No	ns	>0.9999
1 µM:Trypsin vs. 5 µM:Subtilisin	21.56	-4.612 to 47.73	No	ns	0.2416
1 µM:Trypsin vs. 5 µM:Thermolysin	-3.178	-29.35 to 22.99	No	ns	>0.9999
1 µM:Trypsin vs. 5 µM:Proteinase K	-6.946	-33.12 to 19.23	No	ns	>0.9999
1 µM:Trypsin vs. 10 µM:Pepsin	9.937	-16.24 to 36.11	No	ns	0.9979
1 µM:Trypsin vs. 10 µM:Trypsin	-3.018	-29.19 to 23.15	No	ns	>0.9999
1 µM:Trypsin vs. 10 µM:Chymotrypsin	-1.749	-27.92 to 24.42	No	ns	>0.9999
1 µM:Trypsin vs. 10 µM:Subtilisin	14.91	-11.26 to 41.08	No	ns	0.8528
1 µM:Trypsin vs. 10 µM:Thermolysin	14.86	-11.31 to 41.03	No	ns	0.8564
1 µM:Trypsin vs. 10 µM:Proteinase K	-3.571	-29.74 to 22.60	No	ns	>0.9999
1 µM:Trypsin vs. 20 µM:Pepsin	-1.466	-27.64 to 24.71	No	ns	>0.9999
1 µM:Trypsin vs. 20 µM:Trypsin	-4.159	-30.33 to 22.01	No	ns	>0.9999
1 µM:Trypsin vs. 20 µM:Chymotrypsin	0.7101	-25.46 to 26.88	No	ns	>0.9999
1 µM:Trypsin vs. 20 µM:Subtilisin	17.35	-8.826 to 43.52	No	ns	0.6327
1 µM:Trypsin vs. 20 µM:Thermolysin	10.44	-15.73 to 36.61	No	ns	0.9960
1 µM:Trypsin vs. 20 µM:Proteinase K	83.94	57.77 to 110.1	Yes	****	<0.0001
1 µM:Chymotrypsin vs. 1 µM:Subtilisin	23.48	-2.693 to 49.65	No	ns	0.1326
1 µM:Chymotrypsin vs. 1 µM:Thermolysin	-11.42	-37.59 to 14.75	No	ns	0.9881
1 µM:Chymotrypsin vs. 1 µM:Proteinase K	-6.492	-32.67 to 19.68	No	ns	>0.9999
1 µM:Chymotrypsin vs. 5 µM:Pepsin	9.220	-16.95 to 35.39	No	ns	0.9993
1 µM:Chymotrypsin vs. 5 µM:Trypsin	-7.859	-34.03 to 18.31	No	ns	>0.9999

1 µM:Chymotrypsin vs. 5 µM:Chymotrypsin	-5.916	-32.09 to 20.26	No	ns	>0.9999
1 µM:Chymotrypsin vs. 5 µM:Subtilisin	12.35	-13.82 to 38.53	No	ns	0.9718
1 µM:Chymotrypsin vs. 5 µM:Thermolysin	-12.39	-38.56 to 13.79	No	ns	0.9711
1 µM:Chymotrypsin vs. 5 µM:Proteinase K	-16.15	-42.33 to 10.02	No	ns	0.7502
1 µM:Chymotrypsin vs. 10 µM:Pepsin	0.7304	-25.44 to 26.90	No	ns	>0.9999
1 µM:Chymotrypsin vs. 10 µM:Trypsin	-12.22	-38.40 to 13.95	No	ns	0.9747
1 µM:Chymotrypsin vs. 10 µM:Chymotrypsin	-10.96	-37.13 to 15.22	No	ns	0.9927
1 µM:Chymotrypsin vs. 10 µM:Subtilisin	5.705	-20.47 to 31.88	No	ns	>0.9999
1 µM:Chymotrypsin vs. 10 µM:Thermolysin	5.654	-20.52 to 31.83	No	ns	>0.9999
1 µM:Chymotrypsin vs. 10 µM:Proteinase K	-12.78	-38.95 to 13.40	No	ns	0.9605
1 µM:Chymotrypsin vs. 20 µM:Pepsin	-10.67	-36.85 to 15.50	No	ns	0.9947
1 µM:Chymotrypsin vs. 20 µM:Trypsin	-13.37	-39.54 to 12.81	No	ns	0.9396
1 µM:Chymotrypsin vs. 20 µM:Chymotrypsin	-8.496	-34.67 to 17.68	No	ns	0.9998
1 µM:Chymotrypsin vs. 20 µM:Subtilisin	8.141	-18.03 to 34.31	No	ns	0.9999
1 µM:Chymotrypsin vs. 20 µM:Thermolysin	1.235	-24.94 to 27.41	No	ns	>0.9999
1 µM:Chymotrypsin vs. 20 µM:Proteinase K	74.73	48.56 to 100.9	Yes	****	<0.0001
1 µM:Subtilisin vs. 1 µM:Thermolysin	-34.90	-61.07 to -8.726	Yes	**	0.0011
1 µM:Subtilisin vs. 1 µM:Proteinase K	-29.97	-56.15 to -3.799	Yes	*	0.0104
1 µM:Subtilisin vs. 5 µM:Pepsin	-14.26	-40.43 to 11.91	No	ns	0.8952
1 µM:Subtilisin vs. 5 µM:Trypsin	-31.34	-57.51 to -5.166	Yes	**	0.0056
1 µM:Subtilisin vs. 5 µM:Chymotrypsin	-29.40	-55.57 to -3.222	Yes	*	0.0133
1 µM:Subtilisin vs. 5 µM:Subtilisin	-11.13	-37.30 to 15.05	No	ns	0.9912
1 µM:Subtilisin vs. 5 µM:Thermolysin	-35.86	-62.04 to -9.692	Yes	***	0.0007
1 µM:Subtilisin vs. 5 µM:Proteinase K	-39.63	-65.81 to -13.46	Yes	***	0.0001

1 µM:Subtilisin vs. 10 µM:Pepsin	-22.75	-48.92 to 3.424	No	ns	0.1683
1 µM:Subtilisin vs. 10 µM:Trypsin	-35.70	-61.88 to -9.531	Yes	***	0.0007
1 µM:Subtilisin vs. 10 µM:Chymotrypsin	-34.44	-60.61 to -8.262	Yes	**	0.0013
1 µM:Subtilisin vs. 10 µM:Subtilisin	-17.77	-43.95 to 8.398	No	ns	0.5885
1 µM:Subtilisin vs. 10 µM:Thermolysin	-17.83	-44.00 to 8.348	No	ns	0.5833
1 µM:Subtilisin vs. 10 µM:Proteinase K	-36.26	-62.43 to -10.08	Yes	***	0.0006
1 µM:Subtilisin vs. 20 µM:Pepsin	-34.15	-60.33 to -7.980	Yes	**	0.0015
1 µM:Subtilisin vs. 20 µM:Trypsin	-36.85	-63.02 to -10.67	Yes	***	0.0004
1 µM:Subtilisin vs. 20 µM:Chymotrypsin	-31.98	-58.15 to -5.803	Yes	**	0.0042
1 µM:Subtilisin vs. 20 µM:Subtilisin	-15.34	-41.51 to 10.83	No	ns	0.8204
1 µM:Subtilisin vs. 20 µM:Thermolysin	-22.24	-48.42 to 3.928	No	ns	0.1971
1 µM:Subtilisin vs. 20 µM:Proteinase K	51.25	25.08 to 77.43	Yes	****	<0.0001
1 µM:Thermolysin vs. 1 µM:Proteinase K	4.928	-21.25 to 31.10	No	ns	>0.9999
1 µM:Thermolysin vs. 5 µM:Pepsin	20.64	-5.534 to 46.81	No	ns	0.3120
1 µM:Thermolysin vs. 5 µM:Trypsin	3.561	-22.61 to 29.73	No	ns	>0.9999
1 µM:Thermolysin vs. 5 µM:Chymotrypsin	5.504	-20.67 to 31.68	No	ns	>0.9999
1 µM:Thermolysin vs. 5 µM:Subtilisin	23.77	-2.399 to 49.95	No	ns	0.1200
1 µM:Thermolysin vs. 5 µM:Thermolysin	-0.9652	-27.14 to 25.21	No	ns	>0.9999
1 µM:Thermolysin vs. 5 µM:Proteinase K	-4.733	-30.91 to 21.44	No	ns	>0.9999
1 µM:Thermolysin vs. 10 µM:Pepsin	12.15	-14.02 to 38.32	No	ns	0.9763
1 µM:Thermolysin vs. 10 µM:Trypsin	-0.8050	-26.98 to 25.37	No	ns	>0.9999
1 µM:Thermolysin vs. 10 µM:Chymotrypsin	0.4642	-25.71 to 26.64	No	ns	>0.9999
1 µM:Thermolysin vs. 10 µM:Subtilisin	17.12	-9.048 to 43.30	No	ns	0.6555
1 µM:Thermolysin vs. 10 µM:Thermolysin	17.07	-9.099 to 43.25	No	ns	0.6606

1 µM:Thermolysin vs. 10 µM:Proteinase K	-1.358	-27.53 to 24.82	No	ns	>0.9999
1 µM:Thermolysin vs. 20 µM:Pepsin	0.7470	-25.43 to 26.92	No	ns	>0.9999
1 µM:Thermolysin vs. 20 µM:Trypsin	-1.946	-28.12 to 24.23	No	ns	>0.9999
1 µM:Thermolysin vs. 20 µM:Chymotrypsin	2.923	-23.25 to 29.10	No	ns	>0.9999
1 µM:Thermolysin vs. 20 µM:Subtilisin	19.56	-6.612 to 45.73	No	ns	0.4080
1 µM:Thermolysin vs. 20 µM:Thermolysin	12.65	-13.52 to 38.83	No	ns	0.9640
1 µM:Thermolysin vs. 20 µM:Proteinase K	86.15	59.98 to 112.3	Yes	****	<0.0001
1 µM:Proteinase K vs. 5 µM:Pepsin	15.71	-10.46 to 41.89	No	ns	0.7895
1 µM:Proteinase K vs. 5 µM:Trypsin	-1.367	-27.54 to 24.81	No	ns	>0.9999
1 µM:Proteinase K vs. 5 µM:Chymotrypsin	0.5766	-25.60 to 26.75	No	ns	>0.9999
1 µM:Proteinase K vs. 5 µM:Subtilisin	18.85	-7.326 to 45.02	No	ns	0.4781
1 µM:Proteinase K vs. 5 µM:Thermolysin	-5.893	-32.07 to 20.28	No	ns	>0.9999
1 µM:Proteinase K vs. 5 µM:Proteinase K	-9.661	-35.83 to 16.51	No	ns	0.9986
1 µM:Proteinase K vs. 10 µM:Pepsin	7.223	-18.95 to 33.40	No	ns	>0.9999
1 µM:Proteinase K vs. 10 µM:Trypsin	-5.732	-31.91 to 20.44	No	ns	>0.9999
1 µM:Proteinase K vs. 10 µM:Chymotrypsin	-4.463	-30.64 to 21.71	No	ns	>0.9999
1 µM:Proteinase K vs. 10 µM:Subtilisin	12.20	-13.98 to 38.37	No	ns	0.9753
1 µM:Proteinase K vs. 10 µM:Thermolysin	12.15	-14.03 to 38.32	No	ns	0.9764
1 µM:Proteinase K vs. 10 µM:Proteinase K	-6.285	-32.46 to 19.89	No	ns	>0.9999
1 µM:Proteinase K vs. 20 µM:Pepsin	-4.181	-30.35 to 21.99	No	ns	>0.9999
1 µM:Proteinase K vs. 20 µM:Trypsin	-6.874	-33.05 to 19.30	No	ns	>0.9999
1 µM:Proteinase K vs. 20 µM:Chymotrypsin	-2.004	-28.18 to 24.17	No	ns	>0.9999
1 µM:Proteinase K vs. 20 µM:Subtilisin	14.63	-11.54 to 40.81	No	ns	0.8719
1 µM:Proteinase K vs. 20 µM:Thermolysin	7.727	-18.45 to 33.90	No	ns	>0.9999

1 µM:Proteinase K vs. 20 µM:Proteinase K	81.23	55.05 to 107.4	Yes	****	<0.0001
5 µM:Pepsin vs. 5 µM:Trypsin	-17.08	-43.25 to 9.095	No	ns	0.6602
5 µM:Pepsin vs. 5 µM:Chymotrypsin	-15.14	-41.31 to 11.04	No	ns	0.8362
5 µM:Pepsin vs. 5 µM:Subtilisin	3.135	-23.04 to 29.31	No	ns	>0.9999
5 µM:Pepsin vs. 5 µM:Thermolysin	-21.60	-47.78 to 4.569	No	ns	0.2386
5 µM:Pepsin vs. 5 µM:Proteinase K	-25.37	-51.55 to 0.8007	No	ns	0.0678
5 µM:Pepsin vs. 10 µM:Pepsin	-8.489	-34.66 to 17.68	No	ns	0.9998
5 µM:Pepsin vs. 10 µM:Trypsin	-21.44	-47.62 to 4.729	No	ns	0.2499
5 µM:Pepsin vs. 10 µM:Chymotrypsin	-20.18	-46.35 to 5.998	No	ns	0.3516
5 µM:Pepsin vs. 10 µM:Subtilisin	-3.515	-29.69 to 22.66	No	ns	>0.9999
5 µM:Pepsin vs. 10 µM:Thermolysin	-3.565	-29.74 to 22.61	No	ns	>0.9999
5 µM:Pepsin vs. 10 µM:Proteinase K	-22.00	-48.17 to 4.176	No	ns	0.2125
5 µM:Pepsin vs. 20 µM:Pepsin	-19.89	-46.07 to 6.281	No	ns	0.3771
5 µM:Pepsin vs. 20 µM:Trypsin	-22.59	-48.76 to 3.588	No	ns	0.1773
5 µM:Pepsin vs. 20 µM:Chymotrypsin	-17.72	-43.89 to 8.457	No	ns	0.5947
5 µM:Pepsin vs. 20 µM:Subtilisin	-1.079	-27.25 to 25.09	No	ns	>0.9999
5 µM:Pepsin vs. 20 µM:Thermolysin	-7.984	-34.16 to 18.19	No	ns	>0.9999
5 µM:Pepsin vs. 20 µM:Proteinase K	65.51	39.34 to 91.69	Yes	****	<0.0001
5 µM:Trypsin vs. 5 µM:Chymotrypsin	1.943	-24.23 to 28.12	No	ns	>0.9999
5 µM:Trypsin vs. 5 µM:Subtilisin	20.21	-5.959 to 46.39	No	ns	0.3482
5 µM:Trypsin vs. 5 µM:Thermolysin	-4.526	-30.70 to 21.65	No	ns	>0.9999
5 µM:Trypsin vs. 5 µM:Proteinase K	-8.294	-34.47 to 17.88	No	ns	0.9999
5 µM:Trypsin vs. 10 µM:Pepsin	8.590	-17.58 to 34.76	No	ns	0.9997
5 µM:Trypsin vs. 10 µM:Trypsin	-4.366	-30.54 to 21.81	No	ns	>0.9999

5 µM:Trypsin vs. 10 µM:Chymotrypsin	-3.097	-29.27 to 23.08	No	ns	>0.9999
5 µM:Trypsin vs. 10 µM:Subtilisin	13.56	-12.61 to 39.74	No	ns	0.9312
5 µM:Trypsin vs. 10 µM:Thermolysin	13.51	-12.66 to 39.69	No	ns	0.9334
5 µM:Trypsin vs. 10 µM:Proteinase K	-4.918	-31.09 to 21.25	No	ns	>0.9999
5 µM:Trypsin vs. 20 µM:Pepsin	-2.814	-28.99 to 23.36	No	ns	>0.9999
5 µM:Trypsin vs. 20 µM:Trypsin	-5.507	-31.68 to 20.67	No	ns	>0.9999
5 µM:Trypsin vs. 20 µM:Chymotrypsin	-0.6374	-26.81 to 25.54	No	ns	>0.9999
5 µM:Trypsin vs. 20 µM:Subtilisin	16.00	-10.17 to 42.17	No	ns	0.7642
5 µM:Trypsin vs. 20 µM:Thermolysin	9.094	-17.08 to 35.27	No	ns	0.9994
5 µM:Trypsin vs. 20 µM:Proteinase K	82.59	56.42 to 108.8	Yes	****	<0.0001
5 µM:Chymotrypsin vs. 5 µM:Subtilisin	18.27	-7.903 to 44.44	No	ns	0.5371
5 µM:Chymotrypsin vs. 5 µM:Thermolysin	-6.469	-32.64 to 19.70	No	ns	>0.9999
5 µM:Chymotrypsin vs. 5 µM:Proteinase K	-10.24	-36.41 to 15.94	No	ns	0.9969
5 µM:Chymotrypsin vs. 10 µM:Pepsin	6.646	-19.53 to 32.82	No	ns	>0.9999
5 µM:Chymotrypsin vs. 10 µM:Trypsin	-6.309	-32.48 to 19.86	No	ns	>0.9999
5 µM:Chymotrypsin vs. 10 µM:Chymotrypsin	-5.040	-31.21 to 21.13	No	ns	>0.9999
5 µM:Chymotrypsin vs. 10 µM:Subtilisin	11.62	-14.55 to 37.79	No	ns	0.9854
5 µM:Chymotrypsin vs. 10 µM:Thermolysin	11.57	-14.60 to 37.74	No	ns	0.9861
5 µM:Chymotrypsin vs. 10 µM:Proteinase K	-6.862	-33.03 to 19.31	No	ns	>0.9999
5 µM:Chymotrypsin vs. 20 µM:Pepsin	-4.757	-30.93 to 21.42	No	ns	>0.9999
5 µM:Chymotrypsin vs. 20 µM:Trypsin	-7.450	-33.62 to 18.72	No	ns	>0.9999
5 µM:Chymotrypsin vs. 20 µM:Chymotrypsin	-2.581	-28.75 to 23.59	No	ns	>0.9999
5 µM:Chymotrypsin vs. 20 µM:Subtilisin	14.06	-12.12 to 40.23	No	ns	0.9067
5 µM:Chymotrypsin vs. 20 µM:Thermolysin	7.151	-19.02 to 33.32	No	ns	>0.9999

5 µM:Chymotrypsin vs. 20 µM:Proteinase K	80.65	54.48 to 106.8	Yes	****	<0.0001
5 µM:Subtilisin vs. 5 µM:Thermolysin	-24.74	-50.91 to 1.433	No	ns	0.0855
5 µM:Subtilisin vs. 5 µM:Proteinase K	-28.51	-54.68 to -2.335	Yes	*	0.0194
5 µM:Subtilisin vs. 10 µM:Pepsin	-11.62	-37.80 to 14.55	No	ns	0.9854
5 µM:Subtilisin vs. 10 µM:Trypsin	-24.58	-50.75 to 1.594	No	ns	0.0905
5 µM:Subtilisin vs. 10 µM:Chymotrypsin	-23.31	-49.48 to 2.863	No	ns	0.1403
5 µM:Subtilisin vs. 10 µM:Subtilisin	-6.650	-32.82 to 19.52	No	ns	>0.9999
5 µM:Subtilisin vs. 10 µM:Thermolysin	-6.700	-32.87 to 19.47	No	ns	>0.9999
5 µM:Subtilisin vs. 10 µM:Proteinase K	-25.13	-51.31 to 1.041	No	ns	0.0741
5 µM:Subtilisin vs. 20 µM:Pepsin	-23.03	-49.20 to 3.146	No	ns	0.1539
5 µM:Subtilisin vs. 20 µM:Trypsin	-25.72	-51.89 to 0.4525	No	ns	0.0595
5 µM:Subtilisin vs. 20 µM:Chymotrypsin	-20.85	-47.02 to 5.322	No	ns	0.2948
5 µM:Subtilisin vs. 20 µM:Subtilisin	-4.214	-30.39 to 21.96	No	ns	>0.9999
5 µM:Subtilisin vs. 20 µM:Thermolysin	-11.12	-37.29 to 15.05	No	ns	0.9913
5 µM:Subtilisin vs. 20 µM:Proteinase K	62.38	36.21 to 88.55	Yes	****	<0.0001
5 µM:Thermolysin vs. 5 µM:Proteinase K	-3.768	-29.94 to 22.41	No	ns	>0.9999
5 µM:Thermolysin vs. 10 µM:Pepsin	13.12	-13.06 to 39.29	No	ns	0.9493
5 µM:Thermolysin vs. 10 µM:Trypsin	0.1603	-26.01 to 26.33	No	ns	>0.9999
5 µM:Thermolysin vs. 10 µM:Chymotrypsin	1.429	-24.74 to 27.60	No	ns	>0.9999
5 µM:Thermolysin vs. 10 µM:Subtilisin	18.09	-8.083 to 44.26	No	ns	0.5558
5 µM:Thermolysin vs. 10 µM:Thermolysin	18.04	-8.134 to 44.21	No	ns	0.5611
5 µM:Thermolysin vs. 10 µM:Proteinase K	-0.3924	-26.57 to 25.78	No	ns	>0.9999
5 µM:Thermolysin vs. 20 µM:Pepsin	1.712	-24.46 to 27.89	No	ns	>0.9999
5 µM:Thermolysin vs. 20 µM:Trypsin	-0.9809	-27.15 to 25.19	No	ns	>0.9999

5 µM:Thermolysin vs. 20 µM:Chymotrypsin	3.889	-22.28 to 30.06	No	ns	>0.9999
5 µM:Thermolysin vs. 20 µM:Subtilisin	20.53	-5.647 to 46.70	No	ns	0.3214
5 µM:Thermolysin vs. 20 µM:Thermolysin	13.62	-12.55 to 39.79	No	ns	0.9286
5 µM:Thermolysin vs. 20 µM:Proteinase K	87.12	60.95 to 113.3	Yes	****	<0.0001
5 µM:Proteinase K vs. 10 µM:Pepsin	16.88	-9.290 to 43.06	No	ns	0.6798
5 µM:Proteinase K vs. 10 µM:Trypsin	3.928	-22.24 to 30.10	No	ns	>0.9999
5 µM:Proteinase K vs. 10 µM:Chymotrypsin	5.197	-20.98 to 31.37	No	ns	>0.9999
5 µM:Proteinase K vs. 10 µM:Subtilisin	21.86	-4.315 to 48.03	No	ns	0.2215
5 µM:Proteinase K vs. 10 µM:Thermolysin	21.81	-4.366 to 47.98	No	ns	0.2249
5 µM:Proteinase K vs. 10 µM:Proteinase K	3.376	-22.80 to 29.55	No	ns	>0.9999
5 µM:Proteinase K vs. 20 µM:Pepsin	5.480	-20.69 to 31.65	No	ns	>0.9999
5 µM:Proteinase K vs. 20 µM:Trypsin	2.787	-23.39 to 28.96	No	ns	>0.9999
5 µM:Proteinase K vs. 20 µM:Chymotrypsin	7.657	-18.52 to 33.83	No	ns	>0.9999
5 µM:Proteinase K vs. 20 µM:Subtilisin	24.29	-1.879 to 50.47	No	ns	0.1002
5 µM:Proteinase K vs. 20 µM:Thermolysin	17.39	-8.785 to 43.56	No	ns	0.6286
5 µM:Proteinase K vs. 20 µM:Proteinase K	90.89	64.71 to 117.1	Yes	****	<0.0001
10 µM:Pepsin vs. 10 µM:Trypsin	-12.96	-39.13 to 13.22	No	ns	0.9548
10 µM:Pepsin vs. 10 µM:Chymotrypsin	-11.69	-37.86 to 14.49	No	ns	0.9845
10 µM:Pepsin vs. 10 µM:Subtilisin	4.974	-21.20 to 31.15	No	ns	>0.9999
10 µM:Pepsin vs. 10 µM:Thermolysin	4.924	-21.25 to 31.10	No	ns	>0.9999
10 µM:Pepsin vs. 10 µM:Proteinase K	-13.51	-39.68 to 12.67	No	ns	0.9336
10 µM:Pepsin vs. 20 µM:Pepsin	-11.40	-37.58 to 14.77	No	ns	0.9882
10 µM:Pepsin vs. 20 µM:Trypsin	-14.10	-40.27 to 12.08	No	ns	0.9046
10 µM:Pepsin vs. 20 µM:Chymotrypsin	-9.227	-35.40 to 16.95	No	ns	0.9993

10 µM:Pepsin vs. 20 µM:Subtilisin	7.411	-18.76 to 33.58	No	ns	>0.9999
10 µM:Pepsin vs. 20 µM:Thermolysin	0.5047	-25.67 to 26.68	No	ns	>0.9999
10 µM:Pepsin vs. 20 µM:Proteinase K	74.00	47.83 to 100.2	Yes	****	<0.0001
10 µM:Trypsin vs. 10 µM:Chymotrypsin	1.269	-24.90 to 27.44	No	ns	>0.9999
10 µM:Trypsin vs. 10 µM:Subtilisin	17.93	-8.243 to 44.10	No	ns	0.5725
10 µM:Trypsin vs. 10 µM:Thermolysin	17.88	-8.294 to 44.05	No	ns	0.5777
10 µM:Trypsin vs. 10 µM:Proteinase K	-0.5526	-26.73 to 25.62	No	ns	>0.9999
10 µM:Trypsin vs. 20 µM:Pepsin	1.552	-24.62 to 27.73	No	ns	>0.9999
10 µM:Trypsin vs. 20 µM:Trypsin	-1.141	-27.31 to 25.03	No	ns	>0.9999
10 µM:Trypsin vs. 20 µM:Chymotrypsin	3.728	-22.44 to 29.90	No	ns	>0.9999
10 µM:Trypsin vs. 20 µM:Subtilisin	20.37	-5.807 to 46.54	No	ns	0.3350
10 µM:Trypsin vs. 20 µM:Thermolysin	13.46	-12.71 to 39.63	No	ns	0.9357
10 µM:Trypsin vs. 20 µM:Proteinase K	86.96	60.79 to 113.1	Yes	****	<0.0001
10 µM:Chymotrypsin vs. 10 µM:Subtilisin	16.66	-9.513 to 42.83	No	ns	0.7019
10 µM:Chymotrypsin vs. 10 µM:Thermolysin	16.61	-9.563 to 42.78	No	ns	0.7068
10 µM:Chymotrypsin vs. 10 µM:Proteinase K	-1.822	-27.99 to 24.35	No	ns	>0.9999
10 µM:Chymotrypsin vs. 20 µM:Pepsin	0.2828	-25.89 to 26.46	No	ns	>0.9999
10 µM:Chymotrypsin vs. 20 µM:Trypsin	-2.410	-28.58 to 23.76	No	ns	>0.9999
10 µM:Chymotrypsin vs. 20 µM:Chymotrypsin	2.459	-23.71 to 28.63	No	ns	>0.9999
10 µM:Chymotrypsin vs. 20 µM:Subtilisin	19.10	-7.077 to 45.27	No	ns	0.4531
10 µM:Chymotrypsin vs. 20 µM:Thermolysin	12.19	-13.98 to 38.36	No	ns	0.9755
10 µM:Chymotrypsin vs. 20 µM:Proteinase K	85.69	59.52 to 111.9	Yes	****	<0.0001
10 µM:Subtilisin vs. 10 µM:Thermolysin	-0.05066	-26.22 to 26.12	No	ns	>0.9999
10 µM:Subtilisin vs. 10 µM:Proteinase K	-18.48	-44.66 to 7.691	No	ns	0.5153

10 µM:Subtilisin vs. 20 µM:Pepsin	-16.38	-42.55 to 9.795	No	ns	0.7292
10 µM:Subtilisin vs. 20 µM:Trypsin	-19.07	-45.24 to 7.102	No	ns	0.4557
10 µM:Subtilisin vs. 20 µM:Chymotrypsin	-14.20	-40.37 to 11.97	No	ns	0.8986
10 µM:Subtilisin vs. 20 µM:Subtilisin	2.436	-23.74 to 28.61	No	ns	>0.9999
10 µM:Subtilisin vs. 20 µM:Thermolysin	-4.470	-30.64 to 21.70	No	ns	>0.9999
10 µM:Subtilisin vs. 20 µM:Proteinase K	69.03	42.86 to 95.20	Yes	****	<0.0001
10 µM:Thermolysin vs. 10 µM:Proteinase K	-18.43	-44.60 to 7.742	No	ns	0.5205
10 µM:Thermolysin vs. 20 µM:Pepsin	-16.33	-42.50 to 9.846	No	ns	0.7340
10 µM:Thermolysin vs. 20 µM:Trypsin	-19.02	-45.19 to 7.153	No	ns	0.4608
10 µM:Thermolysin vs. 20 µM:Chymotrypsin	-14.15	-40.32 to 12.02	No	ns	0.9015
10 µM:Thermolysin vs. 20 µM:Subtilisin	2.487	-23.69 to 28.66	No	ns	>0.9999
10 µM:Thermolysin vs. 20 µM:Thermolysin	-4.419	-30.59 to 21.75	No	ns	>0.9999
10 µM:Thermolysin vs. 20 µM:Proteinase K	69.08	42.91 to 95.25	Yes	****	<0.0001
10 µM:Proteinase K vs. 20 µM:Pepsin	2.105	-24.07 to 28.28	No	ns	>0.9999
10 µM:Proteinase K vs. 20 µM:Trypsin	-0.5885	-26.76 to 25.58	No	ns	>0.9999
10 µM:Proteinase K vs. 20 µM:Chymotrypsin	4.281	-21.89 to 30.45	No	ns	>0.9999
10 µM:Proteinase K vs. 20 µM:Subtilisin	20.92	-5.255 to 47.09	No	ns	0.2894
10 µM:Proteinase K vs. 20 µM:Thermolysin	14.01	-12.16 to 40.19	No	ns	0.9091
10 µM:Proteinase K vs. 20 µM:Proteinase K	87.51	61.34 to 113.7	Yes	****	<0.0001
20 µM:Pepsin vs. 20 µM:Trypsin	-2.693	-28.87 to 23.48	No	ns	>0.9999
20 µM:Pepsin vs. 20 µM:Chymotrypsin	2.176	-24.00 to 28.35	No	ns	>0.9999
20 µM:Pepsin vs. 20 µM:Subtilisin	18.81	-7.359 to 44.99	No	ns	0.4815
20 µM:Pepsin vs. 20 µM:Thermolysin	11.91	-14.27 to 38.08	No	ns	0.9809
20 µM:Pepsin vs. 20 µM:Proteinase K	85.41	59.23 to 111.6	Yes	****	<0.0001

20 µM:Trypsin vs. 20 µM:Chymotrypsin	4.869	-21.30 to 31.04	No	ns	>0.9999
20 µM:Trypsin vs. 20 µM:Subtilisin	21.51	-4.666 to 47.68	No	ns	0.2455
20 µM:Trypsin vs. 20 µM:Thermolysin	14.60	-11.57 to 40.77	No	ns	0.8741
20 µM:Trypsin vs. 20 µM:Proteinase K	88.10	61.93 to 114.3	Yes	****	<0.0001
20 µM:Chymotrypsin vs. 20 µM:Subtilisin	16.64	-9.536 to 42.81	No	ns	0.7041
20 µM:Chymotrypsin vs. 20 µM:Thermolysin	9.732	-16.44 to 35.90	No	ns	0.9984
20 µM:Chymotrypsin vs. 20 µM:Proteinase K	83.23	57.06 to 109.4	Yes	****	<0.0001
20 µM:Subtilisin vs. 20 µM:Thermolysin	-6.906	-33.08 to 19.27	No	ns	>0.9999
20 µM:Subtilisin vs. 20 µM:Proteinase K	66.59	40.42 to 92.77	Yes	****	<0.0001
20 µM:Thermolysin vs. 20 µM:Proteinase K	73.50	47.33 to 99.67	Yes	****	<0.0001

Table S9. The viability data for *A. platensis* hydrolysates tested on MACK1 cell line by resazurin assay

Sample (µM)	Control			AP - pepsin			AP - trypsin			AP - chymotrypsin			AP - subtilisin			AP - thermolysin			AP - proteinase K		
**0**	101.15	99.11	99.75	x																	
**1**	x			90.66	91.36	93.99	95.62	96.35	95.73	89.77	89.04	89.94	87.00	82.33	90.85	87.55	89.34	94.00	100.65	89.41	83.54
**5**				95.64	95.64	97.88	96.52	92.66	90.22	105.03	100.16	99.49	85.25	86.44	90.13	86.03	93.34	91.76	87.61	86.01	83.22
**10**				108.21	112.15	108.78	90.49	83.27	86.57	91.54	89.26	86.71	87.54	89.45	89.32	101.33	88.55	81.38	80.88	84.12	83.01
**20**				113.68	106.34	111.21	91.63	95.94	93.14	91.02	91.72	92.03	87.05	91.00	89.69	80.52	84.68	81.49	81.38	76.92	76.17

Table S10A. One-way ANOVA statistical analysis for *A. platensis* hydrolysates tested on MACK1 cell line by resazurin assay

**ANOVA summary**	
**F**	13.38
**P value**	<0.0001

**P value summary**	****				
**Significant diff. among means (P < 0.05)?**	Yes				
**R square**	0.8653				
**ANOVA table**	**SS**	**DF**	**MS**	**F (DFn, DFd)**	**P value**
**Treatment (between columns)**	4132	24	172.2	F (24, 50) = 13.38	P<0.0001
**Residual (within columns)**	643.4	50	12.87		
**Total**	4775	74			

Table S10B. Dunnett's multiple comparisons test for *A. platensis* hydrolysates tested on MACK1 cell line by resazurin assay

**Dunnett's multiple comparisons test**	**Mean Diff.**	**95.00% CI of diff.**	**Significant?**	**Summary**	**Adjusted P Value**
Control vs. Pepsin 1 µM	8.000	-1.009 to 17.01	No	ns	0.1123
Control vs. Trypsin 1 µM	4.102	-4.908 to 13.11	No	ns	0.8730
Control vs. Chymotrpysin 1 µM	10.42	1.406 to 19.42	Yes	*	0.0141
Control vs. Pepsin 5 µM	3.617	-5.392 to 12.63	No	ns	0.9471
Control vs. Trypsin 5 µM	6.865	-2.145 to 15.87	No	ns	0.2486
Control vs. Chymotrpysin 5 µM	-1.562	-10.57 to 7.448	No	ns	0.9993
Control vs. Pepsin 10 µM	-9.711	-18.72 to -0.7020	Yes	*	0.0271
Control vs. Trypsin 10 µM	13.23	4.216 to 22.23	Yes	***	0.0008
Control vs. Chymotrypsin 10 µM	10.83	1.823 to 19.84	Yes	**	0.0094
Control vs. Pepsin 20 µM	-10.41	-19.42 to -1.402	Yes	*	0.0142
Control vs. Trypsin 20 µM	6.428	-2.581 to 15.44	No	ns	0.3252
Control vs. Chymotrypsin 20 µM	8.413	-0.5962 to 17.42	No	ns	0.0815
Control vs. Subtilisin 1 µM	13.27	4.265 to 22.28	Yes	***	0.0008
Control vs. Thermoylsin 1 µM	9.702	0.6929 to 18.71	Yes	*	0.0273

Control vs. Proteinase K 1 µM	8.799	-0.2106 to 17.81	No	ns	0.0597
Control vs. Subtilisin 5 µM	12.73	3.717 to 21.74	Yes	**	0.0013
Control vs. Thermolysin 5 µM	9.623	0.6132 to 18.63	Yes	*	0.0293
Control vs. Proteinase K 5 µM	14.39	5.376 to 23.39	Yes	***	0.0002
Control vs. Subtilisin 10 µM	11.23	2.220 to 20.24	Yes	**	0.0064
Control vs. Thermolysin 10 µM	9.580	0.5708 to 18.59	Yes	*	0.0305
Control vs. Proteinase K 10 µM	17.33	8.320 to 26.34	Yes	****	<0.0001
Control vs. Subtilisin 20 µM	10.75	1.743 to 19.76	Yes	*	0.0102
Control vs. Thermolysin 20 µM	17.77	8.763 to 26.78	Yes	****	<0.0001
Control vs. Proteinase K 20 µM	21.84	12.83 to 30.85	Yes	****	<0.0001

Table S10C. Two-way ANOVA statistical analysis for *A. platensis* hydrolysates tested on MACK1 cell line by resazurin assay

**Two-way ANOVA**	Ordinary				
**Alpha**	0.05				
**Source of Variation**	**% of total variation**	**P value**	**P value summary**	**Significant?**	
**Interaction**	35.78	<0.0001	****	Yes	
**Row (concentration) Factor**	0.5856	0.5770	ns	No	
**Column (enzyme) Factor**	49.57	<0.0001	****	Yes	
**ANOVA table**	**SS**	**DF**	**MS**	**F (DFn, DFd)**	**P value**
**Interaction**	1631	15	108.7	F (15, 48) = 8.140	P<0.0001
**Row Factor**	26.69	3	8.897	F (3, 48) = 0.6661	P=0.5770
**Column Factor**	2259	5	451.9	F (5, 48) = 33.83	P<0.0001
**Residual**	641.2	48	13.36		

Table S10D. Tukey's multiple comparisons test for *A. platensis* hydrolysates tested on MACK1 cell line by resazurin assay

Tukey's multiple comparisons test	Mean Diff.	95.00% CI of diff.	Significant?	Summary	Adjusted P Value
1 µM:Pepsin vs. 1 µM:Trypsin	-3.897	-15.40 to 7.606	No	ns	0.9996
1 µM:Pepsin vs. 1 µM:Chymotrypsin	2.420	-9.082 to 13.92	No	ns	>0.9999
1 µM:Pepsin vs. 1 µM:Subtilisin	5.277	-6.226 to 16.78	No	ns	0.9791
1 µM:Pepsin vs. 1 µM:Thermolysin	1.707	-9.796 to 13.21	No	ns	>0.9999
1 µM:Pepsin vs. 1 µM:Proteinase K	0.8033	-10.70 to 12.31	No	ns	>0.9999
1 µM:Pepsin vs. 5 µM:Pepsin	-4.383	-15.89 to 7.119	No	ns	0.9978
1 µM:Pepsin vs. 5 µM:Trypsin	-1.130	-12.63 to 10.37	No	ns	>0.9999
1 µM:Pepsin vs. 5 µM:Chymotrypsin	-9.557	-21.06 to 1.946	No	ns	0.2290
1 µM:Pepsin vs. 5 µM:Subtilisin	4.730	-6.772 to 16.23	No	ns	0.9941
1 µM:Pepsin vs. 5 µM:Thermolysin	1.627	-9.876 to 13.13	No	ns	>0.9999
1 µM:Pepsin vs. 5 µM:Proteinase K	6.390	-5.112 to 17.89	No	ns	0.8780
1 µM:Pepsin vs. 10 µM:Pepsin	-17.71	-29.21 to -6.208	Yes	****	<0.0001
1 µM:Pepsin vs. 10 µM:Trypsin	5.227	-6.276 to 16.73	No	ns	0.9812
1 µM:Pepsin vs. 10 µM:Chymotrypsin	2.833	-8.669 to 14.34	No	ns	>0.9999
1 µM:Pepsin vs. 10 µM:Subtilisin	3.233	-8.269 to 14.74	No	ns	>0.9999
1 µM:Pepsin vs. 10 µM:Thermolysin	1.583	-9.919 to 13.09	No	ns	>0.9999
1 µM:Pepsin vs. 10 µM:Proteinase K	9.333	-2.169 to 20.84	No	ns	0.2650
1 µM:Pepsin vs. 20 µM:Pepsin	-18.41	-29.91 to -6.904	Yes	****	<0.0001
1 µM:Pepsin vs. 20 µM:Trypsin	-1.567	-13.07 to 9.936	No	ns	>0.9999
1 µM:Pepsin vs. 20 µM:Chymotrypsin	0.4133	-11.09 to 11.92	No	ns	>0.9999
1 µM:Pepsin vs. 20 µM:Subtilisin	2.757	-8.746 to 14.26	No	ns	>0.9999
1 µM:Pepsin vs. 20 µM:Thermolysin	9.773	-1.729 to 21.28	No	ns	0.1974

1 µM:Pepsin vs. 20 µM:Proteinase K	13.85	2.344 to 25.35	Yes	**	0.0052
1 µM:Trypsin vs. 1 µM:Chymotrypsin	6.317	-5.186 to 17.82	No	ns	0.8885
1 µM:Trypsin vs. 1 µM:Subtilisin	9.173	-2.329 to 20.68	No	ns	0.2930
1 µM:Trypsin vs. 1 µM:Thermolysin	5.603	-5.899 to 17.11	No	ns	0.9613
1 µM:Trypsin vs. 1 µM:Proteinase K	4.700	-6.802 to 16.20	No	ns	0.9946
1 µM:Trypsin vs. 5 µM:Pepsin	-0.4867	-11.99 to 11.02	No	ns	>0.9999
1 µM:Trypsin vs. 5 µM:Trypsin	2.767	-8.736 to 14.27	No	ns	>0.9999
1 µM:Trypsin vs. 5 µM:Chymotrypsin	-5.660	-17.16 to 5.842	No	ns	0.9573
1 µM:Trypsin vs. 5 µM:Subtilisin	8.627	-2.876 to 20.13	No	ns	0.4015
1 µM:Trypsin vs. 5 µM:Thermolysin	5.523	-5.979 to 17.03	No	ns	0.9664
1 µM:Trypsin vs. 5 µM:Proteinase K	10.29	-1.216 to 21.79	No	ns	0.1358
1 µM:Trypsin vs. 10 µM:Pepsin	-13.81	-25.32 to -2.311	Yes	**	0.0054
1 µM:Trypsin vs. 10 µM:Trypsin	9.123	-2.379 to 20.63	No	ns	0.3021
1 µM:Trypsin vs. 10 µM:Chymotrypsin	6.730	-4.772 to 18.23	No	ns	0.8224
1 µM:Trypsin vs. 10 µM:Subtilisin	7.130	-4.372 to 18.63	No	ns	0.7436
1 µM:Trypsin vs. 10 µM:Thermolysin	5.480	-6.022 to 16.98	No	ns	0.9690
1 µM:Trypsin vs. 10 µM:Proteinase K	13.23	1.728 to 24.73	Yes	**	0.0098
1 µM:Trypsin vs. 20 µM:Pepsin	-14.51	-26.01 to -3.008	Yes	**	0.0026
1 µM:Trypsin vs. 20 µM:Trypsin	2.330	-9.172 to 13.83	No	ns	>0.9999
1 µM:Trypsin vs. 20 µM:Chymotrypsin	4.310	-7.192 to 15.81	No	ns	0.9983
1 µM:Trypsin vs. 20 µM:Subtilisin	6.653	-4.849 to 18.16	No	ns	0.8359
1 µM:Trypsin vs. 20 µM:Thermolysin	13.67	2.168 to 25.17	Yes	**	0.0063
1 µM:Trypsin vs. 20 µM:Proteinase K	17.74	6.241 to 29.25	Yes	****	<0.0001
1 µM:Chymotrypsin vs. 1 µM:Subtilisin	2.857	-8.646 to 14.36	No	ns	>0.9999

1 µM:Chymotrypsin vs. 1 µM:Thermolysin	-0.7133	-12.22 to 10.79	No	ns	>0.9999
1 µM:Chymotrypsin vs. 1 µM:Proteinase K	-1.617	-13.12 to 9.886	No	ns	>0.9999
1 µM:Chymotrypsin vs. 5 µM:Pepsin	-6.803	-18.31 to 4.699	No	ns	0.8089
1 µM:Chymotrypsin vs. 5 µM:Trypsin	-3.550	-15.05 to 7.952	No	ns	>0.9999
1 µM:Chymotrypsin vs. 5 µM:Chymotrypsin	-11.98	-23.48 to -0.4744	Yes	*	0.0326
1 µM:Chymotrypsin vs. 5 µM:Subtilisin	2.310	-9.192 to 13.81	No	ns	>0.9999
1 µM:Chymotrypsin vs. 5 µM:Thermolysin	-0.7933	-12.30 to 10.71	No	ns	>0.9999
1 µM:Chymotrypsin vs. 5 µM:Proteinase K	3.970	-7.532 to 15.47	No	ns	0.9995
1 µM:Chymotrypsin vs. 10 µM:Pepsin	-20.13	-31.63 to -8.628	Yes	****	<0.0001
1 µM:Chymotrypsin vs. 10 µM:Trypsin	2.807	-8.696 to 14.31	No	ns	>0.9999
1 µM:Chymotrypsin vs. 10 µM:Chymotrypsin	0.4133	-11.09 to 11.92	No	ns	>0.9999
1 µM:Chymotrypsin vs. 10 µM:Subtilisin	0.8133	-10.69 to 12.32	No	ns	>0.9999
1 µM:Chymotrypsin vs. 10 µM:Thermolysin	-0.8367	-12.34 to 10.67	No	ns	>0.9999
1 µM:Chymotrypsin vs. 10 µM:Proteinase K	6.913	-4.589 to 18.42	No	ns	0.7879
1 µM:Chymotrypsin vs. 20 µM:Pepsin	-20.83	-32.33 to -9.324	Yes	****	<0.0001
1 µM:Chymotrypsin vs. 20 µM:Trypsin	-3.987	-15.49 to 7.516	No	ns	0.9994
1 µM:Chymotrypsin vs. 20 µM:Chymotrypsin	-2.007	-13.51 to 9.496	No	ns	>0.9999
1 µM:Chymotrypsin vs. 20 µM:Subtilisin	0.3367	-11.17 to 11.84	No	ns	>0.9999
1 µM:Chymotrypsin vs. 20 µM:Thermolysin	7.353	-4.149 to 18.86	No	ns	0.6948
1 µM:Chymotrypsin vs. 20 µM:Proteinase K	11.43	-0.07558 to 22.93	No	ns	0.0534
1 µM:Subtilisin vs. 1 µM:Thermolysin	-3.570	-15.07 to 7.932	No	ns	0.9999
1 µM:Subtilisin vs. 1 µM:Proteinase K	-4.473	-15.98 to 7.029	No	ns	0.9971
1 µM:Subtilisin vs. 5 µM:Pepsin	-9.660	-21.16 to 1.842	No	ns	0.2135
1 µM:Subtilisin vs. 5 µM:Trypsin	-6.407	-17.91 to 5.096	No	ns	0.8755

1 µM:Subtilisin vs. 5 µM:Chymotrypsin	-14.83	-26.34 to -3.331	Yes	**	0.0018
1 µM:Subtilisin vs. 5 µM:Subtilisin	-0.5467	-12.05 to 10.96	No	ns	>0.9999
1 µM:Subtilisin vs. 5 µM:Thermolysin	-3.650	-15.15 to 7.852	No	ns	0.9999
1 µM:Subtilisin vs. 5 µM:Proteinase K	1.113	-10.39 to 12.62	No	ns	>0.9999
1 µM:Subtilisin vs. 10 µM:Pepsin	-22.99	-34.49 to -11.48	Yes	****	<0.0001
1 µM:Subtilisin vs. 10 µM:Trypsin	-0.05000	-11.55 to 11.45	No	ns	>0.9999
1 µM:Subtilisin vs. 10 µM:Chymotrypsin	-2.443	-13.95 to 9.059	No	ns	>0.9999
1 µM:Subtilisin vs. 10 µM:Subtilisin	-2.043	-13.55 to 9.459	No	ns	>0.9999
1 µM:Subtilisin vs. 10 µM:Thermolysin	-3.693	-15.20 to 7.809	No	ns	0.9998
1 µM:Subtilisin vs. 10 µM:Proteinase K	4.057	-7.446 to 15.56	No	ns	0.9993
1 µM:Subtilisin vs. 20 µM:Pepsin	-23.68	-35.19 to -12.18	Yes	****	<0.0001
1 µM:Subtilisin vs. 20 µM:Trypsin	-6.843	-18.35 to 4.659	No	ns	0.8014
1 µM:Subtilisin vs. 20 µM:Chymotrypsin	-4.863	-16.37 to 6.639	No	ns	0.9917
1 µM:Subtilisin vs. 20 µM:Subtilisin	-2.520	-14.02 to 8.982	No	ns	>0.9999
1 µM:Subtilisin vs. 20 µM:Thermolysin	4.497	-7.006 to 16.00	No	ns	0.9969
1 µM:Subtilisin vs. 20 µM:Proteinase K	8.570	-2.932 to 20.07	No	ns	0.4137
1 µM:Thermolysin vs. 1 µM:Proteinase K	-0.9033	-12.41 to 10.60	No	ns	>0.9999
1 µM:Thermolysin vs. 5 µM:Pepsin	-6.090	-17.59 to 5.412	No	ns	0.9172
1 µM:Thermolysin vs. 5 µM:Trypsin	-2.837	-14.34 to 8.666	No	ns	>0.9999
1 µM:Thermolysin vs. 5 µM:Chymotrypsin	-11.26	-22.77 to 0.2389	No	ns	0.0615
1 µM:Thermolysin vs. 5 µM:Subtilisin	3.023	-8.479 to 14.53	No	ns	>0.9999
1 µM:Thermolysin vs. 5 µM:Thermolysin	-0.08000	-11.58 to 11.42	No	ns	>0.9999
1 µM:Thermolysin vs. 5 µM:Proteinase K	4.683	-6.819 to 16.19	No	ns	0.9948
1 µM:Thermolysin vs. 10 µM:Pepsin	-19.42	-30.92 to -7.914	Yes	****	<0.0001

1 µM:Thermolysin vs. 10 µM:Trypsin	3.520	-7.982 to 15.02	No	ns	>0.9999
1 µM:Thermolysin vs. 10 µM:Chymotrypsin	1.127	-10.38 to 12.63	No	ns	>0.9999
1 µM:Thermolysin vs. 10 µM:Subtilisin	1.527	-9.976 to 13.03	No	ns	>0.9999
1 µM:Thermolysin vs. 10 µM:Thermolysin	-0.1233	-11.63 to 11.38	No	ns	>0.9999
1 µM:Thermolysin vs. 10 µM:Proteinase K	7.627	-3.876 to 19.13	No	ns	0.6320
1 µM:Thermolysin vs. 20 µM:Pepsin	-20.11	-31.62 to -8.611	Yes	****	<0.0001
1 µM:Thermolysin vs. 20 µM:Trypsin	-3.273	-14.78 to 8.229	No	ns	>0.9999
1 µM:Thermolysin vs. 20 µM:Chymotrypsin	-1.293	-12.80 to 10.21	No	ns	>0.9999
1 µM:Thermolysin vs. 20 µM:Subtilisin	1.050	-10.45 to 12.55	No	ns	>0.9999
1 µM:Thermolysin vs. 20 µM:Thermolysin	8.067	-3.436 to 19.57	No	ns	0.5283
1 µM:Thermolysin vs. 20 µM:Proteinase K	12.14	0.6378 to 23.64	Yes	*	0.0281
1 µM:Proteinase K vs. 5 µM:Pepsin	-5.187	-16.69 to 6.316	No	ns	0.9827
1 µM:Proteinase K vs. 5 µM:Trypsin	-1.933	-13.44 to 9.569	No	ns	>0.9999
1 µM:Proteinase K vs. 5 µM:Chymotrypsin	-10.36	-21.86 to 1.142	No	ns	0.1284
1 µM:Proteinase K vs. 5 µM:Subtilisin	3.927	-7.576 to 15.43	No	ns	0.9995
1 µM:Proteinase K vs. 5 µM:Thermolysin	0.8233	-10.68 to 12.33	No	ns	>0.9999
1 µM:Proteinase K vs. 5 µM:Proteinase K	5.587	-5.916 to 17.09	No	ns	0.9624
1 µM:Proteinase K vs. 10 µM:Pepsin	-18.51	-30.02 to -7.011	Yes	****	<0.0001
1 µM:Proteinase K vs. 10 µM:Trypsin	4.423	-7.079 to 15.93	No	ns	0.9975
1 µM:Proteinase K vs. 10 µM:Chymotrypsin	2.030	-9.472 to 13.53	No	ns	>0.9999
1 µM:Proteinase K vs. 10 µM:Subtilisin	2.430	-9.072 to 13.93	No	ns	>0.9999
1 µM:Proteinase K vs. 10 µM:Thermolysin	0.7800	-10.72 to 12.28	No	ns	>0.9999
1 µM:Proteinase K vs. 10 µM:Proteinase K	8.530	-2.972 to 20.03	No	ns	0.4225
1 µM:Proteinase K vs. 20 µM:Pepsin	-19.21	-30.71 to -7.708	Yes	****	<0.0001

1 µM:Proteinase K vs. 20 µM:Trypsin	-2.370	-13.87 to 9.132	No	ns	>0.9999
1 µM:Proteinase K vs. 20 µM:Chymotrypsin	-0.3900	-11.89 to 11.11	No	ns	>0.9999
1 µM:Proteinase K vs. 20 µM:Subtilisin	1.953	-9.549 to 13.46	No	ns	>0.9999
1 µM:Proteinase K vs. 20 µM:Thermolysin	8.970	-2.532 to 20.47	No	ns	0.3311
1 µM:Proteinase K vs. 20 µM:Proteinase K	13.04	1.541 to 24.55	Yes	*	0.0118
5 µM:Pepsin vs. 5 µM:Trypsin	3.253	-8.249 to 14.76	No	ns	>0.9999
5 µM:Pepsin vs. 5 µM:Chymotrypsin	-5.173	-16.68 to 6.329	No	ns	0.9832
5 µM:Pepsin vs. 5 µM:Subtilisin	9.113	-2.389 to 20.62	No	ns	0.3039
5 µM:Pepsin vs. 5 µM:Thermolysin	6.010	-5.492 to 17.51	No	ns	0.9261
5 µM:Pepsin vs. 5 µM:Proteinase K	10.77	-0.7289 to 22.28	No	ns	0.0927
5 µM:Pepsin vs. 10 µM:Pepsin	-13.33	-24.83 to -1.824	Yes	**	0.0089
5 µM:Pepsin vs. 10 µM:Trypsin	9.610	-1.892 to 21.11	No	ns	0.2209
5 µM:Pepsin vs. 10 µM:Chymotrypsin	7.217	-4.286 to 18.72	No	ns	0.7250
5 µM:Pepsin vs. 10 µM:Subtilisin	7.617	-3.886 to 19.12	No	ns	0.6344
5 µM:Pepsin vs. 10 µM:Thermolysin	5.967	-5.536 to 17.47	No	ns	0.9306
5 µM:Pepsin vs. 10 µM:Proteinase K	13.72	2.214 to 25.22	Yes	**	0.0060
5 µM:Pepsin vs. 20 µM:Pepsin	-14.02	-25.53 to -2.521	Yes	**	0.0043
5 µM:Pepsin vs. 20 µM:Trypsin	2.817	-8.686 to 14.32	No	ns	>0.9999
5 µM:Pepsin vs. 20 µM:Chymotrypsin	4.797	-6.706 to 16.30	No	ns	0.9930
5 µM:Pepsin vs. 20 µM:Subtilisin	7.140	-4.362 to 18.64	No	ns	0.7415
5 µM:Pepsin vs. 20 µM:Thermolysin	14.16	2.654 to 25.66	Yes	**	0.0038
5 µM:Pepsin vs. 20 µM:Proteinase K	18.23	6.728 to 29.73	Yes	****	<0.0001
5 µM:Trypsin vs. 5 µM:Chymotrypsin	-8.427	-19.93 to 3.076	No	ns	0.4454
5 µM:Trypsin vs. 5 µM:Subtilisin	5.860	-5.642 to 17.36	No	ns	0.9409

5 µM:Trypsin vs. 5 µM:Thermolysin	2.757	-8.746 to 14.26	No	ns	>0.9999
5 µM:Trypsin vs. 5 µM:Proteinase K	7.520	-3.982 to 19.02	No	ns	0.6568
5 µM:Trypsin vs. 10 µM:Pepsin	-16.58	-28.08 to -5.078	Yes	***	0.0003
5 µM:Trypsin vs. 10 µM:Trypsin	6.357	-5.146 to 17.86	No	ns	0.8828
5 µM:Trypsin vs. 10 µM:Chymotrypsin	3.963	-7.539 to 15.47	No	ns	0.9995
5 µM:Trypsin vs. 10 µM:Subtilisin	4.363	-7.139 to 15.87	No	ns	0.9979
5 µM:Trypsin vs. 10 µM:Thermolysin	2.713	-8.789 to 14.22	No	ns	>0.9999
5 µM:Trypsin vs. 10 µM:Proteinase K	10.46	-1.039 to 21.97	No	ns	0.1186
5 µM:Trypsin vs. 20 µM:Pepsin	-17.28	-28.78 to -5.774	Yes	***	0.0001
5 µM:Trypsin vs. 20 µM:Trypsin	-0.4367	-11.94 to 11.07	No	ns	>0.9999
5 µM:Trypsin vs. 20 µM:Chymotrypsin	1.543	-9.959 to 13.05	No	ns	>0.9999
5 µM:Trypsin vs. 20 µM:Subtilisin	3.887	-7.616 to 15.39	No	ns	0.9996
5 µM:Trypsin vs. 20 µM:Thermolysin	10.90	-0.5989 to 22.41	No	ns	0.0833
5 µM:Trypsin vs. 20 µM:Proteinase K	14.98	3.474 to 26.48	Yes	**	0.0016
5 µM:Chymotrypsin vs. 5 µM:Subtilisin	14.29	2.784 to 25.79	Yes	**	0.0033
5 µM:Chymotrypsin vs. 5 µM:Thermolysin	11.18	-0.3189 to 22.69	No	ns	0.0659
5 µM:Chymotrypsin vs. 5 µM:Proteinase K	15.95	4.444 to 27.45	Yes	***	0.0005
5 µM:Chymotrypsin vs. 10 µM:Pepsin	-8.153	-19.66 to 3.349	No	ns	0.5080
5 µM:Chymotrypsin vs. 10 µM:Trypsin	14.78	3.281 to 26.29	Yes	**	0.0019
5 µM:Chymotrypsin vs. 10 µM:Chymotrypsin	12.39	0.8878 to 23.89	Yes	*	0.0222
5 µM:Chymotrypsin vs. 10 µM:Subtilisin	12.79	1.288 to 24.29	Yes	*	0.0151
5 µM:Chymotrypsin vs. 10 µM:Thermolysin	11.14	-0.3622 to 22.64	No	ns	0.0684
5 µM:Chymotrypsin vs. 10 µM:Proteinase K	18.89	7.388 to 30.39	Yes	****	<0.0001
5 µM:Chymotrypsin vs. 20 µM:Pepsin	-8.850	-20.35 to 2.652	No	ns	0.3549

5 µM:Chymotrypsin vs. 20 µM:Trypsin	7.990	-3.512 to 19.49	No	ns	0.5464
5 µM:Chymotrypsin vs. 20 µM:Chymotrypsin	9.970	-1.532 to 21.47	No	ns	0.1717
5 µM:Chymotrypsin vs. 20 µM:Subtilisin	12.31	0.8111 to 23.82	Yes	*	0.0239
5 µM:Chymotrypsin vs. 20 µM:Thermolysin	19.33	7.828 to 30.83	Yes	****	<0.0001
5 µM:Chymotrypsin vs. 20 µM:Proteinase K	23.40	11.90 to 34.91	Yes	****	<0.0001
5 µM:Subtilisin vs. 5 µM:Thermolysin	-3.103	-14.61 to 8.399	No	ns	>0.9999
5 µM:Subtilisin vs. 5 µM:Proteinase K	1.660	-9.842 to 13.16	No	ns	>0.9999
5 µM:Subtilisin vs. 10 µM:Pepsin	-22.44	-33.94 to -10.94	Yes	****	<0.0001
5 µM:Subtilisin vs. 10 µM:Trypsin	0.4967	-11.01 to 12.00	No	ns	>0.9999
5 µM:Subtilisin vs. 10 µM:Chymotrypsin	-1.897	-13.40 to 9.606	No	ns	>0.9999
5 µM:Subtilisin vs. 10 µM:Subtilisin	-1.497	-13.00 to 10.01	No	ns	>0.9999
5 µM:Subtilisin vs. 10 µM:Thermolysin	-3.147	-14.65 to 8.356	No	ns	>0.9999
5 µM:Subtilisin vs. 10 µM:Proteinase K	4.603	-6.899 to 16.11	No	ns	0.9958
5 µM:Subtilisin vs. 20 µM:Pepsin	-23.14	-34.64 to -11.63	Yes	****	<0.0001
5 µM:Subtilisin vs. 20 µM:Trypsin	-6.297	-17.80 to 5.206	No	ns	0.8912
5 µM:Subtilisin vs. 20 µM:Chymotrypsin	-4.317	-15.82 to 7.186	No	ns	0.9982
5 µM:Subtilisin vs. 20 µM:Subtilisin	-1.973	-13.48 to 9.529	No	ns	>0.9999
5 µM:Subtilisin vs. 20 µM:Thermolysin	5.043	-6.459 to 16.55	No	ns	0.9874
5 µM:Subtilisin vs. 20 µM:Proteinase K	9.117	-2.386 to 20.62	No	ns	0.3033
5 µM:Thermolysin vs. 5 µM:Proteinase K	4.763	-6.739 to 16.27	No	ns	0.9936
5 µM:Thermolysin vs. 10 µM:Pepsin	-19.34	-30.84 to -7.834	Yes	****	<0.0001
5 µM:Thermolysin vs. 10 µM:Trypsin	3.600	-7.902 to 15.10	No	ns	0.9999
5 µM:Thermolysin vs. 10 µM:Chymotrypsin	1.207	-10.30 to 12.71	No	ns	>0.9999
5 µM:Thermolysin vs. 10 µM:Subtilisin	1.607	-9.896 to 13.11	No	ns	>0.9999

5 µM:Thermolysin vs. 10 µM:Thermolysin	-0.04333	-11.55 to 11.46	No	ns	>0.9999
5 µM:Thermolysin vs. 10 µM:Proteinase K	7.707	-3.796 to 19.21	No	ns	0.6133
5 µM:Thermolysin vs. 20 µM:Pepsin	-20.03	-31.54 to -8.531	Yes	****	<0.0001
5 µM:Thermolysin vs. 20 µM:Trypsin	-3.193	-14.70 to 8.309	No	ns	>0.9999
5 µM:Thermolysin vs. 20 µM:Chymotrypsin	-1.213	-12.72 to 10.29	No	ns	>0.9999
5 µM:Thermolysin vs. 20 µM:Subtilisin	1.130	-10.37 to 12.63	No	ns	>0.9999
5 µM:Thermolysin vs. 20 µM:Thermolysin	8.147	-3.356 to 19.65	No	ns	0.5096
5 µM:Thermolysin vs. 20 µM:Proteinase K	12.22	0.7178 to 23.72	Yes	*	0.0260
5 µM:Proteinase K vs. 10 µM:Pepsin	-24.10	-35.60 to -12.60	Yes	****	<0.0001
5 µM:Proteinase K vs. 10 µM:Trypsin	-1.163	-12.67 to 10.34	No	ns	>0.9999
5 µM:Proteinase K vs. 10 µM:Chymotrypsin	-3.557	-15.06 to 7.946	No	ns	>0.9999
5 µM:Proteinase K vs. 10 µM:Subtilisin	-3.157	-14.66 to 8.346	No	ns	>0.9999
5 µM:Proteinase K vs. 10 µM:Thermolysin	-4.807	-16.31 to 6.696	No	ns	0.9928
5 µM:Proteinase K vs. 10 µM:Proteinase K	2.943	-8.559 to 14.45	No	ns	>0.9999
5 µM:Proteinase K vs. 20 µM:Pepsin	-24.80	-36.30 to -13.29	Yes	****	<0.0001
5 µM:Proteinase K vs. 20 µM:Trypsin	-7.957	-19.46 to 3.546	No	ns	0.5542
5 µM:Proteinase K vs. 20 µM:Chymotrypsin	-5.977	-17.48 to 5.526	No	ns	0.9296
5 µM:Proteinase K vs. 20 µM:Subtilisin	-3.633	-15.14 to 7.869	No	ns	0.9999
5 µM:Proteinase K vs. 20 µM:Thermolysin	3.383	-8.119 to 14.89	No	ns	>0.9999
5 µM:Proteinase K vs. 20 µM:Proteinase K	7.457	-4.046 to 18.96	No	ns	0.6714
10 µM:Pepsin vs. 10 µM:Trypsin	22.94	11.43 to 34.44	Yes	****	<0.0001
10 µM:Pepsin vs. 10 µM:Chymotrypsin	20.54	9.041 to 32.05	Yes	****	<0.0001
10 µM:Pepsin vs. 10 µM:Subtilisin	20.94	9.441 to 32.45	Yes	****	<0.0001
10 µM:Pepsin vs. 10 µM:Thermolysin	19.29	7.791 to 30.80	Yes	****	<0.0001

10 µM:Pepsin vs. 10 µM:Proteinase K	27.04	15.54 to 38.55	Yes	****	<0.0001
10 µM:Pepsin vs. 20 µM:Pepsin	-0.6967	-12.20 to 10.81	No	ns	>0.9999
10 µM:Pepsin vs. 20 µM:Trypsin	16.14	4.641 to 27.65	Yes	***	0.0004
10 µM:Pepsin vs. 20 µM:Chymotrypsin	18.12	6.621 to 29.63	Yes	****	<0.0001
10 µM:Pepsin vs. 20 µM:Subtilisin	20.47	8.964 to 31.97	Yes	****	<0.0001
10 µM:Pepsin vs. 20 µM:Thermolysin	27.48	15.98 to 38.99	Yes	****	<0.0001
10 µM:Pepsin vs. 20 µM:Proteinase K	31.56	20.05 to 43.06	Yes	****	<0.0001
10 µM:Trypsin vs. 10 µM:Chymotrypsin	-2.393	-13.90 to 9.109	No	ns	>0.9999
10 µM:Trypsin vs. 10 µM:Subtilisin	-1.993	-13.50 to 9.509	No	ns	>0.9999
10 µM:Trypsin vs. 10 µM:Thermolysin	-3.643	-15.15 to 7.859	No	ns	0.9999
10 µM:Trypsin vs. 10 µM:Proteinase K	4.107	-7.396 to 15.61	No	ns	0.9991
10 µM:Trypsin vs. 20 µM:Pepsin	-23.63	-35.14 to -12.13	Yes	****	<0.0001
10 µM:Trypsin vs. 20 µM:Trypsin	-6.793	-18.30 to 4.709	No	ns	0.8108
10 µM:Trypsin vs. 20 µM:Chymotrypsin	-4.813	-16.32 to 6.689	No	ns	0.9927
10 µM:Trypsin vs. 20 µM:Subtilisin	-2.470	-13.97 to 9.032	No	ns	>0.9999
10 µM:Trypsin vs. 20 µM:Thermolysin	4.547	-6.956 to 16.05	No	ns	0.9964
10 µM:Trypsin vs. 20 µM:Proteinase K	8.620	-2.882 to 20.12	No	ns	0.4029
10 µM:Chymotrypsin vs. 10 µM:Subtilisin	0.4000	-11.10 to 11.90	No	ns	>0.9999
10 µM:Chymotrypsin vs. 10 µM:Thermolysin	-1.250	-12.75 to 10.25	No	ns	>0.9999
10 µM:Chymotrypsin vs. 10 µM:Proteinase K	6.500	-5.002 to 18.00	No	ns	0.8613
10 µM:Chymotrypsin vs. 20 µM:Pepsin	-21.24	-32.74 to -9.738	Yes	****	<0.0001
10 µM:Chymotrypsin vs. 20 µM:Trypsin	-4.400	-15.90 to 7.102	No	ns	0.9977
10 µM:Chymotrypsin vs. 20 µM:Chymotrypsin	-2.420	-13.92 to 9.082	No	ns	>0.9999
10 µM:Chymotrypsin vs. 20 µM:Subtilisin	-0.07667	-11.58 to 11.43	No	ns	>0.9999

10 µM:Chymotrypsin vs. 20 µM:Thermolysin	6.940	-4.562 to 18.44	No	ns	0.7826
10 µM:Chymotrypsin vs. 20 µM:Proteinase K	11.01	-0.4889 to 22.52	No	ns	0.0761
10 µM:Subtilisin vs. 10 µM:Thermolysin	-1.650	-13.15 to 9.852	No	ns	>0.9999
10 µM:Subtilisin vs. 10 µM:Proteinase K	6.100	-5.402 to 17.60	No	ns	0.9160
10 µM:Subtilisin vs. 20 µM:Pepsin	-21.64	-33.14 to -10.14	Yes	****	<0.0001
10 µM:Subtilisin vs. 20 µM:Trypsin	-4.800	-16.30 to 6.702	No	ns	0.9930
10 µM:Subtilisin vs. 20 µM:Chymotrypsin	-2.820	-14.32 to 8.682	No	ns	>0.9999
10 µM:Subtilisin vs. 20 µM:Subtilisin	-0.4767	-11.98 to 11.03	No	ns	>0.9999
10 µM:Subtilisin vs. 20 µM:Thermolysin	6.540	-4.962 to 18.04	No	ns	0.8549
10 µM:Subtilisin vs. 20 µM:Proteinase K	10.61	-0.8889 to 22.12	No	ns	0.1054
10 µM:Thermolysin vs. 10 µM:Proteinase K	7.750	-3.752 to 19.25	No	ns	0.6031
10 µM:Thermolysin vs. 20 µM:Pepsin	-19.99	-31.49 to -8.488	Yes	****	<0.0001
10 µM:Thermolysin vs. 20 µM:Trypsin	-3.150	-14.65 to 8.352	No	ns	>0.9999
10 µM:Thermolysin vs. 20 µM:Chymotrypsin	-1.170	-12.67 to 10.33	No	ns	>0.9999
10 µM:Thermolysin vs. 20 µM:Subtilisin	1.173	-10.33 to 12.68	No	ns	>0.9999
10 µM:Thermolysin vs. 20 µM:Thermolysin	8.190	-3.312 to 19.69	No	ns	0.4995
10 µM:Thermolysin vs. 20 µM:Proteinase K	12.26	0.7611 to 23.77	Yes	*	0.0250
10 µM:Proteinase K vs. 20 µM:Pepsin	-27.74	-39.24 to -16.24	Yes	****	<0.0001
10 µM:Proteinase K vs. 20 µM:Trypsin	-10.90	-22.40 to 0.6022	No	ns	0.0836
10 µM:Proteinase K vs. 20 µM:Chymotrypsin	-8.920	-20.42 to 2.582	No	ns	0.3409
10 µM:Proteinase K vs. 20 µM:Subtilisin	-6.577	-18.08 to 4.926	No	ns	0.8489
10 µM:Proteinase K vs. 20 µM:Thermolysin	0.4400	-11.06 to 11.94	No	ns	>0.9999
10 µM:Proteinase K vs. 20 µM:Proteinase K	4.513	-6.989 to 16.02	No	ns	0.9968
20 µM:Pepsin vs. 20 µM:Trypsin	16.84	5.338 to 28.34	Yes	***	0.0002

20 µM:Pepsin vs. 20 µM:Chymotrypsin	18.82	7.318 to 30.32	Yes	****	<0.0001
20 µM:Pepsin vs. 20 µM:Subtilisin	21.16	9.661 to 32.67	Yes	****	<0.0001
20 µM:Pepsin vs. 20 µM:Thermolysin	28.18	16.68 to 39.68	Yes	****	<0.0001
20 µM:Pepsin vs. 20 µM:Proteinase K	32.25	20.75 to 43.76	Yes	****	<0.0001
20 µM:Trypsin vs. 20 µM:Chymotrypsin	1.980	-9.522 to 13.48	No	ns	>0.9999
20 µM:Trypsin vs. 20 µM:Subtilisin	4.323	-7.179 to 15.83	No	ns	0.9982
20 µM:Trypsin vs. 20 µM:Thermolysin	11.34	-0.1622 to 22.84	No	ns	0.0576
20 µM:Trypsin vs. 20 µM:Proteinase K	15.41	3.911 to 26.92	Yes	***	0.0010
20 µM:Chymotrypsin vs. 20 µM:Subtilisin	2.343	-9.159 to 13.85	No	ns	>0.9999
20 µM:Chymotrypsin vs. 20 µM:Thermolysin	9.360	-2.142 to 20.86	No	ns	0.2605
20 µM:Chymotrypsin vs. 20 µM:Proteinase K	13.43	1.931 to 24.94	Yes	**	0.0080
20 µM:Subtilisin vs. 20 µM:Thermolysin	7.017	-4.486 to 18.52	No	ns	0.7672
20 µM:Subtilisin vs. 20 µM:Proteinase K	11.09	-0.4122 to 22.59	No	ns	0.0713
20 µM:Thermolysin vs. 20 µM:Proteinase K	4.073	-7.429 to 15.58	No	ns	0.9992

Table S11. The viability data for *P. haitanensis* hydrolysates tested on MACK1 cell line by resazurin assay

Sample (µM)	Control			PH - pepsin			PH - trypsin			PH - chymotrypsin			PH - subtilisin			PH - thermolysin			PH - proteinase K		
**0**	103.22	97.88	98.90	x																	
**1**	x			93.31	98.01	93.60	62.90	66.09	72.73	71.37	73.65	71.23	102.92	106.91	102.45	92.27	95.64	93.95	97.36	91.95	95.64
**5**				72.49	74.63	66.76	72.09	71.68	66.25	66.04	66.07	60.52	98.77	95.28	92.97	82.34	85.42	87.20	98.19	96.72	94.65
**10**				58.63	67.51	63.07	76.83	74.74	80.84	69.21	65.39	62.17	79.61	78.76	82.73	91.81	95.20	98.21	107.27	116.28	109.20
**20**				66.89	70.95	71.79	93.17	92.16	94.52	85.46	87.70	76.44	74.33	76.92	74.76	109.35	105.78	112.62	83.97	78.49	76.24

Table S12A. One-way statistical analysis for *P. haitanensis* hydrolysates tested on MACK1 cell line by resazurin assay

ANOVA summary					
F	60.67				
P value	<0.0001				
P value summary	****				
Significant diff. among means (P < 0.05)?	Yes				
R square	0.9668				
**ANOVA table**	**SS**	**DF**	**MS**	**F (DFn, DFd)**	**P value**
Treatment (between columns)	15472	24	644.7	F (24, 50) = 60.67	P<0.0001
Residual (within columns)	531.3	50	10.63		
Total	16004	74			

Table S12B. Dunnett's multiple comparisons test for *P. haitanensis* hydrolysates tested on MACK1 cell line by resazurin assay

Dunnett's multiple comparisons test	Mean Diff.	95.00% CI of diff.	Significant?	Summary	Adjusted P Value
Control vs. Pepsin 1 µM	5.025	-3.162 to 13.21	No	ns	0.5261
Control vs. Trypsin 1 µM	32.76	24.57 to 40.95	Yes	****	<0.0001
Control vs. Chymotrpysin 1 µM	27.92	19.73 to 36.11	Yes	****	<0.0001
Control vs. Pepsin 5 µM	28.70	20.52 to 36.89	Yes	****	<0.0001
Control vs. Trypsin 5 µM	29.99	21.81 to 38.18	Yes	****	<0.0001
Control vs. Chymotrpysin 5 µM	35.79	27.60 to 43.98	Yes	****	<0.0001
Control vs. Pepsin 10 µM	36.93	28.74 to 45.11	Yes	****	<0.0001
Control vs. Trypsin 10 µM	22.53	14.34 to 30.72	Yes	****	<0.0001
Control vs. Chymotrypsin 10 µM	34.41	26.22 to 42.60	Yes	****	<0.0001
Control vs. Pepsin 20 µM	30.12	21.94 to 38.31	Yes	****	<0.0001
Control vs. Trypsin 20 µM	6.717	-1.470 to 14.90	No	ns	0.1746

Control vs. Chymotrypsin 20 µM	16.80	8.615 to 24.99	Yes	****	<0.0001
Control vs. Subtilisin 1 µM	-4.093	-12.28 to 4.094	No	ns	0.7868
Control vs. Thermoylsin 1 µM	6.047	-2.139 to 14.23	No	ns	0.2835
Control vs. Proteinase K 1 µM	5.017	-3.170 to 13.20	No	ns	0.5283
Control vs. Subtilisin 5 µM	4.329	-3.858 to 12.52	No	ns	0.7231
Control vs. Thermolysin 5 µM	15.01	6.827 to 23.20	Yes	****	<0.0001
Control vs. Proteinase K 5 µM	3.480	-4.707 to 11.67	No	ns	0.9191
Control vs. Subtilisin 10 µM	19.64	11.45 to 27.82	Yes	****	<0.0001
Control vs. Thermolysin 10 µM	4.929	-3.257 to 13.12	No	ns	0.5527
Control vs. Proteinase K 10 µM	-10.92	-19.10 to -2.731	Yes	**	0.0029
Control vs. Subtilisin 20 µM	24.66	16.47 to 32.85	Yes	****	<0.0001
Control vs. Thermolysin 20 µM	-9.253	-17.44 to -1.066	Yes	*	0.0176
Control vs. Proteinase K 20 µM	20.43	12.25 to 28.62	Yes	****	<0.0001

Table S12C. Two-way ANOVA statistical analysis for *P. haitanensis* hydrolysates tested on MACK1 cell line by resazurin assay

**Two-way ANOVA**	Ordinary				
**Alpha**	0.05				
**Source of Variation**	**% of total variation**	**P value**	**P value summary**	**Significant?**	
**Interaction**	46.24	<0.0001	****	Yes	
**Row (concentration) Factor**	3.842	<0.0001	****	Yes	
**Column (enzyme) Factor**	46.54	<0.0001	****	Yes	
**ANOVA table**	**SS**	**DF**	**MS**	**F (DFn, DFd)**	**P value**
**Interaction**	7046	15	469.7	F (15, 48) = 43.75	P<0.0001
**Row Factor**	585.5	3	195.2	F (3, 48) = 18.18	P<0.0001

**Column Factor**	7092	5	1418	F (5, 48) = 132.1 P<0.0001
**Residual**	515.3	48	10.74	

Table S12D. Tukey's multiple comparisons test for *P. haitanensis* hydrolysates tested on MACK1 cell line by resazurin assay

**Tukey's multiple comparisons test**	**Mean Diff.**	**95.00% CI of diff.**	**Significant?**	**Summary**	**Adjusted P Value**
1 µM:Pepsin vs. 1 µM:Trypsin	27.73	17.42 to 38.05	Yes	****	<0.0001
1 µM:Pepsin vs. 1 µM:Chymotrypsin	22.89	12.58 to 33.20	Yes	****	<0.0001
1 µM:Pepsin vs. 1 µM:Subtilisin	-9.120	-19.43 to 1.192	No	ns	0.1480
1 µM:Pepsin vs. 1 µM:Thermolysin	1.020	-9.292 to 11.33	No	ns	>0.9999
1 µM:Pepsin vs. 1 µM:Proteinase K	-0.01000	-10.32 to 10.30	No	ns	>0.9999
1 µM:Pepsin vs. 5 µM:Pepsin	23.68	13.37 to 33.99	Yes	****	<0.0001
1 µM:Pepsin vs. 5 µM:Trypsin	24.97	14.65 to 35.28	Yes	****	<0.0001
1 µM:Pepsin vs. 5 µM:Chymotrypsin	30.76	20.45 to 41.08	Yes	****	<0.0001
1 µM:Pepsin vs. 5 µM:Subtilisin	-0.7000	-11.01 to 9.612	No	ns	>0.9999
1 µM:Pepsin vs. 5 µM:Thermolysin	9.987	-0.3251 to 20.30	No	ns	0.0684
1 µM:Pepsin vs. 5 µM:Proteinase K	-1.547	-11.86 to 8.765	No	ns	>0.9999
1 µM:Pepsin vs. 10 µM:Pepsin	31.90	21.59 to 42.22	Yes	****	<0.0001
1 µM:Pepsin vs. 10 µM:Trypsin	17.50	7.192 to 27.82	Yes	****	<0.0001
1 µM:Pepsin vs. 10 µM:Chymotrypsin	29.38	19.07 to 39.70	Yes	****	<0.0001
1 µM:Pepsin vs. 10 µM:Subtilisin	14.61	4.295 to 24.92	Yes	***	0.0004
1 µM:Pepsin vs. 10 µM:Thermolysin	-0.1000	-10.41 to 10.21	No	ns	>0.9999
1 µM:Pepsin vs. 10 µM:Proteinase K	-15.94	-26.26 to -5.632	Yes	****	<0.0001
1 µM:Pepsin vs. 20 µM:Pepsin	25.10	14.78 to 35.41	Yes	****	<0.0001
1 µM:Pepsin vs. 20 µM:Trypsin	1.690	-8.622 to 12.00	No	ns	>0.9999

1 µM:Pepsin vs. 20 µM:Chymotrypsin	11.77	1.462 to 22.09	Yes	*	0.0108
1 µM:Pepsin vs. 20 µM:Subtilisin	19.64	9.325 to 29.95	Yes	****	<0.0001
1 µM:Pepsin vs. 20 µM:Thermolysin	-14.28	-24.59 to -3.965	Yes	***	0.0006
1 µM:Pepsin vs. 20 µM:Proteinase K	15.41	5.096 to 25.72	Yes	***	0.0001
1 µM:Trypsin vs. 1 µM:Chymotrypsin	-4.843	-15.16 to 5.468	No	ns	0.9732
1 µM:Trypsin vs. 1 µM:Subtilisin	-36.85	-47.17 to -26.54	Yes	****	<0.0001
1 µM:Trypsin vs. 1 µM:Thermolysin	-26.71	-37.03 to -16.40	Yes	****	<0.0001
1 µM:Trypsin vs. 1 µM:Proteinase K	-27.74	-38.06 to -17.43	Yes	****	<0.0001
1 µM:Trypsin vs. 5 µM:Pepsin	-4.053	-14.37 to 6.258	No	ns	0.9967
1 µM:Trypsin vs. 5 µM:Trypsin	-2.767	-13.08 to 7.545	No	ns	>0.9999
1 µM:Trypsin vs. 5 µM:Chymotrypsin	3.030	-7.282 to 13.34	No	ns	>0.9999
1 µM:Trypsin vs. 5 µM:Subtilisin	-28.43	-38.75 to -18.12	Yes	****	<0.0001
1 µM:Trypsin vs. 5 µM:Thermolysin	-17.75	-28.06 to -7.435	Yes	****	<0.0001
1 µM:Trypsin vs. 5 µM:Proteinase K	-29.28	-39.59 to -18.97	Yes	****	<0.0001
1 µM:Trypsin vs. 10 µM:Pepsin	4.170	-6.142 to 14.48	No	ns	0.9952
1 µM:Trypsin vs. 10 µM:Trypsin	-10.23	-20.54 to 0.08178	No	ns	0.0542
1 µM:Trypsin vs. 10 µM:Chymotrypsin	1.650	-8.662 to 11.96	No	ns	>0.9999
1 µM:Trypsin vs. 10 µM:Subtilisin	-13.13	-23.44 to -2.815	Yes	**	0.0023
1 µM:Trypsin vs. 10 µM:Thermolysin	-27.83	-38.15 to -17.52	Yes	****	<0.0001
1 µM:Trypsin vs. 10 µM:Proteinase K	-43.68	-53.99 to -33.36	Yes	****	<0.0001
1 µM:Trypsin vs. 20 µM:Pepsin	-2.637	-12.95 to 7.675	No	ns	>0.9999
1 µM:Trypsin vs. 20 µM:Trypsin	-26.04	-36.36 to -15.73	Yes	****	<0.0001
1 µM:Trypsin vs. 20 µM:Chymotrypsin	-15.96	-26.27 to -5.648	Yes	****	<0.0001
1 µM:Trypsin vs. 20 µM:Subtilisin	-8.097	-18.41 to 2.215	No	ns	0.3193

1 µM:Trypsin vs. 20 µM:Thermolysin	-42.01	-52.32 to -31.70	Yes	****	<0.0001
1 µM:Trypsin vs. 20 µM:Proteinase K	-12.33	-22.64 to -2.014	Yes	**	0.0058
1 µM:Chymotrypsin vs. 1 µM:Subtilisin	-32.01	-42.32 to -21.70	Yes	****	<0.0001
1 µM:Chymotrypsin vs. 1 µM:Thermolysin	-21.87	-32.18 to -11.56	Yes	****	<0.0001
1 µM:Chymotrypsin vs. 1 µM:Proteinase K	-22.90	-33.21 to -12.59	Yes	****	<0.0001
1 µM:Chymotrypsin vs. 5 µM:Pepsin	0.7900	-9.522 to 11.10	No	ns	>0.9999
1 µM:Chymotrypsin vs. 5 µM:Trypsin	2.077	-8.235 to 12.39	No	ns	>0.9999
1 µM:Chymotrypsin vs. 5 µM:Chymotrypsin	7.873	-2.438 to 18.19	No	ns	0.3687
1 µM:Chymotrypsin vs. 5 µM:Subtilisin	-23.59	-33.90 to -13.28	Yes	****	<0.0001
1 µM:Chymotrypsin vs. 5 µM:Thermolysin	-12.90	-23.22 to -2.592	Yes	**	0.0029
1 µM:Chymotrypsin vs. 5 µM:Proteinase K	-24.44	-34.75 to -14.12	Yes	****	<0.0001
1 µM:Chymotrypsin vs. 10 µM:Pepsin	9.013	-1.298 to 19.33	No	ns	0.1616
1 µM:Chymotrypsin vs. 10 µM:Trypsin	-5.387	-15.70 to 4.925	No	ns	0.9262
1 µM:Chymotrypsin vs. 10 µM:Chymotrypsin	6.493	-3.818 to 16.81	No	ns	0.7193
1 µM:Chymotrypsin vs. 10 µM:Subtilisin	-8.283	-18.60 to 2.028	No	ns	0.2812
1 µM:Chymotrypsin vs. 10 µM:Thermolysin	-22.99	-33.30 to -12.68	Yes	****	<0.0001
1 µM:Chymotrypsin vs. 10 µM:Proteinase K	-38.83	-49.15 to -28.52	Yes	****	<0.0001
1 µM:Chymotrypsin vs. 20 µM:Pepsin	2.207	-8.105 to 12.52	No	ns	>0.9999
1 µM:Chymotrypsin vs. 20 µM:Trypsin	-21.20	-31.51 to -10.89	Yes	****	<0.0001
1 µM:Chymotrypsin vs. 20 µM:Chymotrypsin	-11.12	-21.43 to -0.8049	Yes	*	0.0220
1 µM:Chymotrypsin vs. 20 µM:Subtilisin	-3.253	-13.57 to 7.058	No	ns	0.9999
1 µM:Chymotrypsin vs. 20 µM:Thermolysin	-37.17	-47.48 to -26.85	Yes	****	<0.0001
1 µM:Chymotrypsin vs. 20 µM:Proteinase K	-7.482	-17.79 to 2.830	No	ns	0.4636
1 µM:Subtilisin vs. 1 µM:Thermolysin	10.14	-0.1718 to 20.45	No	ns	0.0591

1 μM:Subtilisin vs. 1 μM:Proteinase K	9.110	-1.202 to 19.42	No	ns	0.1492
1 μM:Subtilisin vs. 5 μM:Pepsin	32.80	22.49 to 43.11	Yes	****	<0.0001
1 μM:Subtilisin vs. 5 μM:Trypsin	34.09	23.77 to 44.40	Yes	****	<0.0001
1 μM:Subtilisin vs. 5 μM:Chymotrypsin	39.88	29.57 to 50.20	Yes	****	<0.0001
1 μM:Subtilisin vs. 5 μM:Subtilisin	8.420	-1.892 to 18.73	No	ns	0.2552
1 μM:Subtilisin vs. 5 μM:Thermolysin	19.11	8.795 to 29.42	Yes	****	<0.0001
1 μM:Subtilisin vs. 5 μM:Proteinase K	7.573	-2.738 to 17.89	No	ns	0.4407
1 μM:Subtilisin vs. 10 μM:Pepsin	41.02	30.71 to 51.34	Yes	****	<0.0001
1 μM:Subtilisin vs. 10 μM:Trypsin	26.62	16.31 to 36.94	Yes	****	<0.0001
1 μM:Subtilisin vs. 10 μM:Chymotrypsin	38.50	28.19 to 48.82	Yes	****	<0.0001
1 μM:Subtilisin vs. 10 μM:Subtilisin	23.73	13.41 to 34.04	Yes	****	<0.0001
1 μM:Subtilisin vs. 10 μM:Thermolysin	9.020	-1.292 to 19.33	No	ns	0.1607
1 μM:Subtilisin vs. 10 μM:Proteinase K	-6.823	-17.14 to 3.488	No	ns	0.6357
1 μM:Subtilisin vs. 20 μM:Pepsin	34.22	23.90 to 44.53	Yes	****	<0.0001
1 μM:Subtilisin vs. 20 μM:Trypsin	10.81	0.4982 to 21.12	Yes	*	0.0303
1 μM:Subtilisin vs. 20 μM:Chymotrypsin	20.89	10.58 to 31.21	Yes	****	<0.0001
1 μM:Subtilisin vs. 20 μM:Subtilisin	28.76	18.44 to 39.07	Yes	****	<0.0001
1 μM:Subtilisin vs. 20 μM:Thermolysin	-5.157	-15.47 to 5.155	No	ns	0.9502
1 μM:Subtilisin vs. 20 μM:Proteinase K	24.53	14.22 to 34.84	Yes	****	<0.0001
1 μM:Thermolysin vs. 1 μM:Proteinase K	-1.030	-11.34 to 9.282	No	ns	>0.9999
1 μM:Thermolysin vs. 5 μM:Pepsin	22.66	12.35 to 32.97	Yes	****	<0.0001
1 μM:Thermolysin vs. 5 μM:Trypsin	23.95	13.63 to 34.26	Yes	****	<0.0001
1 μM:Thermolysin vs. 5 μM:Chymotrypsin	29.74	19.43 to 40.06	Yes	****	<0.0001
1 μM:Thermolysin vs. 5 μM:Subtilisin	-1.720	-12.03 to 8.592	No	ns	>0.9999

1 µM:Thermolysin vs. 5 µM:Thermolysin	8.967	-1.345 to 19.28	No	ns	0.1678
1 µM:Thermolysin vs. 5 µM:Proteinase K	-2.567	-12.88 to 7.745	No	ns	>0.9999
1 µM:Thermolysin vs. 10 µM:Pepsin	30.88	20.57 to 41.20	Yes	****	<0.0001
1 µM:Thermolysin vs. 10 µM:Trypsin	16.48	6.172 to 26.80	Yes	****	<0.0001
1 µM:Thermolysin vs. 10 µM:Chymotrypsin	28.36	18.05 to 38.68	Yes	****	<0.0001
1 µM:Thermolysin vs. 10 µM:Subtilisin	13.59	3.275 to 23.90	Yes	**	0.0013
1 µM:Thermolysin vs. 10 µM:Thermolysin	-1.120	-11.43 to 9.192	No	ns	>0.9999
1 µM:Thermolysin vs. 10 µM:Proteinase K	-16.96	-27.28 to -6.652	Yes	****	<0.0001
1 µM:Thermolysin vs. 20 µM:Pepsin	24.08	13.76 to 34.39	Yes	****	<0.0001
1 µM:Thermolysin vs. 20 µM:Trypsin	0.6700	-9.642 to 10.98	No	ns	>0.9999
1 µM:Thermolysin vs. 20 µM:Chymotrypsin	10.75	0.4415 to 21.07	Yes	*	0.0321
1 µM:Thermolysin vs. 20 µM:Subtilisin	18.62	8.305 to 28.93	Yes	****	<0.0001
1 µM:Thermolysin vs. 20 µM:Thermolysin	-15.30	-25.61 to -4.985	Yes	***	0.0002
1 µM:Thermolysin vs. 20 µM:Proteinase K	14.39	4.076 to 24.70	Yes	***	0.0005
1 µM:Proteinase K vs. 5 µM:Pepsin	23.69	13.38 to 34.00	Yes	****	<0.0001
1 µM:Proteinase K vs. 5 µM:Trypsin	24.98	14.66 to 35.29	Yes	****	<0.0001
1 µM:Proteinase K vs. 5 µM:Chymotrypsin	30.77	20.46 to 41.09	Yes	****	<0.0001
1 µM:Proteinase K vs. 5 µM:Subtilisin	-0.6900	-11.00 to 9.622	No	ns	>0.9999
1 µM:Proteinase K vs. 5 µM:Thermolysin	9.997	-0.3151 to 20.31	No	ns	0.0677
1 µM:Proteinase K vs. 5 µM:Proteinase K	-1.537	-11.85 to 8.775	No	ns	>0.9999
1 µM:Proteinase K vs. 10 µM:Pepsin	31.91	21.60 to 42.23	Yes	****	<0.0001
1 µM:Proteinase K vs. 10 µM:Trypsin	17.51	7.202 to 27.83	Yes	****	<0.0001
1 µM:Proteinase K vs. 10 µM:Chymotrypsin	29.39	19.08 to 39.71	Yes	****	<0.0001
1 µM:Proteinase K vs. 10 µM:Subtilisin	14.62	4.305 to 24.93	Yes	***	0.0004

1 µM:Proteinase K vs. 10 µM:Thermolysin	-0.09000	-10.40 to 10.22	No	ns	>0.9999
1 µM:Proteinase K vs. 10 µM:Proteinase K	-15.93	-26.25 to -5.622	Yes	****	<0.0001
1 µM:Proteinase K vs. 20 µM:Pepsin	25.11	14.79 to 35.42	Yes	****	<0.0001
1 µM:Proteinase K vs. 20 µM:Trypsin	1.700	-8.612 to 12.01	No	ns	>0.9999
1 µM:Proteinase K vs. 20 µM:Chymotrypsin	11.78	1.472 to 22.10	Yes	*	0.0107
1 µM:Proteinase K vs. 20 µM:Subtilisin	19.65	9.335 to 29.96	Yes	****	<0.0001
1 µM:Proteinase K vs. 20 µM:Thermolysin	-14.27	-24.58 to -3.955	Yes	***	0.0006
1 µM:Proteinase K vs. 20 µM:Proteinase K	15.42	5.106 to 25.73	Yes	***	0.0001
5 µM:Pepsin vs. 5 µM:Trypsin	1.287	-9.025 to 11.60	No	ns	>0.9999
5 µM:Pepsin vs. 5 µM:Chymotrypsin	7.083	-3.228 to 17.40	No	ns	0.5674
5 µM:Pepsin vs. 5 µM:Subtilisin	-24.38	-34.69 to -14.07	Yes	****	<0.0001
5 µM:Pepsin vs. 5 µM:Thermolysin	-13.69	-24.01 to -3.382	Yes	**	0.0011
5 µM:Pepsin vs. 5 µM:Proteinase K	-25.23	-35.54 to -14.91	Yes	****	<0.0001
5 µM:Pepsin vs. 10 µM:Pepsin	8.223	-2.088 to 18.54	No	ns	0.2931
5 µM:Pepsin vs. 10 µM:Trypsin	-6.177	-16.49 to 4.135	No	ns	0.7925
5 µM:Pepsin vs. 10 µM:Chymotrypsin	5.703	-4.608 to 16.02	No	ns	0.8821
5 µM:Pepsin vs. 10 µM:Subtilisin	-9.073	-19.39 to 1.238	No	ns	0.1538
5 µM:Pepsin vs. 10 µM:Thermolysin	-23.78	-34.09 to -13.47	Yes	****	<0.0001
5 µM:Pepsin vs. 10 µM:Proteinase K	-39.62	-49.94 to -29.31	Yes	****	<0.0001
5 µM:Pepsin vs. 20 µM:Pepsin	1.417	-8.895 to 11.73	No	ns	>0.9999
5 µM:Pepsin vs. 20 µM:Trypsin	-21.99	-32.30 to -11.68	Yes	****	<0.0001
5 µM:Pepsin vs. 20 µM:Chymotrypsin	-11.91	-22.22 to -1.595	Yes	**	0.0093
5 µM:Pepsin vs. 20 µM:Subtilisin	-4.043	-14.36 to 6.268	No	ns	0.9968
5 µM:Pepsin vs. 20 µM:Thermolysin	-37.96	-48.27 to -27.64	Yes	****	<0.0001

5 µM:Pepsin vs. 20 µM:Proteinase K	-8.272	-18.58 to 2.040	No	ns	0.2833
5 µM:Trypsin vs. 5 µM:Chymotrypsin	5.797	-4.515 to 16.11	No	ns	0.8666
5 µM:Trypsin vs. 5 µM:Subtilisin	-25.67	-35.98 to -15.35	Yes	****	<0.0001
5 µM:Trypsin vs. 5 µM:Thermolysin	-14.98	-25.29 to -4.668	Yes	***	0.0002
5 µM:Trypsin vs. 5 µM:Proteinase K	-26.51	-36.83 to -16.20	Yes	****	<0.0001
5 µM:Trypsin vs. 10 µM:Pepsin	6.937	-3.375 to 17.25	No	ns	0.6060
5 µM:Trypsin vs. 10 µM:Trypsin	-7.463	-17.78 to 2.848	No	ns	0.4684
5 µM:Trypsin vs. 10 µM:Chymotrypsin	4.417	-5.895 to 14.73	No	ns	0.9904
5 µM:Trypsin vs. 10 µM:Subtilisin	-10.36	-20.67 to -0.04822	Yes	*	0.0477
5 µM:Trypsin vs. 10 µM:Thermolysin	-25.07	-35.38 to -14.75	Yes	****	<0.0001
5 µM:Trypsin vs. 10 µM:Proteinase K	-40.91	-51.22 to -30.60	Yes	****	<0.0001
5 µM:Trypsin vs. 20 µM:Pepsin	0.1300	-10.18 to 10.44	No	ns	>0.9999
5 µM:Trypsin vs. 20 µM:Trypsin	-23.28	-33.59 to -12.96	Yes	****	<0.0001
5 µM:Trypsin vs. 20 µM:Chymotrypsin	-13.19	-23.51 to -2.882	Yes	**	0.0021
5 µM:Trypsin vs. 20 µM:Subtilisin	-5.330	-15.64 to 4.982	No	ns	0.9327
5 µM:Trypsin vs. 20 µM:Thermolysin	-39.24	-49.56 to -28.93	Yes	****	<0.0001
5 µM:Trypsin vs. 20 µM:Proteinase K	-9.559	-19.87 to 0.7528	No	ns	0.1013
5 µM:Chymotrypsin vs. 5 µM:Subtilisin	-31.46	-41.78 to -21.15	Yes	****	<0.0001
5 µM:Chymotrypsin vs. 5 µM:Thermolysin	-20.78	-31.09 to -10.46	Yes	****	<0.0001
5 µM:Chymotrypsin vs. 5 µM:Proteinase K	-32.31	-42.62 to -22.00	Yes	****	<0.0001
5 µM:Chymotrypsin vs. 10 µM:Pepsin	1.140	-9.172 to 11.45	No	ns	>0.9999
5 µM:Chymotrypsin vs. 10 µM:Trypsin	-13.26	-23.57 to -2.948	Yes	**	0.0019
5 µM:Chymotrypsin vs. 10 µM:Chymotrypsin	-1.380	-11.69 to 8.932	No	ns	>0.9999
5 µM:Chymotrypsin vs. 10 µM:Subtilisin	-16.16	-26.47 to -5.845	Yes	****	<0.0001

5 µM:Chymotrypsin vs. 10 µM:Thermolysin	-30.86	-41.18 to -20.55	Yes	****	<0.0001
5 µM:Chymotrypsin vs. 10 µM:Proteinase K	-46.71	-57.02 to -36.39	Yes	****	<0.0001
5 µM:Chymotrypsin vs. 20 µM:Pepsin	-5.667	-15.98 to 4.645	No	ns	0.8879
5 µM:Chymotrypsin vs. 20 µM:Trypsin	-29.07	-39.39 to -18.76	Yes	****	<0.0001
5 µM:Chymotrypsin vs. 20 µM:Chymotrypsin	-18.99	-29.30 to -8.678	Yes	****	<0.0001
5 µM:Chymotrypsin vs. 20 µM:Subtilisin	-11.13	-21.44 to -0.8149	Yes	*	0.0217
5 µM:Chymotrypsin vs. 20 µM:Thermolysin	-45.04	-55.35 to -34.73	Yes	****	<0.0001
5 µM:Chymotrypsin vs. 20 µM:Proteinase K	-15.36	-25.67 to -5.044	Yes	***	0.0001
5 µM:Subtilisin vs. 5 µM:Thermolysin	10.69	0.3749 to 21.00	Yes	*	0.0344
5 µM:Subtilisin vs. 5 µM:Proteinase K	-0.8467	-11.16 to 9.465	No	ns	>0.9999
5 µM:Subtilisin vs. 10 µM:Pepsin	32.60	22.29 to 42.92	Yes	****	<0.0001
5 µM:Subtilisin vs. 10 µM:Trypsin	18.20	7.892 to 28.52	Yes	****	<0.0001
5 µM:Subtilisin vs. 10 µM:Chymotrypsin	30.08	19.77 to 40.40	Yes	****	<0.0001
5 µM:Subtilisin vs. 10 µM:Subtilisin	15.31	4.995 to 25.62	Yes	***	0.0002
5 µM:Subtilisin vs. 10 µM:Thermolysin	0.6000	-9.712 to 10.91	No	ns	>0.9999
5 µM:Subtilisin vs. 10 µM:Proteinase K	-15.24	-25.56 to -4.932	Yes	***	0.0002
5 µM:Subtilisin vs. 20 µM:Pepsin	25.80	15.48 to 36.11	Yes	****	<0.0001
5 µM:Subtilisin vs. 20 µM:Trypsin	2.390	-7.922 to 12.70	No	ns	>0.9999
5 µM:Subtilisin vs. 20 µM:Chymotrypsin	12.47	2.162 to 22.79	Yes	**	0.0049
5 µM:Subtilisin vs. 20 µM:Subtilisin	20.34	10.02 to 30.65	Yes	****	<0.0001
5 µM:Subtilisin vs. 20 µM:Thermolysin	-13.58	-23.89 to -3.265	Yes	**	0.0013
5 µM:Subtilisin vs. 20 µM:Proteinase K	16.11	5.796 to 26.42	Yes	****	<0.0001
5 µM:Thermolysin vs. 5 µM:Proteinase K	-11.53	-21.85 to -1.222	Yes	*	0.0140
5 µM:Thermolysin vs. 10 µM:Pepsin	21.92	11.60 to 32.23	Yes	****	<0.0001

5 µM:Thermolysin vs. 10 µM:Trypsin	7.517	-2.795 to 17.83	No	ns	0.4549
5 µM:Thermolysin vs. 10 µM:Chymotrypsin	19.40	9.085 to 29.71	Yes	****	<0.0001
5 µM:Thermolysin vs. 10 µM:Subtilisin	4.620	-5.692 to 14.93	No	ns	0.9839
5 µM:Thermolysin vs. 10 µM:Thermolysin	-10.09	-20.40 to 0.2251	No	ns	0.0622
5 µM:Thermolysin vs. 10 µM:Proteinase K	-25.93	-36.24 to -15.62	Yes	****	<0.0001
5 µM:Thermolysin vs. 20 µM:Pepsin	15.11	4.798 to 25.42	Yes	***	0.0002
5 µM:Thermolysin vs. 20 µM:Trypsin	-8.297	-18.61 to 2.015	No	ns	0.2785
5 µM:Thermolysin vs. 20 µM:Chymotrypsin	1.787	-8.525 to 12.10	No	ns	>0.9999
5 µM:Thermolysin vs. 20 µM:Subtilisin	9.650	-0.6618 to 19.96	No	ns	0.0934
5 µM:Thermolysin vs. 20 µM:Thermolysin	-24.26	-34.58 to -13.95	Yes	****	<0.0001
5 µM:Thermolysin vs. 20 µM:Proteinase K	5.421	-4.891 to 15.73	No	ns	0.9221
5 µM:Proteinase K vs. 10 µM:Pepsin	33.45	23.14 to 43.76	Yes	****	<0.0001
5 µM:Proteinase K vs. 10 µM:Trypsin	19.05	8.738 to 29.36	Yes	****	<0.0001
5 µM:Proteinase K vs. 10 µM:Chymotrypsin	30.93	20.62 to 41.24	Yes	****	<0.0001
5 µM:Proteinase K vs. 10 µM:Subtilisin	16.15	5.842 to 26.47	Yes	****	<0.0001
5 µM:Proteinase K vs. 10 µM:Thermolysin	1.447	-8.865 to 11.76	No	ns	>0.9999
5 µM:Proteinase K vs. 10 µM:Proteinase K	-14.40	-24.71 to -4.085	Yes	***	0.0005
5 µM:Proteinase K vs. 20 µM:Pepsin	26.64	16.33 to 36.96	Yes	****	<0.0001
5 µM:Proteinase K vs. 20 µM:Trypsin	3.237	-7.075 to 13.55	No	ns	0.9999
5 µM:Proteinase K vs. 20 µM:Chymotrypsin	13.32	3.008 to 23.63	Yes	**	0.0018
5 µM:Proteinase K vs. 20 µM:Subtilisin	21.18	10.87 to 31.50	Yes	****	<0.0001
5 µM:Proteinase K vs. 20 µM:Thermolysin	-12.73	-23.04 to -2.418	Yes	**	0.0036
5 µM:Proteinase K vs. 20 µM:Proteinase K	16.95	6.643 to 27.27	Yes	****	<0.0001
10 µM:Pepsin vs. 10 µM:Trypsin	-14.40	-24.71 to -4.088	Yes	***	0.0005

10 µM:Pepsin vs. 10 µM:Chymotrypsin	-2.520	-12.83 to 7.792	No	ns	>0.9999
10 µM:Pepsin vs. 10 µM:Subtilisin	-17.30	-27.61 to -6.985	Yes	****	<0.0001
10 µM:Pepsin vs. 10 µM:Thermolysin	-32.00	-42.32 to -21.69	Yes	****	<0.0001
10 µM:Pepsin vs. 10 µM:Proteinase K	-47.85	-58.16 to -37.53	Yes	****	<0.0001
10 µM:Pepsin vs. 20 µM:Pepsin	-6.807	-17.12 to 3.505	No	ns	0.6400
10 µM:Pepsin vs. 20 µM:Trypsin	-30.21	-40.53 to -19.90	Yes	****	<0.0001
10 µM:Pepsin vs. 20 µM:Chymotrypsin	-20.13	-30.44 to -9.818	Yes	****	<0.0001
10 µM:Pepsin vs. 20 µM:Subtilisin	-12.27	-22.58 to -1.955	Yes	**	0.0062
10 µM:Pepsin vs. 20 µM:Thermolysin	-46.18	-56.49 to -35.87	Yes	****	<0.0001
10 µM:Pepsin vs. 20 µM:Proteinase K	-16.50	-26.81 to -6.184	Yes	****	<0.0001
10 µM:Trypsin vs. 10 µM:Chymotrypsin	11.88	1.568 to 22.19	Yes	**	0.0096
10 µM:Trypsin vs. 10 µM:Subtilisin	-2.897	-13.21 to 7.415	No	ns	>0.9999
10 µM:Trypsin vs. 10 µM:Thermolysin	-17.60	-27.92 to -7.292	Yes	****	<0.0001
10 µM:Trypsin vs. 10 µM:Proteinase K	-33.45	-43.76 to -23.13	Yes	****	<0.0001
10 µM:Trypsin vs. 20 µM:Pepsin	7.593	-2.718 to 17.91	No	ns	0.4358
10 µM:Trypsin vs. 20 µM:Trypsin	-15.81	-26.13 to -5.502	Yes	****	<0.0001
10 µM:Trypsin vs. 20 µM:Chymotrypsin	-5.730	-16.04 to 4.582	No	ns	0.8778
10 µM:Trypsin vs. 20 µM:Subtilisin	2.133	-8.178 to 12.45	No	ns	>0.9999
10 µM:Trypsin vs. 20 µM:Thermolysin	-31.78	-42.09 to -21.47	Yes	****	<0.0001
10 µM:Trypsin vs. 20 µM:Proteinase K	-2.096	-12.41 to 8.216	No	ns	>0.9999
10 µM:Chymotrypsin vs. 10 µM:Subtilisin	-14.78	-25.09 to -4.465	Yes	***	0.0003
10 µM:Chymotrypsin vs. 10 µM:Thermolysin	-29.48	-39.80 to -19.17	Yes	****	<0.0001
10 µM:Chymotrypsin vs. 10 µM:Proteinase K	-45.33	-55.64 to -35.01	Yes	****	<0.0001
10 µM:Chymotrypsin vs. 20 µM:Pepsin	-4.287	-14.60 to 6.025	No	ns	0.9933

10 µM:Chymotrypsin vs. 20 µM:Trypsin	-27.69	-38.01 to -17.38	Yes	****	<0.0001
10 µM:Chymotrypsin vs. 20 µM:Chymotrypsin	-17.61	-27.92 to -7.298	Yes	****	<0.0001
10 µM:Chymotrypsin vs. 20 µM:Subtilisin	-9.747	-20.06 to 0.5651	No	ns	0.0855
10 µM:Chymotrypsin vs. 20 µM:Thermolysin	-43.66	-53.97 to -33.35	Yes	****	<0.0001
10 µM:Chymotrypsin vs. 20 µM:Proteinase K	-13.98	-24.29 to -3.664	Yes	***	0.0008
10 µM:Subtilisin vs. 10 µM:Thermolysin	-14.71	-25.02 to -4.395	Yes	***	0.0003
10 µM:Subtilisin vs. 10 µM:Proteinase K	-30.55	-40.86 to -20.24	Yes	****	<0.0001
10 µM:Subtilisin vs. 20 µM:Pepsin	10.49	0.1782 to 20.80	Yes	*	0.0419
10 µM:Subtilisin vs. 20 µM:Trypsin	-12.92	-23.23 to -2.605	Yes	**	0.0029
10 µM:Subtilisin vs. 20 µM:Chymotrypsin	-2.833	-13.15 to 7.478	No	ns	>0.9999
10 µM:Subtilisin vs. 20 µM:Subtilisin	5.030	-5.282 to 15.34	No	ns	0.9608
10 µM:Subtilisin vs. 20 µM:Thermolysin	-28.88	-39.20 to -18.57	Yes	****	<0.0001
10 µM:Subtilisin vs. 20 µM:Proteinase K	0.8011	-9.511 to 11.11	No	ns	>0.9999
10 µM:Thermolysin vs. 10 µM:Proteinase K	-15.84	-26.16 to -5.532	Yes	****	<0.0001
10 µM:Thermolysin vs. 20 µM:Pepsin	25.20	14.88 to 35.51	Yes	****	<0.0001
10 µM:Thermolysin vs. 20 µM:Trypsin	1.790	-8.522 to 12.10	No	ns	>0.9999
10 µM:Thermolysin vs. 20 µM:Chymotrypsin	11.87	1.562 to 22.19	Yes	**	0.0096
10 µM:Thermolysin vs. 20 µM:Subtilisin	19.74	9.425 to 30.05	Yes	****	<0.0001
10 µM:Thermolysin vs. 20 µM:Thermolysin	-14.18	-24.49 to -3.865	Yes	***	0.0006
10 µM:Thermolysin vs. 20 µM:Proteinase K	15.51	5.196 to 25.82	Yes	***	0.0001
10 µM:Proteinase K vs. 20 µM:Pepsin	41.04	30.73 to 51.35	Yes	****	<0.0001
10 µM:Proteinase K vs. 20 µM:Trypsin	17.63	7.322 to 27.95	Yes	****	<0.0001
10 µM:Proteinase K vs. 20 µM:Chymotrypsin	27.72	17.40 to 38.03	Yes	****	<0.0001
10 µM:Proteinase K vs. 20 µM:Subtilisin	35.58	25.27 to 45.89	Yes	****	<0.0001

10 µM:Proteinase K vs. 20 µM:Thermolysin	1.667	-8.645 to 11.98	No	ns	>0.9999
10 µM:Proteinase K vs. 20 µM:Proteinase K	31.35	21.04 to 41.66	Yes	****	<0.0001
20 µM:Pepsin vs. 20 µM:Trypsin	-23.41	-33.72 to -13.09	Yes	****	<0.0001
20 µM:Pepsin vs. 20 µM:Chymotrypsin	-13.32	-23.64 to -3.012	Yes	**	0.0018
20 µM:Pepsin vs. 20 µM:Subtilisin	-5.460	-15.77 to 4.852	No	ns	0.9172
20 µM:Pepsin vs. 20 µM:Thermolysin	-39.37	-49.69 to -29.06	Yes	****	<0.0001
20 µM:Pepsin vs. 20 µM:Proteinase K	-9.689	-20.00 to 0.6228	No	ns	0.0901
20 µM:Trypsin vs. 20 µM:Chymotrypsin	10.08	-0.2285 to 20.40	No	ns	0.0624
20 µM:Trypsin vs. 20 µM:Subtilisin	17.95	7.635 to 28.26	Yes	****	<0.0001
20 µM:Trypsin vs. 20 µM:Thermolysin	-15.97	-26.28 to -5.655	Yes	****	<0.0001
20 µM:Trypsin vs. 20 µM:Proteinase K	13.72	3.406 to 24.03	Yes	**	0.0011
20 µM:Chymotrypsin vs. 20 µM:Subtilisin	7.863	-2.448 to 18.18	No	ns	0.3710
20 µM:Chymotrypsin vs. 20 µM:Thermolysin	-26.05	-36.36 to -15.74	Yes	****	<0.0001
20 µM:Chymotrypsin vs. 20 µM:Proteinase K	3.634	-6.677 to 13.95	No	ns	0.9993
20 µM:Subtilisin vs. 20 µM:Thermolysin	-33.91	-44.23 to -23.60	Yes	****	<0.0001
20 µM:Subtilisin vs. 20 µM:Proteinase K	-4.229	-14.54 to 6.083	No	ns	0.9943
20 µM:Thermolysin vs. 20 µM:Proteinase K	29.68	19.37 to 40.00	Yes	****	<0.0001

Table S13. The viability data for *A. platensis* hydrolysates tested on QM7 cell line at reduced tryptose phosphate broth condition (2%) by resazurin assay

**Sample (µM)**	**Control**			**AP - trypsin**			**AP - chymotrypsin**			**AP - thermolysin**			**AP - proteinase K**		
**0**	101.22	94.76	104.02	x											
**1**	x			92.73	85.29	89.23	92.02	103.83	85.67	94.82	88.00	90.91	86.77	103.16	97.83
**5**				98.78	96.51	95.55	132.97	127.88	118.40	97.22	91.07	96.48	93.84	103.26	95.06

**10**		101.50	97.65	88.99	118.42	141.49	144.93	97.69	96.72	90.74	83.91	99.35	85.94
**20**		94.33	84.21	83.38	153.75	162.53	165.64	101.55	103.49	107.17	96.18	99.33	101.00

Table S14A. One-way ANOVA statistical analysis for *A. platensis* hydrolysates tested on QM7 cell line at reduced tryptose phosphate broth condition (2%) by resazurin assay

**ANOVA summary**					
**F**	26.77				
**P value**	<0.0001				
**P value summary**	****				
**Significant diff. among means (P < 0.05)?**	Yes				
**R square**	0.9265				
**ANOVA table**	**SS**	**DF**	**MS**	**F (DFn, DFd)**	**P value**
**Treatment (between columns)**	18155	16	1135	F (16, 34) = 26.77	P<0.0001
**Residual (within columns)**	1441	34	42.39		
**Total**	19597	50			

Table S14B. Dunnett's multiple comparisons test for *A. platensis* hydrolysates tested on QM7 cell line at reduced tryptose phosphate broth condition (2%) by resazurin assay

**Dunnett's multiple comparisons test**	**Mean Diff.**	**95.00% CI of diff.**	**Significant?**	**Summary**	**Adjusted P Value**
Control vs. Trypsin 1 µM	10.92	-5.113 to 26.95	No	ns	0.3446
Control vs. Chymotrypsin 1 µM	6.160	-9.869 to 22.19	No	ns	0.9225
Control vs. Thermolysin 1 µM	8.757	-7.273 to 24.79	No	ns	0.6147
Control vs. Proteinase K 1 µM	4.080	-11.95 to 20.11	No	ns	0.9953
Control vs. Trypsin 5 µM	3.053	-12.98 to 19.08	No	ns	0.9991
Control vs. Chymotrpysin 5 µM	-26.42	-42.45 to -10.39	Yes	***	0.0003

Control vs. Thermoylsin 5 µM	5.077	-10.95 to 21.11	No	ns	0.9806
Control vs. Proteinase K 5 µM	2.613	-13.42 to 18.64	No	ns	0.9993
Control vs. Trypsin 10 µM	3.953	-12.08 to 19.98	No	ns	0.9955
Control vs. Chymotrypsin 10 µM	-34.95	-50.98 to -18.92	Yes	****	<0.0001
Control vs. Thermolysin 10 µM	4.950	-11.08 to 20.98	No	ns	0.9849
Control vs. Proteinase K 10 µM	10.27	-5.763 to 26.30	No	ns	0.4177
Control vs. Trypsin 20 µM	12.69	-3.336 to 28.72	No	ns	0.1902
Control vs. Chymotrypsin 20 µM	-60.64	-76.67 to -44.61	Yes	****	<0.0001
Control vs. Thermolysin 20 µM	-4.070	-20.10 to 11.96	No	ns	0.9953
Control vs. Proteinase K 20 µM	1.163	-14.87 to 17.19	No	ns	0.9997

Table S14C. Two-way ANOVA statistical analysis for *A. platensis* hydrolysates tested on QM7 cell line at reduced tryptose phosphate broth condition (2%) by resazurin assay

**Two-way ANOVA**	Ordinary				
**Alpha**	0.05				
**Source of Variation**	**% of total variation**	**P value**	**P value summary**	**Significant?**	
**Interaction**	25.59	<0.0001	****	Yes	
**Row (concentration) Factor**	12.64	<0.0001	****	Yes	
**Column (enzyme) Factor**	54.62	<0.0001	****	Yes	
**ANOVA table**	**SS**	**DF**	**MS**	**F (DFn, DFd)**	**P value**
**Interaction**	4996	9	555.2	F (9, 32) = 12.72	P<0.0001
**Row Factor**	2467	3	822.3	F (3, 32) = 18.85	P<0.0001
**Column Factor**	10662	3	3554	F (3, 32) = 81.46	P<0.0001
**Residual**	1396	32	43.63		

Table S14D. Tukey's multiple comparisons test for *A. platensis* hydrolysates tested on QM7 cell line at reduced tryptose phosphate broth condition (2%) by resazurin assay

Tukey's multiple comparisons test	Mean Diff.	95.00% CI of diff.	Significant?	Summary	Adjusted P Value
1 µM:Trypsin vs. 1 µM:Chymotrypsin	-4.757	-24.76 to 15.24	No	ns	>0.9999
1 µM:Trypsin vs. 1 µM:Thermolysin	-2.160	-22.16 to 17.84	No	ns	>0.9999
1 µM:Trypsin vs. 1 µM:Proteinase K	-6.837	-26.84 to 13.16	No	ns	0.9945
1 µM:Trypsin vs. 5 µM:Trypsin	-7.863	-27.86 to 12.14	No	ns	0.9799
1 µM:Trypsin vs. 5 µM:Chymotrypsin	-37.33	-57.33 to -17.33	Yes	****	<0.0001
1 µM:Trypsin vs. 5 µM:Thermolysin	-5.840	-25.84 to 14.16	No	ns	0.9989
1 µM:Trypsin vs. 5 µM:Proteinase K	-8.303	-28.30 to 11.70	No	ns	0.9682
1 µM:Trypsin vs. 10 µM:Trypsin	-6.963	-26.96 to 13.04	No	ns	0.9935
1 µM:Trypsin vs. 10 µM:Chymotrypsin	-45.86	-65.86 to -25.86	Yes	****	<0.0001
1 µM:Trypsin vs. 10 µM:Thermolysin	-5.967	-25.97 to 14.03	No	ns	0.9987
1 µM:Trypsin vs. 10 µM:Proteinase K	-0.6500	-20.65 to 19.35	No	ns	>0.9999
1 µM:Trypsin vs. 20 µM:Trypsin	1.777	-18.22 to 21.78	No	ns	>0.9999
1 µM:Trypsin vs. 20 µM:Chymotrypsin	-71.56	-91.56 to -51.56	Yes	****	<0.0001
1 µM:Trypsin vs. 20 µM:Thermolysin	-14.99	-34.99 to 5.012	No	ns	0.3292
1 µM:Trypsin vs. 20 µM:Proteinase K	-9.753	-29.75 to 10.25	No	ns	0.8957
1 µM:Chymotrypsin vs. 1 µM:Thermolysin	2.597	-17.40 to 22.60	No	ns	>0.9999
1 µM:Chymotrypsin vs. 1 µM:Proteinase K	-2.080	-22.08 to 17.92	No	ns	>0.9999
1 µM:Chymotrypsin vs. 5 µM:Trypsin	-3.107	-23.11 to 16.89	No	ns	>0.9999
1 µM:Chymotrypsin vs. 5 µM:Chymotrypsin	-32.58	-52.58 to -12.58	Yes	****	<0.0001
1 µM:Chymotrypsin vs. 5 µM:Thermolysin	-1.083	-21.08 to 18.92	No	ns	>0.9999
1 µM:Chymotrypsin vs. 5 µM:Proteinase K	-3.547	-23.55 to 16.45	No	ns	>0.9999

1 µM:Chymotrypsin vs. 10 µM:Trypsin	-2.207	-22.21 to 17.79	No	ns	>0.9999
1 µM:Chymotrypsin vs. 10 µM:Chymotrypsin	-41.11	-61.11 to -21.11	Yes	****	<0.0001
1 µM:Chymotrypsin vs. 10 µM:Thermolysin	-1.210	-21.21 to 18.79	No	ns	>0.9999
1 µM:Chymotrypsin vs. 10 µM:Proteinase K	4.107	-15.89 to 24.11	No	ns	>0.9999
1 µM:Chymotrypsin vs. 20 µM:Trypsin	6.533	-13.47 to 26.53	No	ns	0.9965
1 µM:Chymotrypsin vs. 20 µM:Chymotrypsin	-66.80	-86.80 to -46.80	Yes	****	<0.0001
1 µM:Chymotrypsin vs. 20 µM:Thermolysin	-10.23	-30.23 to 9.768	No	ns	0.8589
1 µM:Chymotrypsin vs. 20 µM:Proteinase K	-4.997	-25.00 to 15.00	No	ns	0.9998
1 µM:Thermolysin vs. 1 µM:Proteinase K	-4.677	-24.68 to 15.32	No	ns	>0.9999
1 µM:Thermolysin vs. 5 µM:Trypsin	-5.703	-25.70 to 14.30	No	ns	0.9992
1 µM:Thermolysin vs. 5 µM:Chymotrypsin	-35.17	-55.17 to -15.17	Yes	****	<0.0001
1 µM:Thermolysin vs. 5 µM:Thermolysin	-3.680	-23.68 to 16.32	No	ns	>0.9999
1 µM:Thermolysin vs. 5 µM:Proteinase K	-6.143	-26.14 to 13.86	No	ns	0.9982
1 µM:Thermolysin vs. 10 µM:Trypsin	-4.803	-24.80 to 15.20	No	ns	0.9999
1 µM:Thermolysin vs. 10 µM:Chymotrypsin	-43.70	-63.70 to -23.70	Yes	****	<0.0001
1 µM:Thermolysin vs. 10 µM:Thermolysin	-3.807	-23.81 to 16.19	No	ns	>0.9999
1 µM:Thermolysin vs. 10 µM:Proteinase K	1.510	-18.49 to 21.51	No	ns	>0.9999
1 µM:Thermolysin vs. 20 µM:Trypsin	3.937	-16.06 to 23.94	No	ns	>0.9999
1 µM:Thermolysin vs. 20 µM:Chymotrypsin	-69.40	-89.40 to -49.40	Yes	****	<0.0001
1 µM:Thermolysin vs. 20 µM:Thermolysin	-12.83	-32.83 to 7.172	No	ns	0.5748
1 µM:Thermolysin vs. 20 µM:Proteinase K	-7.593	-27.59 to 12.41	No	ns	0.9853
1 µM:Proteinase K vs. 5 µM:Trypsin	-1.027	-21.03 to 18.97	No	ns	>0.9999
1 µM:Proteinase K vs. 5 µM:Chymotrypsin	-30.50	-50.50 to -10.50	Yes	***	0.0003
1 µM:Proteinase K vs. 5 µM:Thermolysin	0.9967	-19.00 to 21.00	No	ns	>0.9999
1 µM:Proteinase K vs. 5 µM:Proteinase K	-1.467	-21.47 to 18.53	No	ns	>0.9999

1 µM:Proteinase K vs. 10 µM:Trypsin	-0.1267	-20.13 to 19.87	No	ns	>0.9999
1 µM:Proteinase K vs. 10 µM:Chymotrypsin	-39.03	-59.03 to -19.03	Yes	****	<0.0001
1 µM:Proteinase K vs. 10 µM:Thermolysin	0.8700	-19.13 to 20.87	No	ns	>0.9999
1 µM:Proteinase K vs. 10 µM:Proteinase K	6.187	-13.81 to 26.19	No	ns	0.9980
1 µM:Proteinase K vs. 20 µM:Trypsin	8.613	-11.39 to 28.61	No	ns	0.9574
1 µM:Proteinase K vs. 20 µM:Chymotrypsin	-64.72	-84.72 to -44.72	Yes	****	<0.0001
1 µM:Proteinase K vs. 20 µM:Thermolysin	-8.150	-28.15 to 11.85	No	ns	0.9728
1 µM:Proteinase K vs. 20 µM:Proteinase K	-2.917	-22.92 to 17.08	No	ns	>0.9999
5 µM:Trypsin vs. 5 µM:Chymotrypsin	-29.47	-49.47 to -9.472	Yes	***	0.0005
5 µM:Trypsin vs. 5 µM:Thermolysin	2.023	-17.98 to 22.02	No	ns	>0.9999
5 µM:Trypsin vs. 5 µM:Proteinase K	-0.4400	-20.44 to 19.56	No	ns	>0.9999
5 µM:Trypsin vs. 10 µM:Trypsin	0.9000	-19.10 to 20.90	No	ns	>0.9999
5 µM:Trypsin vs. 10 µM:Chymotrypsin	-38.00	-58.00 to -18.00	Yes	****	<0.0001
5 µM:Trypsin vs. 10 µM:Thermolysin	1.897	-18.10 to 21.90	No	ns	>0.9999
5 µM:Trypsin vs. 10 µM:Proteinase K	7.213	-12.79 to 27.21	No	ns	0.9908
5 µM:Trypsin vs. 20 µM:Trypsin	9.640	-10.36 to 29.64	No	ns	0.9034
5 µM:Trypsin vs. 20 µM:Chymotrypsin	-63.69	-83.69 to -43.69	Yes	****	<0.0001
5 µM:Trypsin vs. 20 µM:Thermolysin	-7.123	-27.12 to 12.88	No	ns	0.9919
5 µM:Trypsin vs. 20 µM:Proteinase K	-1.890	-21.89 to 18.11	No	ns	>0.9999
5 µM:Chymotrypsin vs. 5 µM:Thermolysin	31.49	11.49 to 51.49	Yes	***	0.0002
5 µM:Chymotrypsin vs. 5 µM:Proteinase K	29.03	9.032 to 49.03	Yes	***	0.0006
5 µM:Chymotrypsin vs. 10 µM:Trypsin	30.37	10.37 to 50.37	Yes	***	0.0003
5 µM:Chymotrypsin vs. 10 µM:Chymotrypsin	-8.530	-28.53 to 11.47	No	ns	0.9606
5 µM:Chymotrypsin vs. 10 µM:Thermolysin	31.37	11.37 to 51.37	Yes	***	0.0002
5 µM:Chymotrypsin vs. 10 µM:Proteinase K	36.68	16.68 to 56.68	Yes	****	<0.0001

5 µM:Chymotrypsin vs. 20 µM:Trypsin	39.11	19.11 to 59.11	Yes	****	<0.0001
5 µM:Chymotrypsin vs. 20 µM:Chymotrypsin	-34.22	-54.22 to -14.22	Yes	****	<0.0001
5 µM:Chymotrypsin vs. 20 µM:Thermolysin	22.35	2.348 to 42.35	Yes	*	0.0172
5 µM:Chymotrypsin vs. 20 µM:Proteinase K	27.58	7.582 to 47.58	Yes	**	0.0013
5 µM:Thermolysin vs. 5 µM:Proteinase K	-2.463	-22.46 to 17.54	No	ns	>0.9999
5 µM:Thermolysin vs. 10 µM:Trypsin	-1.123	-21.12 to 18.88	No	ns	>0.9999
5 µM:Thermolysin vs. 10 µM:Chymotrypsin	-40.02	-60.02 to -20.02	Yes	****	<0.0001
5 µM:Thermolysin vs. 10 µM:Thermolysin	-0.1267	-20.13 to 19.87	No	ns	>0.9999
5 µM:Thermolysin vs. 10 µM:Proteinase K	5.190	-14.81 to 25.19	No	ns	0.9997
5 µM:Thermolysin vs. 20 µM:Trypsin	7.617	-12.38 to 27.62	No	ns	0.9849
5 µM:Thermolysin vs. 20 µM:Chymotrypsin	-65.72	-85.72 to -45.72	Yes	****	<0.0001
5 µM:Thermolysin vs. 20 µM:Thermolysin	-9.147	-29.15 to 10.85	No	ns	0.9330
5 µM:Thermolysin vs. 20 µM:Proteinase K	-3.913	-23.91 to 16.09	No	ns	>0.9999
5 µM:Proteinase K vs. 10 µM:Trypsin	1.340	-18.66 to 21.34	No	ns	>0.9999
5 µM:Proteinase K vs. 10 µM:Chymotrypsin	-37.56	-57.56 to -17.56	Yes	****	<0.0001
5 µM:Proteinase K vs. 10 µM:Thermolysin	2.337	-17.66 to 22.34	No	ns	>0.9999
5 µM:Proteinase K vs. 10 µM:Proteinase K	7.653	-12.35 to 27.65	No	ns	0.9842
5 µM:Proteinase K vs. 20 µM:Trypsin	10.08	-9.918 to 30.08	No	ns	0.8711
5 µM:Proteinase K vs. 20 µM:Chymotrypsin	-63.25	-83.25 to -43.25	Yes	****	<0.0001
5 µM:Proteinase K vs. 20 µM:Thermolysin	-6.683	-26.68 to 13.32	No	ns	0.9956
5 µM:Proteinase K vs. 20 µM:Proteinase K	-1.450	-21.45 to 18.55	No	ns	>0.9999
10 µM:Trypsin vs. 10 µM:Chymotrypsin	-38.90	-58.90 to -18.90	Yes	****	<0.0001
10 µM:Trypsin vs. 10 µM:Thermolysin	0.9967	-19.00 to 21.00	No	ns	>0.9999
10 µM:Trypsin vs. 10 µM:Proteinase K	6.313	-13.69 to 26.31	No	ns	0.9976
10 µM:Trypsin vs. 20 µM:Trypsin	8.740	-11.26 to 28.74	No	ns	0.9523

10 µM:Trypsin vs. 20 µM:Chymotrypsin	-64.59	-84.59 to -44.59	Yes	****	<0.0001
10 µM:Trypsin vs. 20 µM:Thermolysin	-8.023	-28.02 to 11.98	No	ns	0.9761
10 µM:Trypsin vs. 20 µM:Proteinase K	-2.790	-22.79 to 17.21	No	ns	>0.9999
10 µM:Chymotrypsin vs. 10 µM:Thermolysin	39.90	19.90 to 59.90	Yes	****	<0.0001
10 µM:Chymotrypsin vs. 10 µM:Proteinase K	45.21	25.21 to 65.21	Yes	****	<0.0001
10 µM:Chymotrypsin vs. 20 µM:Trypsin	47.64	27.64 to 67.64	Yes	****	<0.0001
10 µM:Chymotrypsin vs. 20 µM:Chymotrypsin	-25.69	-45.69 to -5.695	Yes	**	0.0033
10 µM:Chymotrypsin vs. 20 µM:Thermolysin	30.88	10.88 to 50.88	Yes	***	0.0002
10 µM:Chymotrypsin vs. 20 µM:Proteinase K	36.11	16.11 to 56.11	Yes	****	<0.0001
10 µM:Thermolysin vs. 10 µM:Proteinase K	5.317	-14.68 to 25.32	No	ns	0.9996
10 µM:Thermolysin vs. 20 µM:Trypsin	7.743	-12.26 to 27.74	No	ns	0.9825
10 µM:Thermolysin vs. 20 µM:Chymotrypsin	-65.59	-85.59 to -45.59	Yes	****	<0.0001
10 µM:Thermolysin vs. 20 µM:Thermolysin	-9.020	-29.02 to 10.98	No	ns	0.9395
10 µM:Thermolysin vs. 20 µM:Proteinase K	-3.787	-23.79 to 16.21	No	ns	>0.9999
10 µM:Proteinase K vs. 20 µM:Trypsin	2.427	-17.57 to 22.43	No	ns	>0.9999
10 µM:Proteinase K vs. 20 µM:Chymotrypsin	-70.91	-90.91 to -50.91	Yes	****	<0.0001
10 µM:Proteinase K vs. 20 µM:Thermolysin	-14.34	-34.34 to 5.662	No	ns	0.3971
10 µM:Proteinase K vs. 20 µM:Proteinase K	-9.103	-29.10 to 10.90	No	ns	0.9353
20 µM:Trypsin vs. 20 µM:Chymotrypsin	-73.33	-93.33 to -53.33	Yes	****	<0.0001
20 µM:Trypsin vs. 20 µM:Thermolysin	-16.76	-36.76 to 3.235	No	ns	0.1828
20 µM:Trypsin vs. 20 µM:Proteinase K	-11.53	-31.53 to 8.468	No	ns	0.7294
20 µM:Chymotrypsin vs. 20 µM:Thermolysin	56.57	36.57 to 76.57	Yes	****	<0.0001
20 µM:Chymotrypsin vs. 20 µM:Proteinase K	61.80	41.80 to 81.80	Yes	****	<0.0001
20 µM:Thermolysin vs. 20 µM:Proteinase K	5.233	-14.77 to 25.33	No	ns	0.9997

Table S15. The viability data for *P. haitanensis* hydrolysates tested on QM7 cell line at reduced tryptose phosphate broth condition (2%) by resazurin assay

**Sample (µM)**	**Control**			**PH - trypsin**			**PH - chymotrypsin**			**PH - thermolysin**			**PH - proteinase K**		
**0**	101.22	94.76	104.02	X											
**1**	x			93.68	87.36	98.11	91.93	91.36	97.43	102.30	101.52	99.50	85.97	93.38	86.29
**5**				97.68	93.95	95.08	94.2	98.89	107.70	87.16	85.90	81.72	96.74	104.84	98.73
**10**				93.48	91.66	89.16	97.35	103.30	118.66	79.08	79.42	75.98	96.82	96.81	95.21
**20**				92.59	93.57	94.06	106.27	106.70	99.97	81.67	78.85	84.81	77.60	80.88	77.20

Table S16A. One-way ANOVA statistical analysis for *P. haitanensis* hydrolysates tested on QM7 cell line at reduced tryptose phosphate broth condition (2%) by resazurin assay

**ANOVA summary**	
**F**	11.92
**P value**	<0.0001
**P value summary**	****
**Significant diff. among means (P < 0.05)?**	Yes
**R square**	0.8487

**ANOVA table**	**SS**	**DF**	**MS**	**F (DFn, DFd)**	**P value**
**Treatment (between columns)**	3552	16	222.0	F (16, 34) = 11.92	P<0.0001
**Residual (within columns)**	633.5	34	18.63		
**Total**	4186	50			

Table S16B. Dunnett's multiple comparisons test for *P. haitanensis* hydrolysates tested on QM7 cell line at reduced tryptose phosphate broth condition (2%) by resazurin assay

**Dunnett's multiple comparisons test**	**Mean Diff.**	**95.00% CI of diff.**	**Significant?**	**Summary**	**Adjusted P Value**

Control vs. Trypsin 1 µM	6.950	-3.677 to 17.58	No	ns	0.3926
Control vs. Chymotrypsin 1 µM	6.427	-4.200 to 17.05	No	ns	0.4888
Control vs. Thermolysin 1 µM	-1.103	-11.73 to 9.524	No	ns	0.9996
Control vs. Proteinase K 1 µM	11.45	0.8265 to 22.08	Yes	*	0.0286
Control vs. Trypsin 5 µM	4.430	-6.197 to 15.06	No	ns	0.8766
Control vs. Chymotrpysin 5 µM	-0.2500	-10.88 to 10.38	No	ns	>0.9999
Control vs. Thermoylsin 5 µM	15.07	4.446 to 25.70	Yes	**	0.0019
Control vs. Proteinase K 5 µM	-0.1033	-10.73 to 10.52	No	ns	>0.9999
Control vs. Trypsin 10 µM	8.567	-2.060 to 19.19	No	ns	0.1751
Control vs. Chymotrypsin 10 µM	-6.437	-17.06 to 4.190	No	ns	0.4868
Control vs. Thermolysin 10 µM	21.84	11.21 to 32.47	Yes	****	<0.0001
Control vs. Proteinase K 10 µM	3.720	-6.907 to 14.35	No	ns	0.9580
Control vs. Trypsin 20 µM	6.593	-4.034 to 17.22	No	ns	0.4570
Control vs. Chymotrypsin 20 µM	-4.313	-14.94 to 6.314	No	ns	0.8932
Control vs. Thermolysin 20 µM	18.22	7.596 to 28.85	Yes	***	0.0002
Control vs. Proteinase K 20 µM	21.45	10.83 to 32.08	Yes	****	<0.0001

Table S16C. Two-way ANOVA statistical analysis for *P. haitanensis* hydrolysates tested on QM7 cell line at reduced tryptose phosphate broth condition (2%) by resazurin assay

**Two-way ANOVA**	Ordinary			
**Alpha**	0.05			
**Source of Variation**	**% of total variation**	**P value**	**P value summary**	**Significant?**
**Interaction**	45.82	<0.0001	****	Yes
**Row (concentration) Factor**	5.460	0.0166	*	Yes
**Column (enzyme) Factor**	34.00	<0.0001	****	Yes

ANOVA table	SS	DF	MS	F (DFn, DFd)	P value
**Interaction**	1834	9	203.7	F (9, 32) = 11.07	P<0.0001
**Row Factor**	218.5	3	72.83	F (3, 32) = 3.958	P=0.0166
**Column Factor**	1360	3	453.5	F (3, 32) = 24.65	P<0.0001
**Residual**	588.8	32	18.40		

Table S16D. Tukey's multiple comparisons test for *P. haitanensis* hydrolysates tested on QM7 cell line at reduced tryptose phosphate broth condition (2%) by resazurin assay

Tukey's multiple comparisons test	Mean Diff.	95.00% CI of diff.	Significant?	Summary	Adjusted P Value
1 µM:Trypsin vs. 1 µM:Chymotrypsin	-0.5233	-13.51 to 12.46	No	ns	>0.9999
1 µM:Trypsin vs. 1 µM:Thermolysin	-8.057	-21.04 to 4.931	No	ns	0.6261
1 µM:Trypsin vs. 1 µM:Proteinase K	4.503	-8.484 to 17.49	No	ns	0.9937
1 µM:Trypsin vs. 5 µM:Trypsin	-2.520	-15.51 to 10.47	No	ns	>0.9999
1 µM:Trypsin vs. 5 µM:Chymotrypsin	-7.213	-20.20 to 5.774	No	ns	0.7755
1 µM:Trypsin vs. 5 µM:Thermolysin	8.123	-4.864 to 21.11	No	ns	0.6136
1 µM:Trypsin vs. 5 µM:Proteinase K	-7.053	-20.04 to 5.934	No	ns	0.8008
1 µM:Trypsin vs. 10 µM:Trypsin	1.617	-11.37 to 14.60	No	ns	>0.9999
1 µM:Trypsin vs. 10 µM:Chymotrypsin	-13.39	-26.37 to -0.3994	Yes	*	0.0381
1 µM:Trypsin vs. 10 µM:Thermolysin	14.89	1.903 to 27.88	Yes	*	0.0130
1 µM:Trypsin vs. 10 µM:Proteinase K	-3.230	-16.22 to 9.757	No	ns	0.9998
1 µM:Trypsin vs. 20 µM:Trypsin	-0.3567	-13.34 to 12.63	No	ns	>0.9999
1 µM:Trypsin vs. 20 µM:Chymotrypsin	-11.26	-24.25 to 1.724	No	ns	0.1477
1 µM:Trypsin vs. 20 µM:Thermolysin	11.27	-1.714 to 24.26	No	ns	0.1468
1 µM:Trypsin vs. 20 µM:Proteinase K	14.49	1.503 to 27.48	Yes	*	0.0174
1 µM:Chymotrypsin vs. 1 µM:Thermolysin	-7.533	-20.52 to 5.454	No	ns	0.7215

1 µM:Chymotrypsin vs. 1 µM:Proteinase K	5.027	-7.961 to 18.01	No	ns	0.9825
1 µM:Chymotrypsin vs. 5 µM:Trypsin	-1.997	-14.98 to 10.99	No	ns	>0.9999
1 µM:Chymotrypsin vs. 5 µM:Chymotrypsin	-6.690	-19.68 to 6.297	No	ns	0.8528
1 µM:Chymotrypsin vs. 5 µM:Thermolysin	8.647	-4.341 to 21.63	No	ns	0.5154
1 µM:Chymotrypsin vs. 5 µM:Proteinase K	-6.530	-19.52 to 6.457	No	ns	0.8731
1 µM:Chymotrypsin vs. 10 µM:Trypsin	2.140	-10.85 to 15.13	No	ns	>0.9999
1 µM:Chymotrypsin vs. 10 µM:Chymotrypsin	-12.86	-25.85 to 0.1239	No	ns	0.0543
1 µM:Chymotrypsin vs. 10 µM:Thermolysin	15.41	2.426 to 28.40	Yes	**	0.0088
1 µM:Chymotrypsin vs. 10 µM:Proteinase K	-2.707	-15.69 to 10.28	No	ns	>0.9999
1 µM:Chymotrypsin vs. 20 µM:Trypsin	0.1667	-12.82 to 13.15	No	ns	>0.9999
1 µM:Chymotrypsin vs. 20 µM:Chymotrypsin	-10.74	-23.73 to 2.247	No	ns	0.1981
1 µM:Chymotrypsin vs. 20 µM:Thermolysin	11.80	-1.191 to 24.78	No	ns	0.1075
1 µM:Chymotrypsin vs. 20 µM:Proteinase K	15.01	2.026 to 28.00	Yes	*	0.0119
1 µM:Thermolysin vs. 1 µM:Proteinase K	12.56	-0.4272 to 25.55	No	ns	0.0663
1 µM:Thermolysin vs. 5 µM:Trypsin	5.537	-7.451 to 18.52	No	ns	0.9607
1 µM:Thermolysin vs. 5 µM:Chymotrypsin	0.8433	-12.14 to 13.83	No	ns	>0.9999
1 µM:Thermolysin vs. 5 µM:Thermolysin	16.18	3.193 to 29.17	Yes	**	0.0049
1 µM:Thermolysin vs. 5 µM:Proteinase K	1.003	-11.98 to 13.99	No	ns	>0.9999
1 µM:Thermolysin vs. 10 µM:Trypsin	9.673	-3.314 to 22.66	No	ns	0.3383
1 µM:Thermolysin vs. 10 µM:Chymotrypsin	-5.330	-18.32 to 7.657	No	ns	0.9711
1 µM:Thermolysin vs. 10 µM:Thermolysin	22.95	9.959 to 35.93	Yes	****	<0.0001
1 µM:Thermolysin vs. 10 µM:Proteinase K	4.827	-8.161 to 17.81	No	ns	0.9879
1 µM:Thermolysin vs. 20 µM:Trypsin	7.700	-5.287 to 20.69	No	ns	0.6918
1 µM:Thermolysin vs. 20 µM:Chymotrypsin	-3.207	-16.19 to 9.781	No	ns	0.9998
1 µM:Thermolysin vs. 20 µM:Thermolysin	19.33	6.343 to 32.32	Yes	***	0.0004

1 µM:Thermolysin vs. 20 µM:Proteinase K	22.55	9.559 to 35.53	Yes	****	<0.0001
1 µM:Proteinase K vs. 5 µM:Trypsin	-7.023	-20.01 to 5.964	No	ns	0.8054
1 µM:Proteinase K vs. 5 µM:Chymotrypsin	-11.72	-24.70 to 1.271	No	ns	0.1129
1 µM:Proteinase K vs. 5 µM:Thermolysin	3.620	-9.367 to 16.61	No	ns	0.9994
1 µM:Proteinase K vs. 5 µM:Proteinase K	-11.56	-24.54 to 1.431	No	ns	0.1243
1 µM:Proteinase K vs. 10 µM:Trypsin	-2.887	-15.87 to 10.10	No	ns	>0.9999
1 µM:Proteinase K vs. 10 µM:Chymotrypsin	-17.89	-30.88 to -4.903	Yes	**	0.0013
1 µM:Proteinase K vs. 10 µM:Thermolysin	10.39	-2.601 to 23.37	No	ns	0.2389
1 µM:Proteinase K vs. 10 µM:Proteinase K	-7.733	-20.72 to 5.254	No	ns	0.6858
1 µM:Proteinase K vs. 20 µM:Trypsin	-4.860	-17.85 to 8.127	No	ns	0.9871
1 µM:Proteinase K vs. 20 µM:Chymotrypsin	-15.77	-28.75 to -2.779	Yes	**	0.0067
1 µM:Proteinase K vs. 20 µM:Thermolysin	6.770	-6.217 to 19.76	No	ns	0.8420
1 µM:Proteinase K vs. 20 µM:Proteinase K	9.987	-3.001 to 22.97	No	ns	0.2918
5 µM:Trypsin vs. 5 µM:Chymotrypsin	-4.693	-17.68 to 8.294	No	ns	0.9907
5 µM:Trypsin vs. 5 µM:Thermolysin	10.64	-2.344 to 23.63	No	ns	0.2087
5 µM:Trypsin vs. 5 µM:Proteinase K	-4.533	-17.52 to 8.454	No	ns	0.9933
5 µM:Trypsin vs. 10 µM:Trypsin	4.137	-8.851 to 17.12	No	ns	0.9973
5 µM:Trypsin vs. 10 µM:Chymotrypsin	-10.87	-23.85 to 2.121	No	ns	0.1848
5 µM:Trypsin vs. 10 µM:Thermolysin	17.41	4.423 to 30.40	Yes	**	0.0019
5 µM:Trypsin vs. 10 µM:Proteinase K	-0.7100	-13.70 to 12.28	No	ns	>0.9999
5 µM:Trypsin vs. 20 µM:Trypsin	2.163	-10.82 to 15.15	No	ns	>0.9999
5 µM:Trypsin vs. 20 µM:Chymotrypsin	-8.743	-21.73 to 4.244	No	ns	0.4975
5 µM:Trypsin vs. 20 µM:Thermolysin	13.79	0.8061 to 26.78	Yes	*	0.0287
5 µM:Trypsin vs. 20 µM:Proteinase K	17.01	4.023 to 30.00	Yes	**	0.0026
5 µM:Chymotrypsin vs. 5 µM:Thermolysin	15.34	2.349 to 28.32	Yes	**	0.0093

5 µM:Chymotrypsin vs. 5 µM:Proteinase K	0.1600	-12.83 to 13.15	No	ns	>0.9999
5 µM:Chymotrypsin vs. 10 µM:Trypsin	8.830	-4.157 to 21.82	No	ns	0.4816
5 µM:Chymotrypsin vs. 10 µM:Chymotrypsin	-6.173	-19.16 to 6.814	No	ns	0.9122
5 µM:Chymotrypsin vs. 10 µM:Thermolysin	22.10	9.116 to 35.09	Yes	****	<0.0001
5 µM:Chymotrypsin vs. 10 µM:Proteinase K	3.983	-9.004 to 16.97	No	ns	0.9982
5 µM:Chymotrypsin vs. 20 µM:Trypsin	6.857	-6.131 to 19.84	No	ns	0.8299
5 µM:Chymotrypsin vs. 20 µM:Chymotrypsin	-4.050	-17.04 to 8.937	No	ns	0.9979
5 µM:Chymotrypsin vs. 20 µM:Thermolysin	18.49	5.499 to 31.47	Yes	***	0.0008
5 µM:Chymotrypsin vs. 20 µM:Proteinase K	21.70	8.716 to 34.69	Yes	****	<0.0001
5 µM:Thermolysin vs. 5 µM:Proteinase K	-15.18	-28.16 to -2.189	Yes	*	0.0105
5 µM:Thermolysin vs. 10 µM:Trypsin	-6.507	-19.49 to 6.481	No	ns	0.8759
5 µM:Thermolysin vs. 10 µM:Chymotrypsin	-21.51	-34.50 to -8.523	Yes	****	<0.0001
5 µM:Thermolysin vs. 10 µM:Thermolysin	6.767	-6.221 to 19.75	No	ns	0.8425
5 µM:Thermolysin vs. 10 µM:Proteinase K	-11.35	-24.34 to 1.634	No	ns	0.1401
5 µM:Thermolysin vs. 20 µM:Trypsin	-8.480	-21.47 to 4.507	No	ns	0.5465
5 µM:Thermolysin vs. 20 µM:Chymotrypsin	-19.39	-32.37 to -6.399	Yes	***	0.0004
5 µM:Thermolysin vs. 20 µM:Thermolysin	3.150	-9.837 to 16.14	No	ns	0.9999
5 µM:Thermolysin vs. 20 µM:Proteinase K	6.367	-6.621 to 19.35	No	ns	0.8921
5 µM:Proteinase K vs. 10 µM:Trypsin	8.670	-4.317 to 21.66	No	ns	0.5111
5 µM:Proteinase K vs. 10 µM:Chymotrypsin	-6.333	-19.32 to 6.654	No	ns	0.8957
5 µM:Proteinase K vs. 10 µM:Thermolysin	21.94	8.956 to 34.93	Yes	****	<0.0001
5 µM:Proteinase K vs. 10 µM:Proteinase K	3.823	-9.164 to 16.81	No	ns	0.9989
5 µM:Proteinase K vs. 20 µM:Trypsin	6.697	-6.291 to 19.68	No	ns	0.8519
5 µM:Proteinase K vs. 20 µM:Chymotrypsin	-4.210	-17.20 to 8.777	No	ns	0.9968
5 µM:Proteinase K vs. 20 µM:Thermolysin	18.33	5.339 to 31.31	Yes	***	0.0009

5 µM:Proteinase K vs. 20 µM:Proteinase K	21.54	8.556 to 34.53	Yes	****	<0.0001
10 µM:Trypsin vs. 10 µM:Chymotrypsin	-15.00	-27.99 to -2.016	Yes	*	0.0120
10 µM:Trypsin vs. 10 µM:Thermolysin	13.27	0.2861 to 26.26	Yes	*	0.0412
10 µM:Trypsin vs. 10 µM:Proteinase K	-4.847	-17.83 to 8.141	No	ns	0.9874
10 µM:Trypsin vs. 20 µM:Trypsin	-1.973	-14.96 to 11.01	No	ns	>0.9999
10 µM:Trypsin vs. 20 µM:Chymotrypsin	-12.88	-25.87 to 0.1072	No	ns	0.0537
10 µM:Trypsin vs. 20 µM:Thermolysin	9.657	-3.331 to 22.64	No	ns	0.3409
10 µM:Trypsin vs. 20 µM:Proteinase K	12.87	-0.1139 to 25.86	No	ns	0.0540
10 µM:Chymotrypsin vs. 10 µM:Thermolysin	28.28	15.29 to 41.26	Yes	****	<0.0001
10 µM:Chymotrypsin vs. 10 µM:Proteinase K	10.16	-2.831 to 23.14	No	ns	0.2685
10 µM:Chymotrypsin vs. 20 µM:Trypsin	13.03	0.04276 to 26.02	Yes	*	0.0486
10 µM:Chymotrypsin vs. 20 µM:Chymotrypsin	2.123	-10.86 to 15.11	No	ns	>0.9999
10 µM:Chymotrypsin vs. 20 µM:Thermolysin	24.66	11.67 to 37.65	Yes	****	<0.0001
10 µM:Chymotrypsin vs. 20 µM:Proteinase K	27.88	14.89 to 40.86	Yes	****	<0.0001
10 µM:Thermolysin vs. 10 µM:Proteinase K	-18.12	-31.11 to -5.133	Yes	**	0.0011
10 µM:Thermolysin vs. 20 µM:Trypsin	-15.25	-28.23 to -2.259	Yes	**	0.0100
10 µM:Thermolysin vs. 20 µM:Chymotrypsin	-26.15	-39.14 to -13.17	Yes	****	<0.0001
10 µM:Thermolysin vs. 20 µM:Thermolysin	-3.617	-16.60 to 9.371	No	ns	0.9994
10 µM:Thermolysin vs. 20 µM:Proteinase K	-0.4000	-13.39 to 12.59	No	ns	>0.9999
10 µM:Proteinase K vs. 20 µM:Trypsin	2.873	-10.11 to 15.86	No	ns	>0.9999
10 µM:Proteinase K vs. 20 µM:Chymotrypsin	-8.033	-21.02 to 4.954	No	ns	0.6305
10 µM:Proteinase K vs. 20 µM:Thermolysin	14.50	1.516 to 27.49	Yes	*	0.0173
10 µM:Proteinase K vs. 20 µM:Proteinase K	17.72	4.733 to 30.71	Yes	**	0.0015
20 µM:Trypsin vs. 20 µM:Chymotrypsin	-10.91	-23.89 to 2.081	No	ns	0.1808
20 µM:Trypsin vs. 20 µM:Thermolysin	11.63	-1.357 to 24.62	No	ns	0.1189

20 µM:Trypsin vs. 20 µM:Proteinase K	14.85	1.859 to 27.83	Yes	*	0.0134
20 µM:Chymotrypsin vs. 20 µM:Thermolysin	22.54	9.549 to 35.52	Yes	****	<0.0001
20 µM:Chymotrypsin vs. 20 µM:Proteinase K	25.75	12.77 to 38.74	Yes	****	<0.0001
20 µM:Thermolysin vs. 20 µM:Proteinase K	3.217	-9.771 to 16.20	No	ns	0.9998

Table S17. The viability data for pre-selected *P. haitanensis* hydrolysates tested on CHO-K1 cell line by neutral red assay

**Sample (µM)**	**Control**			**PH - pepsin**			**PH - chymotrypsin**		
**0**	90.95	109.39	99.65	x					
**1**	x			77.23	95.59	93.29	48.59	80.15	42.15
**5**				95.05	97.41	103.70	91.86	82.17	71.58
**10**				107.18	94.92	81.29	100.78	92.06	83.21
**20**				75.00	80.49	101.10	98.20	93.38	88.48

Table S18A. One-way ANOVA statistical analysis for pre-selected *P. haitanensis* hydrolysates tested on CHO-K1 cell line by neutral red assay

**ANOVA summary**					
**F**	3.872				
**P value**	0.0081				
**P value summary**	**				
**Significant diff. among means (P < 0.05)?**	Yes				
**R square**	0.6324				
**ANOVA table**	**SS**	**DF**	**MS**	**F (DFn, DFd)**	**P value**
**Treatment (between columns)**	4059	8	507.3	F (8, 18) = 3.872	P=0.0081
**Residual (within columns)**	2359	18	131.0		
**Total**	6417	26			

Table S18B. Dunnett's multiple comparisons test for pre-selected *P. haitanensis* hydrolysates tested on CHO-K1 cell line by neutral red assay

Dunnett's multiple comparisons test	Mean Diff.	95.00% CI of diff.	Significant?	Summary	Adjusted P Value
Control vs. PH - pepsin 1 µM	11.30	-16.14 to 38.73	No	ns	0.7565
Control vs. PH - chymotrypsin 10 µM	43.04	15.60 to 70.47	Yes	**	0.0015
Control vs. PH - pepsin 5 µM	1.280	-26.16 to 28.72	No	ns	0.9998
Control vs. PH - chymotrypsin 5 µM	18.13	-9.304 to 45.57	No	ns	0.3040
Control vs. PH - pepsin 10 µM	5.539	-21.90 to 32.97	No	ns	0.9918
Control vs. PH - chymotrypsin 10 µM	7.983	-19.45 to 35.42	No	ns	0.9392
Control vs. PH - pepsin 20 µM	14.47	-12.96 to 41.91	No	ns	0.5279
Control vs. PH - chymotrypsin 20 µM	6.650	-20.79 to 34.09	No	ns	0.9761

Table S18C. Two-way ANOVA statistical analysis for pre-selected *P. haitanensis* hydrolysates tested on CHO-K1 cell line by neutral red assay

**Two-way ANOVA**	Ordinary				
**Alpha**	0.05				
**Source of Variation**	**% of total variation**	**P value**	**P value summary**	**Significant?**	
**Interaction**	23.23	0.0491	*	Yes	
**Row (concentration) Factor**	26.60	0.0330	*	Yes	
**Column (enzyme) Factor**	12.16	0.0379	*	Yes	
**ANOVA table**	**SS**	**DF**	**MS**	**F (DFn, DFd)**	**P value**
**Interaction**	1337	3	445.8	F (3, 16) = 3.259	P=0.0491
**Row Factor**	1531	3	510.5	F (3, 16) = 3.732	P=0.0330
**Column Factor**	700.2	1	700.2	F (1, 16) = 5.119	P=0.0379
**Residual**	2189	16	136.8		

Table S18D. Tukey's multiple comparisons test for pre-selected *P. haitanensis* hydrolysates tested on CHO-K1 cell line by neutral red assay

Tukey's multiple comparisons test	Mean Diff.	95.00% CI of diff.	Significant?	Summary	Adjusted P Value
1 µM:PH - pepsin vs. 1 µM:PH - chymotrypsin	31.74	-1.323 to 64.80	No	ns	0.0647
1 µM:PH - pepsin vs. 5 µM:PH - pepsin	-10.02	-43.08 to 23.04	No	ns	0.9587
1 µM:PH - pepsin vs. 5 µM:PH - chymotrypsin	6.833	-26.23 to 39.89	No	ns	0.9952
1 µM:PH - pepsin vs. 10 µM:PH - pepsin	-5.759	-38.82 to 27.30	No	ns	0.9983
1 µM:PH - pepsin vs. 10 µM:PH - chymotrypsin	-3.316	-36.38 to 29.75	No	ns	>0.9999
1 µM:PH - pepsin vs. 20 µM:PH - pepsin	3.174	-29.89 to 36.24	No	ns	>0.9999
1 µM:PH - pepsin vs. 20 µM:PH - chymotrypsin	-4.648	-37.71 to 28.41	No	ns	0.9996
1 µM:PH - chymotrypsin vs. 5 µM:PH - pepsin	-41.76	-74.82 to -8.696	Yes	**	0.0087
1 µM:PH - chymotrypsin vs. 5 µM:PH - chymotrypsin	-24.91	-57.97 to 8.155	No	ns	0.2221
1 µM:PH - chymotrypsin vs. 10 µM:PH - pepsin	-37.50	-70.56 to -4.437	Yes	*	0.0206
1 µM:PH - chymotrypsin vs. 10 µM:PH - chymotrypsin	-35.05	-68.12 to -1.993	Yes	*	0.0337
1 µM:PH - chymotrypsin vs. 20 µM:PH - pepsin	-28.56	-61.63 to 4.497	No	ns	0.1175
1 µM:PH - chymotrypsin vs. 20 µM:PH - chymotrypsin	-36.39	-69.45 to -3.325	Yes	*	0.0258
5 µM:PH - pepsin vs. 5 µM:PH - chymotrypsin	16.85	-16.21 to 49.91	No	ns	0.6497
5 µM:PH - pepsin vs. 10 µM:PH - pepsin	4.260	-28.80 to 37.32	No	ns	0.9998
5 µM:PH - pepsin vs. 10 µM:PH - chymotrypsin	6.703	-26.36 to 39.76	No	ns	0.9957
5 µM:PH - pepsin vs. 20 µM:PH - pepsin	13.19	-19.87 to 46.25	No	ns	0.8528
5 µM:PH - pepsin vs. 20 µM:PH - chymotrypsin	5.371	-27.69 to 38.43	No	ns	0.9989
5 µM:PH - chymotrypsin vs. 10 µM:PH - pepsin	-12.59	-45.65 to 20.47	No	ns	0.8789
5 µM:PH - chymotrypsin vs. 10 µM:PH - chymotrypsin	-10.15	-43.21 to 22.91	No	ns	0.9559
5 µM:PH - chymotrypsin vs. 20 µM:PH - pepsin	-3.659	-36.72 to 29.40	No	ns	>0.9999
5 µM:PH - chymotrypsin vs. 20 µM:PH - chymotrypsin	-11.48	-44.54 to 21.58	No	ns	0.9198
10 µM:PH - pepsin vs. 10 µM:PH - chymotrypsin	2.444	-30.62 to 35.50	No	ns	>0.9999
10 µM:PH - pepsin vs. 20 µM:PH - pepsin	8.933	-24.13 to 41.99	No	ns	0.9774

10 µM:PH - pepsin vs. 20 µM:PH - chymotrypsin	1.111	-31.95 to 34.17	No	ns	>0.9999
10 µM:PH - chymotrypsin vs. 20 µM:PH - pepsin	6.490	-26.57 to 39.55	No	ns	0.9965
10 µM:PH - chymotrypsin vs. 20 µM:PH - chymotrypsin	-1.332	-34.39 to 31.73	No	ns	>0.9999
20 µM:PH - pepsin vs. 20 µM:PH - chymotrypsin	-7.822	-40.88 to 25.24	No	ns	0.9893

Table S19. The viability data for pre-selected *A. platensis* and *P. haitanensis* hydrolysates tested on QM7 cell line by neutral red assay

Sample (µM)	Control			AP - chymotrypsin			PH - proteinase K		
0	96.07	100.03	103.90	x					
1	x			77.13	94.25	108.67	89.35	95.10	104.63
5				87.47	105.65	119.51	59.10	57.35	56.85
10				116.10	119.59	123.26	33.54	28.88	29.88
20				107.65	81.10	75.51	0.29	-2.28	0.55

Table S20A. One-way ANOVA statistical analysis for pre-selected *A. platensis* and *P. haitanensis* hydrolysates tested on QM7 cell line by neutral red assay

**ANOVA summary**					
**F**	46.37				
**P value**	<0.0001				
**P value summary**	****				
**Significant diff. among means (P < 0.05)?**	Yes				
**R square**	0.9537				
**ANOVA table**	**SS**	**DF**	**MS**	**F (DFn, DFd)**	**P value**
**Treatment (between columns)**	37088	8	4636	F (8, 18) = 46.37	P<0.0001
**Residual (within columns)**	1800	18	99.98		
**Total**	38887	26			

Table S20B. Dunnett's multiple comparisons test for pre-selected *A. platensis* and *P. haitanensis* hydrolysates tested on QM7 cell line by neutral red assay

Dunnett's multiple comparisons test	Mean Diff.	95.00% CI of diff.	Significant?	Summary	Adjusted P Value
Control vs. AP - chymotrypsin 1 µM	6.647	-17.32 to 30.61	No	ns	0.9518
Control vs. PH - proteinase K 1 µM	3.640	-20.32 to 27.60	No	ns	0.9976
Control vs. AP - chymotrypsin 5 µM	-4.208	-28.17 to 19.76	No	ns	0.9952
Control vs. PH - proteinase K 5 µM	42.23	18.27 to 66.20	Yes	***	0.0005
Control vs. AP - chymotrpysin 10 µM	-19.65	-43.61 to 4.316	No	ns	0.1378
Control vs. PH - proteinase K 10 µM	69.23	45.27 to 93.20	Yes	****	<0.0001
Control vs. AP - chymotrypsin 20 µM	11.91	-12.05 to 35.88	No	ns	0.5875
Control vs. PH - proteinase K 20 µM	100.5	76.52 to 124.4	Yes	****	<0.0001

Table S20C. Two-way ANOVA statistical analysis for pre-selected *A. platensis* and *P. haitanensis* hydrolysates tested on QM7 cell line by neutral red assay

**Two-way ANOVA**	Ordinary				
**Alpha**	0.05				
**Source of Variation**	**% of total variation**	**P value**	**P value summary**	**Significant?**	
**Interaction**	23.15	<0.0001	****	Yes	
**Row (concentration) Factor**	22.64	<0.0001	****	Yes	
**Column (enzyme) Factor**	49.43	<0.0001	****	Yes	
**ANOVA table**	**SS**	**DF**	**MS**	**F (DFn, DFd)**	**P value**
**Interaction**	8569	3	2856	F (3, 16) = 25.83	P<0.0001
**Row Factor**	8380	3	2793	F (3, 16) = 25.27	P<0.0001
**Column Factor**	18296	1	18296	F (1, 16) = 165.5	P<0.0001
**Residual**	1769	16	110.6		

Table S20D. Tukey's multiple comparisons test for pre-selected *A. platensis* and *P. haitanensis* hydrolysates tested on QM7 cell line by neutral red assay

Tukey's multiple comparisons test	Mean Diff.	95.00% CI of diff.	Significant?	Summary	Adjusted P Value
1 µM:AP - chymotrypsin vs. 1 µM:PH - proteinase K	-3.007	-32.73 to 26.72	No	ns	>0.9999
1 µM:AP - chymotrypsin vs. 5 µM:AP - chymotrypsin	-10.86	-40.58 to 18.87	No	ns	0.8992
1 µM:AP - chymotrypsin vs. 5 µM:PH - proteinase K	35.59	5.863 to 65.31	Yes	*	0.0135
1 µM:AP - chymotrypsin vs. 10 µM:AP - chymotrypsin	-26.30	-56.02 to 3.428	No	ns	0.1036
1 µM:AP - chymotrypsin vs. 10 µM:PH - proteinase K	62.58	32.86 to 92.31	Yes	****	<0.0001
1 µM:AP - chymotrypsin vs. 20 µM:AP - chymotrypsin	5.266	-24.46 to 34.99	No	ns	0.9981
1 µM:AP - chymotrypsin vs. 20 µM:PH - proteinase K	93.83	64.11 to 123.6	Yes	****	<0.0001
1 µM:PH - proteinase K vs. 5 µM:AP - chymotrypsin	-7.849	-37.57 to 21.88	No	ns	0.9801
1 µM:PH - proteinase K vs. 5 µM:PH - proteinase K	38.59	8.870 to 68.32	Yes	**	0.0068
1 µM:PH - proteinase K vs. 10 µM:AP - chymotrypsin	-23.29	-53.01 to 6.435	No	ns	0.1880
1 µM:PH - proteinase K vs. 10 µM:PH - proteinase K	65.59	35.87 to 95.32	Yes	****	<0.0001
1 µM:PH - proteinase K vs. 20 µM:AP - chymotrypsin	8.273	-21.45 to 38.00	No	ns	0.9736
1 µM:PH - proteinase K vs. 20 µM:PH - proteinase K	96.84	67.12 to 126.6	Yes	****	<0.0001
5 µM:AP - chymotrypsin vs. 5 µM:PH - proteinase K	46.44	16.72 to 76.17	Yes	**	0.0012
5 µM:AP - chymotrypsin vs. 10 µM:AP - chymotrypsin	-15.44	-45.16 to 14.28	No	ns	0.6297
5 µM:AP - chymotrypsin vs. 10 µM:PH - proteinase K	73.44	43.72 to 103.2	Yes	****	<0.0001
5 µM:AP - chymotrypsin vs. 20 µM:AP - chymotrypsin	16.12	-13.60 to 45.85	No	ns	0.5826
5 µM:AP - chymotrypsin vs. 20 µM:PH - proteinase K	104.7	74.96 to 134.4	Yes	****	<0.0001
5 µM:PH - proteinase K vs. 10 µM:AP - chymotrypsin	-61.88	-91.61 to -32.16	Yes	****	<0.0001
5 µM:PH - proteinase K vs. 10 µM:PH - proteinase K	27.00	-2.727 to 56.72	No	ns	0.0896
5 µM:PH - proteinase K vs. 20 µM:AP - chymotrypsin	-30.32	-60.05 to -0.5976	Yes	*	0.0439
5 µM:PH - proteinase K vs. 20 µM:PH - proteinase K	58.25	28.52 to 87.97	Yes	****	<0.0001
10 µM:AP - chymotrypsin vs. 10 µM:PH - proteinase K	88.88	59.16 to 118.6	Yes	****	<0.0001
10 µM:AP - chymotrypsin vs. 20 µM:AP - chymotrypsin	31.56	1.838 to 61.29	Yes	*	0.0334

10 µM:AP - chymotrypsin vs. 20 µM:PH - proteinase K	120.1	90.40 to 149.9	Yes	****	<0.0001
10 µM:PH - proteinase K vs. 20 µM:AP - chymotrypsin	-57.32	-87.04 to -27.59	Yes	***	0.0001
10 µM:PH - proteinase K vs. 20 µM:PH - proteinase K	31.25	1.524 to 60.97	Yes	*	0.0358
20 µM:AP - chymotrypsin vs. 20 µM:PH - proteinase K	88.57	58.84 to 118.3	Yes	****	<0.0001

Table S21. The viability data for pre-selected *A. platensis* and *P. haitanensis* hydrolysates tested on MACK1 cell line by neutral red assay

**Sample (µM)**	**Control**			**AP - pepsin**			**PH - proteinase K**		
**0**	95.58	106.18	98.24	x					
**1**	x			109.77	113.65	118.90	107.55	115.97	115.22
**5**				114.34	117.16	121.19	105.96	90.69	87.42
**10**				140.24	126.39	129.16	67.48	86.06	67.16
**20**				70.67	56.00	68.92	-1.68	-2.57	0.92

Table S22A. One-way ANOVA statistical analysis for pre-selected *A. platensis* and *P. haitanensis* hydrolysates tested on MACK1 cell line by neutral red assay

**ANOVA summary**	
**F**	115.8
**P value**	<0.0001
**P value summary**	****
**Significant diff. among means (P < 0.05)?**	Yes
**R square**	0.9820

**ANOVA table**	**SS**	**DF**	**MS**	**F (DFn, DFd)**	**P value**
**Treatment (between columns)**	38772	8	4847	F (8, 17) = 115.8	P<0.0001
**Residual (within columns)**	711.7	17	41.86		
**Total**	39484	25			

Table S22B. Dunnett's multiple comparisons test for pre-selected *A. platensis* and *P. haitanensis* hydrolysates tested on MACK1 cell line by neutral red assay

**Dunnett's multiple comparisons test**	**Mean Diff.**	**95.00% CI of diff.**	**Significant?**	**Summary**	**Adjusted P Value**
Control vs. AP - pepsin 1 µM	-14.10	-29.74 to 1.531	No	ns	0.0875
Control vs. PH - proteinase K 1 µM	-12.92	-28.55 to 2.719	No	ns	0.1326
Control vs. AP - pepsin 5 µM	-17.56	-33.20 to -1.926	Yes	*	0.0241
Control vs. PH - proteinase K 5 µM	5.309	-12.17 to 22.79	No	ns	0.9251
Control vs. AP - pepsin 10 µM	-31.93	-47.57 to -16.29	Yes	***	0.0001
Control vs. PH - proteinase K 10 µM	26.43	10.80 to 42.07	Yes	***	0.0007
Control vs. AP - pepsin 20 µM	34.80	19.17 to 50.44	Yes	****	<0.0001
Control vs. PH - proteinase K 20 µM	101.1	85.47 to 116.7	Yes	****	<0.0001

Table S22C. Two-way ANOVA statistical analysis for pre-selected *A. platensis* and *P. haitanensis* hydrolysates tested on MACK1 cell line by neutral red assay

Two-way ANOVA	Ordinary				
**Alpha**	0.05				
**Source of Variation**	% of total variation	P value	P value summary	Significant?	
**Interaction**	10.69	<0.0001	****	Yes	
**Row (concentration) Factor**	66.17	<0.0001	****	Yes	
**Column (enzyme) Factor**	21.15	<0.0001	****	Yes	
**ANOVA table**	**SS**	**DF**	**MS**	**F (DFn, DFd)**	**P value**
**Interaction**	4195	3	1398	F (3, 16) = 28.63	P<0.0001
**Row Factor**	25959	3	8653	F (3, 16) = 177.1	P<0.0001
**Column Factor**	8296	1	8296	F (1, 16) = 169.8	P<0.0001
**Residual**	781.6	16	48.85		

Table S22D. Tukey's multiple comparisons test for pre-selected *A. platensis* and *P. haitanensis* hydrolysates tested on MACK1 cell line by neutral red assay

Tukey's multiple comparisons test	Mean Diff.	95.00% CI of diff.	Significant?	Summary	Adjusted P Value
1 µM:AP - pepsin vs. 1 µM:PH - proteinase K	1.193	-18.56 to 20.95	No	ns	>0.9999
1 µM:AP - pepsin vs. 5 µM:AP - pepsin	-3.457	-23.21 to 16.30	No	ns	0.9983
1 µM:AP - pepsin vs. 5 µM:PH - proteinase K	19.42	-0.3404 to 39.17	No	ns	0.0559
1 µM:AP - pepsin vs. 10 µM:AP - pepsin	-17.82	-37.58 to 1.934	No	ns	0.0931
1 µM:AP - pepsin vs. 10 µM:PH - proteinase K	40.54	20.78 to 60.30	Yes	****	<0.0001
1 µM:AP - pepsin vs. 20 µM:AP - pepsin	48.91	29.15 to 68.67	Yes	****	<0.0001
1 µM:AP - pepsin vs. 20 µM:PH - proteinase K	115.2	95.46 to 135.0	Yes	****	<0.0001
1 µM:PH - proteinase K vs. 5 µM:AP - pepsin	-4.650	-24.41 to 15.11	No	ns	0.9896
1 µM:PH - proteinase K vs. 5 µM:PH - proteinase K	18.22	-1.534 to 37.98	No	ns	0.0820
1 µM:PH - proteinase K vs. 10 µM:AP - pepsin	-19.02	-38.77 to 0.7404	No	ns	0.0636
1 µM:PH - proteinase K vs. 10 µM:PH - proteinase K	39.35	19.59 to 59.10	Yes	****	<0.0001
1 µM:PH - proteinase K vs. 20 µM:AP - pepsin	47.72	27.96 to 67.47	Yes	****	<0.0001
1 µM:PH - proteinase K vs. 20 µM:PH - proteinase K	114.0	94.27 to 133.8	Yes	****	<0.0001
5 µM:AP - pepsin vs. 5 µM:PH - proteinase K	22.87	3.116 to 42.63	Yes	*	0.0176
5 µM:AP - pepsin vs. 10 µM:AP - pepsin	-14.37	-34.12 to 5.390	No	ns	0.2555
5 µM:AP - pepsin vs. 10 µM:PH - proteinase K	44.00	24.24 to 63.75	Yes	****	<0.0001
5 µM:AP - pepsin vs. 20 µM:AP - pepsin	52.37	32.61 to 72.12	Yes	****	<0.0001
5 µM:AP - pepsin vs. 20 µM:PH - proteinase K	118.7	98.92 to 138.4	Yes	****	<0.0001
5 µM:PH - proteinase K vs. 10 µM:AP - pepsin	-37.24	-57.00 to -17.48	Yes	***	0.0001
5 µM:PH - proteinase K vs. 10 µM:PH - proteinase K	21.12	1.366 to 40.88	Yes	*	0.0318
5 µM:PH - proteinase K vs. 20 µM:AP - pepsin	29.49	9.736 to 49.25	Yes	**	0.0018
5 µM:PH - proteinase K vs. 20 µM:PH - proteinase K	95.80	76.04 to 115.6	Yes	****	<0.0001
10 µM:AP - pepsin vs. 10 µM:PH - proteinase K	58.36	38.61 to 78.12	Yes	****	<0.0001
10 µM:AP - pepsin vs. 20 µM:AP - pepsin	66.73	46.98 to 86.49	Yes	****	<0.0001

10 µM:AP - pepsin vs. 20 µM:PH - proteinase K	133.0	113.3 to 152.8	Yes	****	<0.0001
10 µM:PH - proteinase K vs. 20 µM:AP - pepsin	8.370	-11.39 to 28.13	No	ns	0.8135
10 µM:PH - proteinase K vs. 20 µM:PH - proteinase K	74.68	54.92 to 94.43	Yes	****	<0.0001
20 µM:AP - pepsin vs. 20 µM:PH - proteinase K	66.31	46.55 to 86.06	Yes	****	<0.0001

Table S23. The effects of pre-selected *P. haitanensis* hydrolysates on fold change in cell number relative to day 0 of CHO-K1 cell line.

**Sample (µM)**	**Control**			**PH - pepsin**			**PH - chymotrypsin**		
**0**	59.30	49.60	61.60	x					
**1**				45.30	42.10	44.30	38.10	52.70	60.40
**5**	x			58.40	55.40	60.00	42.60	57.50	46.00
**10**				61.40	67.50	77.10	50.50	38.80	39.70
**20**				63.40	38.40	40.70	36.20	41.50	48.80

Table S24A. One-way ANOVA statistical analysis for the effects of pre-selected *P. haitanensis* hydrolysates on fold change in cell number relative to day 0 of CHO-K1 cell line.

**ANOVA summary**					
**F**	3.723				
**P value**	0.0098				
**P value summary**	**				
**Significant diff. among means (P < 0.05)?**	Yes				
**R square**	0.6233				
**ANOVA table**	**SS**	**DF**	**MS**	**F (DFn, DFd)**	**P value**
**Treatment (between columns)**	1814	8	226.7	F (8, 18) = 3.723	P=0.0098
**Residual (within columns)**	1096	18	60.89		
**Total**	2910	26			

Table S24B. Dunnett's multiple comparisons test for the effects of pre-selected *P. haitanensis* hydrolysates on fold change in cell number relative to day 0 of CHO-K1 cell line.

Dunnett's multiple comparisons test	Mean Diff.	95.00% CI of diff.	Significant?	Summary	Adjusted P Value
Control vs. PH - pepsin 1 μM	12.93	-5.769 to 31.64	No	ns	0.2635
Control vs. PH - chymotrypsin 10 μM	6.433	-12.27 to 25.14	No	ns	0.8732
Control vs. PH - pepsin 5 μM	-1.100	-19.80 to 17.60	No	ns	0.9997
Control vs. PH - chymotrypsin 5 μM	8.133	-10.57 to 26.84	No	ns	0.7119
Control vs. PH - pepsin 10 μM	-11.83	-30.54 to 6.869	No	ns	0.3450
Control vs. PH - chymotrypsin 10 μM	13.83	-4.869 to 32.54	No	ns	0.2086
Control vs. PH - pepsin 20 μM	9.333	-9.369 to 28.04	No	ns	0.5837
Control vs. PH - chymotrypsin 20 μM	14.67	-4.036 to 33.37	No	ns	0.1662

Table S24C. Two-way ANOVA statistical analysis for the effects of pre-selected *P. haitanensis* hydrolysates on fold change in cell number relative to day 0 of CHO-K1 cell line.

**Two-way ANOVA**	Ordinary				
**Alpha**	0.05				
**Source of Variation**	**% of total variation**	**P value**	**P value summary**	**Significant?**	
**Interaction**	28.76	0.0271	*	Yes	
**Row (concentration) Factor**	17.25	0.1073	ns	No	
**Column (enzyme) Factor**	15.43	0.0223	*	Yes	
**ANOVA table**	**SS**	**DF**	**MS**	**F (DFn, DFd)**	**P value**
**Interaction**	795.4	3	265.1	F (3, 16) = 3.976	P=0.0271
**Row Factor**	477.1	3	159.0	F (3, 16) = 2.386	P=0.1073
**Column Factor**	426.7	1	426.7	F (1, 16) = 6.400	P=0.0223

**Residual**	1067	16	66.67

Table S24D. Tukey's multiple comparisons test for the effects of pre-selected *P. haitanensis* hydrolysates on fold change in cell number relative to day 0 of CHO-K1 cell line.

Tukey's multiple comparisons test	Mean Diff.	95.00% CI of diff.	Significant?	Summary	Adjusted P Value
1 µM:PH - pepsin vs. 1 µM:PH - chymotrypsin	-6.500	-29.58 to 16.58	No	ns	0.9718
1 µM:PH - pepsin vs. 5 µM:PH - pepsin	-14.03	-37.12 to 9.049	No	ns	0.4513
1 µM:PH - pepsin vs. 5 µM:PH - chymotrypsin	-4.800	-27.88 to 18.28	No	ns	0.9950
1 µM:PH - pepsin vs. 10 µM:PH - pepsin	-24.77	-47.85 to -1.685	Yes	*	0.0310
1 µM:PH - pepsin vs. 10 µM:PH - chymotrypsin	0.9000	-22.18 to 23.98	No	ns	>0.9999
1 µM:PH - pepsin vs. 20 µM:PH - pepsin	-3.600	-26.68 to 19.48	No	ns	0.9992
1 µM:PH - pepsin vs. 20 µM:PH - chymotrypsin	1.733	-21.35 to 24.82	No	ns	>0.9999
1 µM:PH - chymotrypsin vs. 5 µM:PH - pepsin	-7.533	-30.62 to 15.55	No	ns	0.9403
1 µM:PH - chymotrypsin vs. 5 µM:PH - chymotrypsin	1.700	-21.38 to 24.78	No	ns	>0.9999
1 µM:PH - chymotrypsin vs. 10 µM:PH - pepsin	-18.27	-41.35 to 4.815	No	ns	0.1798
1 µM:PH - chymotrypsin vs. 10 µM:PH - chymotrypsin	7.400	-15.68 to 30.48	No	ns	0.9453
1 µM:PH - chymotrypsin vs. 20 µM:PH - pepsin	2.900	-20.18 to 25.98	No	ns	0.9998
1 µM:PH - chymotrypsin vs. 20 µM:PH - chymotrypsin	8.233	-14.85 to 31.32	No	ns	0.9093
5 µM:PH - pepsin vs. 5 µM:PH - chymotrypsin	9.233	-13.85 to 32.32	No	ns	0.8514
5 µM:PH - pepsin vs. 10 µM:PH - pepsin	-10.73	-33.82 to 12.35	No	ns	0.7386
5 µM:PH - pepsin vs. 10 µM:PH - chymotrypsin	14.93	-8.149 to 38.02	No	ns	0.3798
5 µM:PH - pepsin vs. 20 µM:PH - pepsin	10.43	-12.65 to 33.52	No	ns	0.7632
5 µM:PH - pepsin vs. 20 µM:PH - chymotrypsin	15.77	-7.315 to 38.85	No	ns	0.3198
5 µM:PH - chymotrypsin vs. 10 µM:PH - pepsin	-19.97	-43.05 to 3.115	No	ns	0.1167
5 µM:PH - chymotrypsin vs. 10 µM:PH - chymotrypsin	5.700	-17.38 to 28.78	No	ns	0.9863

5 µM:PH - chymotrypsin vs. 20 µM:PH - pepsin	1.200	-21.88 to 24.28	No	ns	>0.9999
5 µM:PH - chymotrypsin vs. 20 µM:PH - chymotrypsin	6.533	-16.55 to 29.62	No	ns	0.9711
10 µM:PH - pepsin vs. 10 µM:PH - chymotrypsin	25.67	2.585 to 48.75	Yes	*	0.0239
10 µM:PH - pepsin vs. 20 µM:PH - pepsin	21.17	-1.915 to 44.25	No	ns	0.0848
10 µM:PH - pepsin vs. 20 µM:PH - chymotrypsin	26.50	3.418 to 49.58	Yes	*	0.0188
10 µM:PH - chymotrypsin vs. 20 µM:PH - pepsin	-4.500	-27.58 to 18.58	No	ns	0.9966
10 µM:PH - chymotrypsin vs. 20 µM:PH - chymotrypsin	0.8333	-22.25 to 23.92	No	ns	>0.9999
20 µM:PH - pepsin vs. 20 µM:PH - chymotrypsin	5.333	-17.75 to 28.42	No	ns	0.9907

Table S25. The effects of pre-selected *A. platensis* and *P. haitanensis* hydrolysates on fold change in cell number relative to day 0 of QM7 cell line.

**Sample (µM)**	**Control**			**AP - chymotrypsin**			**PH - proteinase K**		
**0**	23.20	18.00	25.20	X					
**1**	x			20.00	20.20	20.40	18.00	20.60	21.40
**5**				14.80	21.20	20.00	13.80	15.00	17.20
**10**				13.80	13.20	13.20	7.00	4.60	7.60
**20**				10.00	9.70	10.10	4.20	5.60	5.80

Table S26A. One-way ANOVA statistical analysis for the effects of pre-selected *A. platensis* and *P. haitanensis* hydrolysates on fold change in cell number relative to day 0 of QM7 cell line.

**ANOVA summary**	
**F**	30.14
**P value**	<0.0001
**P value summary**	****
**Significant diff. among means (P < 0.05)?**	Yes
**R square**	0.9305

ANOVA table	SS	DF	MS	F (DFn, DFd)	P value
**Treatment (between columns)**	939.5	8	117.4	F (8, 18) = 30.14	P<0.0001
**Residual (within columns)**	70.12	18	3.896		
**Total**	1010	26			

Table S26B. Dunnett's multiple comparisons test for the effects of pre-selected *A. platensis* and *P. haitanensis* hydrolysates on fold change in cell number relative to day 0 of QM7 cell line.

Dunnett's multiple comparisons test	Mean Diff.	95.00% CI of diff.	Significant?	Summary	Adjusted P Value
Control vs. AP - chymotrypsin 1 µM	1.933	-2.797 to 6.664	No	ns	0.7624
Control vs. PH - proteinase K 1 µM	2.133	-2.597 to 6.864	No	ns	0.6799
Control vs. AP - chymotrypsin 5 µM	3.467	-1.264 to 8.197	No	ns	0.2156
Control vs. PH - proteinase K 5 µM	6.803	2.073 to 11.53	Yes	**	0.0033
Control vs. AP - chymotrpysin 10 µM	8.733	4.003 to 13.46	Yes	***	0.0003
Control vs. PH - proteinase K 10 µM	15.73	11.00 to 20.46	Yes	****	<0.0001
Control vs. AP - chymotrypsin 20 µM	12.20	7.469 to 16.93	Yes	****	<0.0001
Control vs. PH - proteinase K 20 µM	16.93	12.20 to 21.66	Yes	****	<0.0001

Table S26C. Two-way ANOVA statistical analysis for the effects of pre-selected *A. platensis* and *P. haitanensis* hydrolysates on fold change in cell number relative to day 0 of QM7 cell line.

**Two-way ANOVA**	Ordinary			
**Alpha**	0.05			
**Source of Variation**	**% of total variation**	**P value**	**P value summary**	**Significant?**
**Interaction**	4.614	0.0168	*	Yes
**Row (concentration) Factor**	78.95	<0.0001	****	Yes
**Column (enzyme) Factor**	11.07	<0.0001	****	Yes

ANOVA table	SS	DF	MS	F (DFn, DFd)	P value
**Interaction**	36.43	3	12.14	F (3, 16) = 4.585	P=0.0168
**Row Factor**	623.4	3	207.8	F (3, 16) = 78.45	P<0.0001
**Column Factor**	87.40	1	87.40	F (1, 16) = 33.00	P<0.0001
**Residual**	42.38	16	2.649		

Table S26D. Tukey's multiple comparisons test for the effects of pre-selected *A. platensis* and *P. haitanensis* hydrolysates on fold change in cell number relative to day 0 of QM7 cell line.

Tukey's multiple comparisons test	Mean Diff.	95.00% CI of diff.	Significant?	Summary	Adjusted P Value
1 µM:AP - chymotrpysin vs. 1 µM:PH - proteinase K	0.2000	-4.401 to 4.801	No	ns	>0.9999
1 µM:AP - chymotrpysin vs. 5 µM:AP - chymotrpysin	1.533	-3.067 to 6.134	No	ns	0.9339
1 µM:AP - chymotrpysin vs. 5 µM:PH - proteinase K	4.867	0.2660 to 9.467	Yes	*	0.0343
1 µM:AP - chymotrpysin vs. 10 µM:AP - chymotrpysin	6.800	2.199 to 11.40	Yes	**	0.0020
1 µM:AP - chymotrpysin vs. 10 µM:PH - proteinase K	13.80	9.199 to 18.40	Yes	****	<0.0001
1 µM:AP - chymotrpysin vs. 20 µM:AP - chymotrpysin	10.27	5.666 to 14.87	Yes	****	<0.0001
1 µM:AP - chymotrpysin vs. 20 µM:PH - proteinase K	15.00	10.40 to 19.60	Yes	****	<0.0001
1 µM:PH - proteinase K vs. 5 µM:AP - chymotrpysin	1.333	-3.267 to 5.934	No	ns	0.9672
1 µM:PH - proteinase K vs. 5 µM:PH - proteinase K	4.667	0.06600 to 9.267	Yes	*	0.0455
1 µM:PH - proteinase K vs. 10 µM:AP - chymotrpysin	6.600	1.999 to 11.20	Yes	**	0.0027
1 µM:PH - proteinase K vs. 10 µM:PH - proteinase K	13.60	8.999 to 18.20	Yes	****	<0.0001
1 µM:PH - proteinase K vs. 20 µM:AP - chymotrpysin	10.07	5.466 to 14.67	Yes	****	<0.0001
1 µM:PH - proteinase K vs. 20 µM:PH - proteinase K	14.80	10.20 to 19.40	Yes	****	<0.0001
5 µM:AP - chymotrpysin vs. 5 µM:PH - proteinase K	3.333	-1.267 to 7.934	No	ns	0.2590
5 µM:AP - chymotrpysin vs. 10 µM:AP - chymotrpysin	5.267	0.6660 to 9.867	Yes	*	0.0192
5 µM:AP - chymotrpysin vs. 10 µM:PH - proteinase K	12.27	7.666 to 16.87	Yes	****	<0.0001

5 μM:AP - chymotrpysin vs. 20 μM:AP - chymotrpysin	8.733	4.133 to 13.33	Yes	***	0.0001
5 μM:AP - chymotrpysin vs. 20 μM:PH - proteinase K	13.47	8.866 to 18.07	Yes	****	<0.0001
5 μM:PH - proteinase K vs. 10 μM:AP - chymotrpysin	1.933	-2.667 to 6.534	No	ns	0.8192
5 μM:PH - proteinase K vs. 10 μM:PH - proteinase K	8.933	4.333 to 13.53	Yes	***	0.0001
5 μM:PH - proteinase K vs. 20 μM:AP - chymotrpysin	5.400	0.7993 to 10.00	Yes	*	0.0158
5 μM:PH - proteinase K vs. 20 μM:PH - proteinase K	10.13	5.533 to 14.73	Yes	****	<0.0001
10 μM:AP - chymotrpysin vs. 10 μM:PH - proteinase K	7.000	2.399 to 11.60	Yes	**	0.0015
10 μM:AP - chymotrpysin vs. 20 μM:AP - chymotrpysin	3.467	-1.134 to 8.067	No	ns	0.2219
10 μM:AP - chymotrpysin vs. 20 μM:PH - proteinase K	8.200	3.599 to 12.80	Yes	***	0.0003
10 μM:PH - proteinase K vs. 20 μM:AP - chymotrpysin	-3.533	-8.134 to 1.067	No	ns	0.2049
10 μM:PH - proteinase K vs. 20 μM:PH - proteinase K	1.200	-3.401 to 5.801	No	ns	0.9814
20 μM:AP - chymotrpysin vs. 20 μM:PH - proteinase K	4.733	0.1327 to 9.334	Yes	*	0.0414

Table S27. The effects of pre-selected *A. platensis* and *P. haitanensis* hydrolysates on fold change in cell number relative to day 0 of MACK1 cell line.

Sample (μM)	Control			AP - pepsin			PH - proteinase K		
0	4.05	4.05	3.20	X					
1	x			4.25	4.50	6.20	2.80	3.30	3.20
5				4.20	4.10	3.80	3.20	1.80	2.10
10				4.25	2.90	3.50	0.70	0.70	0.50
20				3.55	2.90	2.90	3.00	0.00	0.00

Table S28A. One-way ANOVA statistical analysis for the effects of pre-selected *A. platensis* and *P. haitanensis* hydrolysates on fold change in cell number relative to day 0 of MACK1 cell line.

**ANOVA summary**	
**F**	25.40

**P value**	<0.0001
**P value summary**	****
**Significant diff. among means (P < 0.05)?**	Yes
**R square**	0.9186

**ANOVA table**	**SS**	**DF**	**MS**	**F (DFn, DFd)**	**P value**
**Treatment (between columns)**	60.41	8	7.551	F (8, 18) = 25.40	P<0.0001
**Residual (within columns)**	5.350	18	0.2972		
**Total**	65.76	26			

Table S28B. Dunnett's multiple comparisons test for the effects of pre-selected *A. platensis* and *P. haitanensis* hydrolysates on fold change in cell number relative to day 0 of MACK1 cell line.

**Dunnett's multiple comparisons test**	**Mean Diff.**	**95.00% CI of diff.**	**Significant?**	**Summary**	**Adjusted P Value**
Control vs. AP - pepsin 1 µM	-1.217	-2.523 to 0.09004	No	ns	0.0745
Control vs. PH - proteinase K 1 µM	0.6667	-0.6400 to 1.973	No	ns	0.5615
Control vs. AP - pepsin 5 µM	-0.2666	-1.573 to 1.040	No	ns	0.9914
Control vs. PH - proteinase K 5 µM	1.397	0.09003 to 2.703	Yes	*	0.0332
Control vs. AP - pepsin 10 µM	0.2167	-1.090 to 1.523	No	ns	0.9971
Control vs. PH - proteinase K 10 µM	3.137	1.830 to 4.443	Yes	****	<0.0001
Control vs. AP - pepsin 20 µM	0.6500	-0.6566 to 1.957	No	ns	0.5868
Control vs. PH - proteinase K 20 µM	3.667	2.360 to 4.973	Yes	****	<0.0001

Table S28C. Two-way ANOVA statistical analysis for the effects of pre-selected *A. platensis* and *P. haitanensis* hydrolysates on fold change in cell number relative to day 0 of MACK1 cell line.

**Two-way ANOVA**	Ordinary
**Alpha**	0.05

Source of Variation	% of total variation	P value	P value summary	Significant?	
**Interaction**	2.384	0.5870	ns	No	
**Row (concentration) Factor**	29.29	0.0016	**	Yes	
**Column (enzyme) Factor**	49.14	<0.0001	****	Yes	
**ANOVA table**	**SS**	**DF**	**MS**	**F (DFn, DFd)**	**P value**
**Interaction**	1.340	3	0.4468	F (3, 16) = 0.6626	P=0.5870
**Row Factor**	16.47	3	5.489	F (3, 16) = 8.140	P=0.0016
**Column Factor**	27.63	1	27.63	F (1, 16) = 40.97	P<0.0001
**Residual**	10.79	16	0.6743		

Table S28D. Tukey's multiple comparisons test for the effects of pre-selected *A. platensis* and *P. haitanensis* hydrolysates on fold change in cell number relative to day 0 of MACK1 cell line.

Tukey's multiple comparisons test	Mean Diff.	95.00% CI of diff.	Significant?	Summary	Adjusted P Value
1 µM:AP - pepsin vs. 1 µM:PH - proteinase K	1.883	-0.4379 to 4.205	No	ns	0.1604
1 µM:AP - pepsin vs. 5 µM:AP - pepsin	0.9500	-1.371 to 3.271	No	ns	0.8370
1 µM:AP - pepsin vs. 5 µM:PH - proteinase K	2.617	0.2954 to 4.938	Yes	*	0.0216
1 µM:AP - pepsin vs. 10 µM:AP - pepsin	1.433	-0.8879 to 3.755	No	ns	0.4333
1 µM:AP - pepsin vs. 10 µM:PH - proteinase K	4.350	2.029 to 6.671	Yes	***	0.0002
1 µM:AP - pepsin vs. 20 µM:AP - pepsin	1.867	-0.4546 to 4.188	No	ns	0.1671
1 µM:AP - pepsin vs. 20 µM:PH - proteinase K	3.983	1.662 to 6.305	Yes	***	0.0004
1 µM:PH - proteinase K vs. 5 µM:AP - pepsin	-0.9333	-3.255 to 1.388	No	ns	0.8482
1 µM:PH - proteinase K vs. 5 µM:PH - proteinase K	0.7333	-1.588 to 3.055	No	ns	0.9491
1 µM:PH - proteinase K vs. 10 µM:AP - pepsin	-0.4500	-2.771 to 1.871	No	ns	0.9967
1 µM:PH - proteinase K vs. 10 µM:PH - proteinase K	2.467	0.1454 to 4.788	Yes	*	0.0332
1 µM:PH - proteinase K vs. 20 µM:AP - pepsin	-0.01667	-2.338 to 2.305	No	ns	>0.9999
1 µM:PH - proteinase K vs. 20 µM:PH - proteinase K	2.100	-0.2212 to 4.421	No	ns	0.0916

5 µM:AP - pepsin vs. 5 µM:PH - proteinase K	1.667	-0.6546 to 3.988	No	ns	0.2680
5 µM:AP - pepsin vs. 10 µM:AP - pepsin	0.4833	-1.838 to 2.805	No	ns	0.9949
5 µM:AP - pepsin vs. 10 µM:PH - proteinase K	3.400	1.079 to 5.721	Yes	**	0.0022
5 µM:AP - pepsin vs. 20 µM:AP - pepsin	0.9167	-1.405 to 3.238	No	ns	0.8590
5 µM:AP - pepsin vs. 20 µM:PH - proteinase K	3.033	0.7121 to 5.355	Yes	**	0.0064
5 µM:PH - proteinase K vs. 10 µM:AP - pepsin	-1.183	-3.505 to 1.138	No	ns	0.6495
5 µM:PH - proteinase K vs. 10 µM:PH - proteinase K	1.733	-0.5879 to 4.055	No	ns	0.2302
5 µM:PH - proteinase K vs. 20 µM:AP - pepsin	-0.7500	-3.071 to 1.571	No	ns	0.9431
5 µM:PH - proteinase K vs. 20 µM:PH - proteinase K	1.367	-0.9546 to 3.688	No	ns	0.4886
10 µM:AP - pepsin vs. 10 µM:PH - proteinase K	2.917	0.5954 to 5.238	Yes	**	0.0090
10 µM:AP - pepsin vs. 20 µM:AP - pepsin	0.4333	-1.888 to 2.755	No	ns	0.9974
10 µM:AP - pepsin vs. 20 µM:PH - proteinase K	2.550	0.2288 to 4.871	Yes	*	0.0261
10 µM:PH - proteinase K vs. 20 µM:AP - pepsin	-2.483	-4.805 to -0.1621	Yes	*	0.0316
10 µM:PH - proteinase K vs. 20 µM:PH - proteinase K	-0.3667	-2.688 to 1.955	No	ns	0.9991
20 µM:AP - pepsin vs. 20 µM:PH - proteinase K	2.117	-0.2046 to 4.438	No	ns	0.0876

Table S29. Number of cell divisions (log2fold change; day 7 vs day 0) of CHO-K1, QM7 and MACK1 cells treated with selected algal hydrolysates.

	**CHO-K1**		**QM7**		**MACK1**	
**Hydrolysates (µM)**	**Pepsin – *P. haitanensis***	**Chymotrypsin – *P. haitanensis***	**Chymotrypsin – *A. platensis***	**Proteinase K – *P. haitanensis***	**Pepsin – *A. platensis***	**Proteinase K – *P. haitanensis***
**0**	5.82 ± 0.10		4.45 ± 0.15		1.90 ± 0.11	
**1**	5.46 ± 0.03	5.63 ± 0.2	4.34 ± 0.01	4.32 ± 0.08	2.30 ± 0.17	1.63 ± 0.07
**5**	5.86 ± 0.03	5.59 ± 0.13	4.21 ± 0.16	3.93 ± 0.09	2.01 ± 0.04	1.20 ± 0.25
**10**	6.10 ± 0.10	5.42 ± 0.12	3.74 ± 0.02	2.64 ± 0.22	1.81 ± 0.16	-0.68 ± 0.16

**20**	5.53 ± 0.23	5.39 ± 0.12	3.31 ± 0.02	2.36 ± 0.15	1.63 ± 0.10	-1.74 ± 0.00

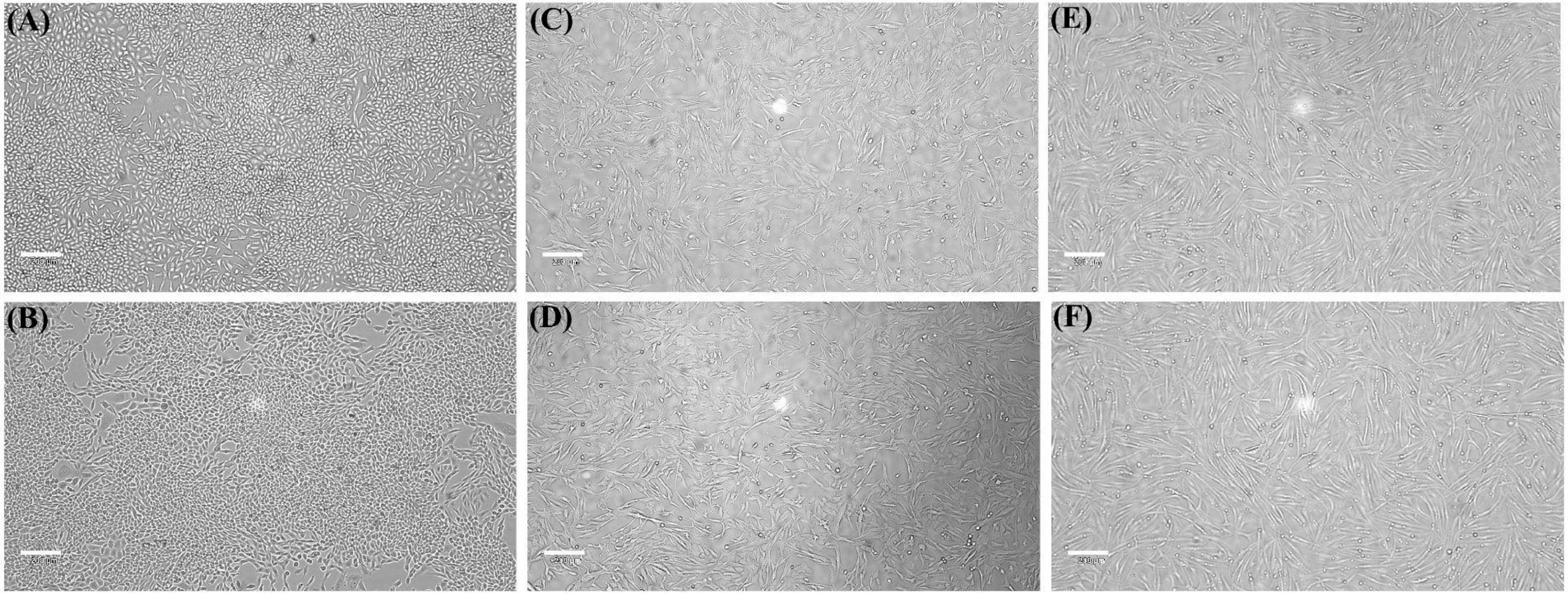

Figure S1. Morphology of CHO-K1, QM7 and MACK1 cells treated with 5 μM of the selected algal hydrolysate: (A) CHO-K1 – Control, (B) CHO-K1 – PH-pepsin, (C) QM7 – Control, (D) QM7 – AP-chymotrypsin, (E) MACK1 – Control (F) MACK1 – AP-pepsin.

Table S30. pH measurments of the cell media following the trypan blue exclusion counting test.

	CHO-K1		QM7		MACK1	
**Hydrolysates (µM)**	**Pepsin – *P. haitanensis***	**Chymotrypsin – *P. haitanensis***	**Chymotrypsin – *A. platensis***	**Proteinase K – *P. haitanensis***	**Pepsin – *A. platensis***	**Proteinase K – *P. haitanensis***
**0**	7.78		7.93		7.36	
**1**	7.81	7.83	8.00	8.02	7.33	7.30
**5**	7.79	7.82	7.93	8.05	7.33	7.30
**10**	7.80	7.80	7.92	7.93	7.31	7.29
**20**	7.81	7.81	7.89	7.85	7.32	7.28

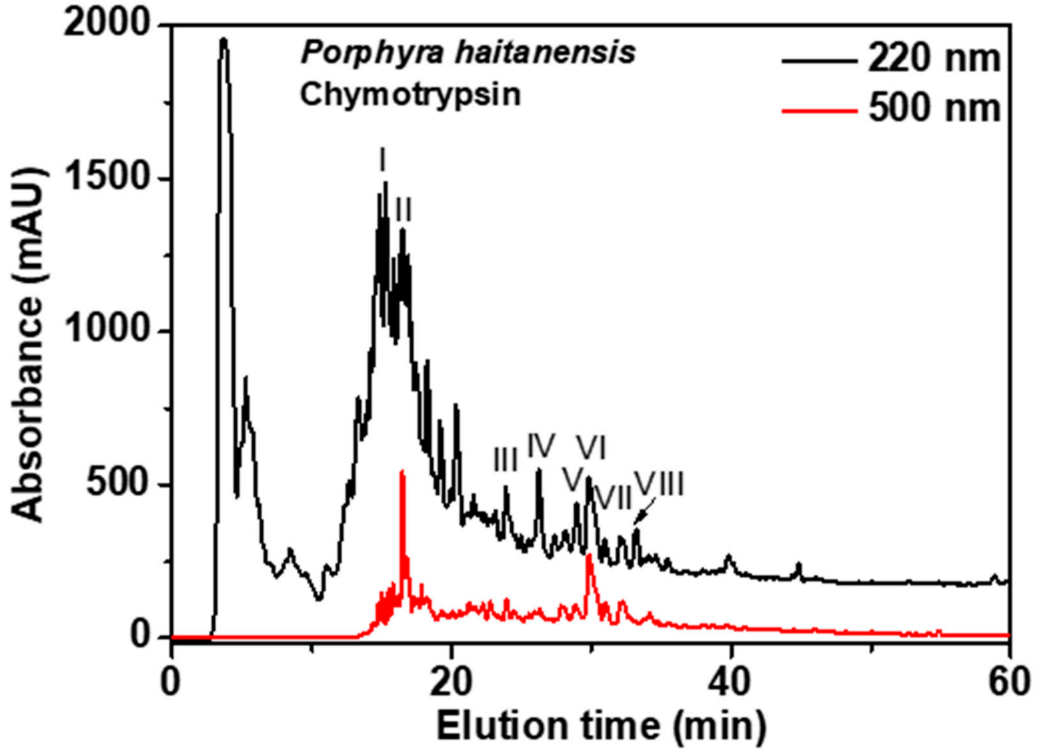

Figure S2. Chromatogram of the separation (C18 column) of chromopeptides originating from PH-chymotrypsin.

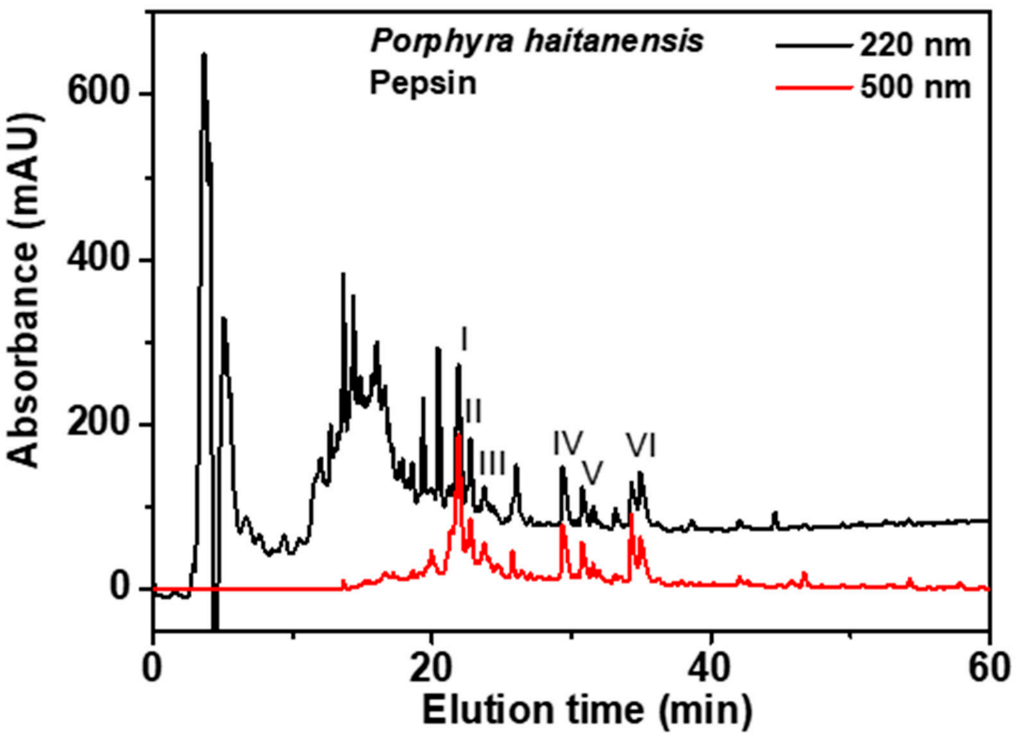

Figure S3. Chromatogram of the separation (C18 column) of chromopeptides originating from PH-pepsin.

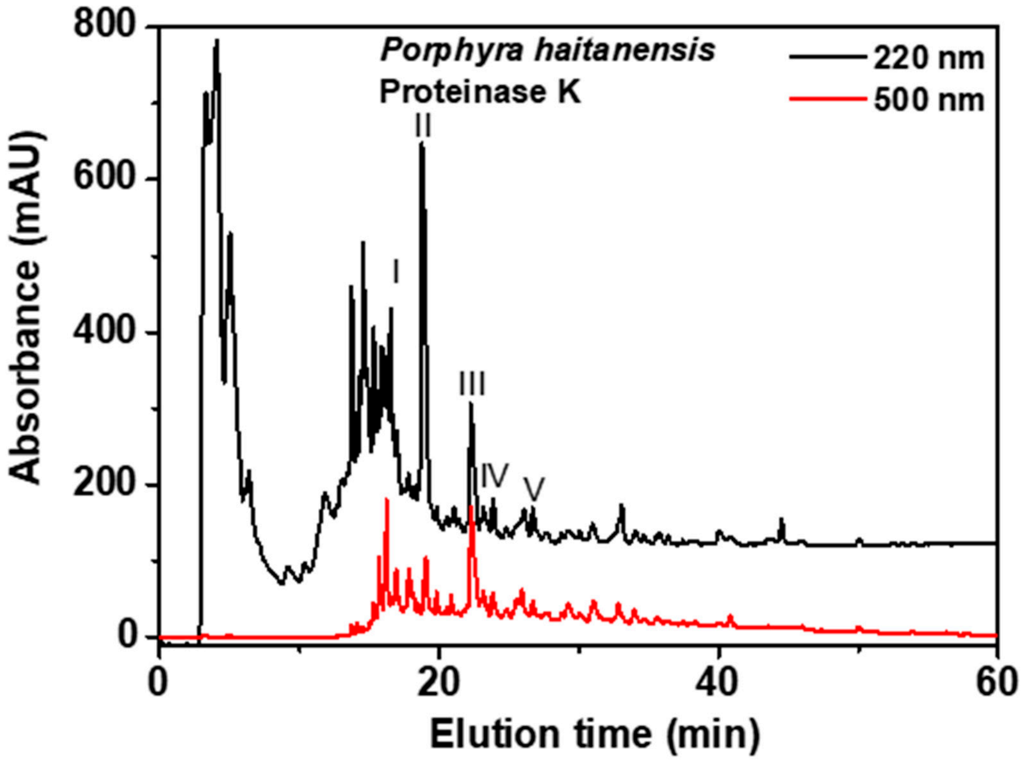

Figure S4. Chromatogram of the separation (C18 column) of chromopeptides originating from PH-proteinase K.

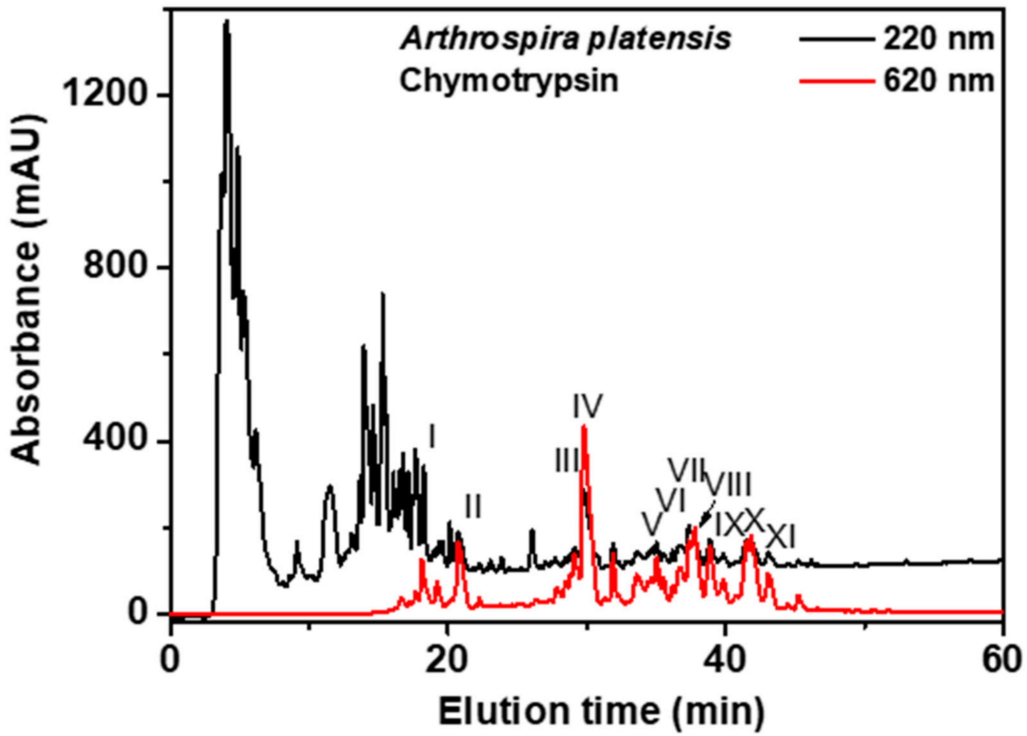

Figure S5. Chromatogram of the separation (C18 column) of chromopeptides originating from AP-chymotrypsin.

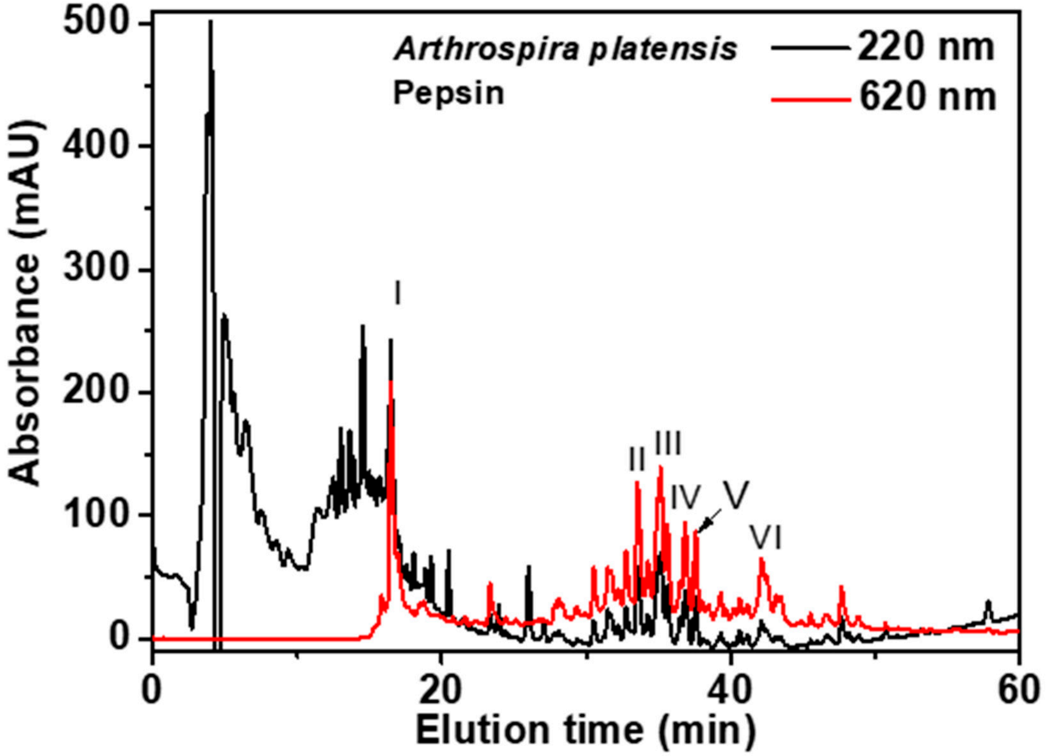

Figure S6. Chromatogram of the separation (C18 column) of chromopeptides originating from AP-pepsin.

Table S31. Chromopeptide sequences identified by tandem mass spectrometry after chymotrypsin digestion of PBPs from *Porphyra haitanensis*

m/z	z	Experimental mass	Theoretical mass	Delta mass	Sequence	Fraction	Phycobiliprotein subunit/position of chromophore binding	UniProt accession number
653.8027	2	1305.5898	1305.5886	0.0012	CVPRDM	1	R-PE α	Q0ZHI7
568.2914	2	1134.5672	1134.576	-0.0088	AKCAR	2	R-PC α (PCB Cys84)	Q0ZHI5
532.7727	2	1063.5297	1063.539	-0.0093	KCAR	2	R-PC α (PCB Cys84)	Q0ZHI5
760.3872	2	1518.7586	1518.735*	0.0236	NRRMAACL	3	R-PE α (PEB (Cys139))	Q0ZHI7
867.39	2	1732.7643	1732.7564*	0.0079	YTSRRMAACLRDME	3	R-PC β (PEB Cys153 (Met ox))	Q0ZHI6
625.8054	2	1249.5951	1249.5988	-0.0037	RMAACL / MAACLR	4	R-PE β / R-PC β (PEB)	Q0ZHI8 / Q0ZHI6
767.8522	2	1533.6888	1533.681	0.0078	GTPQGDCSAL	5	R-PE β (PEB)	Q0ZHI8
576.2629	2	1150.5102	1150.5005	0.0097	GDCSAL	6	R-PE β (PEB)	Q0ZHI8
755.338	2	1508.6604	1508.6493	0.0111	TADGDCSALA	6	R-PE β (PEB)	Q0ZHI8
669.2947	2	1336.5738	1336.5646	0.0092	DGDCSALA	7	R-PE β (PEB)	Q0ZHI8
874.9194	2	1747.8231	1747.8127	0.0104	LTGTPQGDCSAL	7	R-PC β (PCB Cys153-region)	Q0ZHI6
755.3375	2	1508.6593	1508.6493	0.01	TADGDCSALA	7	R-PE β (PEB)	Q0ZHI8
1024.4603	2	2046.905	2046.9357	-0.0307	PGEAGDSQEKVNKC	8	R-PE α	Q0ZHI7
390.1756	2	778.3356	778.336	-0.0004	AC and/or CA	8	R-PE β (PEB)/RPC α (PEB) Cys 84	Q0ZHI8/ Q0ZHI5

* Theoretical mass corrected for ±2H reported instead of the original theoretical mass where it is closer to the experimental mass.
Underlined cysteine (C) in the Sequence column indicates the residue covalently bound to the chromophore.

Table S32. Chromopeptide sequences identified by tandem mass spectrometry after pepsin digestion of PBPs from *Porphyra haitanensis*

m/z	z	Experimental mass	Theoretical mass	Delta mass	Sequence	Fraction	Phycobiliprotein subunit/position of chromophore binding	UniProt accession number
726.3095	2	1450.6044	1450.6075	-0.0031	TGTPQGDCS	1	R-PC β, Cys153	Q0ZHI6
398.1718	2	794.3289	794.3309	-0.002	CS	1	R-PC β, Cys153	Q0ZHI6
663.2704	2	1324.5262	1324.5282	-0.002	ATADGDCS	2	R-PE β, Cys158	Q0ZHI8
577.2279	2	1152.4413	1152.4434	-0.0021	ADGDCS	2	R-PE β, Cys158	Q0ZHI8
484.196	2	966.3773	966.3793	-0.002	GDCS	2	R-PE β / R-PC β, Cys158/Cys153	Q0ZHI8 / Q0ZHI6

m/z	z	Experimental mass	Theoretical mass	Delta mass	Sequence	Fraction	Phycobiliprotein subunit/position of chromophore binding	UniProt accession number
433.6905	2	865.3664	865.368	-0.0016	CSA	2	R-PE β / R-PC β, Cys158/Cys153	Q0ZHI8 / Q0ZHI6
455.6852	2	909.3558	909.3579	-0.0021	DCS	3	R-PE β / R-PC β, Cys158/Cys153	Q0ZHI8 / Q0ZHI6
575.2725	3	1722.7955	1722.7858	0.0097	RMAACLRDGE	3	R-PE β, Cys82 (Met-ox)	Q0ZHI8
782.8514	2	1563.6883	1563.6915	-0.0032	LTGTPQGDCS	3	R-PC β, Cys153	Q0ZHI6
698.7889	2	1395.5632	1395.5653	-0.0021	ATADGDCSA	3	R-PE β, Cys158	Q0ZHI8
404.1906	2	806.3667	806.3673	-0.0006	CV	4	R-PE α, Cys139	Q0ZHI7
490.2323	2	978.4501	978.4521	-0.002	CSAL	5	R-PE β / R-PC β, Cys158/Cys153	Q0ZHI8 / Q0ZHI6
482.235	2	962.4553	962.4572	-0.0019	AACL	6	R-PE β Cys82 (PEB) / R-PC β Cys82 (PCB)	Q0ZHI8 / Q0ZHI6
446.7165	2	891.4184	891.4201	-0.0017	ACL	6	R-PE β Cys82 (PEB) / R-PC β Cys82 (PCB)	Q0ZHI8 / Q0ZHI6
411.1979	2	820.3813	820.3829	-0.0016	CL	6	R-PE β Cys82 (PEB) / R-PC β Cys82 (PCB)	Q0ZHI8 / Q0ZHI6

Underlined cysteine (C) in the Sequence column indicates the residue covalently bound to the chromophore.

Table S33. Chromopeptide sequences identified by tandem mass spectrometry after proteinase K digestion of PBPs from *Porphyra haitanensis*

m/z	z	Experimental mass	Theoretical mass	Delta mass	Sequence	Fraction	Phycobiliprotein subunit/position of chromophore binding	UniProt accession number
726.8375	2	1451.6593	1451.6643*	-0.005	GDCSALVAE	1	R-PC β/Cys153	Q0ZHI6
670.2953	2	1338.5749	1338.5791*	-0.0042	ADGDCSAL	1	R-PE β/Cys158	Q0ZHI8
613.7529	2	1225.4902	1225.4961*	-0.0059	ADGDCSA	1	R-PE β/Cys158	Q0ZHI8
509.7676	2	1017.5196	1017.5114	0.0082	LCVP	1	R-PE α/Cys139	Q0ZHI7
534.2335	2	1066.4513	1066.443*	0.0083	SNASC	2	R-PE β/Cys50	Q0ZHI8
441.688	2	881.3603	881.3629*	-0.0026	GDC	2	R-PE β Cys1β58/ R-PC β Cys153	Q0ZHI8/ Q0ZHI6

m/z	z	Experimental mass	Theoretical mass	Delta mass	Sequence	Fraction	Phycobiliprotein subunit/position of chromophore binding	UniProt accession number
484.1978	2	966.3799	966.3793	0.0006	GDCS	3	R-PE β Cys1β58/ R-PC β Cys153	Q0ZHI8/ Q0ZHI6
519.7198	2	1037.4239	1037.4164	0.0075	GDCSA	4	R-PE β Cys1β58/ R-PC β Cys153	Q0ZHI8/ Q0ZHI6
577.2336	2	1152.4515	1152.4434	0.0081	DGDCSA	4	R-PE β/Cys158	Q0ZHI8
726.8364	2	1451.6571	1451.6643	-0.0072	GDCSALVAE	4	R-PE β Cys1β58/ R-PC β Cys153	Q0ZHI8/ Q0ZHI6
455.6896	2	909.3636	909.3579	0.0057	DCS	4	R-PE β Cys1β58/ R-PC β Cys153	Q0ZHI8/ Q0ZHI6
625.8367	2	1249.6577	1249.6541	0.0036	RDRLC	4	R-PE α/Cys139	Q0ZHI7
541.7117	2	1081.4078	1081.4063	0.0015	DGDCS	4	R-PE β/Cys158	Q0ZHI8
483.1898	2	964.3639	964.3637*	0.0002	GDCS	5	R-PE β Cys1β58/ R-PC β Cys153	Q0ZHI8/ Q0ZHI6
653.8025	2	1305.5894	1305.5886	0.0008	CVPRDM	5	R-PE α/Cys139	Q0ZHI7

*** Theoretical mass corrected for ±2H reported instead of the original theoretical mass where it is closer to the experimental mass.**
Underlined cysteine (C) in the Sequence column indicates the residue covalently bound to the chromophore.

Table S34. Chromopeptide sequences identified by tandem mass spectrometry after chymotrypsin digestion of PBPs from *Arthrospira platensis*

m/z	z	Experimental mass	Theoretical mass	Delta mass	Sequence	Fractio n	Phycobiliprotein subunit/position of chromophore binding	UniProt accession number
559.9409	3	1676.7992	1676.7981	0.0011	ADQRGKDKCA	1	C-PC α, Cys85	P72509
437.9667	4	1747.8355	1747.8352	0.0003	AADQRGKDKCA	1	C-PC α, Cys85	P72509
403.2	3	1206.5765	1206.5743	0.0022	GKDKCA	2	C-PC α, Cys85	P72509
560.2914	2	1118.5671	1118.5583	0.0088	AACLR	3	C-PC β, Cys82	P72508
484.2012	2	966.3867	966.3793	0.0074	GDCS	3	C-PC β, Cys152	P72508
403.2	3	1206.5765	1206.5743	0.0022	GKDKCA	3	C-PC α, Cys85	P72509
519.7165	2	1037.4173	1037.4164	0.0009	GDCSA	4	C-PC β, Cys152	P72508
455.8835	3	1364.627	1364.6257	0.0013	AACLRDM	4	C-PC β, Cys82	P72508
675.313	2	1348.6104	1348.6009	0.0095	TPGDCSAL	5	C-PC β, Cys152	P72508
902.4043	2	1802.793	1802.7822	0.0108	NDPAGITPGDCSA	5	C-PC β, Cys152	P72508

m/z	z	Experimental mass	Theoretical mass	Delta mass	Sequence	Fraction	Phycobiliprotein subunit/position of chromophore binding	UniProt accession number
731.8608	2	1461.706	1461.6962	0.0098	AACIRDLD	5	APC β, Cys81	P72505
722.2943	2	1442.5729	1442.5734	-0.0005	GEEMTATC (Met-ox)	6	APC α, Cys81	P72504
488.2397	3	1461.6956	1461.6962	-0.0006	AACIRDLD	6	APC β, Cys81	P72505
952.4346	2	1902.8536	1902.8532	0.0004	MTATCLRDLDY (Met-ox)	6	APC α, Cys81	P72504
1044.4949	2	2086.9741	2085.9717*	-0.0054	AIVNDPAGITPGDCSA	7	C-PC β, Cys153	P72508
787.8631	2	1573.7106	1573.7123	-0.0017	PAGITPGDCSA	7	C-PC β, Cys153	P72508
542.9279	3	1625.7603	1625.7548	0.0055	DKCARDIGY	7	C-PC α, Cys84	P72509
425.694	2	849.3723	849.3731	-0.0008	AAC	8	C-PC β, Cys82	P72508
491.2231	2	980.4305	980.4314	-0.0009	TATC	8	APC α, Cys81	P72504
714.2976	2	1426.5794	1426.5785	0.0009	GEEMTATC	9	APC α, Cys81	P72504
425.695	2	849.3743	849.3731	0.0012	AAC	9	C-PC β, Cys82	P72508
576.2589	2	1150.5022	1150.5005	0.0017	GDCSAL	9	C-PC β, Cys153	P72508
482.2363	2	962.457	962.4572	-0.0002	AACL	10	C-PC β, Cys82	P72508
390.1756	2	778.3355	778.336	-0.0005	AC	10	C-PC β, Cys82	P72508
446.7174	2	891.4191	891.4193	-0.0002	ACL	11	C-PC β, Cys82	P72508

* Theoretical mass corrected for ±2H reported instead of the original theoretical mass where it is closer to the experimental mass.
Underlined cysteine (C) in the Sequence column indicates the residue covalently bound to the chromophore.

Table S35. Chromopeptide sequences identified by tandem mass spectrometry after pepsin digestion of PBPs from *Arthrospira platensis*

m/z	z	Experimental mass	Theoretical mass	Delta mass	Sequence	Fraction	Phycobiliprotein subunit/postion of chromophore binding	UniProt accession number
505.9992	4	2019.9654	2019.9479*	0.01752	AADQRGKDKCARD	1	C-PC α/Cys84	P72509
487.9897	4	1947.9276	1947.9261	0.0014	ADQRGKDKCARD	1	C-PC α/Cys84	P72509
675.3084	2	1348.6011	1348.6009	0.0001	ITPGDCSA or TPGDCSAL	2	C-PC β/Cys153	P72508
596.2736	2	1190.5315	1190.5430**	-0.0116	TCLRD	3	APC α/Cys 81	P72504
787.3653	2	1575.7228	1575.7279**	-0.0052	PAGITPGDCSA	3	C-PC β/Cys153	P72508
708.8304	2	1415.6452	1415.6431	0.0021	PAGITPGDC	4	C-PC β/Cys153	P72508
844.3707	2	1686.7258	1686.7340**	-0.0082	DPAGITPGDCSA	4	C-PC β/Cys153	P72508

**766.3425**	**2**	**1530.6694**	**1530.6699**	**-0.0005**	**DPAGITPGDC**	**5**	**C-PC β/Cys153**	**P72508**
**491.2220**	**2**	**980.4283**	**980.4312**	**-0.0030**	**TATC**	**5**	**APC α/Cys 81**	**P72504**
**490.2336**	**2**	**978.4516**	**978.4520**	**-0.0003**	**CSAL**	**5**	**C-PC β/Cys153**	**P72508**
**482.2360**	**2**	**962.4563**	**962.4570**	**-0.0007**	**AACL or AACI**	**6**	**APC β/Cys81 or C-PC β/Cys82**	**P72505/P72508**
**497.2412**	**2**	**992.4668**	**992.4676**	**-0.0008**	**ATCL**	**6**	**APC α/Cys 81**	**P72509**
**390.1754**	**2**	**778.3352**	**778.3359**	**-0.0007**	**CA or/and AC**	**6**	**APC β/Cys81, C-PC α/Cys84, C-PC β/Cys82**	**P72505/P72509/P72508**
**547.7649**	**2**	**1093.5141**	**1093.5153**	**-0.0012**	**TATCL**	**6**	**APC α/Cys 81**	**P72504**

*** Corrected theoretical mass (e.g. deamidation correction) reported instead of original theoretical mass.**

**** Theoretical mass corrected for ±2H reported instead of original theoretical mass where it is closer to the experimental ma**

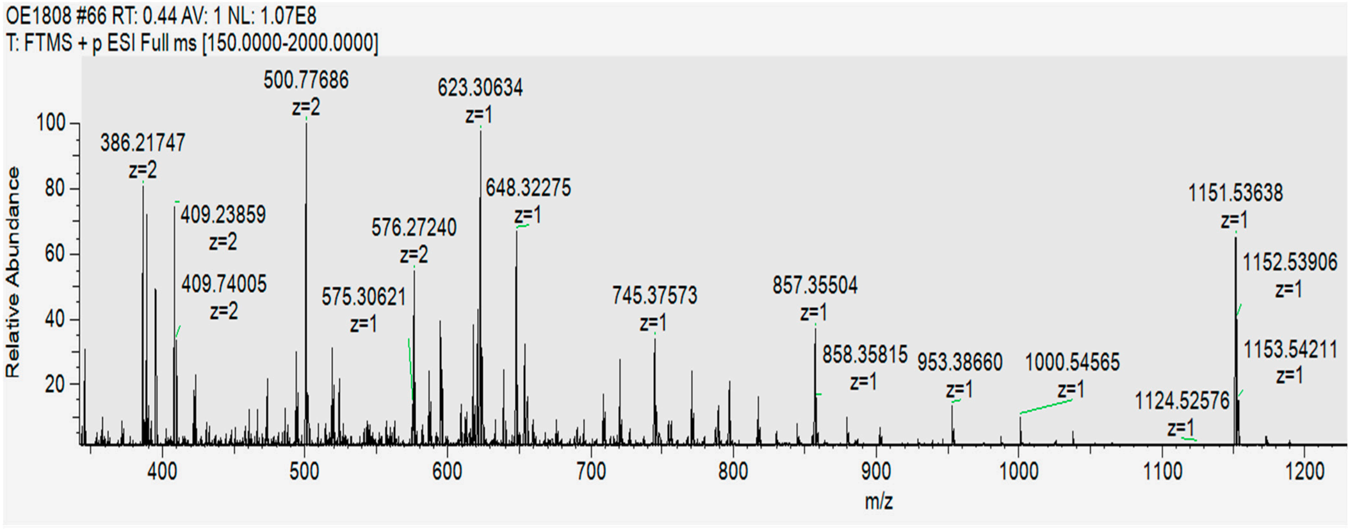

Figure S7. MS spectrum of Fraction 1, obtained after RP-HPLC purification of chymotrypsin digest of PBPs from *Porphyra haitanensis*

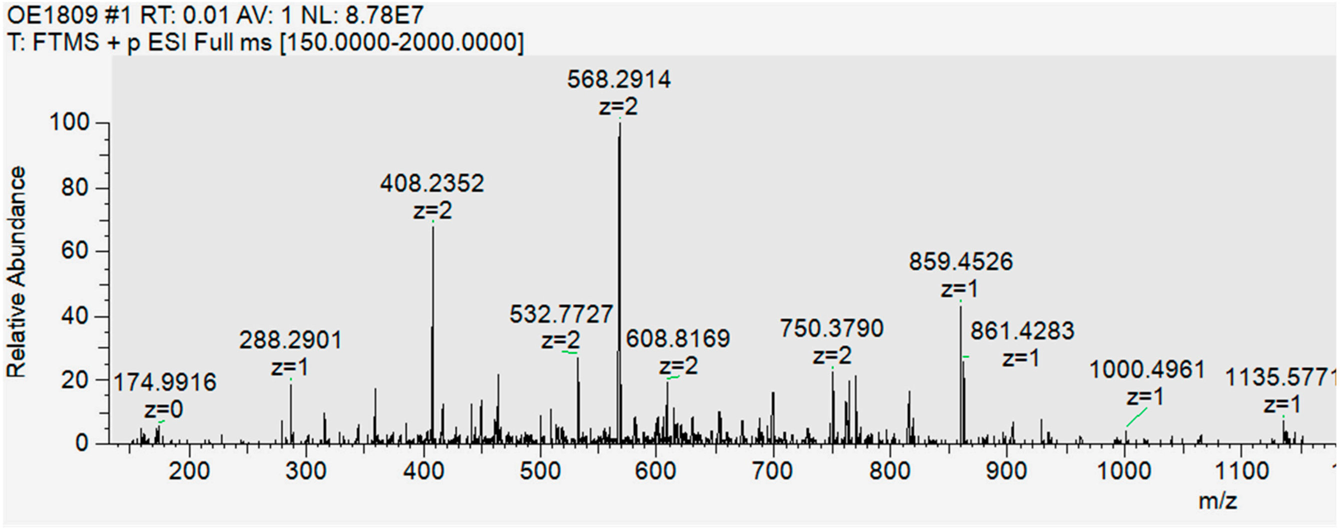

Figure S8. MS spectrum of Fraction 2, obtained after RP-HPLC purification of chymotrypsin digest of PBPs from *Porphyra haitanensis*

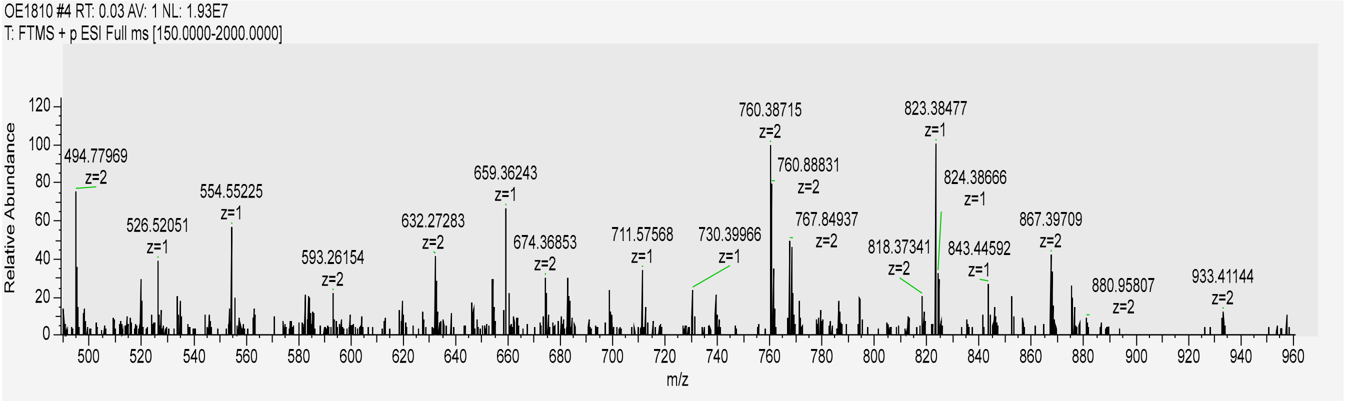

Figure S9. MS spectrum of Fraction 3, obtained after RP-HPLC purification of chymotrypsin digest of PBPs from *Porphyra haitanensis*

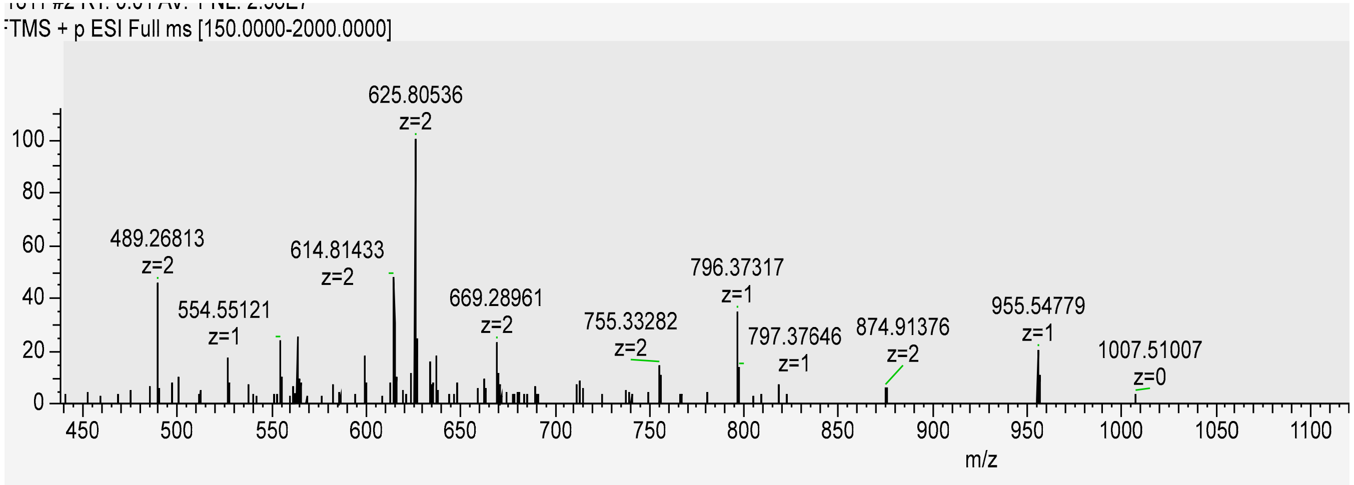

Figure S10. MS spectrum of Fraction 4, obtained after RP-HPLC purification of chymotrypsin digest of PBPs from *Porphyra haitanensis*

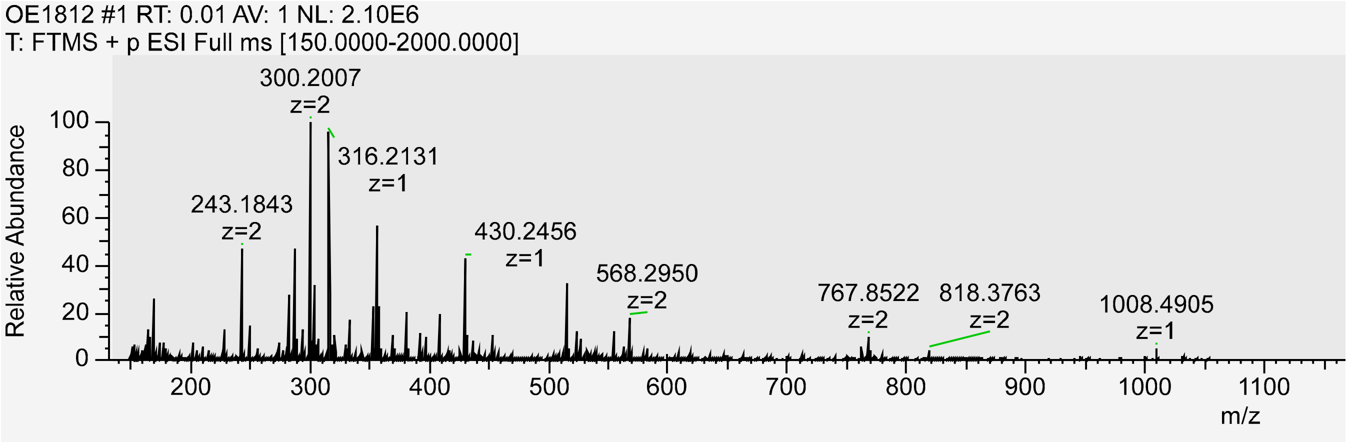

Figure S11. MS spectrum of Fraction 5, obtained after RP-HPLC purification of chymotrypsin digest of PBPs from *Porphyra haitanensis*

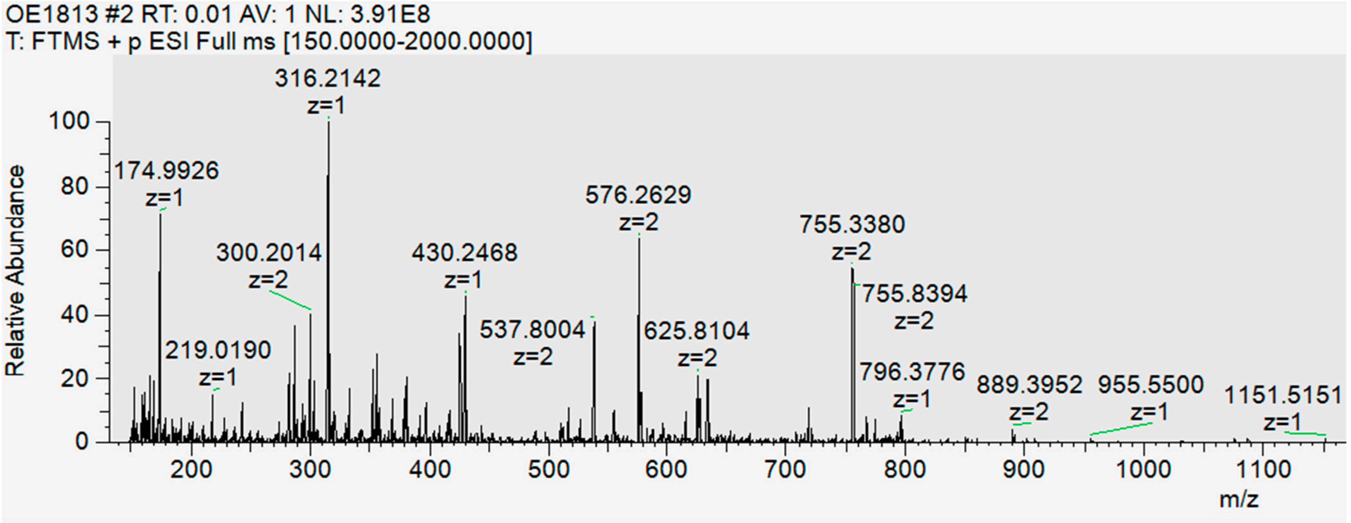

Figure S12. MS spectrum of Fraction 6, obtained after RP-HPLC purification of chymotrypsin digest of PBPs from *Porphyra haitanensis*

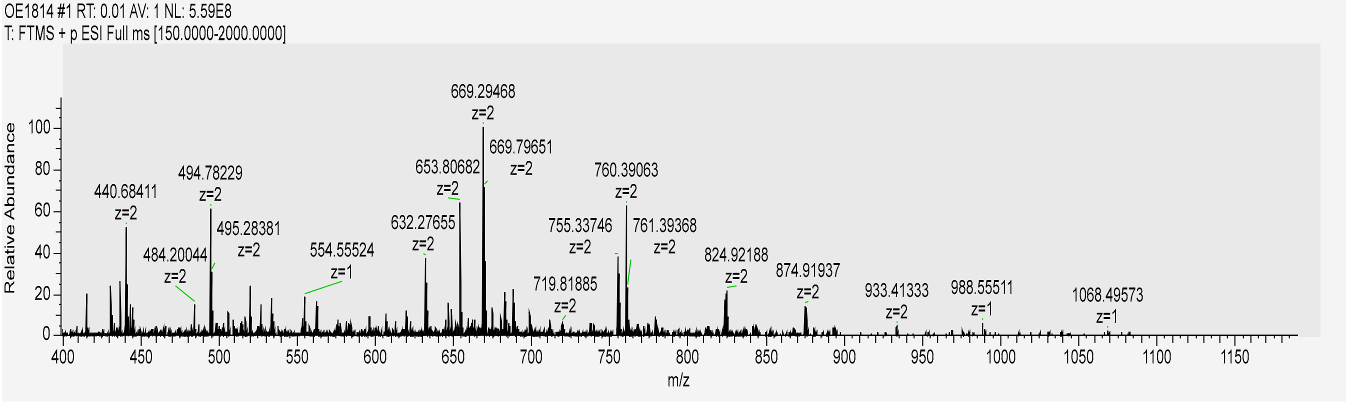

Figure S13. MS spectrum of Fraction 7, obtained after RP-HPLC purification of chymotrypsin digest of PBPs from *Porphyra haitanensis*

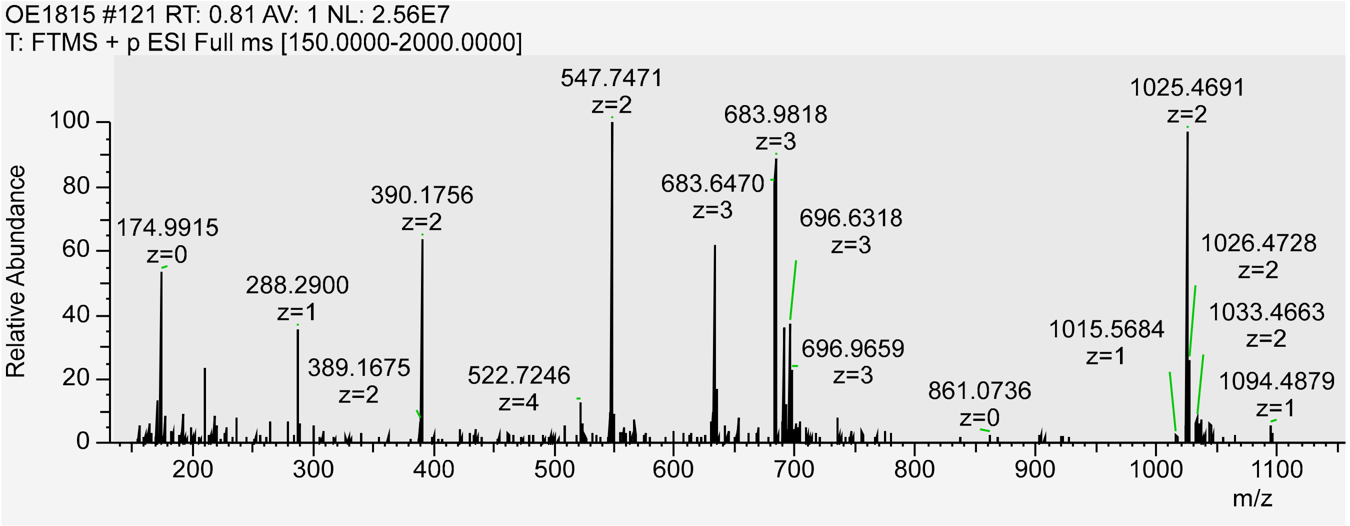

Figure S14. MS spectrum of Fraction 8, obtained after RP-HPLC purification of chymotrypsin digest of PBPs from *Porphyra haitanensis*

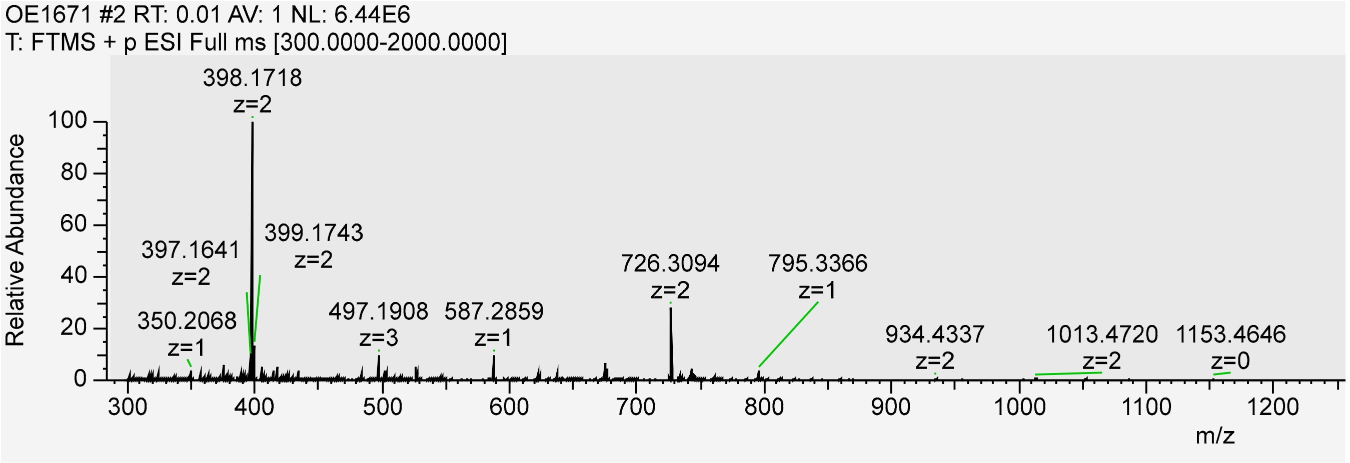

Figure S15. MS spectrum of Fraction 1, obtained after RP-HPLC purification of pepsin digest of PBPs from *Porphyra haitanensis*

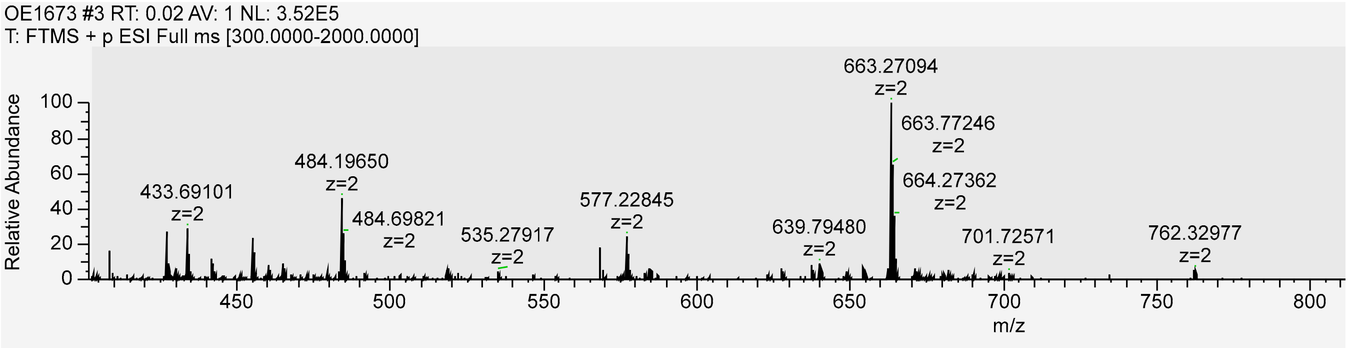

Figure S16. MS spectrum of Fraction 2, obtained after RP-HPLC purification of pepsin digest of PBPs from *Porphyra haitanensis*

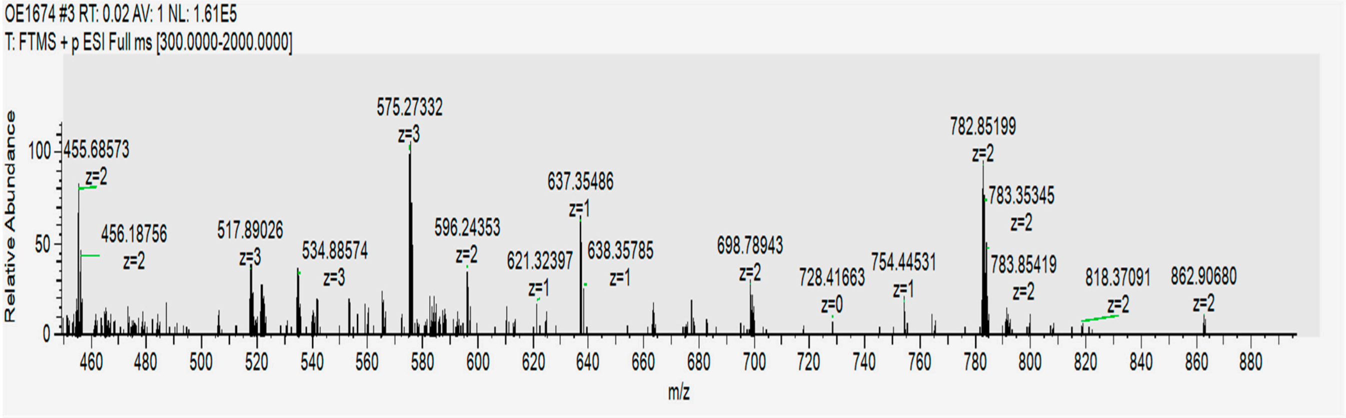

Figure S17. MS spectrum of Fraction 3, obtained after RP-HPLC purification of pepsin digest of PBPs from *Porphyra haitanensis*

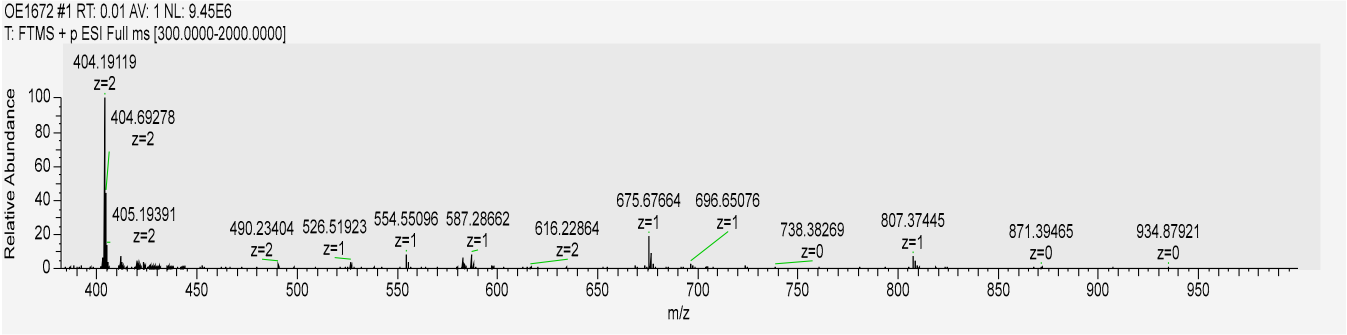

Figure S18. MS spectrum of Fraction 4, obtained after RP-HPLC purification of pepsin digest of PBPs from *Porphyra haitanensis*

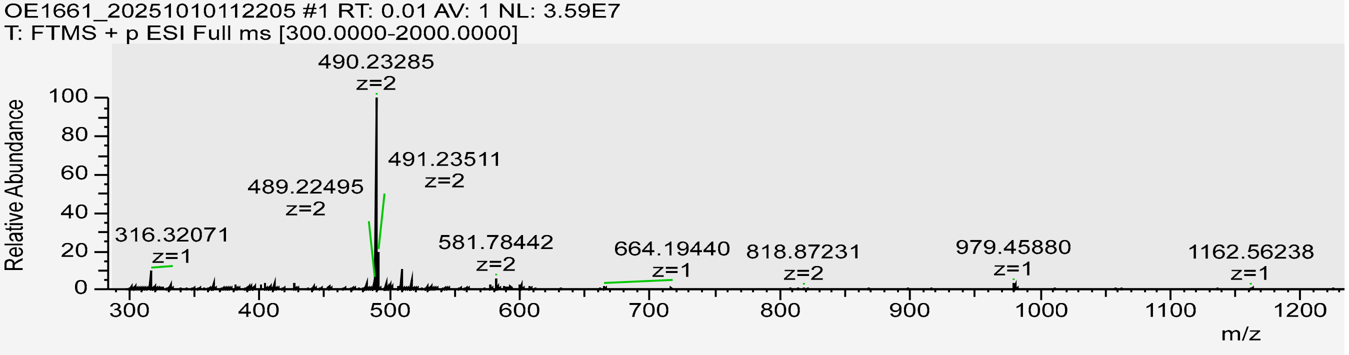

Figure S19. MS spectrum of Fraction 5, obtained after RP-HPLC purification of pepsin digest of PBPs from *Porphyra haitanensis*

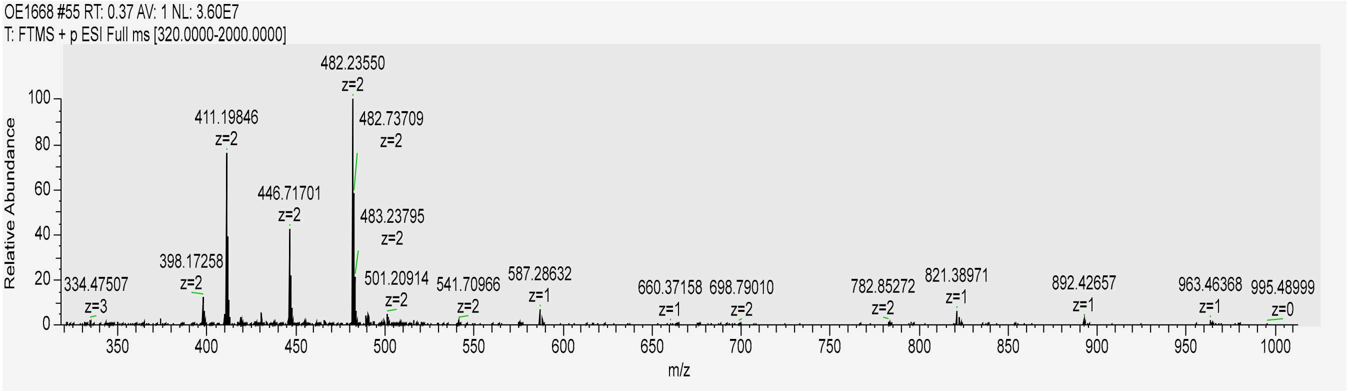

Figure S20. MS spectrum of Fraction 6, obtained after RP-HPLC purification of pepsin digest of PBPs from *Porphyra haitanensis*

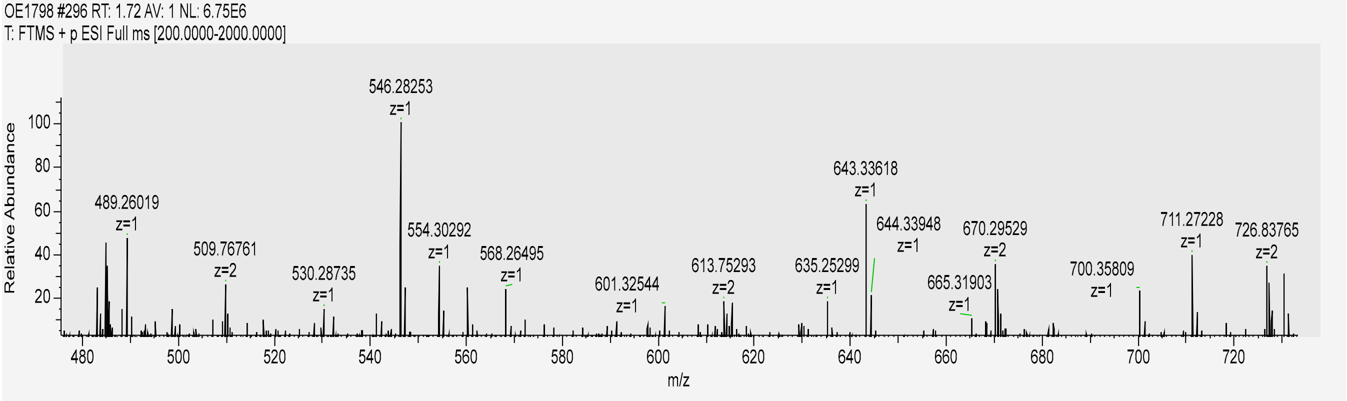

Figure S21. MS spectrum of Fraction 1, obtained after RP-HPLC purification of proteinase K digest of PBPs from *Porphyra haitanensis*

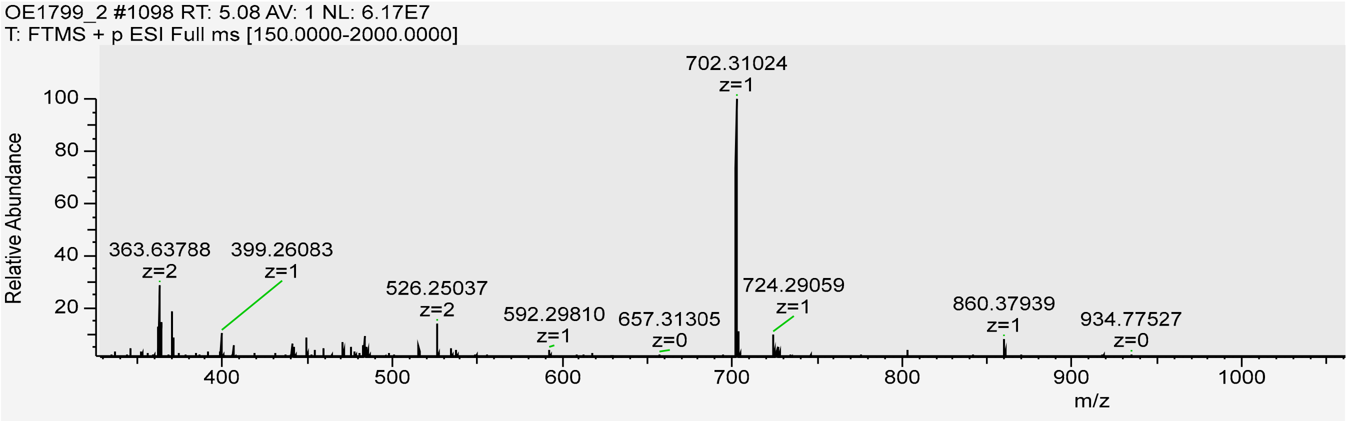

Figure S22. MS spectrum of Fraction 2, obtained after RP-HPLC purification of proteinase K digest of PBPs from *Porphyra haitanensis*

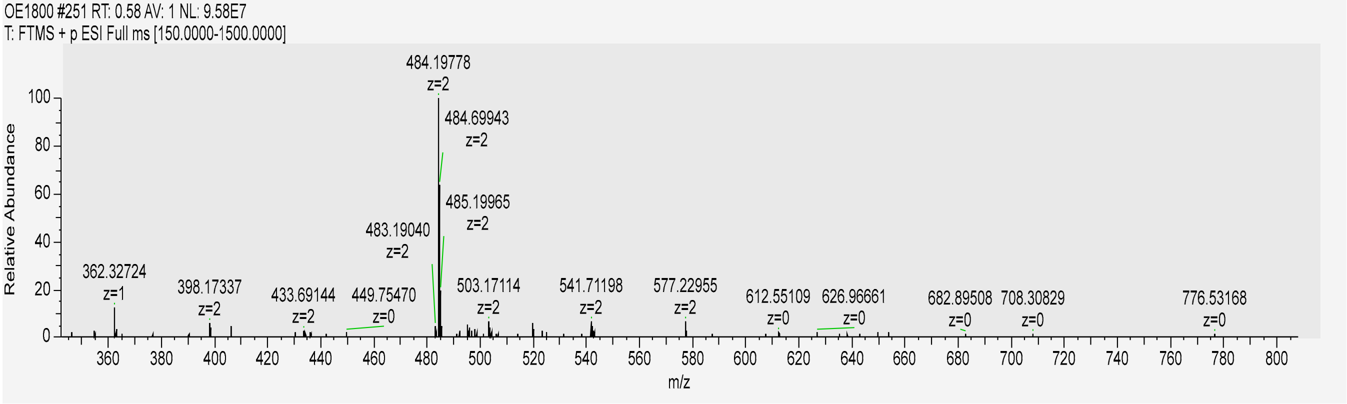

Figure S23. MS spectrum of Fraction 3, obtained after RP-HPLC purification of proteinase K digest of PBPs from *Porphyra haitanensis*

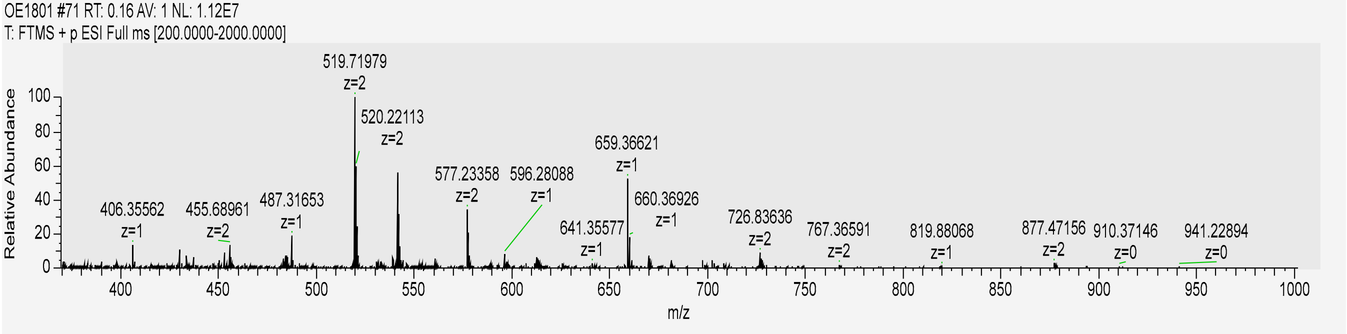

Figure S24. MS spectrum of Fraction 4, obtained after RP-HPLC purification of proteinase K digest of PBPs from *Porphyra haitanensis*

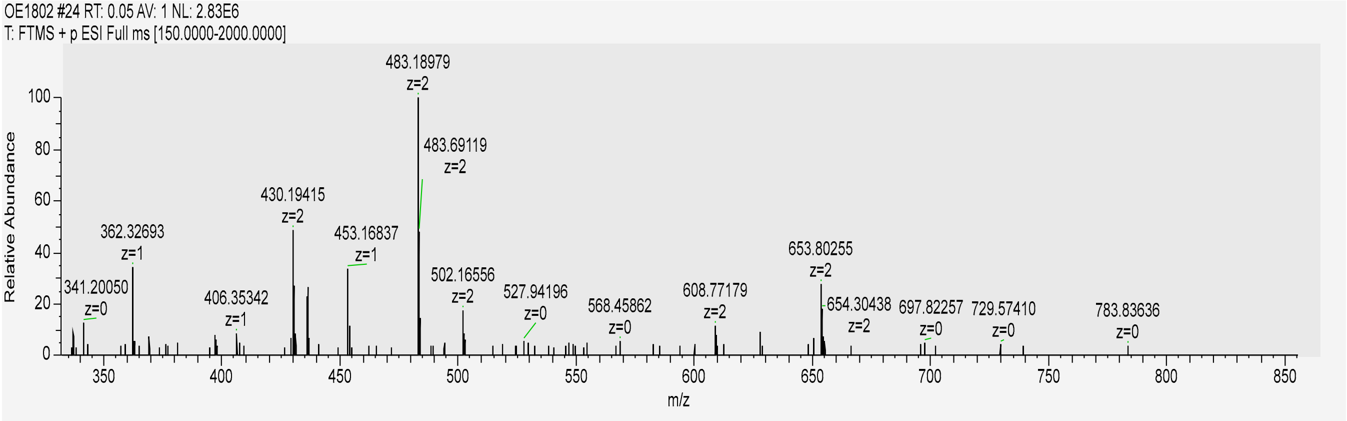

Figure S25. MS spectrum of Fraction 5, obtained after RP-HPLC purification of proteinase K digest of PBPs from *Porphyra haitanensis*

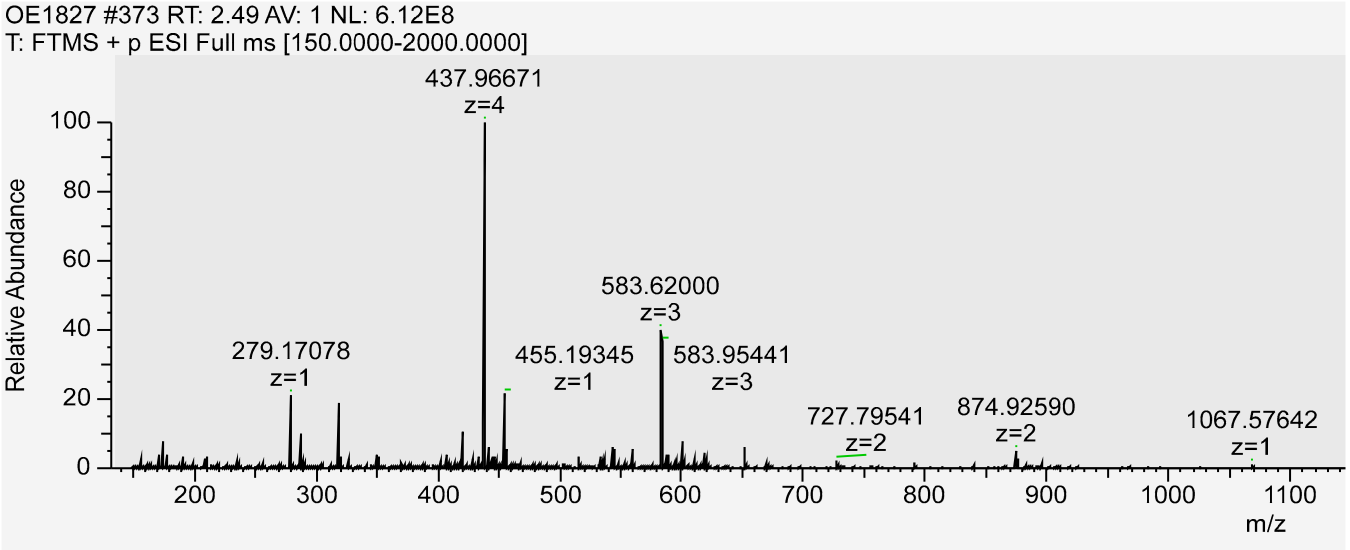

Figure S26. MS spectrum of Fraction 1, obtained after RP-HPLC purification of chymotrypsin digest of PBPs from *Arthrospira platensis*

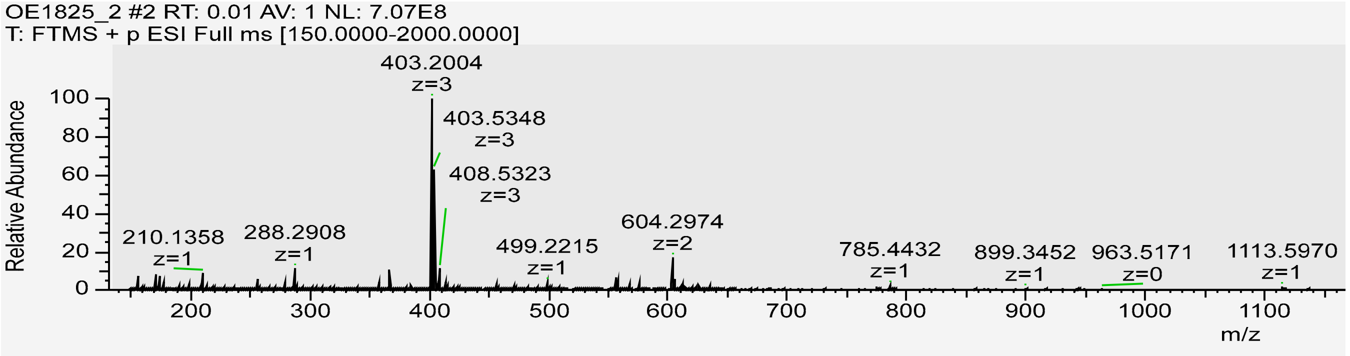

Figure S27. MS spectrum of Fraction 2, obtained after RP-HPLC purification of chymotrypsin digest of PBPs from *Arthrospira platensis*

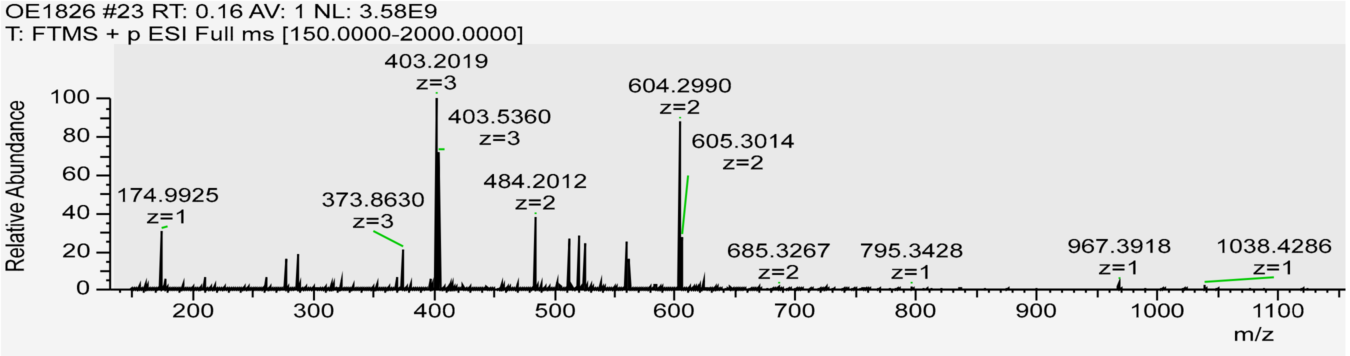

Figure S28. MS spectrum of Fraction 3, obtained after RP-HPLC purification of chymotrypsin digest of PBPs from *Arthrospira platensis*

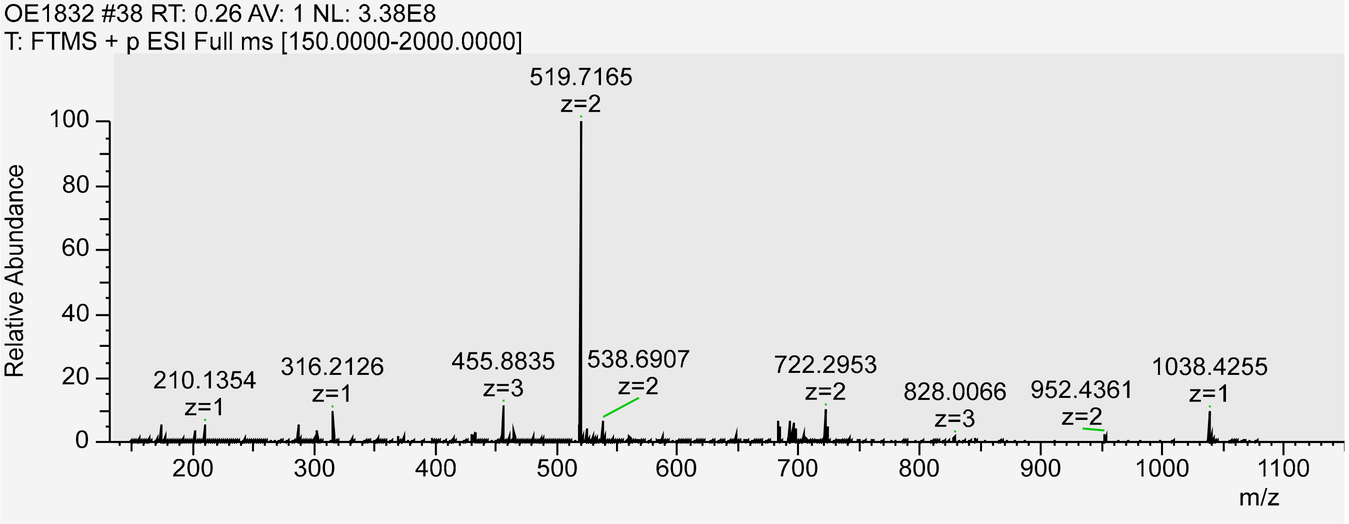

Figure S29. MS spectrum of Fraction 4, obtained after RP-HPLC purification of chymotrypsin digest of PBPs from *Arthrospira platensis*

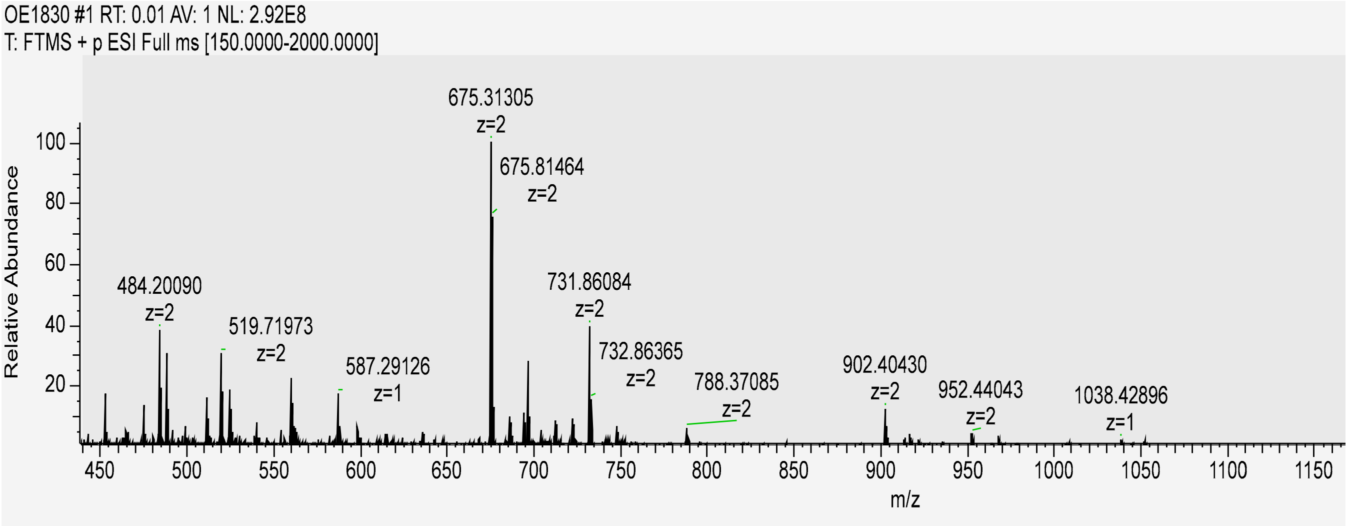

Figure S30. MS spectrum of Fraction 5, obtained after RP-HPLC purification of chymotrypsin digest of PBPs from *Arthrospira platensis*

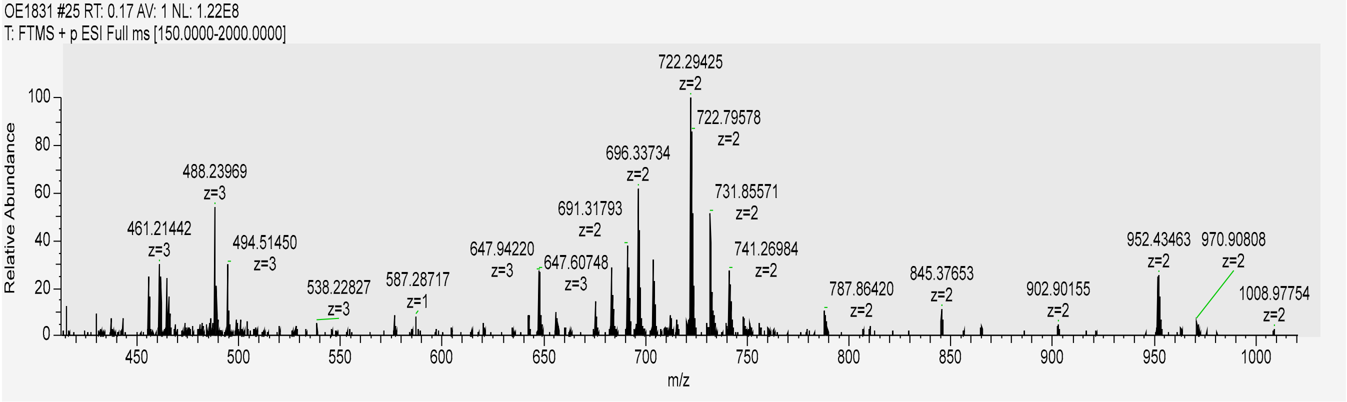

Figure S31. MS spectrum of Fraction 6, obtained after RP-HPLC purification of chymotrypsin digest of PBPs from *Arthrospira platensis*

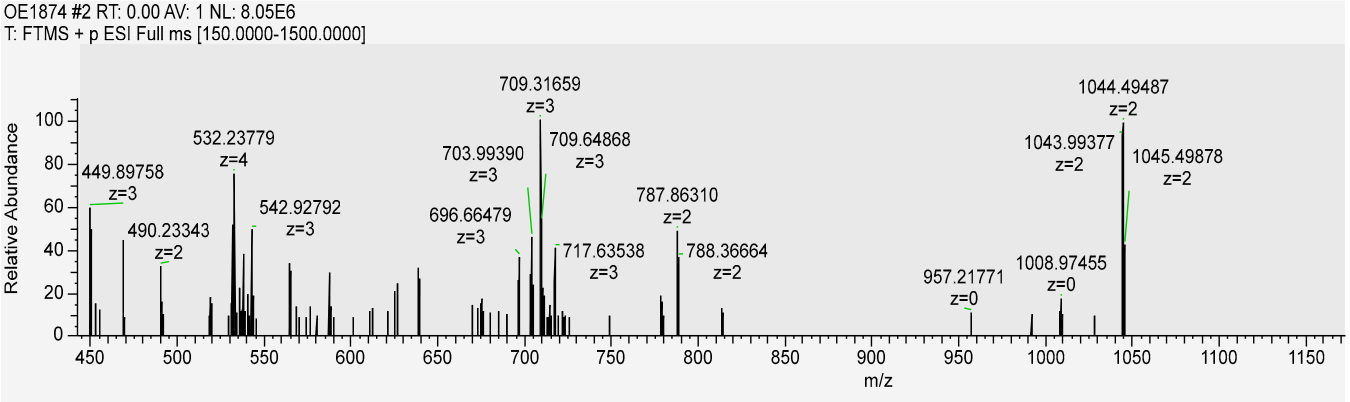

Figure S32. MS spectrum of Fraction 7, obtained after RP-HPLC purification of chymotrypsin digest of PBPs from *Arthrospira platensis*

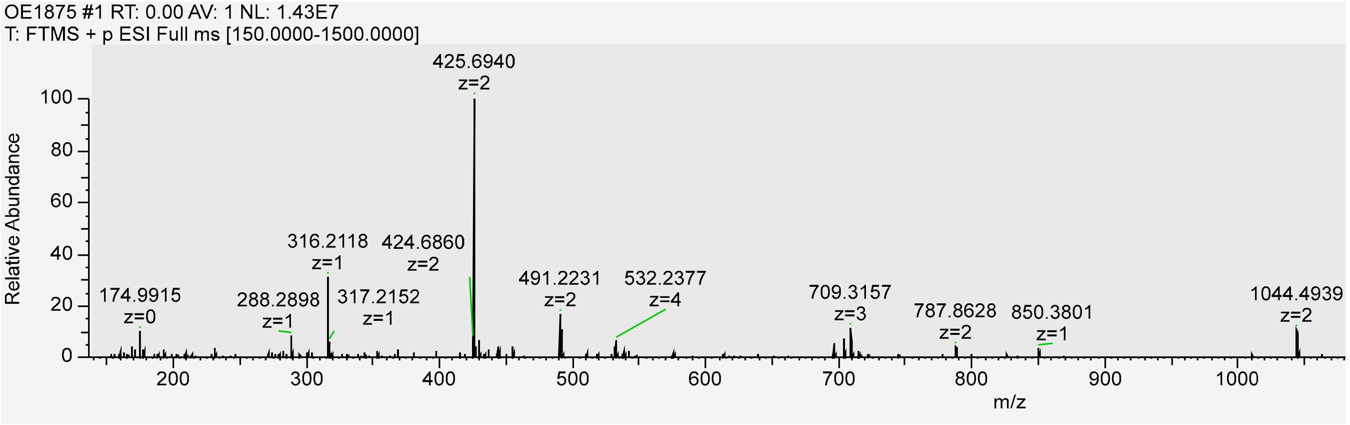

Figure S33. MS spectrum of Fraction 8, obtained after RP-HPLC purification of chymotrypsin digest of PBPs from *Arthrospira platensis*

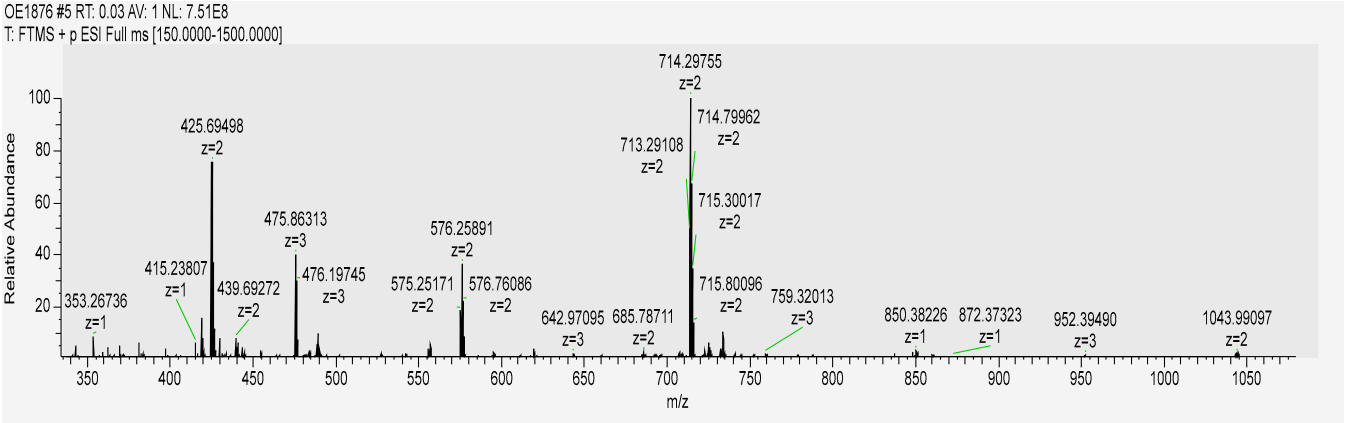

Figure S34. MS spectrum of Fraction 9, obtained after RP-HPLC purification of chymotrypsin digest of PBPs from *Arthrospira platensis*

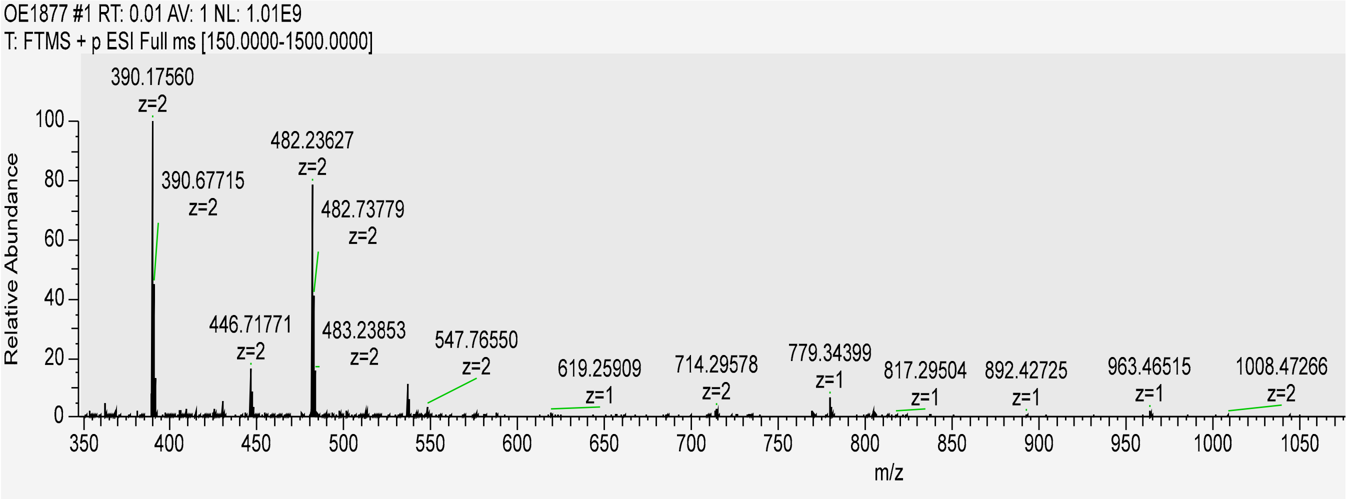

Figure S35. MS spectrum of Fraction 10, obtained after RP-HPLC purification of chymotrypsin digest of PBPs from *Arthrospira platensis*

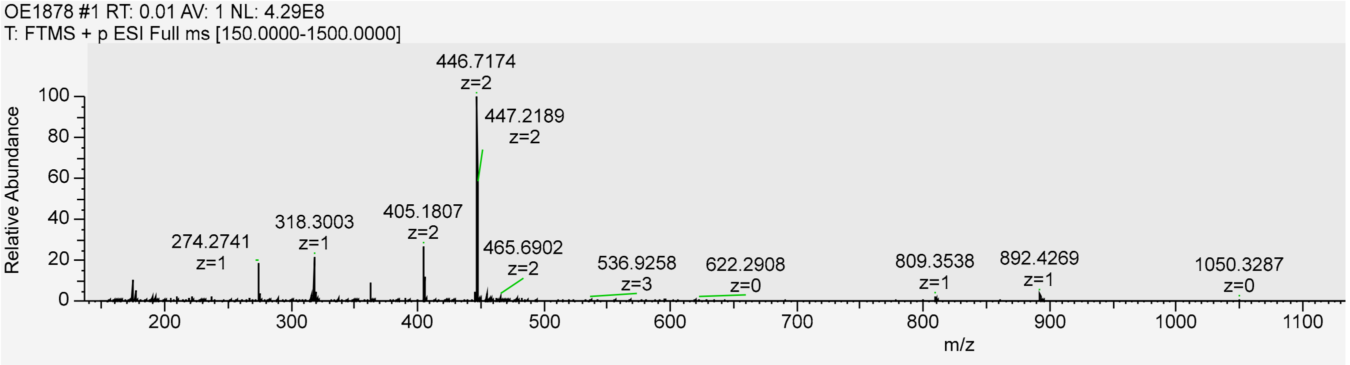

Figure S36. MS spectrum of Fraction 11, obtained after RP-HPLC purification of chymotrypsin digest of PBPs from *Arthrospira platensis*

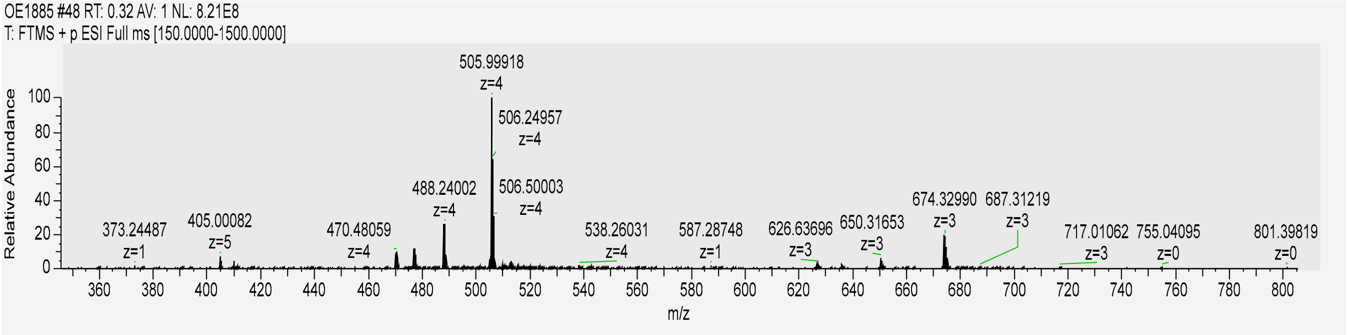

Figure S37. MS spectrum of Fraction 1, obtained after RP-HPLC purification of pepsin digest of PBPs from *Arthrospira platensis*

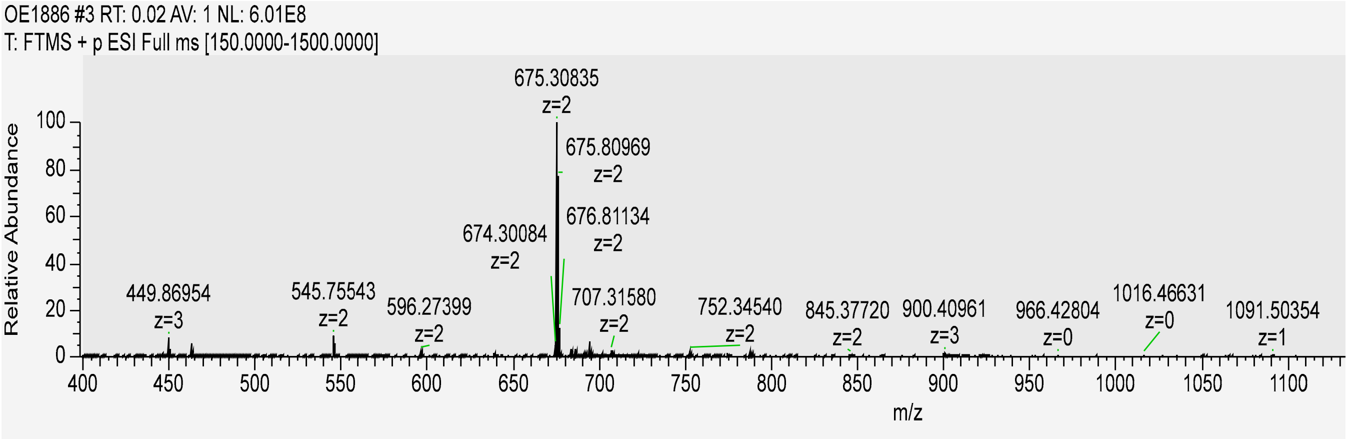

Figure S38. MS spectrum of Fraction 2, obtained after RP-HPLC purification of pepsin digest of PBPs from *Arthrospira platensis*

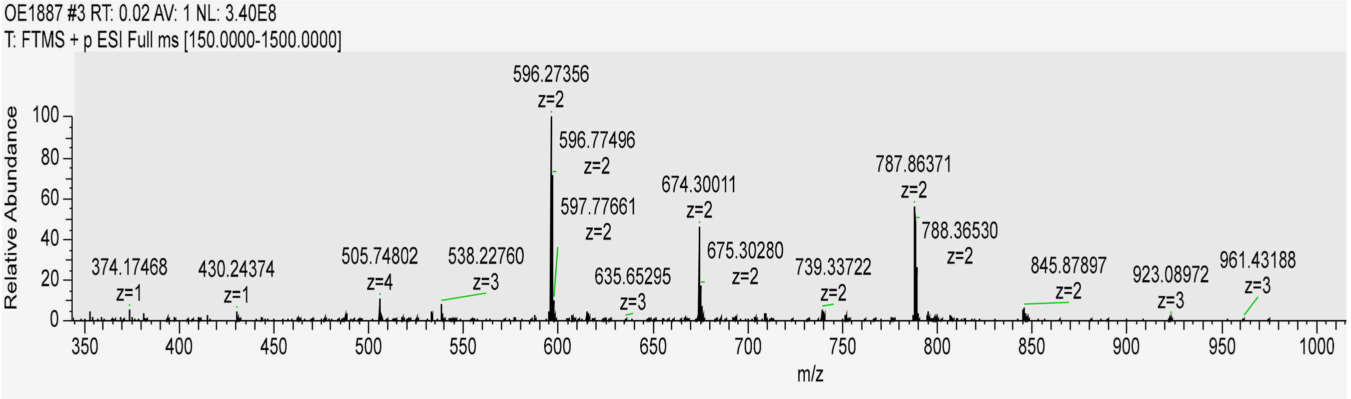

Figure S39. MS spectrum of Fraction 3, obtained after RP-HPLC purification of pepsin digest of PBPs from *Arthrospira platensis*

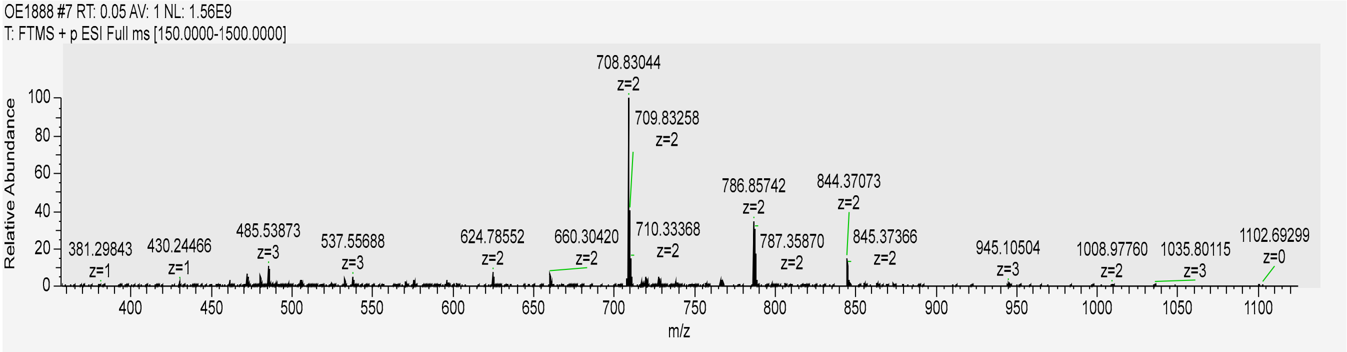

Figure S40. MS spectrum of Fraction 4, obtained after RP-HPLC purification of pepsin digest of PBPs from *Arthrospira platensis*

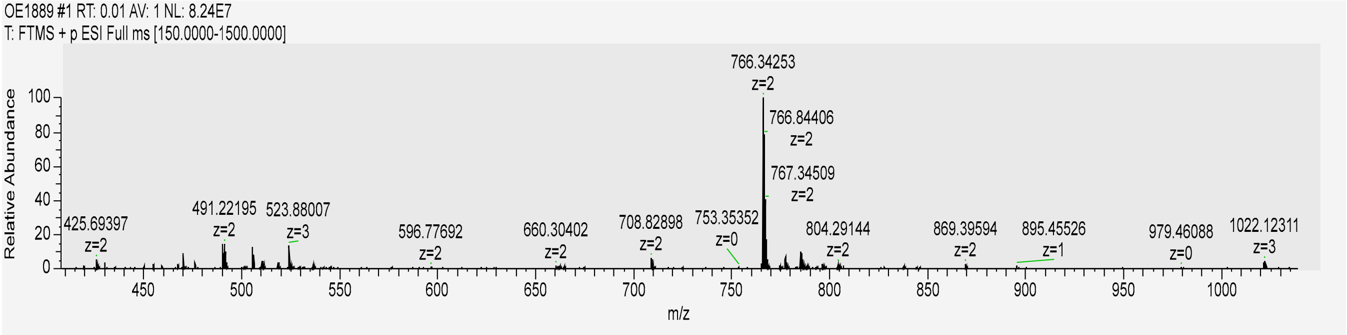

Figure S41. MS spectrum of Fraction 5, obtained after RP-HPLC purification of pepsin digest of PBPs from *Arthrospira platensis*

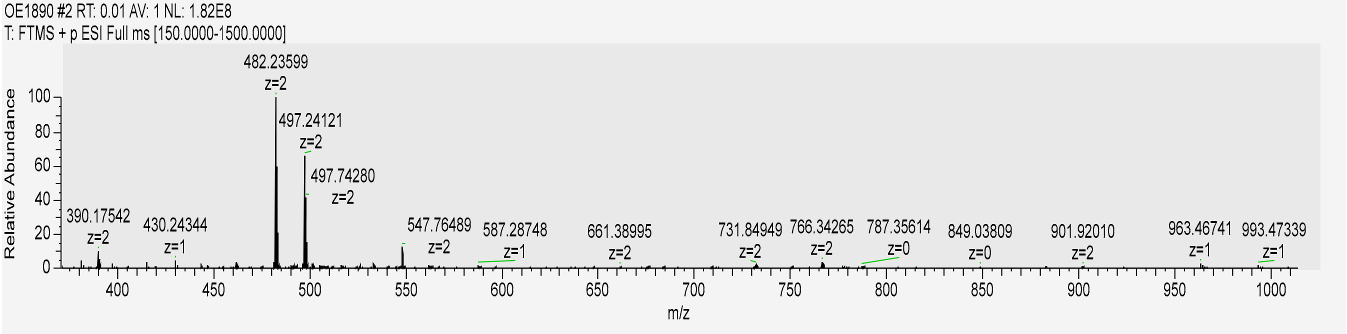

Figure S42. MS spectrum of Fraction 6, obtained after RP-HPLC purification of pepsin digest of PBPs from *Arthrospira platensis*

MS/MS² fragmentation — chymotrypsin digestion, *Porphyra haitanensis*

Table S36. Fragmentation (MS2 spectrum) of a molecular ion with m/z ratio 653.80 (z=2) from the MS spectrum of Fraction 1. Chromopeptide CVPRDM from R-PE α (Q0ZHI7); PEB bound at Cys139.

m/z	z	Exp. mass (Da)	Theor. mass (Da)	Δ mass (Da)	Ion
203.1029	1	202.0951	202.0771	0.018	b_2
300.1552	1	299.1474	299.1298	0.0176	b_3
571.2608	1	570.253	570.2579	-0.0049	b_5
617.2942	1	616.2864	616.2997	-0.0133	y_5
299.1356	?	299.1356	299.1234	0.0122	$C_{17}H_{19}N_2O_3$ (PEB half)
466.1971	?	466.1971	466.1972	-0.0001	$C_{26}H_{32}N_3O_5$ (PEB with one pyrrole ring less)
587.2795	?	587.2795	587.2791	0.0004	PEB

Table S37. Fragmentation (MS2 spectrum) of a molecular ion with m/z ratio 568.29 (z=2) from the MS spectrum of Fraction 2. Chromopeptide AKCAR from R-PC α (Q0ZHI5); PCB bound at Cys84.

m/z	z	Exp. mass (Da)	Theor. mass (Da)	Δ mass (Da)	Ion
200.1392	1	199.1314	199.1315	-0.0001	b_2
374.1817	1	373.1739	373.1778	-0.0039	b_4
477.2565	1	476.2487	476.2524	-0.0037	y_4
297.12	?	297.12	297.1077	0.0123	C17H17N2O3 (PCB half)
464.2102	?	464.2102	464.1816	0.0286	C26H30N3O5 (PCB with one pyrrole ring less)
587.2885	?	587.2885	587.2791	0.00094	PCB

Table S38. Fragmentation (MS2 spectrum) of a molecular ion with m/z ratio 532.77 (z=2) from the MS spectrum of Fraction 2. Chromopeptide KCAR from R-PC α (Q0ZHI5); PCB bound at Cys84.

m/z	z	Exp. mass (Da)	Theor. mass (Da)	Δ mass (Da)	Ion
232.1009	1	231.0931	231.1036	-0.0105	b_2
303.1379	1	302.1301	302.1407	-0.0106	b_3
175.1081	1	174.1003	174.1111	-0.0108	y_1
246.1455	1	245.1377	245.1482	-0.0105	y_2

m/z	z	Exp. mass (Da)	Theor. mass (Da)	Δ mass (Da)	Ion
464.21	?	464.21	464.1816	0.0284	C26H30N3O5 (PCB with one pyrrole ring less)
587.2839	?	587.2885	587.2791	0.0094	PCB

Table S39. Fragmentation (MS2 spectrum) of a molecular ion with m/z ratio 760.39 (z=2) from the MS spectrum of Fraction 3. Chromopeptide NRRMAACL from R-PE α (Q0ZHI7); PEB bound at Cys139.

m/z	z	Exp. mass (Da)	Theor. mass (Da)	Δ mass (Da)	Ion
271.1454	1	270.1376	270.1435	-0.0059	b_2
700.3517	1	699.3439	699.3593	-0.0154	b_6
235.1085	1	234.1007	234.1033	-0.0026	y_2
646.3639	1	645.3561	645.3487	0.0074	c_5
299.1389	?	299.1389	299.1234	0.0155	C17H19N2O3 (PEB half)
466.2005	?	466.2005	466.1972	0.0033	C26H32N3O5 (PEB with one pyrrole ring less)
587.2972	?	587.2972	587.2791	0.0181	PEB

Table S40. Fragmentation (MS2 spectrum) of a molecular ion with m/z ratio 867.39 (z=2) from the MS spectrum of Fraction 3. Chromopeptide YTSRRMAACLRDME from R-PC β (Q0ZHI6); PEB bound at Cys153 (Met oxidised).

m/z	z	Exp. mass (Da)	Theor. mass (Da)	Δ mass (Da)	Ion
866.4313	1	865.4235	865.4223	0.0012	b_7
766.3391	1	765.3313	765.3144	0.0169	y_6
837.3429	1	836.3351	836.3515	-0.0164	y_7
299.1392	?	299.1392	299.1234	0.0158	C17H19N2O3 (PEB half)
466.225	?	466.225	466.1972	0.0278	C26H32N3O5 (PEB with one pyrrole ring less)
587.2888	?	587.2888	587.2791	0.0097	PEB

Table S41. Fragmentation (MS2 spectrum) of a molecular ion with m/z ratio 625.81 (z=2) from the MS spectrum of Fraction 4. Chromopeptide RMAACL / MAACLR from R-PE β / R-PC β (Q0ZHI8 / Q0ZHI6); PEB bound at Cys82.

m/z	z	Exp. mass (Da)	Theor. mass (Da)	Δ mass (Da)	Ion
**288.1348**	**1**	**287.127**	**287.141**	**-0.014**	**b_2**
**132.102**	**1**	**131.0942**	**131.0941**	**0.0001**	**y_1**
**508.245**	**1**	**507.2372**	**507.218**	**0.0192**	**y_5**
**299.1391**	**?**	**299.1391**	**299.1234**	**0.0157**	**C17H19N2O3 (PEB half)**
**466.2252**	**?**	**466.2252**	**466.1972**	**0.028**	**C26H32N3O5 (PEB with one pyrrole ring less)**
**587.2851**	**?**	**587.2851**	**587.2791**	**0.0060**	**PEB**

Table S42. Fragmentation (MS2 spectrum) of a molecular ion with m/z ratio 767.85 (z=2) from the MS spectrum of Fraction 5. Chromopeptide GTPQGDCSAL from R-PE β (Q0ZHI8); PEB bound at Cys158.

m/z	z	Exp. mass (Da)	Theor. mass (Da)	Δ mass (Da)	Ion
**159.0766**	**1**	**158.0688**	**158.0686**	**0.0002**	**b_2**
**441.2096**	**1**	**440.2018**	**440.2014**	**0.0004**	**b_5**
**790.3433**	**1**	**789.3355**	**789.3322**	**0.0033**	**y_8**
**299.139**	**?**	**299.139**	**299.1234**	**0.0156**	**C17H19N2O3 (PEB half)**
**466.2249**	**?**	**466.2249**	**466.1972**	**0.0277**	**C26H32N3O5 (PEB with one pyrrole ring less)**
**587.2888**	**?**	**587.2888**	**587.2791**	**0.0097**	**PEB**

Table S43. Fragmentation (MS2 spectrum) of a molecular ion with m/z ratio 576.26 (z=2) from the MS spectrum of Fraction 6. Chromopeptide GDCSAL from R-PE β (Q0ZHI8); PEB bound at Cys158.

m/z	z	Exp. mass (Da)	Theor. mass (Da)	Δ mass (Da)	Ion
**173.0557**	**1**	**172.0479**	**172.0479**	**0**	**b_2**
**132.1022**	**1**	**131.0944**	**131.0941**	**0.0003**	**y_1**
**203.139**	**1**	**202.1312**	**202.1312**	**0**	**y_2**
**299.1396**	**?**	**299.1396**	**299.1234**	**0.0162**	**C17H19N2O3 (PEB half)**
**466.2261**	**?**	**466.2261**	**466.1972**	**0.0289**	**C26H32N3O5 (PEB with one pyrrole ring less)**
**587.2864**	**?**	**587.2864**	**587.2791**	**0.0073**	**PEB**

Table S44. Fragmentation (MS2 spectrum) of a molecular ion with m/z ratio 755.34 (z=2) from the MS spectrum of Fraction 6. Chromopeptide TADGDCSALA from R-PE β (Q0ZHI8); PEB bound at Cys158.

m/z	z	Exp. mass (Da)	Theor. mass (Da)	Δ mass (Da)	Ion
173.0929	1	172.0851	172.0842	0.0009	b_2
288.1199	1	287.1121	287.1112	0.0009	b_3
203.1395	1	202.1317	202.1312	0.0005	y_2
464.219	1	463.2112	463.2095	0.0017	y_5
299.1404	?	299.1404	299.1234	0.017	C17H19N2O3 (PEB half)
466.2246	?	466.2246	466.1972	0.0274	C26H32N3O5 (PEB with one pyrrole ring less)
587.2885	?	587.2885	587.2791	0.0094	PEB

Table S45. Fragmentation (MS2 spectrum) of a molecular ion with m/z ratio 669.29 (z=2) from the MS spectrum of Fraction 7. Chromopeptide DGDCSALA from R-PE β (Q0ZHI8); PEB bound at Cys158.

m/z	z	Exp. mass (Da)	Theor. mass (Da)	Δ mass (Da)	Ion
173.0558	1	172.048	172.0479	0.0001	b_2
288.0828	1	287.075	287.0748	0.0002	b_3
203.1392	1	202.1314	202.1312	0.0002	y_2
464.2183	1	463.2105	463.2095	0.001	y_5
299.14	?	299.14	299.1234	0.0166	C17H19N2O3 (PEB half)
466.2269	?	466.2269	466.1972	0.0297	C26H32N3O5 (PEB with one pyrrole ring less)
587.2875	?	587.2875	587.2791	0.0084	PEB

Table S46. Fragmentation (MS2 spectrum) of a molecular ion with m/z ratio 874.92 (z=2) from the MS spectrum of Fraction 7. Chromopeptide LTGTPQGDCSAL from R-PC β (Q0ZHI6); PEB bound at Cys153.

m/z	z	Exp. mass (Da)	Theor. mass (Da)	Δ mass (Da)	Ion
215.1391	1	214.1313	214.1312	0.0001	b_2
272.1613	1	271.1535	271.1527	0.0008	b_3
373.2082	1	372.2004	372.2003	0.0001	b_4
790.3437	1	789.3359	789.3322	0.0037	y_8
587.2890	?	587.2890	587.2791	0.0099	PEB

Table S47. Fragmentation (MS2 spectrum) of a molecular ion with m/z ratio 755.34 (z=2) from the MS spectrum of Fraction 7. Chromopeptide TADGDCSALA from R-PE β (Q0ZHI8); PEB bound at Cys158.

m/z	z	Exp. mass (Da)	Theor. mass (Da)	Δ mass (Da)	Ion
173.0925	1	172.0847	172.0842	0.0005	b_2
288.1192	1	287.1114	287.1112	0.0002	b_3
203.139	1	202.1312	202.1312	0	y_2
464.2177	1	463.2099	463.2095	0.0004	y_5
299.1396	?	299.1396	299.1234	0.0162	C17H19N2O3 (PEB half)
466.2263	?	466.2263	466.1972	0.0291	C26H32N3O5 (PEB with one pyrrole ring less)
587.2866	?	587.2866	587.2791	0.0075	PEB

Table S48. Fragmentation (MS2 spectrum) of a molecular ion with m/z ratio 1024.46 (z=2) from the MS spectrum of Fraction 8. Chromopeptide PGEAGDSQEKVNKC from R-PE α (Q0ZHI7); PEB bound at Cys.

m/z	z	Exp. mass (Da)	Theor. mass (Da)	Δ mass (Da)	Ion
155.0817	1	154.0739	154.0737	0.0002	b_2
299.1401	?	299.1401	299.1234	0.0167	C17H19N2O3 (PEB half)
466.2272	?	466.2272	466.1972	0.03	C26H32N3O5 (PEB with one pyrrole ring less)
587.2879	?	587.2879	587.2791	0.0088	PEB

Table S49. Fragmentation (MS2 spectrum) of a molecular ion with m/z ratio 390.18 (z=2) from the MS spectrum of Fraction 8. Chromopeptide AC and/or CA from R-PE β / R-PC α (Q0ZHI8 / Q0ZHI5); PEB bound at Cys82 (R-PE β) or Cys84 (R-PC α).

m/z	z	Exp. mass (Da)	Theor. mass (Da)	Δ mass (Da)	Ion
122.0272	1	121.0194	121.0192	0.0002	y_1
299.1396	?	299.1396	299.1234	0.0162	C17H19N2O3 (PEB/PCB half)
466.2259	?	466.2259	466.1972	0.0287	C26H32N3O5 (PEB with one pyrrole ring less)
587.2859	?	587.2859	587.2791	0.0068	PEB /PCB

MS/MS² fragmentation — pepsin digestion, *Porphyra haitanensis*

Table S50. Fragmentation (MS2 spectrum) of a molecular ion with m/z ratio 726.31 (z=2) from the MS spectrum of Fraction 1. Chromopeptide TGTPQGDCS from R-PC β (Q0ZHI6); PEB bound at Cys153.

m/z	z	Exp. mass (Da)	Theor. mass (Da)	Δ mass (Da)	Ion
159.0763	1	158.0685	158.0686	-0.0001	b2
260.124	1	259.1162	259.1163	-0.0001	b3
485.2351	1	484.2273	484.2276	-0.0003	b5
606.2184	1	605.2106	605.211	-0.0004	y6
299.1388	1	299.1388	299.1396	-0.0008	C17H19N2O3 (PEB half)
466.2329	1	466.2329	466.2342	-0.0013	C26H32N3O5 (PEB with one pyrrole ring less)
587.2859	1	586.278	586.2791	-0.0011	PEB

Table S51. Fragmentation (MS2 spectrum) of a molecular ion with m/z ratio 398.17 (z=2) from the MS spectrum of Fraction 1. Chromopeptide CS from R-PC β (Q0ZHI6); PEB bound at Cys153.

m/z	z	Exp. mass (Da)	Theor. mass (Da)	Δ mass (Da)	Ion
106.0498	1	105.042	105.042	0	y1
299.1389	1	299.1389	299.1396	-0.0007	C17H19N2O3 (PEB half)
466.2324	1	466.2324	466.2342	-0.0018	C26H32N3O5 (PEB with one pyrrole ring less)
587.286	1	586.2782	586.2791	-0.0009	PEB

Table S52. Fragmentation (MS2 spectrum) of a molecular ion with m/z ratio 663.27 (z=2) from the MS spectrum of Fraction 2. Chromopeptide ATADGDCS from R-PE β (Q0ZHI8); PEB bound at Cys158.

m/z	z	Exp. mass (Da)	Theor. mass (Da)	Δ mass (Da)	Ion
173.0921	1	172.0842	172.0842	0	b2
244.1293	1	243.1214	243.1214	0	b3
359.156	1	358.1481	358.1483	-0.0002	b4
531.2042	1	530.1964	530.1967	-0.0003	b6
299.1389	1	299.1389	299.1396	-0.0007	C17H19N2O3 (PEB half)
466.2332	1	466.2332	466.2342	-0.001	C26H32N3O5 (PEB with one pyrrole ring less)

m/z	z	Exp. mass (Da)	Theor. mass (Da)	Δ mass (Da)	Ion
587.2861	1	586.2783	586.2791	-0.0008	PEB

Table S53. Fragmentation (MS2 spectrum) of a molecular ion with m/z ratio 577.23 (z=2) from the MS spectrum of Fraction 2. Chromopeptide ADGDCS from R-PE β (Q0ZHI8); PEB bound at Cys158.

m/z	z	Exp. mass (Da)	Theor. mass (Da)	Δ mass (Da)	Ion
187.0713	1	186.0634	186.0635	-0.0001	b2
244.0928	1	243.085	243.085	0	b3
359.1197	1	358.1118	358.1119	-0.0001	b4
299.1389	1	299.1389	299.1396	-0.0007	C17H19N2O3 (PEB half)
466.2332	1	466.2332	466.2342	-0.001	C26H32N3O5 (PEB with one pyrrole ring less)
587.286	1	586.2782	586.2791	-0.0009	PEB

Table S54. Fragmentation (MS2 spectrum) of a molecular ion with m/z ratio 484.20 (z=2) from the MS spectrum of Fraction 2. Chromopeptide GDCS from R-PE β / R-PC β (Q0ZHI8 / Q0ZHI6); PEB bound at Cys158 / Cys153.

m/z	z	Exp. mass (Da)	Theor. mass (Da)	Δ mass (Da)	Ion
173.0557	1	172.0479	172.0479	0	b2
276.0647	1	275.0569	275.057	-0.0001	b3
106.0498	1	105.042	105.042	0	y1
209.0591	1	208.0512	208.0512	0	y2
299.1389	1	299.1389	299.1396	-0.0007	C17H19N2O3 (PEB/PCB half)
466.2334	1	466.2334	466.2342	-0.0008	C26H32N3O5 (PEB with one pyrrole ring less)
587.286	1	586.2782	586.2791	-0.0009	PEB/PCB

Table S55. Fragmentation (MS2 spectrum) of a molecular ion with m/z ratio 433.69 (z=2) from the MS spectrum of Fraction 2. Chromopeptide CSA from R-PE β / R-PC β (Q0ZHI8 / Q0ZHI6); PEB bound at Cys158 / Cys153.

m/z	z	Exp. mass (Da)	Theor. mass (Da)	Δ mass (Da)	Ion
191.0485	1	190.0407	190.0407	0	b2
177.087	1	176.0792	176.0792	0	y2
299.1388	1	299.1388	299.1396	-0.0008	C17H19N2O3 (PEB/PCB half)

m/z	z	Exp. mass (Da)	Theor. mass (Da)	Δ mass (Da)	Ion
466.2332	1	466.2332	466.2342	-0.001	C26H32N3O5 (PEB with one pyrrole ring less)
587.286	1	586.2782	586.2791	-0.0009	PEB/PCB

Table S56. Fragmentation (MS2 spectrum) of a molecular ion with m/z ratio 455.69 (z=2) from the MS spectrum of Fraction 3. Chromopeptide DCS from R-PE β / R-PC β (Q0ZHI8 / Q0ZHI6); PEB bound at Cys158 / Cys153.

m/z	z	Exp. mass (Da)	Theor. mass (Da)	Δ mass (Da)	Ion
219.0435	1	218.0357	218.0356	0.0001	b2
106.0498	1	105.042	105.042	0	y1
209.0592	1	208.0513	208.0512	0.0001	y2
299.139	1	299.139	299.1396	-0.0006	C17H19N2O3 (PEB/PCB half)
466.2333	1	466.2333	466.2342	-0.0009	C26H32N3O5 (PEB with one pyrrole ring less)
587.2859	1	586.2781	586.2791	-0.001	PEB/PCB

Table S57. Fragmentation (MS2 spectrum) of a molecular ion with m/z ratio 575.27 (z=3) from the MS spectrum of Fraction 3. Chromopeptide RMAACLRDGE from R-PE β (Q0ZHI8); PEB bound at Cys82 (Met oxidised).

m/z	z	Exp. mass (Da)	Theor. mass (Da)	Δ mass (Da)	Ion
359.1923	1	358.1845	358.1782	0.0063	b3
589.2932	1	588.2854	588.2862	-0.0008	y5
692.3109	1	691.3031	691.2954	0.0077	y6
299.139	1	299.139	299.1396	-0.0006	C17H19N2O3 (PEB half)
466.2318	1	466.2318	466.2342	-0.0024	C26H32N3O5 (PEB with one pyrrole ring less)
587.2862	1	586.2784	586.2791	-0.0007	PEB

Table S58. Fragmentation (MS2 spectrum) of a molecular ion with m/z ratio 782.85 (z=2) from the MS spectrum of Fraction 3. Chromopeptide LTGTPQGDCS from R-PC β (Q0ZHI6); PEB bound at Cys153.

m/z	z	Exp. mass (Da)	Theor. mass (Da)	Δ mass (Da)	Ion
215.1391	1	214.1312	214.1312	0	b2
272.1606	1	271.1527	271.1527	0	b3
373.2081	1	372.2003	372.2003	0	b4

m/z	z	Exp. mass (Da)	Theor. mass (Da)	Δ mass (Da)	Ion
606.2187	1	605.2108	605.211	-0.0002	y6
299.1389	1	299.1389	299.1396	-0.0007	C17H19N2O3 (PEB half)
466.233	1	466.233	466.2342	-0.0012	C26H32N3O5 (PEB with one pyrrole ring less)
587.2861	1	586.2783	586.2791	-0.0008	PEB

Table S59. Fragmentation (MS2 spectrum) of a molecular ion with m/z ratio 698.79 (z=2) from the MS spectrum of Fraction 3 Chromopeptide ATADGDCSA from R-PE β (Q0ZHI8); PEB bound at Cys158.

m/z	z	Exp. mass (Da)	Theor. mass (Da)	Δ mass (Da)	Ion
173.0921	1	172.0842	172.0842	0	b2
359.1565	1	358.1486	358.1483	0.0003	b4
416.1765	1	415.1687	415.1698	-0.0011	b5
177.087	1	176.0791	176.0792	-0.0001	y2
299.1391	1	299.1391	299.1396	-0.0005	C17H19N2O3 (PEB half)
466.2332	1	466.2332	466.2342	-0.001	C26H32N3O5 (PEB with one pyrrole ring less)
587.2861	1	586.2783	586.2791	-0.0008	PEB

Table S60. Fragmentation (MS2 spectrum) of a molecular ion with m/z ratio 404.19 (z=2) from the MS spectrum of Fraction 4. Chromopeptide CV from R-PE α (Q0ZHI7); PEB bound at Cys139.

m/z	z	Exp. mass (Da)	Theor. mass (Da)	Δ mass (Da)	Ion
118.0862	1	117.0784	117.0784	0	y1 (V)
299.1391	1	299.1391	299.1396	-0.0005	C17H19N2O3 (PEB half)
466.2325	1	466.2325	466.2342	-0.0017	C26H32N3O5 (PEB with one pyrrole ring less)
587.2862	1	586.2784	586.2791	-0.0007	PEB

Table S61. Fragmentation (MS2 spectrum) of a molecular ion with m/z ratio 490.23 (z=2) from the MS spectrum of Fraction 5. Chromopeptide CSAL from R-PE β / R-PC β (Q0ZHI8 / Q0ZHI6); PEB bound at Cys158 / Cys153.

m/z	z	Exp. mass (Da)	Theor. mass (Da)	Δ mass (Da)	Ion
191.0484	1	190.0406	190.0407	-0.0001	b2
262.0855	1	261.0777	261.0778	-0.0001	b3

m/z	z	Exp. mass (Da)	Theor. mass (Da)	Δ mass (Da)	Ion
132.1017	1	131.0939	131.0941	-0.0002	y1
203.1388	1	202.131	202.1312	-0.0002	y2
290.1709	1	289.163	289.1632	-0.0002	y3
299.1388	1	299.1388	299.1396	-0.0008	C17H19N2O3 (PEB/PCB half)
466.233	1	466.2330	466.2342	-0.0012	C26H32N3O5 (PEB with one pyrrole ring less)
587.2858	1	586.278	586.2791	-0.0013	PEB/PCB

Table S62. Fragmentation (MS2 spectrum) of a molecular ion with m/z ratio 482.24 (z=2) from the MS spectrum of Fraction 6. Chromopeptide AACL from R-PE β (Q0ZHI8) / R-PC β (Q0ZHI6); chromophore at Cys82 — PEB (R-PE β) or PCB (R-PC β).

m/z	z	Exp. mass (Da)	Theor. mass (Da)	Δ mass (Da)	Ion
143.0814	1	142.0736	142.0737	-0.0001	b2
246.0906	1	245.0827	245.0829	-0.0002	b3
132.1018	1	131.094	131.0941	-0.0001	y1
235.1109	1	234.1031	234.1033	-0.0002	y2
464.2175	1	464.2175	464.2185	-0.001	C26H30N3O5 (PCB with one pyrrole ring less)
587.2857	1	587.2857	587.2791	-0.0066	PEB/PCB
299.1388	1	299.1388	299.1396	-0.0008	C17H19N2O3 (PEB/PCB half)
466.2332	1	466.2332	466.2342	-0.001	C26H32N3O5 (PEB with one pyrrole ring less)

Table S63. Fragmentation (MS2 spectrum) of a molecular ion with m/z ratio 446.72 (z=2) from the MS spectrum of Fraction 6. Chromopeptide ACL from R-PE β (Q0ZHI8) / R-PC β (Q0ZHI6); chromophore at Cys82 — PEB (R-PE β) or PCB (R-PC β).

m/z	z	Exp. mass (Da)	Theor. mass (Da)	Δ mass (Da)	Ion
175.0536	1	174.0457	174.0458	-0.0001	b2
132.1018	1	131.094	131.0941	-0.0001	y1
235.1097	1	234.1019	234.1033	-0.0014	y2
464.2175	1	464.2175	464.2185	-0.001	C26H30N3O5 (PCB with one pyrrole ring less)
299.1388	1	299.1388	299.1396	-0.0008	C17H19N2O3 (PEB/PCB half)

m/z	z	Exp. mass (Da)	Theor. mass (Da)	Δ mass (Da)	Ion
**466.2333**	**1**	**466.2333**	**466.2342**	**-0.0009**	**C26H32N3O5 (PEB with one pyrrole ring less)**
**587.2859**	**1**	**586.2781**	**586.2791**	**-0.001**	**PEB/PCB**

Table S64. Fragmentation (MS2 spectrum) of a molecular ion with m/z ratio 411.20 (z=2) from the MS spectrum of Fraction 6. Chromopeptide CL from R-PE β (Q0ZHI8) / R-PC β (Q0ZHI6); chromophore at Cys82 — PEB (R-PE β) or PCB (R-PC β).

m/z	z	Exp. mass (Da)	Theor. mass (Da)	Δ mass (Da)	Ion
**132.1018**	**1**	**131.0939**	**131.0941**	**-0.0002**	**y1**
**464.2175**	**1**	**464.2175**	**464.2185**	**-0.001**	**C26H30N3O5 (PCB with one pyrrole ring less)**
**299.1389**	**1**	**299.1389**	**299.1396**	**-0.0007**	**C17H19N2O3 (PEB/PCB half)**
**466.2322**	**1**	**466.2322**	**466.2342**	**-0.002**	**C26H32N3O5 (PEB with one pyrrole ring less)**
**587.2859**	**1**	**587.2859**	**587.2791**	**0.0068**	**PEB/PCB**

MS/MS² fragmentation — proteinase K digestion, *Porphyra haitanensis*

Table S65. Fragmentation (MS2 spectrum) of a molecular ion with m/z ratio 726.84 (z=2) from the MS spectrum of Fraction 1. Chromopeptide GDCSALVAE from R-PC β (Q0ZHI6); PEB bound at Cys153.

m/z	z	Exp. mass (Da)	Theor. mass (Da)	Δ mass (Da)	Ion
276.0653	1	275.0574	275.0570	+0.0004	b3
547.2102	1	546.2024	546.2102	−0.0078	b6
431.2502	1	430.2424	430.2422	+0.0002	y4
589.3025	1	588.2946	588.3113	−0.0167	y6
464.2202	1	464.2202	464.2213	−0.0011	C26H30N3O5 (PCB with one pyrrole ring less)
587.2856	1	587.2856	587.2879	−0.0023	PCB/PEB
466.2348	1	466.2348	466.2361	−0.0013	C26H32N3O5 (PEB with one pyrrole ring less)

Table S66. Fragmentation (MS2 spectrum) of a molecular ion with m/z ratio 670.30 (z=2) from the MS spectrum of Fraction 1. Chromopeptide ADGDCSAL from R-PE β (Q0ZHI8); PEB bound at Cys158.

m/z	z	Exp. mass (Da)	Theor. mass (Da)	Δ mass (Da)	Ion
187.0714	1	186.0635	186.0635	0.0000	b2
244.0936	1	243.0858	243.085	+0.0008	b3
508.218	1	507.2101	507.1994	+0.0107	y5
464.2201	1	464.2201	464.2213	−0.0012	C26H30N3O5 (PCB with one pyrrole ring less)
587.2856	1	587.2856	587.2879	−0.0023	PCB/PEB
466.2317	1	466.2317	466.2361	−0.0044	C26H32N3O5 (PEB with one pyrrole ring less)

Table S67. Fragmentation (MS2 spectrum) of a molecular ion with m/z ratio 613.75 (z=2) from the MS spectrum of Fraction 1. Chromopeptide ADGDCSA from R-PE β (Q0ZHI8); PEB bound at Cys158.

m/z	z	Exp. mass (Da)	Theor. mass (Da)	Δ mass (Da)	Ion
187.0714	1	186.0635	186.0635	0.0000	b2
244.0936	1	243.0858	243.085	+0.0008	b3
177.0874	1	176.0796	176.0792	+0.0004	y2

m/z	z	Exp. mass (Da)	Theor. mass (Da)	Δ mass (Da)	Ion
464.2203	1	464.2203	464.2213	−0.0010	C26H30N3O5 (PCB with one pyrrole ring less)
587.2859	1	587.2859	587.2879	−0.0020	PCB/PEB
466.235	1	466.235	466.2361	−0.0011	C26H32N3O5 (PEB with one pyrrole ring less)

Table S68 Fragmentation (MS2 spectrum) of a molecular ion with m/z ratio 509.77 (z=2) from the MS spectrum of Fraction 1. Chromopeptide LCVP from R-PE α (Q0ZHI7); PEB bound at Cys139.

m/z	z	Exp. mass (Da)	Theor. mass (Da)	Δ mass (Da)	Ion
217.0969	1	216.0891	216.0927	−0.0036	b2
316.1662	1	315.1584	315.1611	−0.0027	b3
116.0707	1	115.0629	115.0628	+0.0001	y1
215.1395	1	214.1317	214.1312	+0.0005	y2
318.1545	1	317.1467	317.1404	+0.0063	y3
587.2856	1	587.2856	587.2879	−0.0023	PCB/PEB

Table S69. Fragmentation (MS2 spectrum) of a molecular ion with m/z ratio 534.23 (z=2) from the MS spectrum of Fraction 2. Chromopeptide SNASC from R-PE β (Q0ZHI8); PUB bound at Cys50.

m/z	z	Exp. mass (Da)	Theor. mass (Da)	Δ mass (Da)	Ion
273.1274	1	272.1196	272.1115	+0.0081	b3
209.059	1	208.0511	208.0512	−0.0001	y2
589.2950	?	588.2872	588.2948	-0.0076	PUB

Table S70. Fragmentation (MS2 spectrum) of a molecular ion with m/z ratio 441.69 (z=2) from the MS spectrum of Fraction 2. Chromopeptide GDC from R-PE β (Q0ZHI8); PEB bound at Cys158.

m/z	z	Exp. mass (Da)	Theor. mass (Da)	Δ mass (Da)	Ion
173.0555	1	172.0477	172.0479	−0.0002	b2
122.0271	1	121.0193	121.0192	+0.0001	y1
464.2186	1	464.2186	464.2213	−0.0027	C26H30N3O5 (PCB with one pyrrole ring less)
587.2876	1	587.2876	587.2879	−0.0003	PCB/PEB

m/z	z	Exp. mass (Da)	Theor. mass (Da)	Δ mass (Da)	Ion
**466.2332**	**1**	**466.2332**	**466.2361**	**−0.0029**	**C26H32N3O5 (PEB with one pyrrole ring less)**

Table S71. Fragmentation (MS2 spectrum) of a molecular ion with m/z ratio 484.20 (z=2) from the MS spectrum of Fraction 3. Chromopeptide GDCS from R-PE β (Q0ZHI8); PEB bound at Cys158.

m/z	z	Exp. mass (Da)	Theor. mass (Da)	Δ mass (Da)	Ion
**173.0558**	**1**	**172.0479**	**172.0479**	**0.0000**	**b2**
**276.0652**	**1**	**275.0574**	**275.057**	**+0.0004**	**b3**
**106.0502**	**1**	**105.0424**	**105.042**	**+0.0004**	**y1**
**209.0592**	**1**	**208.0514**	**208.0512**	**+0.0002**	**y2**
**324.0864**	**1**	**323.0786**	**323.0782**	**+0.0004**	**y3**
**464.2174**	**1**	**464.2174**	**464.2213**	**−0.0039**	**C26H30N3O5 (PCB with one pyrrole ring less)**
**587.2861**	**1**	**587.2861**	**587.2879**	**−0.0018**	**PCB/PEB**
**466.2351**	**1**	**466.2351**	**466.2361**	**−0.0010**	**C26H32N3O5 (PEB with one pyrrole ring less)**

Table S72. Fragmentation (MS2 spectrum) of a molecular ion with m/z ratio 519.72 (z=2) from the MS spectrum of Fraction 4. Chromopeptide GDCSA from R-PE β (Q0ZHI8); PEB bound at Cys158.

m/z	z	Exp. mass (Da)	Theor. mass (Da)	Δ mass (Da)	Ion
**173.0558**	**1**	**172.048**	**172.0479**	**+0.0001**	**b2**
**276.0654**	**1**	**275.0575**	**275.057**	**+0.0005**	**b3**
**177.0875**	**1**	**176.0797**	**176.0792**	**+0.0005**	**y2**
**280.0968**	**1**	**279.089**	**279.0883**	**+0.0007**	**y3**
**464.2178**	**1**	**464.2178**	**464.2213**	**−0.0035**	**C26H30N3O5 (PCB with one pyrrole ring less)**
**587.2865**	**1**	**587.2865**	**587.2879**	**−0.0014**	**PCB/PEB**
**466.2354**	**1**	**466.2354**	**466.2361**	**−0.0007**	**C26H32N3O5 (PEB with one pyrrole ring less)**

Table S73. Fragmentation (MS2 spectrum) of a molecular ion with m/z ratio 577.23 (z=2) from the MS spectrum of Fraction 4. Chromopeptide DGDCSA from R-PE β (Q0ZHI8); PEB bound at Cys158.

m/z	z	Exp. mass (Da)	Theor. mass (Da)	Δ mass (Da)	Ion
**173.0561**	**1**	**172.0483**	**172.0479**	**+0.0004**	**b2**
**288.0832**	**1**	**287.0753**	**287.0748**	**+0.0005**	**b3**
**177.0871**	**1**	**176.0793**	**176.0792**	**+0.0001**	**y2**
**464.2187**	**1**	**464.2187**	**464.2213**	**−0.0026**	**C26H30N3O5 (PCB with one pyrrole ring less)**
**587.2877**	**1**	**587.2877**	**587.2879**	**−0.0002**	**PCB/PEB**
**466.2333**	**1**	**466.2333**	**466.2361**	**−0.0028**	**C26H32N3O5 (PEB with one pyrrole ring less)**

Table S74. Fragmentation (MS2 spectrum) of a molecular ion with m/z ratio 726.84 (z=2) from the MS spectrum of Fraction 4. Chromopeptide GDCSALVAE from R-PC β (Q0ZHI6); PCB bound at Cys153.

m/z	z	Exp. mass (Da)	Theor. mass (Da)	Δ mass (Da)	Ion
**173.0558**	**1**	**172.048**	**172.0479**	**+0.0001**	**b2**
**288.0828**	**1**	**287.075**	**287.0748**	**+0.0002**	**b3**
**464.218**	**1**	**464.218**	**464.2213**	**−0.0033**	**C26H30N3O5 (PCB with one pyrrole ring less)**
**587.2869**	**1**	**587.2869**	**587.2879**	**−0.0010**	**PCB/PEB**
**466.2357**	**1**	**466.2357**	**466.2361**	**−0.0004**	**C26H32N3O5 (PEB with one pyrrole ring less)**

Table S75. Fragmentation (MS2 spectrum) of a molecular ion with m/z ratio 455.69 (z=2) from the MS spectrum of Fraction 4. Chromopeptide DCS from R-PE β (Q0ZHI8); PEB bound at Cys158.

m/z	z	Exp. mass (Da)	Theor. mass (Da)	Δ mass (Da)	Ion
**219.0441**	**1**	**218.0363**	**218.0356**	**+0.0007**	**b2**
**106.0502**	**1**	**105.0423**	**105.042**	**+0.0003**	**y1**
**464.2194**	**1**	**464.2194**	**464.2213**	**−0.0019**	**C26H30N3O5 (PCB with one pyrrole ring less)**
**587.2887**	**1**	**587.2887**	**587.2879**	**+0.0008**	**PCB/PEB**
**466.234**	**1**	**466.234**	**466.2361**	**−0.0021**	**C26H32N3O5 (PEB with one pyrrole ring less)**

Table S76. Fragmentation (MS2 spectrum) of a molecular ion with m/z ratio 625.84 (z=2) from the MS spectrum of Fraction 4. Chromopeptide RDRLC from R-PE α (Q0ZHI7); PEB bound at Cys139.

m/z	z	Exp. mass (Da)	Theor. mass (Da)	Δ mass (Da)	Ion
391.2124	1	390.2046	390.2044	+0.0002	y3
541.339	1	540.3312	540.3127	+0.0185	b4
464.2178	1	464.2178	464.2213	−0.0035	$C_{26}H_{30}N_3O_5$ (PCB with one pyrrole ring less)
587.2866	1	587.2866	587.2879	−0.0013	PCB/PEB

Table S77. Fragmentation (MS2 spectrum) of a molecular ion with m/z ratio 541.71 (z=2) from the MS spectrum of Fraction 4. Chromopeptide DGDCS from R-PE β (Q0ZHI8); PEB bound at Cys158.

m/z	z	Exp. mass (Da)	Theor. mass (Da)	Δ mass (Da)	Ion
173.0559	1	172.048	172.0479	+0.0001	b2
288.0828	1	287.075	287.0748	+0.0002	b3
464.218	1	464.218	464.2213	−0.0033	$C_{26}H_{30}N_3O_5$ (PCB with one pyrrole ring less)
587.2869	1	587.2869	587.2879	−0.0010	PCB/PEB
466.2357	1	466.2357	466.2361	−0.0004	$C_{26}H_{32}N_3O_5$ (PEB with one pyrrole ring less)

Table S78. Fragmentation (MS2 spectrum) of a molecular ion with m/z ratio 653.80 (z=2) from the MS spectrum of Fraction 5. Chromopeptide CVPRDM from R-PE α (Q0ZHI7); PEB bound at Cys139.

m/z	z	Exp. mass (Da)	Theor. mass (Da)	Δ mass (Da)	Ion
203.1069	1	202.0991	202.0771	+0.0220	b2
300.143	1	299.1351	299.1298	+0.0053	b3
456.2393	1	455.2315	455.2309	+0.0006	b4
571.2665	1	570.2587	570.2579	+0.0008	b5
421.1884	1	420.1806	420.1786	+0.0020	y3
617.3082	1	616.3004	616.2997	+0.0007	y5
587.2877	1	587.2877	587.2879	−0.0002	PCB/PEB

MS/MS² fragmentation — chymotrypsin digestion, *Arthrospira platensis*

Table S79. Fragmentation (MS2 spectrum) of a molecular ion with m/z ratio 559.94 (z=3) from the MS spectrum of Fraction 1. Chromopeptide ADQRGKDKCA from C-PC α (P72509); PCB bound at Cys84.

m/z	z	Exp. mass (Da)	Theor. mass (Da)	Δ mass (Da)	Ion
187.0713	1	186.0635	186.0635	0	b2
656.3487	1	655.3409	655.3396	0.0013	b6
771.3775	1	770.3697	770.3666	0.0031	b7
905.4623	1	904.4545	904.4543	0.0002	y8
464.2176	1	464.2176	464.2185	-0.0009	C26H30N3O5 (PCB with one pyrrole ring less)
587.2865	1	587.2865	587.2879	0.0014	PCB

Table S80. Fragmentation (MS2 spectrum) of a molecular ion with m/z ratio 437.96 (z=4) from the MS spectrum of Fraction 1. Chromopeptide AADQRGKDKCA from C-PC α (P72509); PCB bound at Cys84.

m/z	z	Exp. mass (Da)	Theor. mass (Da)	Δ mass (Da)	Ion
258.1086	1	257.1008	257.1006	0.0002	b3
905.4654	1	904.4576	904.4543	0.0033	y8
1020.4927	1	1019.4849	1019.4813	0.0036	y9
1091.5227	1	1090.5148	1090.5184	-0.0036	y10
464.2188	1	464.2188	464.2185	0.0003	C26H30N3O5 (PCB with one pyrrole ring less)
587.2894	1	587.2894	587.2879	0.0015	PCB

Table S81. Fragmentation (MS2 spectrum) of a molecular ion with m/z ratio 403.20 (z=3) from the MS spectrum of Fraction 2. Chromopeptide GKDKCA from C-PC α (P72509); PCB bound at Cys84.

m/z	z	Exp. mass (Da)	Theor. mass (Da)	Δ mass (Da)	Ion
186.1238	1	185.116	185.1159	0.0001	b2
301.1511	1	300.1433	300.1428	0.0005	b3
436.1851	1	435.1773	435.1782	-0.0009	y4
429.2457	1	428.2379	428.2378	0.0001	b4

m/z	z	Exp. mass (Da)	Theor. mass (Da)	Δ mass (Da)	Ion
464.2189	1	464.2189	464.2185	0.0004	C26H30N3O5 (PCB with one pyrrole ring less)
587.2881	1	587.2881	587.2879	0.0002	PCB

Table S82. Fragmentation (MS2 spectrum) of a molecular ion with m/z ratio 560.29 (z=2) from the MS spectrum of Fraction 3. Chromopeptide AACLR from C-PC β (P72508); PCB bound at Cys82.

m/z	z	Exp. mass (Da)	Theor. mass (Da)	Δ mass (Da)	Ion
143.0817	1	142.0739	142.0737	0.0002	b2
246.091	1	245.0832	245.0829	0.0003	b3
175.1195	1	174.1117	174.1111	0.0006	y1
464.2177	1	464.2177	464.2185	-0.0008	C26H30N3O5 (PCB with one pyrrole ring less)
587.2857	1	587.2857	587.2879	0.0022	PCB

Table S83. Fragmentation (MS2 spectrum) of a molecular ion with m/z ratio 484.20 (z=2) from the MS spectrum of Fraction 3. Chromopeptide GDCS from C-PC β (P72508); PCB bound at Cys153.

m/z	z	Exp. mass (Da)	Theor. mass (Da)	Δ mass (Da)	Ion
173.0558	1	172.048	172.0479	0.0001	b2
276.0651	1	275.0573	275.057	0.0003	b3
106.0501	1	105.0423	105.042	0.0003	y1
209.0591	1	208.0513	208.0512	0.0001	y2
464.2171	1	464.2171	464.2185	-0.0014	C26H30N3O5 (PCB with one pyrrole ring less)
587.2894	1	587.2894	587.2879	0.0015	PCB

Table S84. Fragmentation (MS2 spectrum) of a molecular ion with m/z ratio 519.72 (z=2) from the MS spectrum of Fraction 4. Chromopeptide GDCSA from C-PC β (P72508); PCB bound at Cys153.

m/z	z	Exp. mass (Da)	Theor. mass (Da)	Δ mass (Da)	Ion
173.0559	1	172.0481	172.0479	0.0002	b2
276.0653	1	275.0575	275.057	0.0005	b3
177.0869	1	176.0791	176.0792	-0.0001	y2
464.2186	1	464.2186	464.2185	0.0001	C26H30N3O5 (PCB with one pyrrole ring less)
587.2877	1	587.2877	587.2879	-0.0002	PCB

Table S85. Fragmentation (MS2 spectrum) of a molecular ion with m/z ratio 455.88 (z=3) from the MS spectrum of Fraction 4. Chromopeptide AACLRDM from C-PC β (P72508); PCB bound at Cys82.

m/z	z	Exp. mass (Da)	Theor. mass (Da)	Δ mass (Da)	Ion
143.0818	1	142.074	142.0737	0.0003	b2
246.0913	1	245.0835	245.0829	0.0006	b3
421.1876	1	420.1798	420.1786	0.0012	y4
464.2189	1	464.2189	464.2185	0.0004	C26H30N3O5 (PCB with one pyrrole ring less)
587.2883	1	587.2883	587.2879	-0.0004	PCB

Table S86. Fragmentation (MS2 spectrum) of a molecular ion with m/z ratio 675.31 (z=2) from the MS spectrum of Fraction 5. Chromopeptide TPGDCSAL from C-PC β (P72508); PCB bound at Cys153.

m/z	z	Exp. mass (Da)	Theor. mass (Da)	Δ mass (Da)	Ion
199.1079	1	198.1001	198.0999	0.0002	b2
256.1293	1	255.1215	255.1214	0.0001	b3
371.1565	1	370.1487	370.1483	0.0004	b4
464.2188	1	464.2188	464.2185	0.0003	C26H30N3O5 (PCB with one pyrrole ring less)
587.2882	1	587.2882	587.2879	0.0003	PCB

Table S87. Fragmentation (MS2 spectrum) of a molecular ion with m/z ratio 902.40 (z=2) from the MS spectrum of Fraction 5. Chromopeptide NDPAGITPGDCSA from C-PC β (P72508); PCB bound at Cys153.

m/z	z	Exp. mass (Da)	Theor. mass (Da)	Δ mass (Da)	Ion
230.0773	1	229.0695	229.0693	0.0002	b2
327.1303	1	326.1225	326.1221	0.0004	b3
464.2195	1	464.2195	464.2185	0.001	C26H30N3O5 (PCB with one pyrrole ring less)
587.2849	1	587.2849	587.2879	0.0030	PCB

Table S88. Fragmentation (MS2 spectrum) of a molecular ion with m/z ratio 731.85 (z=2) from the MS spectrum of Fraction 5. Chromopeptide AACIRDLD from APC β (P72505); PCB bound at Cys81.

m/z	z	Exp. mass (Da)	Theor. mass (Da)	Δ mass (Da)	Ion
630.3025	1	629.2947	629.295	-0.0003	b6
743.3892	1	742.3814	742.379	0.0024	b7
631.3438	1	630.336	630.3331	0.0029	y5
734.3544	1	733.3466	733.3423	0.0043	y6
464.2197	1	464.2197	464.2185	0.0012	C26H30N3O5 (PCB with one pyrrole ring less)
587.2895	1	587.2895	587.2879	0.0016	PCB

Table S89. Fragmentation (MS2 spectrum) of a molecular ion with m/z ratio 722.30 (z=2) from the MS spectrum of Fraction 6. Chromopeptide GEEMTATC (Met-ox) from APC α (P72504); PCB bound at Cys81.

m/z	z	Exp. mass (Da)	Theor. mass (Da)	Δ mass (Da)	Ion
187.0716	1	186.0638	186.0635	0.0003	b2
316.1147	1	315.1069	315.1061	0.0008	b3
619.2505	1	618.2426	618.2314	0.0112	b6
464.2188	1	464.2188	464.2185	0.0003	C26H30N3O5 (PCB with one pyrrole ring less)
587.2881	1	587.2881	587.2879	0.0002	PCB

Table S90. Fragmentation (MS2 spectrum) of a molecular ion with m/z ratio 952.43 (z=2) from the MS spectrum of Fraction 6. Chromopeptide MTATCLRDLDY (Met-ox) from APC α (P72504); PCB bound at Cys81.

m/z	z	Exp. mass (Da)	Theor. mass (Da)	Δ mass (Da)	Ion
621.277	1	620.2692	620.2657	0.0035	b6
464.2192	1	464.2192	464.2185	0.0007	C26H30N3O5 (PCB with one pyrrole ring less)
587.2887	1	587.2887	587.2879	0.0008	PCB

Table S91. Fragmentation (MS2 spectrum) of a molecular ion with m/z ratio 1044.49 (z=2) from the MS spectrum of Fraction 7. Chromopeptide AIVNDPAGITPGDCSA from C-PC β (P72508); PCB bound at Cys153.

m/z	z	Exp. mass (Da)	Theor. mass (Da)	Δ mass (Da)	Ion
185.1285	1	184.1207	184.1206	0.0001	b2
284.1969	1	283.1891	283.189	0.0001	b3
513.2668	1	512.259	512.2589	0.0001	b5
738.3815	1	737.3737	737.3703	0.0034	b8
464.2188	1	464.2188	464.2185	0.0003	C26H30N3O5 (PCB with one pyrrole ring less)
587.2879	1	587.2879	587.2879	0.0000	PCB

Table S92. Fragmentation (MS2 spectrum) of a molecular ion with m/z ratio 787.86 (z=2) from the MS spectrum of Fraction 7. Chromopeptide PAGITPGDCSA from C-PC β (P72508); PCB bound at Cys153.

m/z	z	Exp. mass (Da)	Theor. mass (Da)	Δ mass (Da)	Ion
169.0972	1	168.0894	168.0893	0.0001	b2
226.1188	1	225.111	225.1108	0.0002	b3
339.2038	1	338.196	338.1949	0.0011	b4
440.2505	1	439.2427	439.2425	0.0002	b5
464.218	1	464.218	464.2185	-0.0005	C26H30N3O5 (PCB with one pyrrole ring less)
587.2867	1	587.2867	587.2879	-0.0012	PCB

Table S93. Fragmentation (MS2 spectrum) of a molecular ion with m/z ratio 542.93 (z=3) from the MS spectrum of Fraction 7. Chromopeptide DKCARDIGY from C-PC α (P72509); PCB bound at Cys84.

m/z	z	Exp. mass (Da)	Theor. mass (Da)	Δ mass (Da)	Ion
797.3583	1	796.3505	796.3532	-0.0027	y7
859.4187	1	858.4109	858.4012	0.0097	b8
574.2777	1	573.2699	573.2688	0.0011	b5
464.2171	1	464.2171	464.2185	-0.0014	C26H30N3O5 (PCB with one pyrrole ring less)
587.2854	1	587.2854	587.2879	-0.0025	PCB

Table S94. Fragmentation (MS2 spectrum) of a molecular ion with m/z ratio 425.69 (z=2) from the MS spectrum of Fraction 8. Chromopeptide AAC from C-PC β (P72508); PCB bound at Cys82.

m/z	z	Exp. mass (Da)	Theor. mass (Da)	Δ mass (Da)	Ion
143.0815	1	142.0737	142.0737	0.0000	b2
193.0641	1	192.0563	192.0563	0.0000	y2
122.0271	1	121.0193	121.0192	0.0001	y1
464.2182	1	464.2182	464.2185	-0.0003	C26H30N3O5 (PCB with one pyrrole ring less)
587.2868	1	587.2868	587.2879	-0.0011	PCB

Table S95. Fragmentation (MS2 spectrum) of a molecular ion with m/z ratio 491.22 (z=2) from the MS spectrum of Fraction 8. Chromopeptide TATC from APC α (P72504); PCB bound at Cys81.

m/z	z	Exp. mass (Da)	Theor. mass (Da)	Δ mass (Da)	Ion
173.0925	1	172.0846	172.0842	0.0004	b2
274.1407	1	273.1328	273.1319	0.0009	b3
190.1187	1	189.1109	189.1108	0.0001	c2
223.0751	1	222.0672	222.0669	0.0003	y2
464.2187	1	464.2187	464.2185	0.0002	C26H30N3O5 (PCB with one pyrrole ring less)
587.2876	1	587.2876	587.2879	-0.0003	PCB

m/z	z	Exp. mass (Da)	Theor. mass (Da)	Δ mass (Da)	Ion

Table S96. Fragmentation (MS2 spectrum) of a molecular ion with m/z ratio 714.29 (z=2) from the MS spectrum of Fraction 9. Chromopeptide GEEMTATC from APC α (P72504); PCB bound at Cys81.

m/z	z	Exp. mass (Da)	Theor. mass (Da)	Δ mass (Da)	Ion
187.0716	1	186.0638	186.0635	0.0003	b2
316.1142	1	315.1064	315.1061	0.0003	b3
548.2031	1	547.1953	547.1943	0.001	b5
619.2527	1	618.2449	618.2314	0.0135	b6
464.2175	1	464.2175	464.2185	-0.001	C26H30N3O5 (PCB with one pyrrole ring less)
587.2860	1	587.2860	587.2879	-0.0019	PCB

Table S97. Fragmentation (MS2 spectrum) of a molecular ion with m/z ratio 576.26 (z=2) from the MS spectrum of Fraction 9. Chromopeptide GDCSAL from C-PC β (P72508); PCB bound at Cys153.

m/z	z	Exp. mass (Da)	Theor. mass (Da)	Δ mass (Da)	Ion
173.0558	1	172.048	172.0479	0.0001	b2
276.0653	1	275.0575	275.057	0.0005	b3
434.1348	1	433.127	433.1262	0.0008	b5
203.1395	1	202.1317	202.1312	0.0005	y2
464.2182	1	464.2182	464.2185	-0.0003	C26H30N3O5 (PCB with one pyrrole ring less)
587.2869	1	587.2869	587.2879	-0.0010	PCB

Table S98. Fragmentation (MS2 spectrum) of a molecular ion with m/z ratio 482.24 (z=2) from the MS spectrum of Fraction 10. Chromopeptide AACL from C-PC β (P72508); PCB bound at Cys82.

m/z	z	Exp. mass (Da)	Theor. mass (Da)	Δ mass (Da)	Ion
143.0815	1	142.0737	142.0737	0.0000	b2
246.0908	1	245.083	245.0829	0.0001	b3
132.1017	1	131.0939	131.0941	-0.0002	y1

m/z	z	Exp. mass (Da)	Theor. mass (Da)	Δ mass (Da)	Ion
235.1107	1	234.1029	234.1033	-0.0004	y2
464.2185	1	464.2185	464.2185	0.0000	C26H30N3O5 (PCB with one pyrrole ring less)
587.2874	1	587.2874	587.2879	-0.0005	PCB

Table S99. Fragmentation (MS2 spectrum) of a molecular ion with m/z ratio 390.18 (z=2) from the MS spectrum of Fraction 10. Chromopeptide AC from C-PC β (P72508); PCB bound at Cys82.

m/z	z	Exp. mass (Da)	Theor. mass (Da)	Δ mass (Da)	Ion
122.027	1	121.0192	121.0192	0.0000	y1
464.2175	1	464.2175	464.2185	-0.001	C26H30N3O5 (PCB with one pyrrole ring less)
587.2858	1	587.2858	587.2879	-0.0021	PCB

Table S100. Fragmentation (MS2 spectrum) of a molecular ion with m/z ratio 446.72 (z=2) from the MS spectrum of Fraction 11. Chromopeptide ACL from C-PC β (P72508); PCB bound at Cys82.

m/z	z	Exp. mass (Da)	Theor. mass (Da)	Δ mass (Da)	Ion
175.0536	1	174.0458	174.0458	0.0000	b2
235.1111	1	234.1033	234.1033	0.0000	y2
132.1019	1	131.0941	131.0941	0.0000	y1
464.2166	1	464.2166	464.2185	-0.0019	C26H30N3O5 (PCB with one pyrrole ring less)
587.2845	1	587.2845	587.2879	-0.0034	PCB

MS/MS² fragmentation — pepsin digestion, *Arthrospira platensis*

Table S101. Fragmentation (MS2 spectrum) of a molecular ion with m/z ratio 506.00 (z=4) from the MS spectrum of Fraction 1. Chromopeptide AADQRGKDKCARD from C-PC α (P72509); PCB bound at Cys84.

m/z	z	Exp. mass (Da)	Theor. mass (Da)	Δ mass (Da)	Ion
592.288	1	591.2802	591.2793	0.0009	y5
258.1091	1	257.1013	257.1006	0.0007	b3
707.3115	1	706.3037	706.3063	-0.0026	y6
361.1827	1	360.1749	360.1752	-0.0003	y3
464.2165	1	464.2165	464.2185	-0.002	C26H30N3O5 (PCB with one pyrrole ring less)
587.2866	1	587.2866	587.2879	0.0013	PCB

Table S102. Fragmentation (MS2 spectrum) of a molecular ion with m/z ratio 487.99 (z=4) from the MS spectrum of Fraction 1. Chromopeptide ADQRGKDKCARD from C-PC α (P72509); PCB bound at Cys84.

m/z	z	Exp. mass (Da)	Theor. mass (Da)	Δ mass (Da)	Ion
187.0714	1	186.0636	186.0635	0.0001	b2
328.6775	2	327.6696	327.6695	0.0001	b6
236.1193	2	235.1114	235.1113	0.0001	b4
296.6473	2	295.6395	295.6394	0.0001	y5
464.2182	1	464.2182	464.2185	-0.0003	C26H30N3O5 (PCB with one pyrrole ring less)
587.2871	1	587.2871	587.2879	-0.0008	PCB

Table S103. Fragmentation (MS2 spectrum) of a molecular ion with m/z ratio 675.31 (z=2) from the MS spectrum of Fraction 2. Chromopeptide ITPGDCSA or TPGDCSAL from C-PC β (P72508); PCB bound at Cys153.

m/z	z	Exp. mass (Da)	Theor. mass (Da)	Δ mass (Da)	Ion
549.1974	1	548.1896	548.1895	0.0001	y6

m/z	z	Exp. mass (Da)	Theor. mass (Da)	Δ mass (Da)	Ion
215.1391	1	214.1313	214.1312	0.0001	b2
650.2455	1	649.2377	649.2372	0.0005	y7
177.087	1	176.0792	176.0792	0.0000	y2
464.2183	1	464.2183	464.2185	-0.0002	C26H30N3O5 (PCB with one pyrrole ring less)
587.2866	1	587.2866	587.2879	-0.0013	PCB

Table S104. Fragmentation (MS2 spectrum) of a molecular ion with m/z ratio 596.27 (z=2) from the MS spectrum of Fraction 3. Chromopeptide TCLRD from APC α (P72504); PCB bound at Cys81.

m/z	z	Exp. mass (Da)	Theor. mass (Da)	Δ mass (Da)	Ion
506.2498	1	505.242	505.2313	0.0107	y4
290.1581	1	289.1503	289.1381	0.0122	y2
464.218	1	464.218	464.2185	-0.0005	C26H30N3O5 (PCB with one pyrrole ring less)
587.28647	1	587.28647	587.2879	-0.0032	PCB

Table S105. Fragmentation (MS2 spectrum) of a molecular ion with m/z ratio 787.37 (z=2) from the MS spectrum of Fraction 3. Chromopeptide PAGITPGDCSA from C-PC β (P72508); PCB bound at Cys153.

m/z	z	Exp. mass (Da)	Theor. mass (Da)	Δ mass (Da)	Ion
226.1188	1	225.1109	225.1108	0.0001	b3
169.0972	1	168.0894	168.0893	0.0001	b2
339.2029	1	338.1951	338.1949	0.0002	b4
440.2508	1	439.243	439.2425	0.0005	b5
464.2184	1	464.2184	464.2185	-0.0001	C26H30N3O5 (PCB with one pyrrole ring less)

m/z	z	Exp. mass (Da)	Theor. mass (Da)	Δ mass (Da)	Ion
587.28697	1	587.28697	587.2879	-0.0018	PCB

Table S106. Fragmentation (MS2 spectrum) of a molecular ion with m/z ratio 708.83 (z=2) from the MS spectrum of Fraction 4. Chromopeptide PAGITPGDC from C-PC β (P72508); PCB bound at Cys153.

m/z	z	Exp. mass (Da)	Theor. mass (Da)	Δ mass (Da)	Ion
226.1187	1	225.1108	225.1108	0.0000	b3
169.0972	1	168.0894	168.0893	0.0001	b2
339.2028	1	338.195	338.1949	0.0001	b4
440.2507	1	439.2429	439.2425	0.0004	b5
464.2183	1	464.2183	464.2185	-0.0002	C26H30N3O5 (PCB with one pyrrole ring less)
587.28674	1	587.28674	587.2879	-0.0005	PCB

Table S107. Fragmentation (MS2 spectrum) of a molecular ion with m/z ratio 844.37 (z=2) from the MS spectrum of Fraction 4. Chromopeptide DPAGITPGDCSA from C-PC β (P72508); PCB bound at Cys153.

m/z	z	Exp. mass (Da)	Theor. mass (Da)	Δ mass (Da)	Ion
341.1457	1	340.1379	340.1377	0.0002	b4
454.2297	1	453.2219	453.2218	0.0001	b5
284.1243	1	283.1164	283.1163	0.0001	b3
555.2775	1	554.2697	554.2695	0.0002	b6
464.2182	1	464.2182	464.2185	-0.0003	C26H30N3O5 (PCB with one pyrrole ring less)
587.2854	1	587.2854	587.2879	-0.0025	PCB

Table S108. Fragmentation (MS2 spectrum) of a molecular ion with m/z ratio 766.34 (z=2) from the MS spectrum of Fraction 5. Chromopeptide DPAGITPGDC from C-PC β (P72508); PCB bound at Cys153.

m/z	z	Exp. mass (Da)	Theor. mass (Da)	Δ mass (Da)	Ion
213.0872	1	212.0793	212.0792	0.0001	b2
237.0542	1	236.0463	236.0461	0.0002	y2

m/z	z	Exp. mass (Da)	Theor. mass (Da)	Δ mass (Da)	Ion
341.1458	1	340.138	340.1377	0.0003	b4
284.1244	1	283.1166	283.1163	0.0003	b3
297.1264	1	297.1264	297.1239	0.0025	C17H17N2O3 (PCB half)
464.2185	1	464.2185	464.2185	0.0000	C26H30N3O5 (PCB with one pyrrole ring less)
587.2870	1	587.2870	587.2879	-0.0009	PCB

Table S109. Fragmentation (MS2 spectrum) of a molecular ion with m/z ratio 491.22 (z=2) from the MS spectrum of Fraction 5. Chromopeptide TATC from APC α (P72504); PCB bound at Cys81.

m/z	z	Exp. mass (Da)	Theor. mass (Da)	Δ mass (Da)	Ion
173.0924	1	172.0845	172.0842	0.0003	b2
464.2194	1	464.2194	464.2185	0.0009	C26H30N3O5 (PCB with one pyrrole ring less)
587.28837	1	587.2883	587.2879	0.0004	PCB

Table S110. Fragmentation (MS2 spectrum) of a molecular ion with m/z ratio 490.23 (z=2) from the MS spectrum of Fraction 5. Chromopeptide CSAL from C-PC β (P72508); PCB bound at Cys153.

m/z	z	Exp. mass (Da)	Theor. mass (Da)	Δ mass (Da)	Ion
132.1019	1	131.0941	131.0941	0	y1
290.1582	1	289.1504	289.1632	-0.0128	y3
464.2182	1	464.2182	464.2185	-0.0003	C26H30N3O5 (PCB with one pyrrole ring less)
587.28637	1	587.2863	587.2879	-0.0016	PCB

Table S111. Fragmentation (MS2 spectrum) of a molecular ion with m/z ratio 482.24 (z=2) from the MS spectrum of Fraction 6. Chromopeptide AACL or AACI from APC β / C-PC β (P72505 / P72508); PCB bound at Cys81 / Cys82.

m/z	z	Exp. mass (Da)	Theor. mass (Da)	Δ mass (Da)	Ion
143.0815	1	142.0737	142.0737	0.0000	b2
132.1019	1	131.0941	131.0941	0.0000	y1
235.1112	1	234.1033	234.1033	0.0000	y2
246.0908	1	245.083	245.0829	0.0001	b3

m/z	z	Exp. mass (Da)	Theor. mass (Da)	Δ mass (Da)	Ion
464.2183	1	464.2183	464.2185	-0.0002	C26H30N3O5 (PCB with one pyrrole ring less)
587.28662	1	587.28662	587.2879	-0.0017	PCB

Table S112. Fragmentation (MS2 spectrum) of a molecular ion with m/z ratio 497.24 (z=2) from the MS spectrum of Fraction 6. Chromopeptide ATCL from APC α (P72504); PCB bound at Cys81.

m/z	z	Exp. mass (Da)	Theor. mass (Da)	Δ mass (Da)	Ion
173.0919	1	172.084	172.0842	-0.0002	b2
132.1018	1	131.094	131.0941	-0.0001	y1
235.1109	1	234.1031	234.1033	-0.0002	y2
336.1597	1	335.1519	335.1509	0.001	y3
464.2188	1	464.2188	464.2185	0.0003	C26H30N3O5 (PCB with one pyrrole ring less)
587.2866	1	587.2866	587.2879	-0.0013	PCB

Table S113. Fragmentation (MS2 spectrum) of a molecular ion with m/z ratio 390.18 (z=2) from the MS spectrum of Fraction 6. Chromopeptide CA or/and AC from APC β / C-PC α / C-PC β (P72505 / P72509 / P72508); PCB bound at Cys.

m/z	z	Exp. mass (Da)	Theor. mass (Da)	Δ mass (Da)	Ion
122.0271	1	121.0193	121.0198	-0.0005	y1 (AC)
90.055	1	89.0471	89.0472	-0.0001	y1 (CA)
464.2179	1	464.2179	464.2185	-0.0006	C26H30N3O5 (PCB with one pyrrole ring less)
587.2862	1	587.2862	587.2879	-0.0017	PCB

Table S114. Fragmentation (MS2 spectrum) of a molecular ion with m/z ratio 547.76 (z=2) from the MS spectrum of Fraction 6. Chromopeptide TATCL from APC α (P72504); PCB bound at Cys81.

m/z	z	Exp. mass (Da)	Theor. mass (Da)	Δ mass (Da)	Ion
132.1019	1	131.094	131.0941	-0.0001	y1
173.092	1	172.0842	172.0842	0.0000	b2
235.1112	1	234.1034	234.1033	0.0001	y2
274.1398	1	273.132	273.1319	0.0001	b3

m/z	z	Exp. mass (Da)	Theor. mass (Da)	Δ mass (Da)	Ion
464.218	1	464.218	464.2185	-0.0005	$C_{26}H_{30}N_3O_5$ (PCB with one pyrrole ring less)
587.2862	1	587.2862	587.2879	-0.0017	PCB
